# Imported Pet Reptiles and Their “Blind Passengers”—In-Depth Characterization of 80 *Acinetobacter* Species Isolates

**DOI:** 10.3390/microorganisms10050893

**Published:** 2022-04-24

**Authors:** Franziska Unger, Tobias Eisenberg, Ellen Prenger-Berninghoff, Ursula Leidner, Torsten Semmler, Christa Ewers

**Affiliations:** 1 Institute of Hygiene and Infectious Diseases of Animals, Faculty of Veterinary Medicine, Justus-Liebig University Giessen, 35392 Giessen, Germany; franziska.unger@vetmed.uni-giessen.de (F.U.); ellen.prenger-berninghoff@vetmed.uni-giessen.de (E.P.-B.); ursula.leidner@vetmed.uni-giessen.de (U.L.); 2 Hessian State Laboratory, 35392 Giessen, Germany; tobias.eisenberg@lhl.hessen.de; 3 NG1 Microbial Genomics, Robert Koch Institute, 13353 Berlin, Germany; semmlert@rki.de

**Keywords:** reptile, WGS, *Acinetobacter baumannii*, international clone, OXA, phylogeny, antimicrobial resistance

## Abstract

Reptiles are popular pet animals and important food sources, but the trade of this vertebrate class is—besides welfare and conservation—under debate due to zoonotic microbiota. Ninety-two shipments of live reptiles were sampled during border inspections at Europe’s most relevant transshipment point for the live animal trade. *Acinetobacter* spp. represented one significant fraction of potentially MDR bacteria that were further analyzed following non-selective isolation or selective enrichment from feces, urinate, or skin samples. Taxonomic positions of respective isolates were confirmed by MALDI-TOF MS and whole-genome sequencing analysis (GBDP, dDDH, ANIb, and rMLST). The majority of the 80 isolates represented established species; however, a proportion of potentially novel taxa was found. Antimicrobial properties and genome-resistance gene screening revealed novel and existing resistance mechanisms. *Acinetobacter* spp. strains were most often resistant to 6–10 substance groups (*n* = 63) in vitro. Resistance to fluorchinolones (*n* = 4) and colistin (*n* = 7), but not to carbapenems, was noted, and novel oxacillinase variants (*n* = 39) were detected among other genes. Phylogenetic analysis (MLST) assigned few isolates to the known STs (25, 46, 49, 220, and 249) and to a number of novel STs. No correlation was found to indicate that MDR *Acinetobacter* spp. in reptiles were associated with harvesting mode, e.g., captive-bred, wild-caught, or farmed in natural ecosystems. The community of *Acinetobacter* spp. in healthy reptiles turned out to be highly variable, with many isolates displaying a MDR phenotype or genotype.

## 1. Introduction

The genus *Acinetobacter* is highly diverse [1] and comprises, at the time of writing, approximately 73 validly published species (https://lpsn.dsmz.de/genus/acinetobacter (accessed on 8 February 2022)). Members of this genus are Gram-stain negative cocci or cocco-bacilli that are non-motile, non-spore-forming, strictly aerobic, cytochrome-oxidase negative, and catalase-positive bacteria [2]. Despite one of the genus’ major characteristics of being non-fermentative bacteria, members have been found to survive under different environmental conditions without oxygen for prolonged periods of several weeks. This is also true for *A. baumannii* [3], one of the most important nosocomial microorganisms that forms a species complex with the emerging opportunistic pathogens *A. calcoaceticus*, *A. pittii*, *A. nosocomialis,* and some other species [4] that are responsible for healthcare-associated outbreaks and severe infections, particularly in critically ill patients with impaired immunity [5,6]. *Acinetobacter* spp. are notorious for the accumulation of antibiotic resistance genes, which represents—together with other multiple drug resistant Gram-negative (MRGN) bacteria—an ever-increasing problem for global public health [6]. Furthermore, *A. baumannii* is referred to as an “ESKAPE pathogen”, an acronym that further includes *Enterococcus faecium*, *Staphylococcus aureus*, *Klebsiella pneumoniae*, *Pseudomonas aeruginosa*, and *Enterobacter* spp., as potentially life-threatening, multiple-drug resistant (MDR), and virulent nosocomial pathogens [7]. A high number of *Acinetobacter* spp. has originally been described from human clinical specimens [8]. However, other sources from the environment include activated sludge [9], wetlands [10], forest soil [11], seawater [12], dumpsites [13], manure and anaerobic digestates of biogas plants [3], wastewater [14], and freshwater [15].

From a veterinary perspective, *Acinetobacter* spp. are also frequently isolated from companion [16,17,18], zoo [19], wildlife animals [20,21] and livestock [22,23,24,25,26], and their role as nosocomial MRGNs has also been evaluated as critically important in animal clinics and patients suffering from, e.g., skin, wound, and systemic infections [27]. Direct zoonotic transmission chains seem to be rare, but companion animals represent a potential source for spillover in this regard [16,28,29,30]. Furthermore, *Acinetobacter* spp., particularly those colonizing livestock, were discussed as being adapted to certain animal reservoirs or as representing a potential source for food contamination, and can thus contribute to the colonization among humans and the contamination of their surroundings in the context of *One Health* issues [22,23,31,32,33,34]. 

Eventually, several studies also found *Acinetobacter* spp. in free-ranging, as well as in ex-situ-bred, members of the vertebrate class of reptiles [35,36], which might have implications with respect to their roles as important food sources in some countries [37] and as popular pet animals [38,39]. Possible spread events of MDR between reptiles and humans seem to be, at the least, probable since identical clones of ESBL-producing *Enterobacteriaceae* have recently been found in geckos living in close proximity to patients in a hospital in Ghana [40]. Our group has previously published preliminary findings on *mcr-1* positive *Escherichia coli* isolated from imported reptiles during border inspections [41]. We hypothesized that intemperance and amount of antimicrobial use in third countries might have an influence also on MDR microbiota in reptiles, especially when they are farmed in or near natural ecosystems. We here provide results on MDR of the fraction of *Acinetobacter* spp. that were isolated in a cross-sectional study evaluating 92 shipments of live reptiles, representing 160 batches of single species. Importations and samplings were carried out at the Frankfurt Airport, Germany, which represents the most relevant transshipment point for the trade of live animals worldwide [42].

## 2. Materials and Methods

### 2.1. Sample Collection

Between July 2013 and May 2014, samples from imported reptiles were drawn at the Frankfurt Airport, Germany. The sampling was carried out in cooperation with authorized veterinarians from the Border Control Post of the Frankfurt Airport during import control at the Frankfurt Animal Lounge directly after the arrival of the animals.

The investigated reptiles were assigned to the orders *Squamata* (lizards and snakes) and *Testudines* (turtles). The different shipments contained either one or several species that were transported in separate boxes, according to the guidelines of the International Air Transport Association (IATA). A total of 92 shipments were included, and each animal species per shipment was considered as one sample batch (*n* = 160), from which one or more samples were obtained (*n* = 183). Specimens collected were remnants of ecdysis (shed skin), feces (90% of the specimens), urinate (separate or mixed; *n* = 164) or swabs from the skin surface. The 92 shipments came from 23 different countries in Africa (Egypt, Madagascar, Mozambique, Uganda, South Africa, and the United Republic of Tanzania); North America (Canada and USA); Central and South America (Brazil, Ecuador, El Salvador, Guatemala, Guyana, Colombia, Nicaragua, and Panama); Asia (China, Japan, Turkey, Uzbekistan, and Vietnam), and Europe (Macedonia and Ukraine). The majority of shipments came from the USA (38.0%), Vietnam (10.9%), Uzbekistan (9.8%), Ukraine, and Canada (5.4% each).

The reptiles from this study could be assigned to different natural sources (Supplementary Table S1). A major proportion represented obviously captive-bred species (CB (33.8%); e.g., *Pogona vitticeps* from the USA). In a few cases, a clear assignment remained uncertain because captive breeding seemed principally possible, but not economical in large quantities and in the light of nearby, wild, autochthonous or allochthonous (i.e., neozoon) populations (presumably CB (3.2%); e.g., declared CB *Sceloporus malachiticus* from the USA that naturally occur in nearby Central America). Based on our experience, we assigned the remaining species as wild-caught (WC (28.3%); e.g., *Physignathus cocincinus* from Vietnam), farm-bred (FB (19.8%)); also including ranched species (reared in a controlled environment, taken as gravid females, eggs, or juveniles from the wild) that according to the CITES glossary [43] would “otherwise have had a low probability of surviving to adulthood”, and releasing a proportion back into the wild [26]. A minority of samples were obtained from species that could not unequivocally be assigned to one of the former groups and represented wild-caught or farm-bred/captive-bred (WC/FB (14.0%) or WC/CB (1.1%)) reptiles.

### 2.2. Bacterial Isolates and DNA Extraction

All samples were stored in sterile, fecal sample tubes at 4–7 °C until they were transported to the laboratory. Within no more than 48 h after sampling, fecal and urinate samples were directly streaked on blood agar (Merck, Darmstadt, Germany), supplemented with 5% sheep blood (SBA), water-blue-metachrome-yellow-lactose agar (Gassner), (Oxoid, Wesel, Germany), and on MacConkey agar (Oxoid) containing 1 mg/L cefotaxime (Sigma-Aldrich/Merck). Skin samples were re-suspended in 0.9% NaCl prior to cultivation, and 100 µL aliquots were streaked onto the same media. The plates were cultured for 24 h at 37 °C. In addition, all samples were cultivated in 3 mL, standard I nutrient broth (Roth GmbH + Co. KG, Karlsruhe, Germany), supplemented with a 10 µg meropenem disc (Mast Group Ltd., Reinfeld, Germany) for 24 h at 37 °C. In case of visible growth (at least slight turbidity of the broth), 50 µL of the solution was streaked onto SBA and Gassner agar and incubated for 24 h at 37 °C. Each sample was additionally inoculated into 5 mL of nutrient broth supplemented with bovine serum (cultivated at 37 °C for 24 h) as a kind of enrichment culture. Morphologically different colonies were again subcultured on SBA and Gassner agar, and presumptive Gram-negative bacteria, including putative *Acinetobacter* spp., were stored at −80 °C in a liquid nutrient broth containing 20% glycerol before they were used for further analysis. DNA was extracted with a “Master Pure™ DNA Purification Kit” (Biozym Scientific GmbH, Hessisch Oldendorf, Germany).

### 2.3. Matrix-Assisted Laser Desorption Ionization Time-of-Flight Mass Spectrometry (MALDI-TOF MS)

To identify *Acinetobacter* spp. among the presumptive Gram-negative isolates MALDI-TOF MS was used with isolates grown on SBA for 24 h. The workflow was performed as recently described [44]. Biomass was transferred to steel targets using the direct transfer protocol according to the manufacturer’s instructions (MALDI Biotyper; Bruker Daltonik, Bremen, Germany). Analysis was performed on a microflex LT mass spectrometer (Biotyper version 3.3.1.0 with MBT compass software). The database used (DB 9045 plus user-derived spectra of *A. pseudolwoffii* and *A. stercoris*-type strains) comprised 40 species and 154 strains of *Acinetobacter* spp. in total. The MALDI Biotyper real-time classification (RTC) software calculates log scores based on similarities between the observed results and stored database entries. Correct species-level identifications were assumed when the first- and second-best matches indicated the same species with log scores of >2.0, or when the best match indicated a species with a log score of >2.0, and the second-best match indicated a different species with a log score of <2.0 and consistency category A. The identification was done in duplicate to verify the original findings. Isolates identified as at least *Acinetobacter* spp. were stored at −80 °C for further investigations.

### 2.4. Antimicrobial Susceptibility Testing

All 80 non-duplicate *Acinetobacter* spp. isolates were screened for reduced susceptibility to different antimicrobial substances. Briefly, antimicrobial susceptibility testing was carried out using the broth microdilution susceptibility testing method. A commercially available panel layout for livestock (Micronaut/Bruker according to the guidelines of the working group for antimicrobial resistance of the German Veterinary Society, (DVG) was used. In this layout, 16 different antimicrobial substances (in µg/mL: amoxicillin/clavulanic acid (2/1–16/8), ampicillin (0.25–16), ceftiofur (0.125–4), cephalothin (1–16), colistin (0.5–2), enrofloxacin (0.016–1), erythromycin (0.125–4), florfenicol (1–8), gentamicin (0.125–8), penicillin G (0.063–8), spectinomycin (4–64), tetracycline (0.25–8), tiamulin (0.25–32), tilmicosin (0.5–16), trimethoprim/sulfamethoxazole (0.25/4.75–1/19), and tulathromycin (1–64))) were employed. Results were interpreted for human *Acinetobacter* species according to European Committee on Antimicrobial Susceptibility Testing (EUCAST, version 11.0) for broth dilution susceptibility testing and when clinical breakpoints were available.

### 2.5. Whole-Genome Sequencing Analysis and Bacterial Species Confirmation 

All isolates were subjected to whole-genome sequencing. Sequencing libraries were prepared using the Nextera XT Library Preparation Kit (Illumina GmbH, Munich, Germany) for a 250 bp paired-end sequencing run on an Illumina MiSeq sequencer (Illumina Inc., San Diego, CA, USA) with a minimum coverage of 100-fold. FASTQ files were quality trimmed before they were assembled de novo and annotated using SPAdes v.3.15.1 (http://cab.spbu.ru/software/spades/ (accessed on 10 September 2021)) and RAST v.2.0 (http://rast.nmpdr.org/ (accessed on 10 September 2021)).

Draft genome sequence data of 80 *Acinetobacter* spp. were uploaded to the Type (Strain) Genome Server (TYGS), a free bioinformatics platform available under https://tygs.dsmz.de (accessed on 8 February 2022), for a whole-genome-based taxonomic analysis [45]. The analysis also made use of recently introduced methodological updates and features [46]. Information on nomenclature, synonymy, and associated taxonomic literature was provided by the TYGS’s sister database, the List of Prokaryotic names with Standing in Nomenclature (LPSN, available at https://lpsn.dsmz.de (accessed on 8 February 2022)) [46]. The results were provided by the TYGS on 9 February 2022. The TYGS analysis was subdivided into the following steps:

Determination of the closest type strain genomes was done in two complementary ways: First, all user genomes were compared against all type strain genomes available in the TYGS database via the MASH algorithm, a fast approximation of intergenomic relatedness [47], and the 10 type strains with the smallest MASH distances were chosen per user genome. Second, an additional set of 10, closely related type strains was determined via the 16S rRNA gene sequences. These were extracted from the user genomes using RNAmmer [48], and each sequence was subsequently BLASTed [49] against the 16S rRNA gene sequence of each of the 15,873 type strains available in the TYGS database (as of 9 February 2022. This was used as a proxy to find the best 50 matching type strains (according to the bitscore) for each user genome, and to subsequently calculate precise distances using the Genome BLAST Distance Phylogeny approach (GBDP) under the algorithm “coverage” and distance formula (d5) [50]. These distances were finally used to determine the 10 closest type strain genomes for each of the user genomes.

For the phylogenomic inference, all pairwise comparisons among the set of genomes were conducted using GBDP and accurate intergenomic distances inferred under the algorithm “trimming” and distance formula (d5) [50]. A total of 100 distance replicates were calculated for each one. Digital DNA–DNA hybridization (dDDH) values and confidence intervals were calculated using the recommended settings of the GGDC 3.0 [46,50]. The resulting intergenomic distances were used to infer a balanced minimum evolution tree with branch support via FASTME 2.1.6.1, including SPR postprocessing [51]. Branch support was inferred from 100 pseudo-bootstrap replicates each. The trees were rooted at the midpoint [52] and visualized with PhyD3 [53].

The type-based species clustering using a 70% dDDH radius around each of the 79 type strains was performed as previously described. Draft genomes of the 80 *Acinetobacter* species were additionally uploaded to JSPeciesWS (Ribocon GmbH, Bremen, Germany, Version 3.9.0), an online platform for a BLAST-based measurement of the average nucleotide identity (ANIb), which contained 14,976 type strain genomes and a total of 55,565 genomes at the date of analysis (21 February 2022) [54]. Finally, we performed a ribosomal multilocus sequence typing (rMLST) analysis using the PubMLST.org website [55].

### 2.6. Antimicrobial Resistance Gene Screening 

The online platform tool ResFinder version 4.1, provided by the Center of Genomic and Epidemiology [56], was used to identify resistance genes based on whole-genome sequence data. In addition, we used the Resistance Gene Identifier (RGI) software of the Comprehensive Antibiotic Resistance Database to predict resistance genes [57].

### 2.7. Detection of Oxacillinase Genes and Phylogenetic Analysis

Nucleotide and amino acid sequences of intrinsic oxacillinases were used to query the NCBI nucleotide, genome, and protein databases using BLAST, implemented in Geneious (version 8.1.9, Biomatter Ltd., Auckland, New Zealand). A total of 970 *bla*_OXA_ sequences (available on 30 December 2021) were aligned with *bla*_OXA_ sequences from the reptile *A. baumannii* isolates. A maximum likelihood phylogeny of *bla*_OXA_ genes was estimated using FastTree. A translation of the nucleotide alignment was used to identify all OXAs that were different from currently known variants. Novel *bla*_OXA_ genes were submitted to GenBank.

### 2.8. Multilocus Sequence Typing of Acinetobacter Species and Assignment of A. baumannii Isolates to International Clones

Multilocus sequence types (STs) according to the MLST scheme developed by the Pasteur Institute [58] were deduced from whole-genome sequence data using PubMLST (https://pubmlst.org/bigsdb?db=pubmlst_abaumannii_seqdef (accessed on 1 October 2021)). This typing tool allows for the definition of STs for *A. baumannii* and for other *Acinetobacter* species. In the case that novel alleles and allele profiles were identified, they were submitted to the database curator to designate novel allele and ST numbers. International clones (IC) IC1 to IC3 were identified by multiplex-PCR [59]. In addition, a whole-genome-based comparison of all *A. baumannii* isolates from reptiles with representative genomes of IC1 to IC9 was performed to categorize the strains under study into the different clonal groups. In the case that isolates could not be assigned to an IC, their genomes were submitted to BacWGSTdb 2.0, a repository for bacterial whole-genome sequence typing and source tracking [60]. The tool “single genome analysis” was used to identify close isolates from the database based on a SNP strategy using a threshold of 1000 SNPs. The first three genomes that matched with our isolates were included in an SNP-based genome comparison.

## 3. Results

### 3.1. Sample Collection

Out of 160 sample batches, 183 specimens (162 fecal, 15 skin, 3 urinate, and 3 mixed fecal/urinate) were acquired. Based on MALDI-TOF MS analysis data, presumptive *Acinetobacter* spp. were present in 45.7% of 92 shipments, in 57 (35.6%) of 160 batches/sample units, and in 58 (31.7%) of 183 specimens. In accordance with the high number of shipments from the USA and some other countries, most of the 80 *Acinetobacter* spp. isolates were obtained from samples from the USA (46.25%); Ukraine (10.0%); Canada (8.75%); and Vietnam (7.5%); but also from South Africa (5.0%); El Salvador, Madagascar, and Nicaragua (3.75% each); Uzbekistan (2.5%); and Brazil, Ecuador, Japan, Colombia, Mozambique, the United Republic of Tanzania, and Uganda (1.25% each). *Acinetobacter* spp. isolates were retrieved from *Squamata* (77.5%) (83.9% lizards and 16.1% snakes) and from *Testudines* (22.5%) (100% turtles and tortoises). 

The reptiles from which the 80 *Acinetobacter* spp. were isolated represented both, obviously CB species, which accounted for 42.5% of the isolates (Table 1). Contrarily, 23.8% of the isolates were obtained from WC animals, followed by FB reptiles (21.3%). A small proportion of 10 isolates (12.5%) was gained from wild-caught or farm-bred/captive-bred (WC/FB or WC/CB) reptiles. 

### 3.2. Species Identification Based on MALDI-TOF MS Analysis and Whole-Genome Sequence Analysis

Using MALDI-TOF MS, 60 of the 80 isolates from this study could be assigned to the following species with unequivocal quality results (Supplementary Table S2): *A. baumannii* (*n* = 23), *A. bereziniae* (*n* = 4), *A. calcoaceticus* (*n* = 5), *A. courvalinii* (*n* = 1), *A. lactucae* (syn. *A. dijkshoorniae*) (*n* = 3), *A. indicus* (*n* = 1), *A. johnsonii* (*n* = 1), *A. nosocomialis* (*n* = 3), *A. pittii* (*n* = 12), *A. radioresistens* (*n* = 4), *A. schindleri* (*n* = 1), *A. seifertii* (*n* = 1), and *A. towneri* (*n* = 1). Twenty strains gave unreliable results with respect to a species-specific identification. 

As a countercheck to MALDI-TOF MS analysis, species IDs were investigated by different sequence-based analysis tools. The results are summarized in Supplementary Table S2. The species determination and dDDH values resulting from TYGS analysis revealed 62 isolates that were clearly assigned on a species level, while 18 isolates were identified as potentially new species (d4 value < 70.0%). The species clusters resulting from this analysis are additionally listed in Supplementary Table S3, whereas the taxonomic identification of the query strains is found in Supplementary Table S4. The clustering of non-*baumannii Acinetobacter* spp. isolates yielded 81 species clusters, and the provided query strains were assigned to 30 of these. The taxonomic placement of 57 non-*baumannii Acinetobacter* spp. isolates is shown in Supplementary Figure S1. 

Based on ANIb analysis, 70 isolates were reliably assigned to a species level, while 10 isolates revealed identity values below the threshold of 95%. Lastly, when using the rMLST species ID tool (https://pubmlst.org/species-id (accessed on 8 February 2022)), 62 isolates fulfilled the criteria for a clear species identification, while the genomes of 18 isolates where either below the cutoff value (≤95%) or showed percentages of similarity to reference strains of two different *Acinetobacter* species. 

Overall, congruent results by the different methods were only produced for 43 isolates (53.75%), including all 23 *A. baumannii* isolates. In the case that an isolate revealed congruent results with at least two of the methods, we assumed that as a reliable species identification. This was the case for 67 isolates (83.75%), in alphabetical order: *A. baumannii* (*n* = 23), *A. bereziniae* (*n* = 6), *A. calcoaceticus* (*n* = 2), *A. courvalinii* (*n* = 2), “*A. geminorum*” (*n* = 1; not validly published), *A. gerneri* (*n* = 1), *A. indicus* (*n* = 1), *A. lactucae* (*n* = 2), *A. nosocomialis* (*n* = 2), *A. oleivorans* (*n* = 2), *A. pittii* (*n* = 9), *A. radioresistens* (*n* = 4), *A. schindleri* (*n* = 2), *A. seifertii* (*n* = 2), *A. soli* (*n* = 1), *A. tandoii* (*n* = 1), *A. towneri* (*n* = 1), *A. variabilis* (*n* = 1), and *A. vivianii* (*n* = 4). Another 13 isolates showed incongruent results in at least three of the methods applied and were therefore defined as *A. lactucae*-like (*n* = 3), *A. pittii*-like (*n* = 2), *A. calcoaceticus*-like (*n* = 2), *A. courvalinii*-like (*n* = 3), *A. gerneri*-like (*n* = 1), *A. tandoii*-like (*n* = 1), and *A. johnsonii*-like (*n* = 1) (Table 1). 

### 3.3. Phenotypic Antimicrobial Resistance

Using the broth microdilution method in a commercial veterinary layout that includes 16 antimicrobial substances from 10 different substance groups, MIC values and—where available—clinical breakpoints (according to EUCAST v. 11.0) were obtained for all isolates in order to screen for MDR patterns. Most of the 80 *Acinetobacter* isolates under study showed high MIC values (in µg mL^−1^) as expected for penicillin (>8; *n* = 73), ampicillin (≥4; *n* = 73), amoxicillin/clavulanic acid (≥4/2; *n* = 71), cephalothin (≥16; *n* = 78), and ceftiofur (≥4; *n* = 72) (Supplementary Table S5). Mainly high MIC values were also found for florfenicol (≥4; *n* = 71), erythromycin (≥4; *n* = 74), tilmicosin (≥16; *n* = 74), tulathromycin (≥32; *n* = 68), tiamulin (≥32; *n* = 68), and spectinomycin (≥64; *n* = 62). Contrarily, the majority of strains displayed low MIC values for gentamicin (<4; *n* = 79), fluorchinolones (enrofloxacin <1; *n* = 75), colistin (≤2; *n* = 73), tetracycline (≤4; *n* = 66), and trimethoprim/sulfamethoxazole (≤2/38; *n* = 70). Irrespective of intrinsic resistances but rather based on high MIC values, *Acinetobacter* spp. strains were most often resistant to six substance groups (*n* = 49). A rather uncommon phenotype was observed in 9 strains with resistance to only 1–4 substance groups, the majority of which were characterized by low MIC values against penicillins (IHIT33475 (IHIT is an acronym for the German term for the Institute of Hygiene and Infectious Diseases of Animals), IHIT44651, IHIT44670, IHIT44680, and IHIT44681). A total of 14 strains were resistant against 7, 8, and 10 substance groups with *A. bereziniae* IHIT44655 (bearded dragon, Ukraine) showing the highest resistance phenotype. With respect to critically important antimicrobials, in vitro resistance to fluorchinolones and colistin are of particular interest. Whereas one of each strain of *A. towneri* (IHIT44651), *A. bereziniae* (IHIT44655), *A. schindleri* (IHIT44667), and *A. pittii* (IHIT44689) were resistant to enrofloxacin, three strains of *A. bereziniae* (IHIT44655, bearded dragon, Ukraine; IHIT44657, Turtle, USA; and IHIT44690, water dragon, Vietnam), one *A. gerneri* (IHIT44652, sand monitor, Vietnam), one *A. courvalinii* (IHIT44688, green spiny lizard, USA), one *A. oleivorans* (IHIT44694, sand monitor, Canada), and one *A. johnsonii*-like isolate (IHIT44693, sand monitor, Canada) showed colistin resistance in vitro.

### 3.4. Antimicrobial Resistance Genes

Among the 80 *Acinetobacter* spp. isolates under study, 9 (11.3%) carried *tet*(39) and 6 strains (7.5%) were positive for *tet*(B). A novel *tet*(X)-variant (*tet*(X5)-like) was identified in an *A. towneri* (IHIT44651) that was isolated from a red-footed tortoise from Colombia (Supplementary Table S5). Sulfonamide gene *sul2* was displayed by 11 strains (13.8%). Several genes encoding aminoglycoside-modifying enzymes were identified, however in low frequencies: *aac(6‘)-Ir* (7.5%), *strA*, *strB* and *aph(3‘)-III* (2.5% each), *aadB*, *aac(3)-IIa*, *aac(6‘)-Ia*, *aph(3‘)-IX*, and *aph(3‘)-X* (1.25% each). One strain of *A. schindleri* (IHIT44667) possessed the florfenicol resistance gene *floR*, whereas all of the *A. baumannii* isolates and nearly half (49.1%) of the 57 of non-*baumannii* isolates had the chromosomally encoded ADC cephalosporinase.

### 3.5. Intrinsic Oxacillinase Genes and Novel OXA Protein Variants

**Figure 1:**
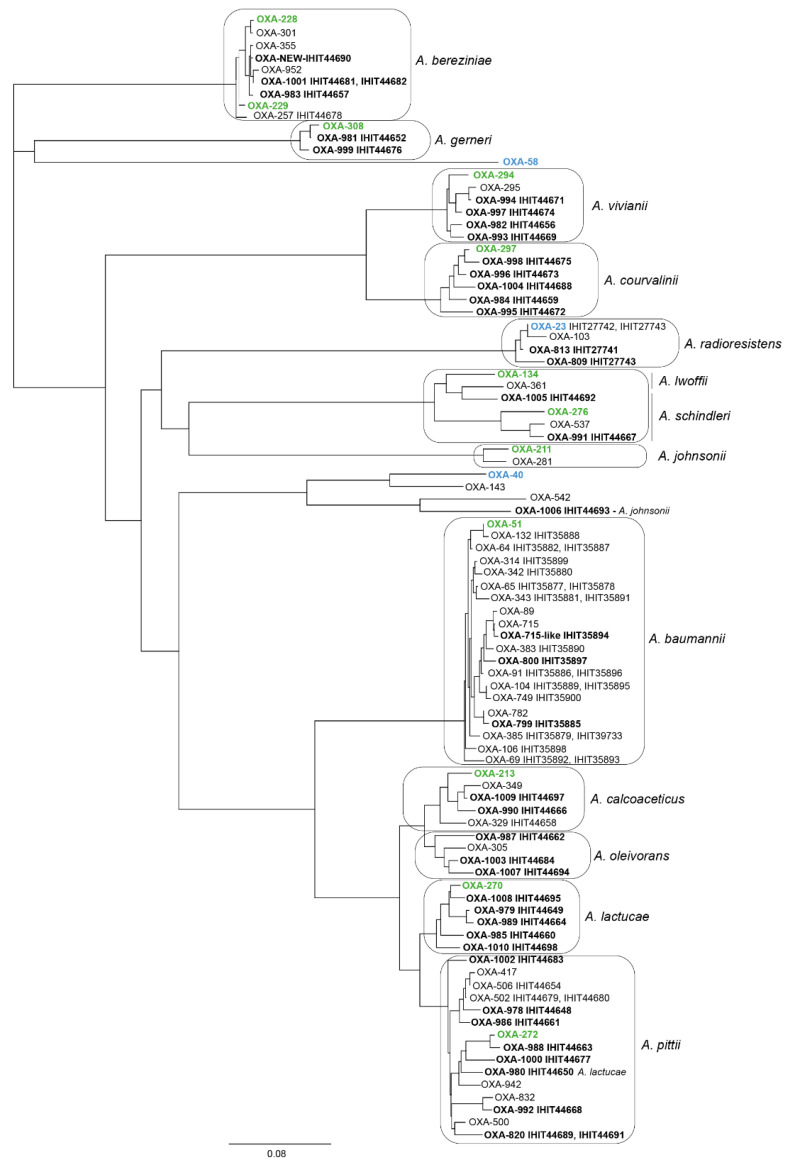
Phylogenetic tree based on a comparison of the amino acid sequences of 91 OXA variants from the NCBI database and from this study. Novel OXA-alleles identified in this study are written in black bold letters. OXA types typical for a certain *Acinetobacter* species are written in green. Grey frames indicate that OXA proteins clustered with the different *Acinetobacter* species. Protein sequences were aligned with MAFFT v7.017 [62] implemented in Geneious. The tree was built using the neighbor-joining method. The bar represents the percentage of differences in amino acids.

The diversity of the *bla*_OXA-like_ gene sequences from reptile *Acinetobacter* spp. isolates was high, not only between the different species, but also among isolates of the same bacterial species. A total of 70 strains (87.5%) carried an intrinsic oxacillinase gene with 19 known and 39 novel protein variants, designated as OXA-799, OXA-800, OXA-809, OXA-813, OXA-820, OXA-978 to OXA-1010, and OXA-1052 (Table 1, Supplementary Table S7). An additional OXA-allele variant with 99.3% amino acid similarity to OXA-715 of the OXA-51 family was found to be non-functional due to the introduction of an internal stop codon at position 42 (*A. baumannii* isolate IHIT35894). Based on a comparison of OXA amino acid sequences from reptile *Acinetobacter* spp. isolates and sequences available in the database, we identified the most-closely related OXA-alleles. They were included in a phylogenetic tree that was created based on aligned amino acid sequences (Figure 1). As expected, OXA-alleles basically clustered with OXA-type families and their variants that are intrinsic in the different *Acinetobacter* species, such as the OXA-51-like variant in *A. baumannii*, the OXA-272-like variant in *A. pittii*, or the OXA-23-like variant in *A. radioresistens*. For *A. baumannii*, it has been shown that the clonal affiliation of an isolate basically correlates well with specific variants of the *bla*_OXA-51-like_ gene [61]. Here, some of the OXA-variants did not follow the phylogeny of *A. baumannii*. For example, OXA-65 occurred in ST727 (IHIT35877; shipment 2/batch 5; sand monitor; USA) and ST1211 (IHIT35878; shipment 18/batch 23; green basilisk, USA), two sequence types that differ in all alleles of the seven-gene MLST^Past^ scheme. Also, other OXA-types, such as OXA-69 (ST1212 and ST1295), OXA-91 (ST1111 and ST1297), and OXA-104 (ST46 and ST1293) were identified in different STs that varied by 4 to 7 MLST alleles. This incongruence was also seen for some of the other *Acinetobacter* species, as shown in Supplementary Table S6.

### 3.6. MLST and International Clones

The 23 *A. baumannii* isolates belonged to 20 distinct STs according to the Pasteur scheme; there were 13 novel STs (Table 1, Supplementary Table S6). Two strains, isolated from a ball python from Canada (IHIT35882; shipment 33/batch 50) and a rainbow boa from the USA (IHIT35887, shipment 42/batch 64) belonged to the worldwide-distributed ST25, which belongs to IC7. Based on cgMLST analysis using the BacWGSTdb online tool and its data deposits, IHIT35887 particularly revealed a close relationship, differing by 62 to 139 alleles to ST25 isolates from other sources, such as IHIT38008 (dog, urine, Germany, 2018), AB24 (human, pus, Malaysia, 2012), 4300STDY7045893 (human, Thailand, 2016), and NM3 (human, sputum, United Arab Emirates, 2008). The second isolate, IHIT35882, revealed the lowest number of different alleles (136–266) to *A. baumannii* OCU_Ac2 (blood culture, hospitalized patient, Japan, 2014), OIFC143 (human, USA, 2003), IHIT38008 (urine, dog, Germany, 2018), and MRSN14237 (wound, human, Honduras, 2012).

**Figure 2:**
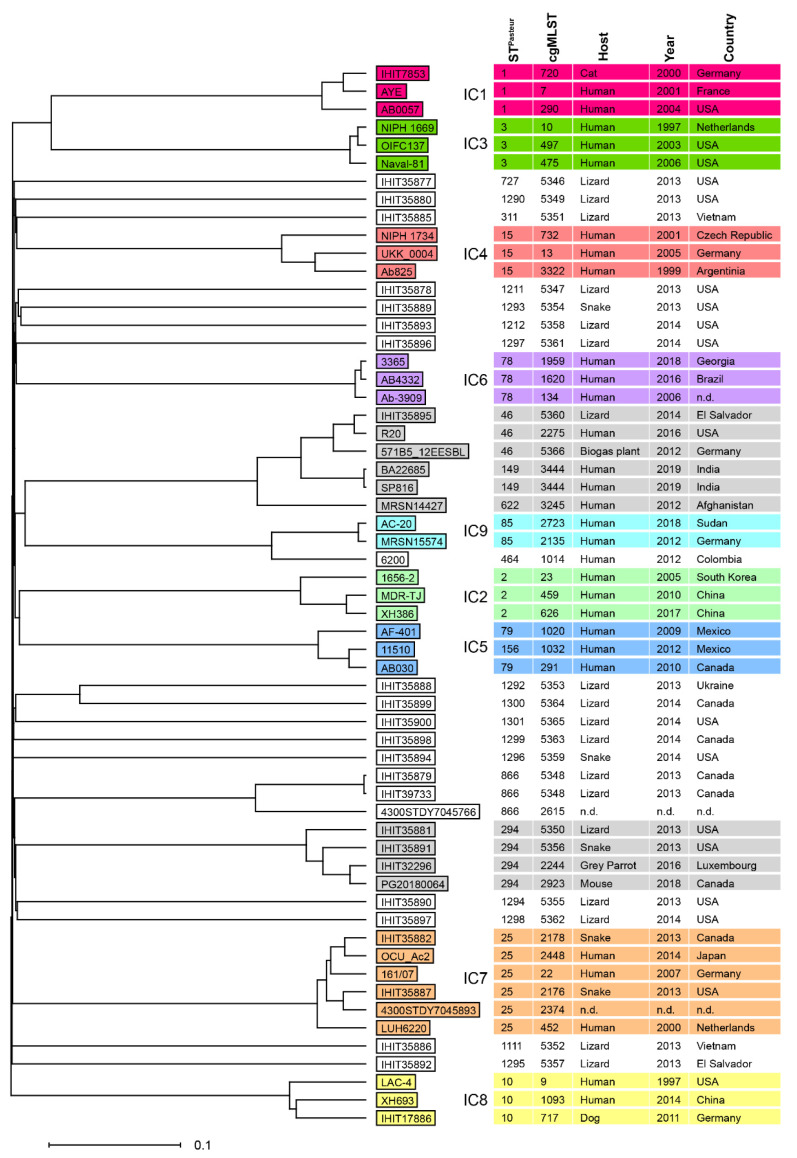
Phylogenetic tree based on the comparison of 2390 core genome genes of 23 *A. baumannii* from reptiles and 36 representative *A. baumannii* genomes of international clones IC1–IC9 (NCBI reference sequences are provided in Supplementary Table S7). Three *A. baumannii* isolates found to be closely related to non-IC1–IC9 reptile isolates (as determined by the online tool BacWGSTdb [60]) are additionally included. Groups of clustered isolates are shaded in different colors. Multilocus sequence types (ST^Past^), cgMLST cluster types, host, year, and country of isolates are indicated next to isolate numbers. The comparison was performed with Ridom SeqSphere+, and the tree was constructed with UPGMA, implemented in the software. The scale indicates the percentage of different cgMLST alleles. n.d. = not determined.

Based on the comparison of the core genome of reptile *A. baumannii* isolates and representative *A. baumannii* isolates of IC1–IC9, other ICs (other than IC7) could not be assigned to the reptile isolates (Figure 2). The majority of non-IC1–IC9 isolates were singletons for which no related genome could be found in the database (BacWGSTdb). However, ST294 isolates IHIT35881 (central bearded dragon, USA, shipment 27/batch 40) and IHIT35891 (rough green snake, USA, shipment 51/batch 80) clustered close to ST294 isolate PG20180064, which was isolated from a mouse in Canada in 2018) and to IHIT32296, an OXA-72-producing strain recently published by our group (grey parrot, Luxembourg, 2016) [63]. Also, ST46 isolate IHIT35895 (common green iguana, El Salvador) clustered together with genomes from the database, e.g., ST46 isolates 57185_12EESBL (biogas plant, Germany, 2012), R20 (human, USA, 2016), and ST149 isolates SP816 and BA22685 (both isolated from humans, India, 2019).

Non-baumannii *Acinetobacter* spp. isolates were mostly assigned to novel STs. Only *A. pittii* isolates IHIT44689 (yellow-headed gecko, Japan) and IHIT44691 (painted wood turtle, Nicaragua), belonged to the previously known ST220 that has been described for human clinical isolates in different countries, including carbapenemase-producing isolates in Japan [64] and Thailand [65].

## 4. Discussion

Although antimicrobial treatments are usually not carried out in wildlife animals, a growing number of reports have been published on MDR bacteria in wild and game animals. The spillover of antimicrobials and MDR bacteria into ecosystems and direct or indirect contact with MDR-shedders also seems to play a pivotal role in the acquisition of resistance genes in wildlife. In this regard, we hypothesized that poor hygienic and environmental conditions in habitats and breeding facilities might be correlated with increased rates of MDR bacteria and their global distribution in imported reptiles [66]. Very few studies have sampled reptiles directly at the point of entry in order to prevent bias of the detected microbiota from inland sources. Therefore, the aim of the current study was to evaluate a fraction of *Acinetobacter* spp. in healthy reptiles during importation at border inspection. Results on other microbiota from the same sampling have been reported [41] and will be published elsewhere. The Frankfurt Airport in Germany, is regarded as one of the most important hubs in animal importation, worldwide. In 2019, 306 shipments declared as ‘reptiles’ were registered for import into the European Union, and 93 shipments were sent in transit with a total number of 968,192 live reptiles imported from 21 third-party countries [67]. Although the importation of wild-caught animals has been widely banned, a considerable number of the imported species under study seemed to represent specimens that had been collected from natural resources (Supplementary Table S1), while the majority of species had been bred or raised in breeding facilities for the pet market and as laboratory animals. In the present study, 92 shipments containing live reptiles were tested for the presence of MDR bacteria, and the dataset of *Acinetobacter* spp. isolates has been further elucidated. Generally, *Acinetobacter* spp. are regarded as non-pathogenic bacteria in reptiles; some infections seem to be closely related to immune compromise [36]. This bacterial group may possess a highly diverse array of beta-lactamases that hydrolyze and confer resistance to penicillins, cephalosporins, and carbapenems [5,44]. This was in agreement with our study population since *Acinetobacter* spp. isolated from pet reptiles displayed in more than 60% of the isolates the chromosomally encoded ADC cephalosporinase. In addition, 87.5% of the isolates contained highly diverse intrinsic oxacillinase genes with 37 novel variants. There are several indications that typing of clinical isolates of *A. baumannii* belonging to ICs by MLST correlates well with specific OXA-51-like variants [21,61]. We have identified some OXA-51-like variants in the reptile *A. baumannii* isolates, such as OXA-64, OXA-65, and OXA-69, that are commonly found in the well-described clinical *A. baumannii* international clonal lineages IC7, IC5, and IC1. However, only the OXA-64 isolates clustered well with IC7 reference genomes, while we found no significant similarity between OXA-65 (IHIT35877 and IHIT35877; both from lizards from the USA) and OXA-69 isolates (IHIT35892 and IHIT35893; lizards, El Salvador and USA) and any of the clinical *A. baumannii* IC lineages (Figure 2). This, together with findings from others [21,25], indicates that MLST types and OXA-51-like variants are not strictly correlated. Notably, both studies observed this convergent evolution of *bla*_OXA-51-like_ genes among *A. baumannii* isolates from non-human sources, namely white storks in Poland and Germany, and livestock and food samples in Lebanon. We recommend further investigating the extent to which OXA proteins from animal sources may act as indicators of the clonal affiliation of *A. baumannii* isolates. 

Almost 20% of the reptile isolates simultaneously displayed reduced susceptibility or resistance to tetracyclines, which are encoded by two major groups of *tet* genes. The first group mediates energy-dependent efflux pumps for tetracyclines (*tet*(A), *tet*(B), *tet*(H), and *tet*(39)) and was represented by 21 isolates, whereas the second group confers resistance by ribosomal protection (*tet*(M)). *Acinetobacter towneri* isolate IHIT44651 (red-footed tortoise, Colombia) contained a *tet*(X3)-like gene associated with tigecycline resistance. However, the isolate demonstrated low MIC for tigecycline as determined by a MIC test strip (MIC 0.38 mg/L; Liofilchem Diagnostica). Other resistance patterns included aminoglycoside resistance genes in 18 strains, sulfonamide resistance in 11 strains, and a florfenicol resistance gene in *A. schindleri* (strain IHIT44667). The goal of the current study was to assess the presence of *Acinetobacter* species and other Gram-negative bacterial species (data not shown) of supposed pet reptiles during the process of importation. The magnitude and variability of resistance, assessed solely in one bacterial genus, *Acinetobacter*, suggests that imported reptiles are an underestimated source of resistant bacteria. In this regard, the vast majority (78.8%) of the study population showed phenotypical resistance to 3 (*n* = 2), 4 (*n* = 1), 5 (*n* = 7), 6 (*n* = 49), 7 (*n* = 1), 8 (*n* = 3), and 10 (*n* = 1) antimicrobial drug classes. Contrarily, resistance gene screening revealed evidence for only 0 (*n* = 3), 1 (*n* = 43), 2 (*n* = 20), 3 (*n* = 12), 4 (*n* = 1), and 5 (*n* = 1) groups of specific resistance genes, which can best be explained by as-yet-undetermined, intrinsic resistance mechanisms. Eventually, strains IHIT33475 and IHIT44676 both contained at least one *tet* resistance gene that was seemingly not expressed in vitro. None of the strains from the present study was found with phenotypical or molecular evidence for carbapenem resistance.

The SNP-based phylogeny, as well as the assessment of the MDR genotype, revealed several relationships to the resistant *Acinetobacter* spp. that have been isolated from other clinical sources. The two *A. baumannii* strains, IHIT35882 and IHIT35887 from a ball python (Canada) and a rainbow boa (USA), respectively, turned out to belong to ST25 (IC7), which represents a clonal lineage of clinically relevant strains with an MDR phenotype (including carbapenem resistance) that emerged on different continents over the last decade and was also reported in animals, including pets and wild birds [21,68,69,70,71]. One of our isolates was affiliated with a group of human clinical isolates which belonged to ST46 and related STs and which were not linked to a distinct ST. It remains to be seen if the worldwide, increasing number of *A. baumannii* genomes that is becoming available from different sources, including clinical, non-clinical, animal, and human sources, will lead to the establishment of novel *A. baumannii* ICs and a more complex understanding of the molecular epidemiology of this important pathogen. 

From a zoonotic perspective, imported reptiles are almost exclusively sold as pets and—contrarily to the situation in some of their home countries—are not utilized as food sources following importation. However, zoonotic bacteria, especially *Salmonella* [72], are well-known pathogens that frequently occur in reptiles and might—under inadequate hygienic conditions—be transferred to humans. The same situation must also be anticipated with MDR bacteria, and a spillover of these bacterial genes might occur via wastewater and terrarium soil, for example. Studies on findings of MDR bacteria in reptiles have increased in recent years. Among the reasons for conducting this kind of research were proofs for anthropogenic changes, including conservation issues, ecosystem health status, and environmental pollution [66,73,74]; veterinary aspects [75,76]; and possible zoonotic infections [40]. Most studies have been conducted to assess the health situation of reptiles in their ecosystems or when injured reptiles suffered from infection. Deems et al. [77] have analyzed a number of *Acinetobacter* species from the feces of wild painted turtles (*Chrysemys picta*). Although the authors also could not find strains displaying phenotypical carbapenem resistance, they demonstrated the capability of biodiesel degradation and biofilm formation in one *A. oleivorans* strain that possessed a putative type 6 gene cluster. However, as with other animal and herbal goods, the global trade does play a role in the dissemination of resistant bacteria in reptiles. Since imported live reptiles are not consumed as food items and are known to harbor a wide variety of possible zoonotic microorganisms, public health issues should focus on proper hygienic precautions in order to prevent human infections and the spread of MDR bacteria. The sole prohibition of reptile importation would most likely not prevent the spread since respective bacteria will be introduced by other routes, including humans themselves. Another study focused on investigating meat microbiota from recently imported reptiles and amphibians that are sold in specialty markets in Canada [37]. The authors found evidence for a further, highly virulent, NDM-1-producing *Acinetobacter* sp. that was isolated from a dried turtle carapace, suggesting that future studies should focus on the full diversity of genotypes as well as a comparison with captive-bred, pet reptiles. 

Interestingly, reptile β-defensins, toxin components, and peptides with antimicrobial and antibiofilm activity against *A. baumannii* and other MDR bacteria have been described as a component of the innate immune system in different species, which might represent promising treatment options, even in human infections, especially when outperforming human homologues [78,79,80,81,82,83]. Reptiles might also cope with facultative pathogenic bacterial loads by a higher activity of antimicrobial molecules that could, for instance, be found in snakes and water monitors inhabiting polluted environments [84].

In conclusion, the results from the present study have shown that imported, healthy pet reptiles represent another mosaic stone in the distribution pattern of *Acinetobacter* spp. Although the sole presence of these widely distributed bacteria in animal samples is not surprising, their discovery in every other shipment and the expression of a MDR phenotype in over 78% of the isolates should however, address future awareness on the fate of these lineages. With regard to the initial hypothesis, we could not confirm a higher load with MDR bacteria in reptiles from ‘antimicrobially polluted’ environments alone, but the strains with the highest resistance properties also seemed to be equally distributed in the group of supposed captive-bred species. Fortunately, the vast majority of *Acinetobacter* spp. isolated as “blind passengers” from pet reptiles proved susceptible to critically important antimicrobials, and there is currently no suggestion that reptile isolates represent a serious public health issue.

## Figures and Tables

**Table 1:** Animal origin, species designation, multilocus sequence types, and OXA types of 80 *Acinetobacter* spp. isolated in this study.

Strain ID	Species *	ST^Past^ **	OXA Type **	Sample			Animal ****		
				Shipment	Batch	Type ***	Species	Country	Origin
IHIT27741	*Ar*	2046	813	17	21	S	Jackson’s chameleon	USA	CB
IHIT27742	*Ar*	2053	23	46	69	F	Horsfield’s tortoise	UZB	FB
IHIT27743	*Ar*	2073	809	73	125	F	Fat-tail gecko	USA	CB
IHIT27744	*Ar*	2085	23	85	151	F	Horsfield’s tortoise	UKR	FB
IHIT33475	*Aind*	2061	neg.	60	100	F	Leopard tortoise	TZA	FB
IHIT35877	*Ab*	727	65	2	5	S	Sand monitor	USA	CB
IHIT35878	*Ab*	1211	65	18	23	S	Green basilisk	USA	FB
IHIT35879	*Ab*	866	385	21	27	F	Common leopard gecko	CAN	CB
IHIT35880	*Ab*	1290	342	23	30	F	Eastern collared lizard	USA	WC
IHIT35881	*Ab*	294	343	27	40	F	Central bearded dragon	USA	CB
IHIT35882	*Ab*	25	64	33	50	F	Ball python	CAN	CB
IHIT35884	*Aseif*	1291	neg.	37	54	S	Rough green snake	USA	WC
IHIT35885	*Ab*	311	799	38	55	F	Armored pricklenape	VNM	WC
IHIT35886	*Ab*	1111	91	39	62	F	Chinese water dragon	VNM	WC
IHIT35887	*Ab*	25	64	42	64	F	Rainbow boa	USA	CB
IHIT35888	*Ab*	1292	132	44	67	F	Madagascar day gecko	UKR	CB
IHIT35889	*Ab*	1293	104	50	76	F	Indigo snake	USA	pr. CB
IHIT35890	*Ab*	1294	383	50	77	F	Fat-tail gecko	USA	CB
IHIT35891	*Ab*	294	343	51	80	S	Rough green snake	USA	WC
IHIT35892	*Ab*	1295	69	52	81	F	Common green iguana	SLV	FB
IHIT35893	*Ab*	1212	69	58	95	F	Saw-scaled curly-tail	USA	WC/FB
IHIT35894	*Ab*	1296	#	70	118	F	Boa constrictor	USA	CB
IHIT35895	*Ab*	46	104	72	122	F	Common green iguana	SLV	FB
IHIT35896	*Ab*	1297	91	75	131	F	Green spiny lizard	USA	pr. CB
IHIT35897	*Ab*	1298	800	75	135	S	Savannah monitor	USA	CB
IHIT35898	*Ab*	1299	106	82	145	F	Sand monitor	CAN	CB
IHIT35899	*Ab*	1300	314	83	146	F	Common leopard gecko	CAN	CB
IHIT35900	*Ab*	1301	749	84	148	F	Common leopard gecko	USA	CB
IHIT39733	*Ab*	866	385	21	28	F	Crested gecko	CAN	CB
IHIT44648	*Ap*	2038	978	1	1	F	Green pricklenape	VNM	WC
IHIT44649	*Alac-like*	2047	979	18	25	F	Green spiny lizard	USA	pr. CB
IHIT44650	*Alac*	2048	980	27	40	F	Central bearded dragon	USA	CB
IHIT44651	*Atown*	2049	neg.	35	52	F	Red-footed tortoise	COL	WC/FB
IHIT44652	*Ager*	2050	981	39	60	F	Eastern garden lizard	VNM	WC
IHIT44653	*Anoso*	1269	neg.	39	60	F	Eastern garden lizard	VNM	WC
IHIT44654	*Ap*	2039	506	44	66	F	Central bearded dragon	UKR	CB
IHIT44655	*Aber*	2051	301	44	66	F	Central bearded dragon	UKR	CB
IHIT44656	*Aviv*	2052	982	44	66	F	Central bearded dragon	UKR	CB
IHIT44657	*Aber*	2054	983	48	72	F	Yellow mud turtle	USA	FB
IHIT44658	*Acalc*	2055	329	50	77	F	Fat-tail gecko	USA	CB
IHIT44659	*Acour*	2056	984	51	80	S	Rough green snake	USA	WC
IHIT44660	*Ap-like*	2057	985	51	80	S	Rough green snake	USA	WC
IHIT44661	*Ap-like*	2040	986	52	81	F	Common green iguana	SLV	FB
IHIT44662	*Aolei*	2058	987	53	82	F	Red-footed tortoise	BRA	WC/FB
IHIT44663	*Ap*	2041	988	58	93	F	Hispaniolan masked curly-tail	USA	WC/FB
IHIT44664	*Alac-like*	2059	989	58	95	F	Saw-scaled curly-tail	USA	WC/FB
IHIT44665	*Atan*	2060	neg.	58	97	F	Striped mud turtle	USA	FB
IHIT44666	*Acalc*	2062	990	64	110	F	East African black mud turtle	MOZ	WC
IHIT44667	*Aschin*	2063	991	65	111	F	Leopard tortoise	ECU	FB
IHIT44668	*Agem*	2064	992	66	113	F	Yellow-headed gecko	NIC	WC
IHIT44669	*Aviv*	2065	993	66	113	F	Yellow-headed gecko	NIC	WC
IHIT44670	*Avar*	2066	neg.	67	114	F	Horsfield’s tortoise	UZB	FB
IHIT44671	*Aviv*	2067	994	68	115	F	Jackson’s chameleon	UGA	CB
IHIT44672	*Acour*	2068	995	69	116	F	Cuvier’s Madagascar swift	MDG	WC
IHIT44673	*Acour*	2069	996	69	116	F	Cuvier’s Madagascar swift	MDG	WC
IHIT44674	*Aviv*	2070	997	69	117	F	Southeastern girdled lizard	MDG	WC
IHIT44675	*Acour*	2071	998	71	119	F	Rough green snake	USA	WC
IHIT44676	*Ager-like*	2072	999	73	124	F	Red corn snake	USA	CB
IHIT44677	*Ap*	2042	1000	73	125	F	Fat-tail gecko	USA	CB
IHIT44678	*Aber*	2074	257	73	126	F	New Caledonia giant gecko	USA	CB
IHIT44679	*Ap*	2043	502	73	126	F	New Caledonia giant gecko	USA	CB
IHIT44680	*Ap*	2044	502	74	128	F	Leopard tortoise	ZAF	FB
IHIT44681	*Aber*	2075	1001	74	128	F	Leopard tortoise	ZAF	FB
IHIT44682	*Aber*	2075	1001	74	128	F	Leopard tortoise	ZAF	FB
IHIT44683	*Ap*	2045	1002	74	128	F	Leopard tortoise	ZAF	FB
IHIT44684	*Aolei*	2076	1003	75	129	F	Cuban giant anole	USA	WC/FB
IHIT44685	*Aseif*	2077	neg.	75	130	F	Argentine black and white tegu	USA	CB
IHIT44686	*Atan-like*	n.d.	neg	75	130	F	Argentine black and white tegu	USA	CB
IHIT44687	*Anoso*	2078	neg	75	130	F	Argentine black and white tegu	USA	CB
IHIT44688	*Acour*	2079	1004	75	131	F	Green spiny lizard	USA	pr. CB
IHIT44689	*Ap*	220	820	76	136	F	Shingleback lizard	JPN	CB
IHIT44690	*Aber*	2080	1052	78	138	F	Chinese water dragon	VNM	WC
IHIT44691	*Ap*	220	820	79	142	F	Painted wood turtle	NIC	WC
IHIT44692	*Aschin*	2081	1005	80	143	F	Painted wood turtle	USA	pr. CB
IHIT44693	*Aj-like*	2082	1006	82	145	F	Sand monitor	CAN	CB
IHIT44694	*Aolei*	2083	1007	82	145	F	Sand monitor	CAN	CB
IHIT44695	*Alac-like*	2084	1008	84	148	F	Common leopard gecko	USA	CB
IHIT44696	*Asol*	2086	neg	87	154	F	Horsfield’s tortoise	UKR	FB
IHIT44697	*Acalc*	2087	1009	87	154	F	Horsfield’s tortoise	UKR	FB
IHIT44698	*Alac*	2088	1010	91	159	F	Central bearded dragon	UKR	CB

* *Ab* = *A. baumannii*, *Aber* = *A. bereziniae*, *Acalc* = *A. calcoaceticus*, *Acour* = *A. courvalinii*, *Agem* = *A. geminorum*, *Ager* = *A. gerneri*, *Aind* = *A. indicus*, *Aj* = *A. johsonii*, *Alac* = *A. lactucae*, *Anoso* = *A. nosocomialis*, *Aolei* = *A. oleivorans*, *Ap* = *A. pittii*, *Ar* = *A. radioresistens*, *Aschin* = *A. schindleri*, *Aseif* = *A. seifertii*, *Asol* = *A. soli*, *Atan* = *A. tandoii*, *Atown* = *A. towneri*, *Avari* = *A. variabilis*, and *Aviv* = *A. vivianii*. ** Sample type: F = feces; S = skin. *** Novel multilocus sequence types and novel OXA protein variants are underlined. **** CB = captive-bred, FB = farm-bred, WC = wild-caught, and pr. = presumably.

## Data Availability

Data supporting reported results can be found in the supplementary material and in the data repositories cited there.

## Supplementary Materials

The following supporting information can be downloaded at: https://www.mdpi.com/article/10.3390/microorganisms10050893/s1: Figure S1: Taxonomic placement of 57 non-*A. baumannii* members of the genus *Acinetobacter* isolated from reptiles, and of 79 automatically determined closest type strains available in the TYGS database. The tree was inferred with FastME 2.1.6.1 [51] from GBDP distances calculated from genome sequences. The branch lengths are scaled in terms of GBDP distance formula *d5*. The numbers above branches are GBDP pseudo-bootstrap support values >60% from 100 replications, with an average branch support of 70.2%. The tree was rooted at the midpoint [52]. Species names that are flanked by apostrophes represent *Acinetobacter* species that have not been validly published according to LPSN (https://lpsn.dsmz.de/ (accessed on 8 February 2022)). Table S1: Animal species sampled in this study—country origin and categorization as captive-bred (CB), farm-bred (FB), and wild-caught (WC). Table S2: Identification of 80 *Acinetobacter* species isolated from reptiles according to different taxonomic typing methods. Table S3: Joint dataset of automatically determined closest type strains and the 57 non-*A. baumannii* members of the genus *Acinetobacter* from this study. Table S4: Pairwise comparisons of the genomes of 57 non-*A. baumannii* members of the genus *Acinetobacter* vs. type strain genomes. Table S5: Antimicrobial susceptibility and resistance genes detected in 80 *Acinetobacter* species from reptiles. Table S6: Multilocus sequence types and OXA alleles determined for 80 *Acinetobacter* species isolates from reptiles. Table S7: NCBI reference numbers of *A. baumannii* genomes included in Figure 2 of the main text.
