# Supplementary material for: Imported Pet Reptiles and Their “Blind Passengers”—In-Depth Characterization of 80 Acinetobacter Species Isolates
Source: Microorganisms. 2022 Apr 24;10(5):893. doi: 10.3390/microorganisms10050893 (PMC9144363; doi:10.3390/microorganisms10050893)
Supplement: Supplementary file 1 [file microorganisms-10-00893-s001.zip › Suppl. Tab. S1_Animals sampled and categorization-20-03-21.pdf]

Table S1: Animal species sampled in this study - country origin and categorization as captive bred (CB), farm bred (FB) and wild-caught (WC)

Shipment No.	Sample batch	Animal species	Scientific name of animal/s	Order	Suborder	Country origin	Continent	Captive bred (CB), farm bred (FB), wild-caught (WC)	Web URL used for assigning animal species to categories CB, FB and WC
1	1	Green pricklenape	Acanthosaura capra	Squamata	Lizard	Vietnam	Asia	WC	https://reptile-database.reptarium.cz/species?genus=Acanthosaura&species=capra&search_param=%28%28common_name%3D%27Acanthosaura+capra%27%29%29
1	1	Green pricklenape	Acanthosaura capra	Squamata	Lizard	Vietnam	Asia	WC	https://reptile-database.reptarium.cz/species?genus=Acanthosaura&species=capra&search_param=%28%28common_name%3D%27Acanthosaura+capra%27%29%29
1	1	Green pricklenape	Acanthosaura capra	Squamata	Lizard	Vietnam	Asia	WC	https://reptile-database.reptarium.cz/species?genus=Acanthosaura&species=capra&search_param=%28%28common_name%3D%27Acanthosaura+capra%27%29%29
1	2	Chinese water dragon / Butterfly lizard	Physignatus cocincinus/Leiolepis belliana	Squamata	Lizard	Vietnam	Asia	WC	https://reptile-database.reptarium.cz/species?genus=Physignathus&species=cocincinus&search_param=%28%28common_name%3D%27gr%C3%BCne+wasseragame%27%29%29 , https://reptile-database.reptarium.cz/species?genus=Leiolepis&species=belliana&search_param=%28%28common_name%3D%27Leiolepis+belliana%27%29%29
1	2	Chinese water dragon / Butterfly lizard	Physignatus cocincinus/Leiolepis belliana	Squamata	Lizard	Vietnam	Asia	WC	https://reptile-database.reptarium.cz/species?genus=Physignathus&species=cocincinus&search_param=%28%28common_name%3D%27gr%C3%BCne+wasseragame%27%29%29 , https://reptile-database.reptarium.cz/species?genus=Leiolepis&species=belliana&search_param=%28%28common_name%3D%27Leiolepis+belliana%27%29%29
1	2	Chinese water dragon / Butterfly lizard	Physignatus cocincinus/Leiolepis belliana	Squamata	Lizard	Vietnam	Asia	WC	https://reptile-database.reptarium.cz/species?genus=Physignathus&species=cocincinus&search_param=%28%28common_name%3D%27gr%C3%BCne+wasseragame%27%29%29 , https://reptile-database.reptarium.cz/species?genus=Leiolepis&species=belliana&search_param=%28%28common_name%3D%27Leiolepis+belliana%27%29%29
1	3	Asian grass lizard	Takydromus sexlineatus	Squamata	Lizard	Vietnam	Asia	WC	https://reptile-database.reptarium.cz/species?genus=Takydromus&species=sexlineatus&search_param=%28%28common_name%3D%27Takydromus+sexlineatus%27%29%29
1	3	Asian grass lizard	Takydromus sexlineatus	Squamata	Lizard	Vietnam	Asia	WC	https://reptile-database.reptarium.cz/species?genus=Takydromus&species=sexlineatus&search_param=%28%28common_name%3D%27Takydromus+sexlineatus%27%29%29
1	3	Asian grass lizard	Takydromus sexlineatus	Squamata	Lizard	Vietnam	Asia	WC	https://reptile-database.reptarium.cz/species?genus=Takydromus&species=sexlineatus&search_param=%28%28common_name%3D%27Takydromus+sexlineatus%27%29%29
1	3	Asian grass lizard	Takydromus sexlineatus	Squamata	Lizard	Vietnam	Asia	WC	https://reptile-database.reptarium.cz/species?genus=Takydromus&species=sexlineatus&search_param=%28%28common_name%3D%27Takydromus+sexlineatus%27%29%29

Table S1: Animal species sampled in this study - country origin and categorization as captive bred (CB), farm bred (FB) and wild-caught (WC)

Shipment No.	Sample batch	Animal species	Scientific name of animal/s	Order	Suborder	Country origin	Continent	Captive bred (CB), farm bred (FB), wild-caught (WC)	Web URL used for assigning animal species to categories CB, FB and WC
1	3	Asian grass lizard	Takydromus sexlineatus	Squamata	Lizard	Vietnam	Asia	WC	https://reptile-database.reptarium.cz/species?genus=Takydromus&species=sexlineatus&search_param=%28%28common_name%3D%27Takydromus+sexlineatus%27%29%29
1	3	Asian grass lizard	Takydromus sexlineatus	Squamata	Lizard	Vietnam	Asia	WC	https://reptile-database.reptarium.cz/species?genus=Takydromus&species=sexlineatus&search_param=%28%28common_name%3D%27Takydromus+sexlineatus%27%29%29
1	3	Asian grass lizard	Takydromus sexlineatus	Squamata	Lizard	Vietnam	Asia	WC	https://reptile-database.reptarium.cz/species?genus=Takydromus&species=sexlineatus&search_param=%28%28common_name%3D%27Takydromus+sexlineatus%27%29%29
1	3	Asian grass lizard	Takydromus sexlineatus	Squamata	Lizard	Vietnam	Asia	WC	https://reptile-database.reptarium.cz/species?genus=Takydromus&species=sexlineatus&search_param=%28%28common_name%3D%27Takydromus+sexlineatus%27%29%29
1	pre-trial	Golden gecko / Tokay gecko	Gekko badenii/Gekko gecko	Squamata	Lizard	Vietnam	Asia	WC	https://reptile-database.reptarium.cz/species?genus=Gekko&species=badenii&search_param=%28%28common_name%3D%27golden+gecko%27%29%29 , https://reptile-database.reptarium.cz/species?genus=Gekko&species=grossmanni&search_param=%28%28common_name%3D%27gekko+grossmanni%27%29%29
1	4	Golden gecko / Tokay gecko	Gekko badenii/Gekko gecko	Squamata	Lizard	Vietnam	Asia	WC	https://reptile-database.reptarium.cz/species?genus=Gekko&species=badenii&search_param=%28%28common_name%3D%27golden+gecko%27%29%29 , https://reptile-database.reptarium.cz/species?genus=Gekko&species=grossmanni&search_param=%28%28common_name%3D%27gekko+grossmanni%27%29%29
1	4	Golden gecko / Tokay gecko	Gekko badenii/Gekko gecko	Squamata	Lizard	Vietnam	Asia	WC	https://reptile-database.reptarium.cz/species?genus=Gekko&species=badenii&search_param=%28%28common_name%3D%27golden+gecko%27%29%29 , https://reptile-database.reptarium.cz/species?genus=Gekko&species=grossmanni&search_param=%28%28common_name%3D%27gekko+grossmanni%27%29%29
1	4	Golden gecko / Tokay gecko	Gekko badenii/Gekko gecko	Squamata	Lizard	Vietnam	Asia	WC	https://reptile-database.reptarium.cz/species?genus=Gekko&species=badenii&search_param=%28%28common_name%3D%27golden+gecko%27%29%29 , https://reptile-database.reptarium.cz/species?genus=Gekko&species=grossmanni&search_param=%28%28common_name%3D%27gekko+grossmanni%27%29%29

Table S1: Animal species sampled in this study - country origin and categorization as captive bred (CB), farm bred (FB) and wild-caught (WC)

Shipment No.	Sample batch	Animal species	Scientific name of animal/s	Order	Suborder	Country origin	Continent	Captive bred (CB), farm bred (FB), wild-caught (WC)	Web URL used for assigning animal species to categories CB, FB and WC
1	4	Golden gecko / Tokay gecko	Gekko badenii/Gekko gecko	Squamata	Lizard	Vietnam	Asia	WC	https://reptile-database.reptarium.cz/species?genus=Gekko&species=badenii&search_param=%28%28common_name%3D%27golden+gecko%27%29%29 , https://reptile-database.reptarium.cz/species?genus=Gekko&species=grossmanni&search_param=%28%28common_name%3D%27gekko+grossmanni%27%29%29
1	2	Chinese water dragon / Butterfly lizard	Physignatus cocincinus/Leiolepis belliana	Squamata	Lizard	Vietnam	Asia	WC	https://reptile-database.reptarium.cz/species?genus=Physignathus&species=cocincinus&search_param=%28%28common_name%3D%27gr%C3%BCne+wasseragame%27%29%29 , https://reptile-database.reptarium.cz/species?genus=Leiolepis&species=belliana&search_param=%28%28common_name%3D%27Leiolepis+belliana%27%29%29
1	2	Chinese water dragon / Butterfly lizard	Physignatus cocincinus/Leiolepis belliana	Squamata	Lizard	Vietnam	Asia	WC	https://reptile-database.reptarium.cz/species?genus=Physignathus&species=cocincinus&search_param=%28%28common_name%3D%27gr%C3%BCne+wasseragame%27%29%29 , https://reptile-database.reptarium.cz/species?genus=Leiolepis&species=belliana&search_param=%28%28common_name%3D%27Leiolepis+belliana%27%29%29
1	2	Chinese water dragon / Butterfly lizard	Physignatus cocincinus/Leiolepis belliana	Squamata	Lizard	Vietnam	Asia	WC	https://reptile-database.reptarium.cz/species?genus=Physignathus&species=cocincinus&search_param=%28%28common_name%3D%27gr%C3%BCne+wasseragame%27%29%29 , https://reptile-database.reptarium.cz/species?genus=Leiolepis&species=belliana&search_param=%28%28common_name%3D%27Leiolepis+belliana%27%29%29
1	2	Chinese water dragon / Butterfly lizard	Physignatus cocincinus/Leiolepis belliana	Squamata	Lizard	Vietnam	Asia	WC	https://reptile-database.reptarium.cz/species?genus=Physignathus&species=cocincinus&search_param=%28%28common_name%3D%27gr%C3%BCne+wasseragame%27%29%29 , https://reptile-database.reptarium.cz/species?genus=Leiolepis&species=belliana&search_param=%28%28common_name%3D%27Leiolepis+belliana%27%29%29
1	2	Chinese water dragon / Butterfly lizard	Physignatus cocincinus/Leiolepis belliana	Squamata	Lizard	Vietnam	Asia	WC	https://reptile-database.reptarium.cz/species?genus=Physignathus&species=cocincinus&search_param=%28%28common_name%3D%27gr%C3%BCne+wasseragame%27%29%29 , https://reptile-database.reptarium.cz/species?genus=Leiolepis&species=belliana&search_param=%28%28common_name%3D%27Leiolepis+belliana%27%29%29
1	2	Chinese water dragon / Butterfly lizard	Physignatus cocincinus/Leiolepis belliana	Squamata	Lizard	Vietnam	Asia	WC	https://reptile-database.reptarium.cz/species?genus=Physignathus&species=cocincinus&search_param=%28%28common_name%3D%27gr%C3%BCne+wasseragame%27%29%29 , https://reptile-database.reptarium.cz/species?genus=Leiolepis&species=belliana&search_param=%28%28common_name%3D%27Leiolepis+belliana%27%29%29

Table S1: Animal species sampled in this study - country origin and categorization as captive bred (CB), farm bred (FB) and wild-caught (WC)

Shipment No.	Sample batch	Animal species	Scientific name of animal/s	Order	Suborder	Country origin	Continent	Captive bred (CB), farm bred (FB), wild-caught (WC)	Web URL used for assigning animal species to categories CB, FB and WC
2	5	Sand monitor	Varanus gouldii	Squamata	Lizard	USA	America	CB	https://reptile-database.reptarium.cz/species?genus=Varanus&species=gouldii&search_param=%28%28common_name%3D%27Varanus+gouldii%27%29%29
2	5	Sand monitor	Varanus gouldii	Squamata	Lizard	USA	America	CB	https://reptile-database.reptarium.cz/species?genus=Varanus&species=gouldii&search_param=%28%28common_name%3D%27Varanus+gouldii%27%29%29
2	pre-trial	Sand monitor	Varanus gouldii	Squamata	Lizard	USA	America	CB	https://reptile-database.reptarium.cz/species?genus=Varanus&species=gouldii&search_param=%28%28common_name%3D%27Varanus+gouldii%27%29%29
2	pre-trial	Sand monitor	Varanus gouldii	Squamata	Lizard	USA	America	CB	https://reptile-database.reptarium.cz/species?genus=Varanus&species=gouldii&search_param=%28%28common_name%3D%27Varanus+gouldii%27%29%29
2	pre-trial	Sand monitor	Varanus gouldii	Squamata	Lizard	USA	America	CB	https://reptile-database.reptarium.cz/species?genus=Varanus&species=gouldii&search_param=%28%28common_name%3D%27Varanus+gouldii%27%29%29
2	pre-trial	Sand monitor	Varanus gouldii	Squamata	Lizard	USA	America	CB	https://reptile-database.reptarium.cz/species?genus=Varanus&species=gouldii&search_param=%28%28common_name%3D%27Varanus+gouldii%27%29%29
2	pre-trial	Sand monitor	Varanus gouldii	Squamata	Lizard	USA	America	CB	https://reptile-database.reptarium.cz/species?genus=Varanus&species=gouldii&search_param=%28%28common_name%3D%27Varanus+gouldii%27%29%29
2	pre-trial	Sand monitor	Varanus gouldii	Squamata	Lizard	USA	America	CB	https://reptile-database.reptarium.cz/species?genus=Varanus&species=gouldii&search_param=%28%28common_name%3D%27Varanus+gouldii%27%29%29
2	pre-trial	Sand monitor	Varanus gouldii	Squamata	Lizard	USA	America	CB	https://reptile-database.reptarium.cz/species?genus=Varanus&species=gouldii&search_param=%28%28common_name%3D%27Varanus+gouldii%27%29%29
3	6	Mediterranean spur-thighed tortoise	Testudo graeca	Testudines	Turtle	Turkey	Asia	WC/FB	https://www.academia.edu/4668767/Testudo_trade_in_Turkey
3	6	Mediterranean spur-thighed tortoise	Testudo graeca	Testudines	Turtle	Turkey	Asia	WC/FB	https://www.academia.edu/4668767/Testudo_trade_in_Turkey
3	6	Mediterranean spur-thighed tortoise	Testudo graeca	Testudines	Turtle	Turkey	Asia	WC/FB	https://www.academia.edu/4668767/Testudo_trade_in_Turkey
3	6	Mediterranean spur-thighed tortoise	Testudo graeca	Testudines	Turtle	Turkey	Asia	WC/FB	https://www.academia.edu/4668767/Testudo_trade_in_Turkey
3	pre-trial	Mediterranean spur-thighed tortoise	Testudo graeca	Testudines	Turtle	Turkey	Asia	WC/FB	https://www.academia.edu/4668767/Testudo_trade_in_Turkey
3	pre-trial	Mediterranean spur-thighed tortoise	Testudo graeca	Testudines	Turtle	Turkey	Asia	WC/FB	https://www.academia.edu/4668767/Testudo_trade_in_Turkey

Table S1: Animal species sampled in this study - country origin and categorization as captive bred (CB), farm bred (FB) and wild-caught (WC)

Shipment No.	Sample batch	Animal species	Scientific name of animal/s	Order	Suborder	Country origin	Continent	Captive bred (CB), farm bred (FB), wild-caught (WC)	Web URL used for assigning animal species to categories CB, FB and WC
3	pre-trial	Mediterranean spur-thighed tortoise	Testudo graeca	Testudines	Turtle	Turkey	Asia	WC/FB	https://www.academia.edu/4668767/Testudo_trade_in_Turkey
3	pre-trial	Mediterranean spur-thighed tortoise	Testudo graeca	Testudines	Turtle	Turkey	Asia	WC/FB	https://www.academia.edu/4668767/Testudo_trade_in_Turkey
3	pre-trial	Mediterranean spur-thighed tortoise	Testudo graeca	Testudines	Turtle	Turkey	Asia	WC/FB	https://www.academia.edu/4668767/Testudo_trade_in_Turkey
3	pre-trial	Mediterranean spur-thighed tortoise	Testudo graeca	Testudines	Turtle	Turkey	Asia	WC/FB	https://www.academia.edu/4668767/Testudo_trade_in_Turkey
4	7	White-throated monitor	Varanus albigularis	Squamata	Lizard	Tanzania	Africa	WC/FB	https://reptile-database.reptarium.cz/species?genus=Varanus&species=albigularis&search_param=%28%28common_name%3D%27Varanus+albigularis%27%29%29
4	7	White-throated monitor	Varanus albigularis	Squamata	Lizard	Tanzania	Africa	WC/FB	https://reptile-database.reptarium.cz/species?genus=Varanus&species=albigularis&search_param=%28%28common_name%3D%27Varanus+albigularis%27%29%29
4	7	White-throated monitor	Varanus albigularis	Squamata	Lizard	Tanzania	Africa	WC/FB	https://reptile-database.reptarium.cz/species?genus=Varanus&species=albigularis&search_param=%28%28common_name%3D%27Varanus+albigularis%27%29%29
4	7	White-throated monitor	Varanus albigularis	Squamata	Lizard	Tanzania	Africa	WC/FB	https://reptile-database.reptarium.cz/species?genus=Varanus&species=albigularis&search_param=%28%28common_name%3D%27Varanus+albigularis%27%29%29
4	7	White-throated monitor	Varanus albigularis	Squamata	Lizard	Tanzania	Africa	WC/FB	https://reptile-database.reptarium.cz/species?genus=Varanus&species=albigularis&search_param=%28%28common_name%3D%27Varanus+albigularis%27%29%29
4	7	White-throated monitor	Varanus albigularis	Squamata	Lizard	Tanzania	Africa	WC/FB	https://reptile-database.reptarium.cz/species?genus=Varanus&species=albigularis&search_param=%28%28common_name%3D%27Varanus+albigularis%27%29%29
4	7	White-throated monitor	Varanus albigularis	Squamata	Lizard	Tanzania	Africa	WC/FB	https://reptile-database.reptarium.cz/species?genus=Varanus&species=albigularis&search_param=%28%28common_name%3D%27Varanus+albigularis%27%29%29
4	7	White-throated monitor	Varanus albigularis	Squamata	Lizard	Tanzania	Africa	WC/FB	https://reptile-database.reptarium.cz/species?genus=Varanus&species=albigularis&search_param=%28%28common_name%3D%27Varanus+albigularis%27%29%29
4	7	White-throated monitor	Varanus albigularis	Squamata	Lizard	Tanzania	Africa	WC/FB	https://reptile-database.reptarium.cz/species?genus=Varanus&species=albigularis&search_param=%28%28common_name%3D%27Varanus+albigularis%27%29%29
5	8	Horsefield's tortoise	Testudo horsfieldii	Testudines	Turtle	Uzbekistan	Asia	FB	https://reptile-database.reptarium.cz/species?genus=Testudo&species=horsfieldii&search_param=%28%28common_name%3D%27Testudo+horsfieldii%27%29%29

Table S1: Animal species sampled in this study - country origin and categorization as captive bred (CB), farm bred (FB) and wild-caught (WC)

Shipment No.	Sample batch	Animal species	Scientific name of animal/s	Order	Suborder	Country origin	Continent	Captive bred (CB), farm bred (FB), wild-caught (WC)	Web URL used for assigning animal species to categories CB, FB and WC
5	8	Horsefield's tortoise	Testudo horsfieldii	Testudines	Turtle	Uzbekistan	Asia	FB	https://reptile-database.reptarium.cz/species?genus=Testudo&species=horsfieldii&search_param=%28%28common_name%3D%27Testudo+horsfieldii%27%29%29
5	8	Horsefield's tortoise	Testudo horsfieldii	Testudines	Turtle	Uzbekistan	Asia	FB	https://reptile-database.reptarium.cz/species?genus=Testudo&species=horsfieldii&search_param=%28%28common_name%3D%27Testudo+horsfieldii%27%29%29
5	8	Horsefield's tortoise	Testudo horsfieldii	Testudines	Turtle	Uzbekistan	Asia	FB	https://reptile-database.reptarium.cz/species?genus=Testudo&species=horsfieldii&search_param=%28%28common_name%3D%27Testudo+horsfieldii%27%29%29
5	8	Horsefield's tortoise	Testudo horsfieldii	Testudines	Turtle	Uzbekistan	Asia	FB	https://reptile-database.reptarium.cz/species?genus=Testudo&species=horsfieldii&search_param=%28%28common_name%3D%27Testudo+horsfieldii%27%29%29
5	8	Horsefield's tortoise	Testudo horsfieldii	Testudines	Turtle	Uzbekistan	Asia	FB	https://reptile-database.reptarium.cz/species?genus=Testudo&species=horsfieldii&search_param=%28%28common_name%3D%27Testudo+horsfieldii%27%29%29
5	8	Horsefield's tortoise	Testudo horsfieldii	Testudines	Turtle	Uzbekistan	Asia	FB	https://reptile-database.reptarium.cz/species?genus=Testudo&species=horsfieldii&search_param=%28%28common_name%3D%27Testudo+horsfieldii%27%29%29
5	8	Horsefield's tortoise	Testudo horsfieldii	Testudines	Turtle	Uzbekistan	Asia	FB	https://reptile-database.reptarium.cz/species?genus=Testudo&species=horsfieldii&search_param=%28%28common_name%3D%27Testudo+horsfieldii%27%29%29
5	8	Horsefield's tortoise	Testudo horsfieldii	Testudines	Turtle	Uzbekistan	Asia	FB	https://reptile-database.reptarium.cz/species?genus=Testudo&species=horsfieldii&search_param=%28%28common_name%3D%27Testudo+horsfieldii%27%29%29
5	8	Horsefield's tortoise	Testudo horsfieldii	Testudines	Turtle	Uzbekistan	Asia	FB	https://reptile-database.reptarium.cz/species?genus=Testudo&species=horsfieldii&search_param=%28%28common_name%3D%27Testudo+horsfieldii%27%29%29
5	8	Horsefield's tortoise	Testudo horsfieldii	Testudines	Turtle	Uzbekistan	Asia	FB	https://reptile-database.reptarium.cz/species?genus=Testudo&species=horsfieldii&search_param=%28%28common_name%3D%27Testudo+horsfieldii%27%29%29
5	8	Horsefield's tortoise	Testudo horsfieldii	Testudines	Turtle	Uzbekistan	Asia	FB	https://reptile-database.reptarium.cz/species?genus=Testudo&species=horsfieldii&search_param=%28%28common_name%3D%27Testudo+horsfieldii%27%29%29
5	8	Horsefield's tortoise	Testudo horsfieldii	Testudines	Turtle	Uzbekistan	Asia	FB	https://reptile-database.reptarium.cz/species?genus=Testudo&species=horsfieldii&search_param=%28%28common_name%3D%27Testudo+horsfieldii%27%29%29

Table S1: Animal species sampled in this study - country origin and categorization as captive bred (CB), farm bred (FB) and wild-caught (WC)

Shipment No.	Sample batch	Animal species	Scientific name of animal/s	Order	Suborder	Country origin	Continent	Captive bred (CB), farm bred (FB), wild-caught (WC)	Web URL used for assigning animal species to categories CB, FB and WC
5	8	Horsefield's tortoise	Testudo horsfieldii	Testudines	Turtle	Uzbekistan	Asia	FB	https://reptile-database.reptarium.cz/species?genus=Testudo&species=horsfieldii&search_param=%28%28common_name%3D%27Testudo+horsfieldii%27%29%29
5	8	Horsefield's tortoise	Testudo horsfieldii	Testudines	Turtle	Uzbekistan	Asia	FB	https://reptile-database.reptarium.cz/species?genus=Testudo&species=horsfieldii&search_param=%28%28common_name%3D%27Testudo+horsfieldii%27%29%29
5	8	Horsefield's tortoise	Testudo horsfieldii	Testudines	Turtle	Uzbekistan	Asia	FB	https://reptile-database.reptarium.cz/species?genus=Testudo&species=horsfieldii&search_param=%28%28common_name%3D%27Testudo+horsfieldii%27%29%29
5	8	Horsefield's tortoise	Testudo horsfieldii	Testudines	Turtle	Uzbekistan	Asia	FB	https://reptile-database.reptarium.cz/species?genus=Testudo&species=horsfieldii&search_param=%28%28common_name%3D%27Testudo+horsfieldii%27%29%29
5	8	Horsefield's tortoise	Testudo horsfieldii	Testudines	Turtle	Uzbekistan	Asia	FB	https://reptile-database.reptarium.cz/species?genus=Testudo&species=horsfieldii&search_param=%28%28common_name%3D%27Testudo+horsfieldii%27%29%29
5	8	Horsefield's tortoise	Testudo horsfieldii	Testudines	Turtle	Uzbekistan	Asia	FB	https://reptile-database.reptarium.cz/species?genus=Testudo&species=horsfieldii&search_param=%28%28common_name%3D%27Testudo+horsfieldii%27%29%29
5	8	Horsefield's tortoise	Testudo horsfieldii	Testudines	Turtle	Uzbekistan	Asia	FB	https://reptile-database.reptarium.cz/species?genus=Testudo&species=horsfieldii&search_param=%28%28common_name%3D%27Testudo+horsfieldii%27%29%29
5	8	Horsefield's tortoise	Testudo horsfieldii	Testudines	Turtle	Uzbekistan	Asia	FB	https://reptile-database.reptarium.cz/species?genus=Testudo&species=horsfieldii&search_param=%28%28common_name%3D%27Testudo+horsfieldii%27%29%29
5	8	Horsefield's tortoise	Testudo horsfieldii	Testudines	Turtle	Uzbekistan	Asia	FB	https://reptile-database.reptarium.cz/species?genus=Testudo&species=horsfieldii&search_param=%28%28common_name%3D%27Testudo+horsfieldii%27%29%29
6	9	Red-footed tortoise	Chelonoidis carbonarius	Testudines	Turtle	Colombia	America	WC/FB	https://reptile-database.reptarium.cz/species?genus=Chelonoidis&species=carbonarius&search_param=%28%28common_name%3D%27Chelonoidis+carbonarius%27%29%29
6	9	Red-footed tortoise	Chelonoidis carbonarius	Testudines	Turtle	Colombia	America	WC/FB	https://reptile-database.reptarium.cz/species?genus=Chelonoidis&species=carbonarius&search_param=%28%28common_name%3D%27Chelonoidis+carbonarius%27%29%29
6	9	Red-footed tortoise	Chelonoidis carbonarius	Testudines	Turtle	Colombia	America	WC/FB	https://reptile-database.reptarium.cz/species?genus=Chelonoidis&species=carbonarius&search_param=%28%28common_name%3D%27Chelonoidis+carbonarius%27%29%29

Table S1: Animal species sampled in this study - country origin and categorization as captive bred (CB), farm bred (FB) and wild-caught (WC)

Shipment No.	Sample batch	Animal species	Scientific name of animal/s	Order	Suborder	Country origin	Continent	Captive bred (CB), farm bred (FB), wild-caught (WC)	Web URL used for assigning animal species to categories CB, FB and WC
6	9	Red-footed tortoise	Chelonoidis carbonarius	Testudines	Turtle	Colombia	America	WC/FB	https://reptile-database.reptarium.cz/species?genus=Chelonoidis&species=carbonarius&search_param=%28%28common_name%3D%27Chelonoidis+carbonarius%27%29%29
6	9	Red-footed tortoise	Chelonoidis carbonarius	Testudines	Turtle	Colombia	America	WC/FB	https://reptile-database.reptarium.cz/species?genus=Chelonoidis&species=carbonarius&search_param=%28%28common_name%3D%27Chelonoidis+carbonarius%27%29%29
6	9	Red-footed tortoise	Chelonoidis carbonarius	Testudines	Turtle	Colombia	America	WC/FB	https://reptile-database.reptarium.cz/species?genus=Chelonoidis&species=carbonarius&search_param=%28%28common_name%3D%27Chelonoidis+carbonarius%27%29%29
6	9	Red-footed tortoise	Chelonoidis carbonarius	Testudines	Turtle	Colombia	America	WC/FB	https://reptile-database.reptarium.cz/species?genus=Chelonoidis&species=carbonarius&search_param=%28%28common_name%3D%27Chelonoidis+carbonarius%27%29%29
6	9	Red-footed tortoise	Chelonoidis carbonarius	Testudines	Turtle	Colombia	America	WC/FB	https://reptile-database.reptarium.cz/species?genus=Chelonoidis&species=carbonarius&search_param=%28%28common_name%3D%27Chelonoidis+carbonarius%27%29%29
6	9	Red-footed tortoise	Chelonoidis carbonarius	Testudines	Turtle	Colombia	America	WC/FB	https://reptile-database.reptarium.cz/species?genus=Chelonoidis&species=carbonarius&search_param=%28%28common_name%3D%27Chelonoidis+carbonarius%27%29%29
6	9	Red-footed tortoise	Chelonoidis carbonarius	Testudines	Turtle	Colombia	America	WC/FB	https://reptile-database.reptarium.cz/species?genus=Chelonoidis&species=carbonarius&search_param=%28%28common_name%3D%27Chelonoidis+carbonarius%27%29%29
6	9	Red-footed tortoise	Chelonoidis carbonarius	Testudines	Turtle	Colombia	America	WC/FB	https://reptile-database.reptarium.cz/species?genus=Chelonoidis&species=carbonarius&search_param=%28%28common_name%3D%27Chelonoidis+carbonarius%27%29%29
7	pre-trial	Peninsula cooter	Pseudemys peninsularis	Testudines	Turtle	USA	America	FB	https://reptile-database.reptarium.cz/species?genus=Pseudemys&species=peninsularis&search_param=%28%28common_name%3D%27Pseudemys+peninsularis%27%29%29
7	pre-trial	Peninsula cooter	Pseudemys peninsularis	Testudines	Turtle	USA	America	FB	https://reptile-database.reptarium.cz/species?genus=Pseudemys&species=peninsularis&search_param=%28%28common_name%3D%27Pseudemys+peninsularis%27%29%29
7	pre-trial	Peninsula cooter	Pseudemys peninsularis	Testudines	Turtle	USA	America	FB	https://reptile-database.reptarium.cz/species?genus=Pseudemys&species=peninsularis&search_param=%28%28common_name%3D%27Pseudemys+peninsularis%27%29%29
7	pre-trial	Peninsula cooter	Pseudemys peninsularis	Testudines	Turtle	USA	America	FB	https://reptile-database.reptarium.cz/species?genus=Pseudemys&species=peninsularis&search_param=%28%28common_name%3D%27Pseudemys+peninsularis%27%29%29

Table S1: Animal species sampled in this study - country origin and categorization as captive bred (CB), farm bred (FB) and wild-caught (WC)

Shipment No.	Sample batch	Animal species	Scientific name of animal/s	Order	Suborder	Country origin	Continent	Captive bred (CB), farm bred (FB), wild-caught (WC)	Web URL used for assigning animal species to categories CB, FB and WC
7	pre-trial	Peninsula cooter	Pseudemys peninsularis	Testudines	Turtle	USA	America	FB	https://reptile-database.reptarium.cz/species?genus=Pseudemys&species=peninsularis&search_param=%28%28common_name%3D%27Pseudemys+peninsularis%27%29%29
8	10	Forest garden lizard / Chinese water dragon	Calotes emma/Physignatus cocincinus	Squamata	Lizard	Vietnam	Asia	WC	https://reptile-database.reptarium.cz/species?genus=Calotes&species=emma&search_param=%28%28common_name%3D%27Calotes+emma%27%29%29 , https://reptile-database.reptarium.cz/species?genus=Physignathus&species=cocincinus&search_param=%28%28common_name%3D%27Physignatus+cocincinus%27%29%29
8	10	Forest garden lizard / Chinese water dragon	Calotes emma/Physignatus cocincinus	Squamata	Lizard	Vietnam	Asia	WC	https://reptile-database.reptarium.cz/species?genus=Calotes&species=emma&search_param=%28%28common_name%3D%27Calotes+emma%27%29%29 , https://reptile-database.reptarium.cz/species?genus=Physignathus&species=cocincinus&search_param=%28%28common_name%3D%27Physignatus+cocincinus%27%29%29
8	10	Forest garden lizard / Chinese water dragon	Calotes emma/Physignatus cocincinus	Squamata	Lizard	Vietnam	Asia	WC	https://reptile-database.reptarium.cz/species?genus=Calotes&species=emma&search_param=%28%28common_name%3D%27Calotes+emma%27%29%29 , https://reptile-database.reptarium.cz/species?genus=Physignathus&species=cocincinus&search_param=%28%28common_name%3D%27Physignatus+cocincinus%27%29%29
8	10	Forest garden lizard / Chinese water dragon	Calotes emma/Physignatus cocincinus	Squamata	Lizard	Vietnam	Asia	WC	https://reptile-database.reptarium.cz/species?genus=Calotes&species=emma&search_param=%28%28common_name%3D%27Calotes+emma%27%29%29 , https://reptile-database.reptarium.cz/species?genus=Physignathus&species=cocincinus&search_param=%28%28common_name%3D%27Physignatus+cocincinus%27%29%29
8	10	Forest garden lizard / Chinese water dragon	Calotes emma/Physignatus cocincinus	Squamata	Lizard	Vietnam	Asia	WC	https://reptile-database.reptarium.cz/species?genus=Calotes&species=emma&search_param=%28%28common_name%3D%27Calotes+emma%27%29%29 , https://reptile-database.reptarium.cz/species?genus=Physignathus&species=cocincinus&search_param=%28%28common_name%3D%27Physignatus+cocincinus%27%29%29
8	10	Forest garden lizard / Chinese water dragon	Calotes emma/Physignatus cocincinus	Squamata	Lizard	Vietnam	Asia	WC	https://reptile-database.reptarium.cz/species?genus=Calotes&species=emma&search_param=%28%28common_name%3D%27Calotes+emma%27%29%29 , https://reptile-database.reptarium.cz/species?genus=Physignathus&species=cocincinus&search_param=%28%28common_name%3D%27Physignatus+cocincinus%27%29%29

Table S1: Animal species sampled in this study - country origin and categorization as captive bred (CB), farm bred (FB) and wild-caught (WC)

Shipment No.	Sample batch	Animal species	Scientific name of animal/s	Order	Suborder	Country origin	Continent	Captive bred (CB), farm bred (FB), wild-caught (WC)	Web URL used for assigning animal species to categories CB, FB and WC
8	10	Forest garden lizard / Chinese water dragon	Calotes emma/Physignatus cocincinus	Squamata	Lizard	Vietnam	Asia	WC	https://reptile-database.reptarium.cz/species?genus=Calotes&species=emma&search_param=%28%28common_name%3D%27Calotes+emma%27%29%29 , https://reptile-database.reptarium.cz/species?genus=Physignathus&species=cocincinus&search_param=%28%28common_name%3D%27Physignatus+cocincinus%27%29%29
8	10	Forest garden lizard / Chinese water dragon	Calotes emma/Physignatus cocincinus	Squamata	Lizard	Vietnam	Asia	WC	https://reptile-database.reptarium.cz/species?genus=Calotes&species=emma&search_param=%28%28common_name%3D%27Calotes+emma%27%29%29 , https://reptile-database.reptarium.cz/species?genus=Physignathus&species=cocincinus&search_param=%28%28common_name%3D%27Physignatus+cocincinus%27%29%29
8	10	Forest garden lizard / Chinese water dragon	Calotes emma/Physignatus cocincinus	Squamata	Lizard	Vietnam	Asia	WC	https://reptile-database.reptarium.cz/species?genus=Calotes&species=emma&search_param=%28%28common_name%3D%27Calotes+emma%27%29%29 , https://reptile-database.reptarium.cz/species?genus=Physignathus&species=cocincinus&search_param=%28%28common_name%3D%27Physignatus+cocincinus%27%29%29
8	10	Forest garden lizard / Chinese water dragon	Calotes emma/Physignatus cocincinus	Squamata	Lizard	Vietnam	Asia	WC	https://reptile-database.reptarium.cz/species?genus=Calotes&species=emma&search_param=%28%28common_name%3D%27Calotes+emma%27%29%29 , https://reptile-database.reptarium.cz/species?genus=Physignathus&species=cocincinus&search_param=%28%28common_name%3D%27Physignatus+cocincinus%27%29%29
8	10	Forest garden lizard / Chinese water dragon	Calotes emma/Physignatus cocincinus	Squamata	Lizard	Vietnam	Asia	WC	https://reptile-database.reptarium.cz/species?genus=Calotes&species=emma&search_param=%28%28common_name%3D%27Calotes+emma%27%29%29 , https://reptile-database.reptarium.cz/species?genus=Physignathus&species=cocincinus&search_param=%28%28common_name%3D%27Physignatus+cocincinus%27%29%29
8	10	Forest garden lizard / Chinese water dragon	Calotes emma/Physignatus cocincinus	Squamata	Lizard	Vietnam	Asia	WC	https://reptile-database.reptarium.cz/species?genus=Calotes&species=emma&search_param=%28%28common_name%3D%27Calotes+emma%27%29%29 , https://reptile-database.reptarium.cz/species?genus=Physignathus&species=cocincinus&search_param=%28%28common_name%3D%27Physignatus+cocincinus%27%29%29
8	10	Forest garden lizard / Chinese water dragon	Calotes emma/Physignatus cocincinus	Squamata	Lizard	Vietnam	Asia	WC	https://reptile-database.reptarium.cz/species?genus=Calotes&species=emma&search_param=%28%28common_name%3D%27Calotes+emma%27%29%29 , https://reptile-database.reptarium.cz/species?genus=Physignathus&species=cocincinus&search_param=%28%28common_name%3D%27Physignatus+cocincinus%27%29%29

Table S1: Animal species sampled in this study - country origin and categorization as captive bred (CB), farm bred (FB) and wild-caught (WC)

Shipment No.	Sample batch	Animal species	Scientific name of animal/s	Order	Suborder	Country origin	Continent	Captive bred (CB), farm bred (FB), wild-caught (WC)	Web URL used for assigning animal species to categories CB, FB and WC
8	10	Forest garden lizard / Chinese water dragon	Calotes emma/Physignatus cocincinus	Squamata	Lizard	Vietnam	Asia	WC	https://reptile-database.reptarium.cz/species?genus=Calotes&species=emma&search_param=%28%28common_name%3D%27Calotes+emma%27%29%29 , https://reptile-database.reptarium.cz/species?genus=Physignathus&species=cocincinus&search_param=%28%28common_name%3D%27Physignatus+cocincinus%27%29%29
8	10	Forest garden lizard / Chinese water dragon	Calotes emma/Physignatus cocincinus	Squamata	Lizard	Vietnam	Asia	WC	https://reptile-database.reptarium.cz/species?genus=Calotes&species=emma&search_param=%28%28common_name%3D%27Calotes+emma%27%29%29 , https://reptile-database.reptarium.cz/species?genus=Physignathus&species=cocincinus&search_param=%28%28common_name%3D%27Physignatus+cocincinus%27%29%29
8	10	Forest garden lizard / Chinese water dragon	Calotes emma/Physignatus cocincinus	Squamata	Lizard	Vietnam	Asia	WC	https://reptile-database.reptarium.cz/species?genus=Calotes&species=emma&search_param=%28%28common_name%3D%27Calotes+emma%27%29%29 , https://reptile-database.reptarium.cz/species?genus=Physignathus&species=cocincinus&search_param=%28%28common_name%3D%27Physignatus+cocincinus%27%29%29
8	10	Forest garden lizard / Chinese water dragon	Calotes emma/Physignatus cocincinus	Squamata	Lizard	Vietnam	Asia	WC	https://reptile-database.reptarium.cz/species?genus=Calotes&species=emma&search_param=%28%28common_name%3D%27Calotes+emma%27%29%29 , https://reptile-database.reptarium.cz/species?genus=Physignathus&species=cocincinus&search_param=%28%28common_name%3D%27Physignatus+cocincinus%27%29%29
8	10	Forest garden lizard / Chinese water dragon	Calotes emma/Physignatus cocincinus	Squamata	Lizard	Vietnam	Asia	WC	https://reptile-database.reptarium.cz/species?genus=Calotes&species=emma&search_param=%28%28common_name%3D%27Calotes+emma%27%29%29 , https://reptile-database.reptarium.cz/species?genus=Physignathus&species=cocincinus&search_param=%28%28common_name%3D%27Physignatus+cocincinus%27%29%29
8	10	Forest garden lizard / Chinese water dragon	Calotes emma/Physignatus cocincinus	Squamata	Lizard	Vietnam	Asia	WC	https://reptile-database.reptarium.cz/species?genus=Calotes&species=emma&search_param=%28%28common_name%3D%27Calotes+emma%27%29%29 , https://reptile-database.reptarium.cz/species?genus=Physignathus&species=cocincinus&search_param=%28%28common_name%3D%27Physignatus+cocincinus%27%29%29
8	10	Forest garden lizard / Chinese water dragon	Calotes emma/Physignatus cocincinus	Squamata	Lizard	Vietnam	Asia	WC	https://reptile-database.reptarium.cz/species?genus=Calotes&species=emma&search_param=%28%28common_name%3D%27Calotes+emma%27%29%29 , https://reptile-database.reptarium.cz/species?genus=Physignathus&species=cocincinus&search_param=%28%28common_name%3D%27Physignatus+cocincinus%27%29%29

Table S1: Animal species sampled in this study - country origin and categorization as captive bred (CB), farm bred (FB) and wild-caught (WC)

Shipment No.	Sample batch	Animal species	Scientific name of animal/s	Order	Suborder	Country origin	Continent	Captive bred (CB), farm bred (FB), wild-caught (WC)	Web URL used for assigning animal species to categories CB, FB and WC
8	10	Forest garden lizard / Chinese water dragon	Calotes emma/Physignatus cocincinus	Squamata	Lizard	Vietnam	Asia	WC	https://reptile-database.reptarium.cz/species?genus=Calotes&species=emma&search_param=%28%28common_name%3D%27Calotes+emma%27%29%29 , https://reptile-database.reptarium.cz/species?genus=Physignathus&species=cocincinus&search_param=%28%28common_name%3D%27Physignatus+cocincinus%27%29%29
8	10	Forest garden lizard / Chinese water dragon	Calotes emma/Physignatus cocincinus	Squamata	Lizard	Vietnam	Asia	WC	https://reptile-database.reptarium.cz/species?genus=Calotes&species=emma&search_param=%28%28common_name%3D%27Calotes+emma%27%29%29 , https://reptile-database.reptarium.cz/species?genus=Physignathus&species=cocincinus&search_param=%28%28common_name%3D%27Physignatus+cocincinus%27%29%29
8	10	Forest garden lizard / Chinese water dragon	Calotes emma/Physignatus cocincinus	Squamata	Lizard	Vietnam	Asia	WC	https://reptile-database.reptarium.cz/species?genus=Calotes&species=emma&search_param=%28%28common_name%3D%27Calotes+emma%27%29%29 , https://reptile-database.reptarium.cz/species?genus=Physignathus&species=cocincinus&search_param=%28%28common_name%3D%27Physignatus+cocincinus%27%29%29
8	10	Forest garden lizard / Chinese water dragon	Calotes emma/Physignatus cocincinus	Squamata	Lizard	Vietnam	Asia	WC	https://reptile-database.reptarium.cz/species?genus=Calotes&species=emma&search_param=%28%28common_name%3D%27Calotes+emma%27%29%29 , https://reptile-database.reptarium.cz/species?genus=Physignathus&species=cocincinus&search_param=%28%28common_name%3D%27Physignatus+cocincinus%27%29%29
8	10	Forest garden lizard / Chinese water dragon	Calotes emma/Physignatus cocincinus	Squamata	Lizard	Vietnam	Asia	WC	https://reptile-database.reptarium.cz/species?genus=Calotes&species=emma&search_param=%28%28common_name%3D%27Calotes+emma%27%29%29 , https://reptile-database.reptarium.cz/species?genus=Physignathus&species=cocincinus&search_param=%28%28common_name%3D%27Physignatus+cocincinus%27%29%29
8	10	Forest garden lizard / Chinese water dragon	Calotes emma/Physignatus cocincinus	Squamata	Lizard	Vietnam	Asia	WC	https://reptile-database.reptarium.cz/species?genus=Calotes&species=emma&search_param=%28%28common_name%3D%27Calotes+emma%27%29%29 , https://reptile-database.reptarium.cz/species?genus=Physignathus&species=cocincinus&search_param=%28%28common_name%3D%27Physignatus+cocincinus%27%29%29
8	10	Forest garden lizard / Chinese water dragon	Calotes emma/Physignatus cocincinus	Squamata	Lizard	Vietnam	Asia	WC	https://reptile-database.reptarium.cz/species?genus=Calotes&species=emma&search_param=%28%28common_name%3D%27Calotes+emma%27%29%29 , https://reptile-database.reptarium.cz/species?genus=Physignathus&species=cocincinus&search_param=%28%28common_name%3D%27Physignatus+cocincinus%27%29%29

Table S1: Animal species sampled in this study - country origin and categorization as captive bred (CB), farm bred (FB) and wild-caught (WC)

Shipment No.	Sample batch	Animal species	Scientific name of animal/s	Order	Suborder	Country origin	Continent	Captive bred (CB), farm bred (FB), wild-caught (WC)	Web URL used for assigning animal species to categories CB, FB and WC
8	10	Forest garden lizard / Chinese water dragon	Calotes emma/Physignatus cocincinus	Squamata	Lizard	Vietnam	Asia	WC	https://reptile-database.reptarium.cz/species?genus=Calotes&species=emma&search_param=%28%28common_name%3D%27Calotes+emma%27%29%29 , https://reptile-database.reptarium.cz/species?genus=Physignathus&species=cocincinus&search_param=%28%28common_name%3D%27Physignatus+cocincinus%27%29%29
8	10	Forest garden lizard / Chinese water dragon	Calotes emma/Physignatus cocincinus	Squamata	Lizard	Vietnam	Asia	WC	https://reptile-database.reptarium.cz/species?genus=Calotes&species=emma&search_param=%28%28common_name%3D%27Calotes+emma%27%29%29 , https://reptile-database.reptarium.cz/species?genus=Physignathus&species=cocincinus&search_param=%28%28common_name%3D%27Physignatus+cocincinus%27%29%29
8	10	Forest garden lizard / Chinese water dragon	Calotes emma/Physignatus cocincinus	Squamata	Lizard	Vietnam	Asia	WC	https://reptile-database.reptarium.cz/species?genus=Calotes&species=emma&search_param=%28%28common_name%3D%27Calotes+emma%27%29%29 , https://reptile-database.reptarium.cz/species?genus=Physignathus&species=cocincinus&search_param=%28%28common_name%3D%27Physignatus+cocincinus%27%29%29
8	10	Forest garden lizard / Chinese water dragon	Calotes emma/Physignatus cocincinus	Squamata	Lizard	Vietnam	Asia	WC	https://reptile-database.reptarium.cz/species?genus=Calotes&species=emma&search_param=%28%28common_name%3D%27Calotes+emma%27%29%29 , https://reptile-database.reptarium.cz/species?genus=Physignathus&species=cocincinus&search_param=%28%28common_name%3D%27Physignatus+cocincinus%27%29%29
8	10	Forest garden lizard / Chinese water dragon	Calotes emma/Physignatus cocincinus	Squamata	Lizard	Vietnam	Asia	WC	https://reptile-database.reptarium.cz/species?genus=Calotes&species=emma&search_param=%28%28common_name%3D%27Calotes+emma%27%29%29 , https://reptile-database.reptarium.cz/species?genus=Physignathus&species=cocincinus&search_param=%28%28common_name%3D%27Physignatus+cocincinus%27%29%29
8	10	Forest garden lizard / Chinese water dragon	Calotes emma/Physignatus cocincinus	Squamata	Lizard	Vietnam	Asia	WC	https://reptile-database.reptarium.cz/species?genus=Calotes&species=emma&search_param=%28%28common_name%3D%27Calotes+emma%27%29%29 , https://reptile-database.reptarium.cz/species?genus=Physignathus&species=cocincinus&search_param=%28%28common_name%3D%27Physignatus+cocincinus%27%29%29
8	10	Forest garden lizard / Chinese water dragon	Calotes emma/Physignatus cocincinus	Squamata	Lizard	Vietnam	Asia	WC	https://reptile-database.reptarium.cz/species?genus=Calotes&species=emma&search_param=%28%28common_name%3D%27Calotes+emma%27%29%29 , https://reptile-database.reptarium.cz/species?genus=Physignathus&species=cocincinus&search_param=%28%28common_name%3D%27Physignatus+cocincinus%27%29%29

Table S1: Animal species sampled in this study - country origin and categorization as captive bred (CB), farm bred (FB) and wild-caught (WC)

Shipment No.	Sample batch	Animal species	Scientific name of animal/s	Order	Suborder	Country origin	Continent	Captive bred (CB), farm bred (FB), wild-caught (WC)	Web URL used for assigning animal species to categories CB, FB and WC
8	10	Forest garden lizard / Chinese water dragon	Calotes emma/Physignatus cocincinus	Squamata	Lizard	Vietnam	Asia	WC	https://reptile-database.reptarium.cz/species?genus=Calotes&species=emma&search_param=%28%28common_name%3D%27Calotes+emma%27%29%29 , https://reptile-database.reptarium.cz/species?genus=Physignathus&species=cocincinus&search_param=%28%28common_name%3D%27Physignatus+cocincinus%27%29%29
8	10	Forest garden lizard / Chinese water dragon	Calotes emma/Physignatus cocincinus	Squamata	Lizard	Vietnam	Asia	WC	https://reptile-database.reptarium.cz/species?genus=Calotes&species=emma&search_param=%28%28common_name%3D%27Calotes+emma%27%29%29 , https://reptile-database.reptarium.cz/species?genus=Physignathus&species=cocincinus&search_param=%28%28common_name%3D%27Physignatus+cocincinus%27%29%29
8	10	Forest garden lizard / Chinese water dragon	Calotes emma/Physignatus cocincinus	Squamata	Lizard	Vietnam	Asia	WC	https://reptile-database.reptarium.cz/species?genus=Calotes&species=emma&search_param=%28%28common_name%3D%27Calotes+emma%27%29%29 , https://reptile-database.reptarium.cz/species?genus=Physignathus&species=cocincinus&search_param=%28%28common_name%3D%27Physignatus+cocincinus%27%29%29
8	10	Forest garden lizard / Chinese water dragon	Calotes emma/Physignatus cocincinus	Squamata	Lizard	Vietnam	Asia	WC	https://reptile-database.reptarium.cz/species?genus=Calotes&species=emma&search_param=%28%28common_name%3D%27Calotes+emma%27%29%29 , https://reptile-database.reptarium.cz/species?genus=Physignathus&species=cocincinus&search_param=%28%28common_name%3D%27Physignatus+cocincinus%27%29%29
8	10	Forest garden lizard / Chinese water dragon	Calotes emma/Physignatus cocincinus	Squamata	Lizard	Vietnam	Asia	WC	https://reptile-database.reptarium.cz/species?genus=Calotes&species=emma&search_param=%28%28common_name%3D%27Calotes+emma%27%29%29 , https://reptile-database.reptarium.cz/species?genus=Physignathus&species=cocincinus&search_param=%28%28common_name%3D%27Physignatus+cocincinus%27%29%29
8	10	Forest garden lizard / Chinese water dragon	Calotes emma/Physignatus cocincinus	Squamata	Lizard	Vietnam	Asia	WC	https://reptile-database.reptarium.cz/species?genus=Calotes&species=emma&search_param=%28%28common_name%3D%27Calotes+emma%27%29%29 , https://reptile-database.reptarium.cz/species?genus=Physignathus&species=cocincinus&search_param=%28%28common_name%3D%27Physignatus+cocincinus%27%29%29
8	10	Forest garden lizard / Chinese water dragon	Calotes emma/Physignatus cocincinus	Squamata	Lizard	Vietnam	Asia	WC	https://reptile-database.reptarium.cz/species?genus=Calotes&species=emma&search_param=%28%28common_name%3D%27Calotes+emma%27%29%29 , https://reptile-database.reptarium.cz/species?genus=Physignathus&species=cocincinus&search_param=%28%28common_name%3D%27Physignatus+cocincinus%27%29%29

Table S1: Animal species sampled in this study - country origin and categorization as captive bred (CB), farm bred (FB) and wild-caught (WC)

Shipment No.	Sample batch	Animal species	Scientific name of animal/s	Order	Suborder	Country origin	Continent	Captive bred (CB), farm bred (FB), wild-caught (WC)	Web URL used for assigning animal species to categories CB, FB and WC
8	10	Forest garden lizard / Chinese water dragon	Calotes emma/Physignatus cocincinus	Squamata	Lizard	Vietnam	Asia	WC	https://reptile-database.reptarium.cz/species?genus=Calotes&species=emma&search_param=%28%28common_name%3D%27Calotes+emma%27%29%29 , https://reptile-database.reptarium.cz/species?genus=Physignathus&species=cocincinus&search_param=%28%28common_name%3D%27Physignatus+cocincinus%27%29%29
8	10	Forest garden lizard / Chinese water dragon	Calotes emma/Physignatus cocincinus	Squamata	Lizard	Vietnam	Asia	WC	https://reptile-database.reptarium.cz/species?genus=Calotes&species=emma&search_param=%28%28common_name%3D%27Calotes+emma%27%29%29 , https://reptile-database.reptarium.cz/species?genus=Physignathus&species=cocincinus&search_param=%28%28common_name%3D%27Physignatus+cocincinus%27%29%29
8	10	Chinese water dragon	Physignatus cocincinus	Squamata	Lizard	Vietnam	Asia	WC	https://reptile-database.reptarium.cz/species?genus=Physignathus&species=cocincinus&search_param=%28%28common_name%3D%27gr%C3%BCne+wasseragame%27%29%29
8	10	Chinese water dragon	Physignatus cocincinus	Squamata	Lizard	Vietnam	Asia	WC	https://reptile-database.reptarium.cz/species?genus=Physignathus&species=cocincinus&search_param=%28%28common_name%3D%27gr%C3%BCne+wasseragame%27%29%29
8	10	Chinese water dragon	Physignatus cocincinus	Squamata	Lizard	Vietnam	Asia	WC	https://reptile-database.reptarium.cz/species?genus=Physignathus&species=cocincinus&search_param=%28%28common_name%3D%27gr%C3%BCne+wasseragame%27%29%29
8	10	Chinese water dragon	Physignatus cocincinus	Squamata	Lizard	Vietnam	Asia	WC	https://reptile-database.reptarium.cz/species?genus=Physignathus&species=cocincinus&search_param=%28%28common_name%3D%27gr%C3%BCne+wasseragame%27%29%29
8	10	Chinese water dragon	Physignatus cocincinus	Squamata	Lizard	Vietnam	Asia	WC	https://reptile-database.reptarium.cz/species?genus=Physignathus&species=cocincinus&search_param=%28%28common_name%3D%27gr%C3%BCne+wasseragame%27%29%29
9	11	Crested gecko	Correlophus ciliatus	Squamata	Lizard	Japan	Asia	CB	https://reptile-database.reptarium.cz/species?genus=Correlophus&species=ciliatus&search_param=%28%28common_name%3D%27Correlophus+ciliatus%27%29%29
9	11	Crested gecko	Correlophus ciliatus	Squamata	Lizard	Japan	Asia	CB	https://reptile-database.reptarium.cz/species?genus=Correlophus&species=ciliatus&search_param=%28%28common_name%3D%27Correlophus+ciliatus%27%29%29
9	11	Crested gecko	Correlophus ciliatus	Squamata	Lizard	Japan	Asia	CB	https://reptile-database.reptarium.cz/species?genus=Correlophus&species=ciliatus&search_param=%28%28common_name%3D%27Correlophus+ciliatus%27%29%29

Table S1: Animal species sampled in this study - country origin and categorization as captive bred (CB), farm bred (FB) and wild-caught (WC)

Shipment No.	Sample batch	Animal species	Scientific name of animal/s	Order	Suborder	Country origin	Continent	Captive bred (CB), farm bred (FB), wild-caught (WC)	Web URL used for assigning animal species to categories CB, FB and WC
9	11	Crested gecko	Correlophus ciliatus	Squamata	Lizard	Japan	Asia	CB	https://reptile-database.reptarium.cz/species?genus=Correlophus&species=ciliatus&search_param=%28%28common_name%3D%27Correlophus+ciliatus%27%29%29
9	11	Crested gecko	Correlophus ciliatus	Squamata	Lizard	Japan	Asia	CB	https://reptile-database.reptarium.cz/species?genus=Correlophus&species=ciliatus&search_param=%28%28common_name%3D%27Correlophus+ciliatus%27%29%29
9	12	Central bearded dragon	Pogona vitticeps	Squamata	Lizard	Japan	Asia	CB	https://reptile-database.reptarium.cz/species?genus=Pogona&species=vitticeps&search_param=%28%28common_name%3D%27Pogona+vitticeps%27%29%29
9	12	Central bearded dragon	Pogona vitticeps	Squamata	Lizard	Japan	Asia	CB	https://reptile-database.reptarium.cz/species?genus=Pogona&species=vitticeps&search_param=%28%28common_name%3D%27Pogona+vitticeps%27%29%29
9	12	Central bearded dragon	Pogona vitticeps	Squamata	Lizard	Japan	Asia	CB	https://reptile-database.reptarium.cz/species?genus=Pogona&species=vitticeps&search_param=%28%28common_name%3D%27Pogona+vitticeps%27%29%29
9	12	Central bearded dragon	Pogona vitticeps	Squamata	Lizard	Japan	Asia	CB	https://reptile-database.reptarium.cz/species?genus=Pogona&species=vitticeps&search_param=%28%28common_name%3D%27Pogona+vitticeps%27%29%29
9	13	Giant horned lizard	Phrynosoma asio	Squamata	Lizard	Japan	Asia	CB	https://reptile-database.reptarium.cz/species?genus=Phrynosoma&species=asio&search_param=%28%28common_name%3D%27Phrynosoma+asio%27%29%29
9	13	Giant horned lizard	Phrynosoma asio	Squamata	Lizard	Japan	Asia	CB	https://reptile-database.reptarium.cz/species?genus=Phrynosoma&species=asio&search_param=%28%28common_name%3D%27Phrynosoma+asio%27%29%29
10	14	Peninsula cooter	Pseudemys peninsularis	Testudines	Turtle	USA	America	FB	https://reptile-database.reptarium.cz/species?genus=Pseudemys&species=peninsularis&search_param=%28%28common_name%3D%27Pseudemys+peninsularis%27%29%29
10	14	Peninsula cooter	Pseudemys peninsularis	Testudines	Turtle	USA	America	FB	https://reptile-database.reptarium.cz/species?genus=Pseudemys&species=peninsularis&search_param=%28%28common_name%3D%27Pseudemys+peninsularis%27%29%29
10	14	Peninsula cooter	Pseudemys peninsularis	Testudines	Turtle	USA	America	FB	https://reptile-database.reptarium.cz/species?genus=Pseudemys&species=peninsularis&search_param=%28%28common_name%3D%27Pseudemys+peninsularis%27%29%29
10	14	Peninsula cooter	Pseudemys peninsularis	Testudines	Turtle	USA	America	FB	https://reptile-database.reptarium.cz/species?genus=Pseudemys&species=peninsularis&search_param=%28%28common_name%3D%27Pseudemys+peninsularis%27%29%29

Table S1: Animal species sampled in this study - country origin and categorization as captive bred (CB), farm bred (FB) and wild-caught (WC)

Shipment No.	Sample batch	Animal species	Scientific name of animal/s	Order	Suborder	Country origin	Continent	Captive bred (CB), farm bred (FB), wild-caught (WC)	Web URL used for assigning animal species to categories CB, FB and WC
10	14	Peninsula cooter	Pseudemys peninsularis	Testudines	Turtle	USA	America	FB	https://reptile-database.reptarium.cz/species?genus=Pseudemys&species=peninsularis&search_param=%28%28common_name%3D%27Pseudemys+peninsularis%27%29%29
10	14	Peninsula cooter	Pseudemys peninsularis	Testudines	Turtle	USA	America	FB	https://reptile-database.reptarium.cz/species?genus=Pseudemys&species=peninsularis&search_param=%28%28common_name%3D%27Pseudemys+peninsularis%27%29%29
10	14	Peninsula cooter	Pseudemys peninsularis	Testudines	Turtle	USA	America	FB	https://reptile-database.reptarium.cz/species?genus=Pseudemys&species=peninsularis&search_param=%28%28common_name%3D%27Pseudemys+peninsularis%27%29%29
10	14	Peninsula cooter	Pseudemys peninsularis	Testudines	Turtle	USA	America	FB	https://reptile-database.reptarium.cz/species?genus=Pseudemys&species=peninsularis&search_param=%28%28common_name%3D%27Pseudemys+peninsularis%27%29%29
10	14	Peninsula cooter	Pseudemys peninsularis	Testudines	Turtle	USA	America	FB	https://reptile-database.reptarium.cz/species?genus=Pseudemys&species=peninsularis&search_param=%28%28common_name%3D%27Pseudemys+peninsularis%27%29%29
10	14	Peninsula cooter	Pseudemys peninsularis	Testudines	Turtle	USA	America	FB	https://reptile-database.reptarium.cz/species?genus=Pseudemys&species=peninsularis&search_param=%28%28common_name%3D%27Pseudemys+peninsularis%27%29%29
10	14	Peninsula cooter	Pseudemys peninsularis	Testudines	Turtle	USA	America	FB	https://reptile-database.reptarium.cz/species?genus=Pseudemys&species=peninsularis&search_param=%28%28common_name%3D%27Pseudemys+peninsularis%27%29%29
11	15	Collared lizard	Crotaphytus collaris	Squamata	Lizard	USA	America	WC	https://reptile-database.reptarium.cz/species?genus=Crotaphytus&species=collaris&search_param=%28%28common_name%3D%27Crotaphytus+collaris%27%29%29
11	15	Collared lizard	Crotaphytus collaris	Squamata	Lizard	USA	America	WC	https://reptile-database.reptarium.cz/species?genus=Crotaphytus&species=collaris&search_param=%28%28common_name%3D%27Crotaphytus+collaris%27%29%29
11	15	Collared lizard	Crotaphytus collaris	Squamata	Lizard	USA	America	WC	https://reptile-database.reptarium.cz/species?genus=Crotaphytus&species=collaris&search_param=%28%28common_name%3D%27Crotaphytus+collaris%27%29%29
11	15	Collared lizard	Crotaphytus collaris	Squamata	Lizard	USA	America	WC	https://reptile-database.reptarium.cz/species?genus=Crotaphytus&species=collaris&search_param=%28%28common_name%3D%27Crotaphytus+collaris%27%29%29
12	16	Carpet python	Morelia spilota (variegata)	Squamata	Snake	Canada	America	CB	https://reptile-database.reptarium.cz/species?genus=Morelia&species=spilota&search_param=%28%28common_name%3D%27Morelia+spilota%27%29%29

Table S1: Animal species sampled in this study - country origin and categorization as captive bred (CB), farm bred (FB) and wild-caught (WC)

Shipment No.	Sample batch	Animal species	Scientific name of animal/s	Order	Suborder	Country origin	Continent	Captive bred (CB), farm bred (FB), wild-caught (WC)	Web URL used for assigning animal species to categories CB, FB and WC
12	16	Carpet python	Morelia spilota (variegata)	Squamata	Snake	Canada	America	CB	https://reptile-database.reptarium.cz/species?genus=Morelia&species=spilota&search_param=%28%28common_name%3D%27Morelia+spilota%27%29%29
12	16	Carpet python	Morelia spilota (variegata)	Squamata	Snake	Canada	America	CB	https://reptile-database.reptarium.cz/species?genus=Morelia&species=spilota&search_param=%28%28common_name%3D%27Morelia+spilota%27%29%29
12	16	Carpet python	Morelia spilota (variegata)	Squamata	Snake	Canada	America	CB	https://reptile-database.reptarium.cz/species?genus=Morelia&species=spilota&search_param=%28%28common_name%3D%27Morelia+spilota%27%29%29
12	16	Carpet python	Morelia spilota (variegata)	Squamata	Snake	Canada	America	CB	https://reptile-database.reptarium.cz/species?genus=Morelia&species=spilota&search_param=%28%28common_name%3D%27Morelia+spilota%27%29%29
12	16	Carpet python	Morelia spilota (variegata)	Squamata	Snake	Canada	America	CB	https://reptile-database.reptarium.cz/species?genus=Morelia&species=spilota&search_param=%28%28common_name%3D%27Morelia+spilota%27%29%29
12	16	Carpet python	Morelia spilota (variegata)	Squamata	Snake	Canada	America	CB	https://reptile-database.reptarium.cz/species?genus=Morelia&species=spilota&search_param=%28%28common_name%3D%27Morelia+spilota%27%29%29
12	16	Carpet python	Morelia spilota (variegata)	Squamata	Snake	Canada	America	CB	https://reptile-database.reptarium.cz/species?genus=Morelia&species=spilota&search_param=%28%28common_name%3D%27Morelia+spilota%27%29%29
12	16	Carpet python	Morelia spilota (variegata)	Squamata	Snake	Canada	America	CB	https://reptile-database.reptarium.cz/species?genus=Morelia&species=spilota&search_param=%28%28common_name%3D%27Morelia+spilota%27%29%29
12	16	Carpet python	Morelia spilota (variegata)	Squamata	Snake	Canada	America	CB	https://reptile-database.reptarium.cz/species?genus=Morelia&species=spilota&search_param=%28%28common_name%3D%27Morelia+spilota%27%29%29
12	16	Carpet python	Morelia spilota (variegata)	Squamata	Snake	Canada	America	CB	https://reptile-database.reptarium.cz/species?genus=Morelia&species=spilota&search_param=%28%28common_name%3D%27Morelia+spilota%27%29%29
13	17	Yellow-bellied slider	Trachemys scripta scripta	Testudines	Turtle	USA	America	FB	https://reptile-database.reptarium.cz/species?genus=Trachemys&species=scripta&search_param=%28%28common_name%3D%27Trachemys+scripta+scripta%27%29%29
13	17	Yellow-bellied slider	Trachemys scripta scripta	Testudines	Turtle	USA	America	FB	https://reptile-database.reptarium.cz/species?genus=Trachemys&species=scripta&search_param=%28%28common_name%3D%27Trachemys+scripta+scripta%27%29%29

Table S1: Animal species sampled in this study - country origin and categorization as captive bred (CB), farm bred (FB) and wild-caught (WC)

Shipment No.	Sample batch	Animal species	Scientific name of animal/s	Order	Suborder	Country origin	Continent	Captive bred (CB), farm bred (FB), wild-caught (WC)	Web URL used for assigning animal species to categories CB, FB and WC
13	17	Yellow-bellied slider	Trachemys scripta scripta	Testudines	Turtle	USA	America	FB	https://reptile-database.reptarium.cz/species?genus=Trachemys&species=scripta&search_param=%28%28common_name%3D%27Trachemys+scripta+scripta%27%29%29
13	17	Yellow-bellied slider	Trachemys scripta scripta	Testudines	Turtle	USA	America	FB	https://reptile-database.reptarium.cz/species?genus=Trachemys&species=scripta&search_param=%28%28common_name%3D%27Trachemys+scripta+scripta%27%29%29
13	17	Yellow-bellied slider	Trachemys scripta scripta	Testudines	Turtle	USA	America	FB	https://reptile-database.reptarium.cz/species?genus=Trachemys&species=scripta&search_param=%28%28common_name%3D%27Trachemys+scripta+scripta%27%29%29
13	17	Yellow-bellied slider	Trachemys scripta scripta	Testudines	Turtle	USA	America	FB	https://reptile-database.reptarium.cz/species?genus=Trachemys&species=scripta&search_param=%28%28common_name%3D%27Trachemys+scripta+scripta%27%29%29
13	17	Yellow-bellied slider	Trachemys scripta scripta	Testudines	Turtle	USA	America	FB	https://reptile-database.reptarium.cz/species?genus=Trachemys&species=scripta&search_param=%28%28common_name%3D%27Trachemys+scripta+scripta%27%29%29
13	17	Yellow-bellied slider	Trachemys scripta scripta	Testudines	Turtle	USA	America	FB	https://reptile-database.reptarium.cz/species?genus=Trachemys&species=scripta&search_param=%28%28common_name%3D%27Trachemys+scripta+scripta%27%29%29
13	17	Yellow-bellied slider	Trachemys scripta scripta	Testudines	Turtle	USA	America	FB	https://reptile-database.reptarium.cz/species?genus=Trachemys&species=scripta&search_param=%28%28common_name%3D%27Trachemys+scripta+scripta%27%29%29
13	17	Yellow-bellied slider	Trachemys scripta scripta	Testudines	Turtle	USA	America	FB	https://reptile-database.reptarium.cz/species?genus=Trachemys&species=scripta&search_param=%28%28common_name%3D%27Trachemys+scripta+scripta%27%29%29
14	18	Green tree python	Morelia viridis	Squamata	Snake	USA	America	CB	https://reptile-database.reptarium.cz/species?genus=Morelia&species=viridis&search_param=%28%28common_name%3D%27Morelia+viridis%27%29%29
14	18	Green tree python	Morelia viridis	Squamata	Snake	USA	America	CB	https://reptile-database.reptarium.cz/species?genus=Morelia&species=viridis&search_param=%28%28common_name%3D%27Morelia+viridis%27%29%29
15	19	Veiled chameleon	Chamaeleo calyptratus	Squamata	Lizard	Ukraine	Europe	CB	https://reptile-database.reptarium.cz/species?genus=Chamaeleo&species=calyptratus&search_param=%28%28common_name%3D%27Chamaeleo+calyptratus%27%29%29
15	19	Veiled chameleon	Chamaeleo calyptratus	Squamata	Lizard	Ukraine	Europe	CB	https://reptile-database.reptarium.cz/species?genus=Chamaeleo&species=calyptratus&search_param=%28%28common_name%3D%27Chamaeleo+calyptratus%27%29%29

Table S1: Animal species sampled in this study - country origin and categorization as captive bred (CB), farm bred (FB) and wild-caught (WC)

Shipment No.	Sample batch	Animal species	Scientific name of animal/s	Order	Suborder	Country origin	Continent	Captive bred (CB), farm bred (FB), wild-caught (WC)	Web URL used for assigning animal species to categories CB, FB and WC
15	19	Veiled chameleon	Chamaeleo calyptratus	Squamata	Lizard	Ukraine	Europe	CB	https://reptile-database.reptarium.cz/species?genus=Chamaeleo&species=calyptratus&search_param=%28%28common_name%3D%27Chamaeleo+calyptratus%27%29%29
15	19	Veiled chameleon	Chamaeleo calyptratus	Squamata	Lizard	Ukraine	Europe	CB	https://reptile-database.reptarium.cz/species?genus=Chamaeleo&species=calyptratus&search_param=%28%28common_name%3D%27Chamaeleo+calyptratus%27%29%29
15	19	Veiled chameleon	Chamaeleo calyptratus	Squamata	Lizard	Ukraine	Europe	CB	https://reptile-database.reptarium.cz/species?genus=Chamaeleo&species=calyptratus&search_param=%28%28common_name%3D%27Chamaeleo+calyptratus%27%29%29
15	19	Veiled chameleon	Chamaeleo calyptratus	Squamata	Lizard	Ukraine	Europe	CB	https://reptile-database.reptarium.cz/species?genus=Chamaeleo&species=calyptratus&search_param=%28%28common_name%3D%27Chamaeleo+calyptratus%27%29%29
15	19	Veiled chameleon	Chamaeleo calyptratus	Squamata	Lizard	Ukraine	Europe	CB	https://reptile-database.reptarium.cz/species?genus=Chamaeleo&species=calyptratus&search_param=%28%28common_name%3D%27Chamaeleo+calyptratus%27%29%29
16	20	Central bearded dragon	Pogona vitticeps	Squamata	Lizard	Japan	Asia	CB	https://reptile-database.reptarium.cz/species?genus=Pogona&species=vitticeps&search_param=%28%28common_name%3D%27Pogona+vitticeps%27%29%29
16	20	Central bearded dragon	Pogona vitticeps	Squamata	Lizard	Japan	Asia	CB	https://reptile-database.reptarium.cz/species?genus=Pogona&species=vitticeps&search_param=%28%28common_name%3D%27Pogona+vitticeps%27%29%29
16	20	Central bearded dragon	Pogona vitticeps	Squamata	Lizard	Japan	Asia	CB	https://reptile-database.reptarium.cz/species?genus=Pogona&species=vitticeps&search_param=%28%28common_name%3D%27Pogona+vitticeps%27%29%29
16	20	Central bearded dragon	Pogona vitticeps	Squamata	Lizard	Japan	Asia	CB	https://reptile-database.reptarium.cz/species?genus=Pogona&species=vitticeps&search_param=%28%28common_name%3D%27Pogona+vitticeps%27%29%29
16	20	Central bearded dragon	Pogona vitticeps	Squamata	Lizard	Japan	Asia	CB	https://reptile-database.reptarium.cz/species?genus=Pogona&species=vitticeps&search_param=%28%28common_name%3D%27Pogona+vitticeps%27%29%29
17	21	Jackson's chameleon	Trioceros jacksonii	Squamata	Lizard	USA	America	CB	https://reptile-database.reptarium.cz/species?genus=Trioceros&species=jacksonii&search_param=%28%28common_name%3D%27Trioceros+jacksonii%27%29%29
17	21	Jackson's chameleon	Trioceros jacksonii	Squamata	Lizard	USA	America	CB	https://reptile-database.reptarium.cz/species?genus=Trioceros&species=jacksonii&search_param=%28%28common_name%3D%27Trioceros+jacksonii%27%29%29

Table S1: Animal species sampled in this study - country origin and categorization as captive bred (CB), farm bred (FB) and wild-caught (WC)

Shipment No.	Sample batch	Animal species	Scientific name of animal/s	Order	Suborder	Country origin	Continent	Captive bred (CB), farm bred (FB), wild-caught (WC)	Web URL used for assigning animal species to categories CB, FB and WC
18	22	Collared lizard	Crotaphytus collaris	Squamata	Lizard	USA	America	WC	https://reptile-database.reptarium.cz/species?genus=Crotaphytus&species=collaris&search_param=%28%28common_name%3D%27Crotaphytus+collaris%27%29%29
18	22	Collared lizard	Crotaphytus collaris	Squamata	Lizard	USA	America	WC	https://reptile-database.reptarium.cz/species?genus=Crotaphytus&species=collaris&search_param=%28%28common_name%3D%27Crotaphytus+collaris%27%29%29
18	22	Collared lizard	Crotaphytus collaris	Squamata	Lizard	USA	America	WC	https://reptile-database.reptarium.cz/species?genus=Crotaphytus&species=collaris&search_param=%28%28common_name%3D%27Crotaphytus+collaris%27%29%29
18	22	Collared lizard	Crotaphytus collaris	Squamata	Lizard	USA	America	WC	https://reptile-database.reptarium.cz/species?genus=Crotaphytus&species=collaris&search_param=%28%28common_name%3D%27Crotaphytus+collaris%27%29%29
18	22	Collared lizard	Crotaphytus collaris	Squamata	Lizard	USA	America	WC	https://reptile-database.reptarium.cz/species?genus=Crotaphytus&species=collaris&search_param=%28%28common_name%3D%27Crotaphytus+collaris%27%29%29
18	22	Collared lizard	Crotaphytus collaris	Squamata	Lizard	USA	America	WC	https://reptile-database.reptarium.cz/species?genus=Crotaphytus&species=collaris&search_param=%28%28common_name%3D%27Crotaphytus+collaris%27%29%29
18	22	Collared lizard	Crotaphytus collaris	Squamata	Lizard	USA	America	WC	https://reptile-database.reptarium.cz/species?genus=Crotaphytus&species=collaris&search_param=%28%28common_name%3D%27Crotaphytus+collaris%27%29%29
18	22	Collared lizard	Crotaphytus collaris	Squamata	Lizard	USA	America	WC	https://reptile-database.reptarium.cz/species?genus=Crotaphytus&species=collaris&search_param=%28%28common_name%3D%27Crotaphytus+collaris%27%29%29
18	22	Collared lizard	Crotaphytus collaris	Squamata	Lizard	USA	America	WC	https://reptile-database.reptarium.cz/species?genus=Crotaphytus&species=collaris&search_param=%28%28common_name%3D%27Crotaphytus+collaris%27%29%29
18	22	Collared lizard	Crotaphytus collaris	Squamata	Lizard	USA	America	WC	https://reptile-database.reptarium.cz/species?genus=Crotaphytus&species=collaris&search_param=%28%28common_name%3D%27Crotaphytus+collaris%27%29%29
18	22	Collared lizard	Crotaphytus collaris	Squamata	Lizard	USA	America	WC	https://reptile-database.reptarium.cz/species?genus=Crotaphytus&species=collaris&search_param=%28%28common_name%3D%27Crotaphytus+collaris%27%29%29
18	23	Green basilisk	Basiliscus plumifrons	Squamata	Lizard	USA	America	FB	https://reptile-database.reptarium.cz/species?genus=Basiliscus&species=plumifrons&search_param=%28%28common_name%3D%27Basiliscus+plumifrons%27%29%29

Table S1: Animal species sampled in this study - country origin and categorization as captive bred (CB), farm bred (FB) and wild-caught (WC)

Shipment No.	Sample batch	Animal species	Scientific name of animal/s	Order	Suborder	Country origin	Continent	Captive bred (CB), farm bred (FB), wild-caught (WC)	Web URL used for assigning animal species to categories CB, FB and WC
18	23	Green basilisk	Basiliscus plumifrons	Squamata	Lizard	USA	America	FB	https://reptile-database.reptarium.cz/species?genus=Basiliscus&species=plumifrons&search_param=%28%28common_name%3D%27Basiliscus+plumifrons%27%29%29
18	23	Green basilisk	Basiliscus plumifrons	Squamata	Lizard	USA	America	FB	https://reptile-database.reptarium.cz/species?genus=Basiliscus&species=plumifrons&search_param=%28%28common_name%3D%27Basiliscus+plumifrons%27%29%29
18	23	Green basilisk	Basiliscus plumifrons	Squamata	Lizard	USA	America	FB	https://reptile-database.reptarium.cz/species?genus=Basiliscus&species=plumifrons&search_param=%28%28common_name%3D%27Basiliscus+plumifrons%27%29%29
18	23	Green basilisk	Basiliscus plumifrons	Squamata	Lizard	USA	America	FB	https://reptile-database.reptarium.cz/species?genus=Basiliscus&species=plumifrons&search_param=%28%28common_name%3D%27Basiliscus+plumifrons%27%29%29
18	23	Green basilisk	Basiliscus plumifrons	Squamata	Lizard	USA	America	FB	https://reptile-database.reptarium.cz/species?genus=Basiliscus&species=plumifrons&search_param=%28%28common_name%3D%27Basiliscus+plumifrons%27%29%29
18	23	Green basilisk	Basiliscus plumifrons	Squamata	Lizard	USA	America	FB	https://reptile-database.reptarium.cz/species?genus=Basiliscus&species=plumifrons&search_param=%28%28common_name%3D%27Basiliscus+plumifrons%27%29%29
18	24	Cuban giant anole	Anolis equestris	Squamata	Lizard	USA	America	WC/FB	https://reptile-database.reptarium.cz/species?genus=Anolis&species=equestris&search_param=%28%28common_name%3D%27Anolis+equestris%27%29%29
18	24	Cuban giant anole	Anolis equestris	Squamata	Lizard	USA	America	WC/FB	https://reptile-database.reptarium.cz/species?genus=Anolis&species=equestris&search_param=%28%28common_name%3D%27Anolis+equestris%27%29%29
18	24	Cuban giant anole	Anolis equestris	Squamata	Lizard	USA	America	WC/FB	https://reptile-database.reptarium.cz/species?genus=Anolis&species=equestris&search_param=%28%28common_name%3D%27Anolis+equestris%27%29%29
18	24	Cuban giant anole	Anolis equestris	Squamata	Lizard	USA	America	WC/FB	https://reptile-database.reptarium.cz/species?genus=Anolis&species=equestris&search_param=%28%28common_name%3D%27Anolis+equestris%27%29%29
18	24	Cuban giant anole	Anolis equestris	Squamata	Lizard	USA	America	WC/FB	https://reptile-database.reptarium.cz/species?genus=Anolis&species=equestris&search_param=%28%28common_name%3D%27Anolis+equestris%27%29%29
18	25	Green spiny lizard	Sceloporus malachiticus	Squamata	Lizard	USA	America	presumably CB	https://reptile-database.reptarium.cz/species?genus=Sceloporus&species=malachiticus&search_param=%28%28common_name%3D%27Sceloporus+malachiticus%27%29%29

Table S1: Animal species sampled in this study - country origin and categorization as captive bred (CB), farm bred (FB) and wild-caught (WC)

Shipment No.	Sample batch	Animal species	Scientific name of animal/s	Order	Suborder	Country origin	Continent	Captive bred (CB), farm bred (FB), wild-caught (WC)	Web URL used for assigning animal species to categories CB, FB and WC
18	25	Green spiny lizard	Sceloporus malachiticus	Squamata	Lizard	USA	America	presumably CB	https://reptile-database.reptarium.cz/species?genus=Sceloporus&species=malachiticus&search_param=%28%28common_name%3D%27Sceloporus+malachiticus%27%29%29
18	25	Green spiny lizard	Sceloporus malachiticus	Squamata	Lizard	USA	America	presumably CB	https://reptile-database.reptarium.cz/species?genus=Sceloporus&species=malachiticus&search_param=%28%28common_name%3D%27Sceloporus+malachiticus%27%29%29
18	25	Green spiny lizard	Sceloporus malachiticus	Squamata	Lizard	USA	America	presumably CB	https://reptile-database.reptarium.cz/species?genus=Sceloporus&species=malachiticus&search_param=%28%28common_name%3D%27Sceloporus+malachiticus%27%29%29
18	25	Green spiny lizard	Sceloporus malachiticus	Squamata	Lizard	USA	America	presumably CB	https://reptile-database.reptarium.cz/species?genus=Sceloporus&species=malachiticus&search_param=%28%28common_name%3D%27Sceloporus+malachiticus%27%29%29
18	25	Green spiny lizard	Sceloporus malachiticus	Squamata	Lizard	USA	America	presumably CB	https://reptile-database.reptarium.cz/species?genus=Sceloporus&species=malachiticus&search_param=%28%28common_name%3D%27Sceloporus+malachiticus%27%29%29
18	25	Green spiny lizard	Sceloporus malachiticus	Squamata	Lizard	USA	America	presumably CB	https://reptile-database.reptarium.cz/species?genus=Sceloporus&species=malachiticus&search_param=%28%28common_name%3D%27Sceloporus+malachiticus%27%29%29
18	25	Green spiny lizard	Sceloporus malachiticus	Squamata	Lizard	USA	America	presumably CB	https://reptile-database.reptarium.cz/species?genus=Sceloporus&species=malachiticus&search_param=%28%28common_name%3D%27Sceloporus+malachiticus%27%29%29
18	25	Green spiny lizard	Sceloporus malachiticus	Squamata	Lizard	USA	America	presumably CB	https://reptile-database.reptarium.cz/species?genus=Sceloporus&species=malachiticus&search_param=%28%28common_name%3D%27Sceloporus+malachiticus%27%29%29
18	25	Green spiny lizard	Sceloporus malachiticus	Squamata	Lizard	USA	America	presumably CB	https://reptile-database.reptarium.cz/species?genus=Sceloporus&species=malachiticus&search_param=%28%28common_name%3D%27Sceloporus+malachiticus%27%29%29
18	25	Green spiny lizard	Sceloporus malachiticus	Squamata	Lizard	USA	America	presumably CB	https://reptile-database.reptarium.cz/species?genus=Sceloporus&species=malachiticus&search_param=%28%28common_name%3D%27Sceloporus+malachiticus%27%29%29
18	25	Green spiny lizard	Sceloporus malachiticus	Squamata	Lizard	USA	America	presumably CB	https://reptile-database.reptarium.cz/species?genus=Sceloporus&species=malachiticus&search_param=%28%28common_name%3D%27Sceloporus+malachiticus%27%29%29
19	pre-trial	Florida softshel turtle	Apalone ferox	Testudines	Turtle	USA	America	FB	https://reptile-database.reptarium.cz/species?genus=Apalone&species=ferox&search_param=%28%28common_name%3D%27Apalone+ferox%27%29%29

Table S1: Animal species sampled in this study - country origin and categorization as captive bred (CB), farm bred (FB) and wild-caught (WC)

Shipment No.	Sample batch	Animal species	Scientific name of animal/s	Order	Suborder	Country origin	Continent	Captive bred (CB), farm bred (FB), wild-caught (WC)	Web URL used for assigning animal species to categories CB, FB and WC
19	pre-trial	Florida softshel turtle	Apalone ferox	Testudines	Turtle	USA	America	FB	https://reptile-database.reptarium.cz/species?genus=Apalone&species=ferox&search_param=%28%28common_name%3D%27Apalone+ferox%27%29%29
19	pre-trial	Florida softshel turtle	Apalone ferox	Testudines	Turtle	USA	America	FB	https://reptile-database.reptarium.cz/species?genus=Apalone&species=ferox&search_param=%28%28common_name%3D%27Apalone+ferox%27%29%29
19	pre-trial	Florida softshel turtle	Apalone ferox	Testudines	Turtle	USA	America	FB	https://reptile-database.reptarium.cz/species?genus=Apalone&species=ferox&search_param=%28%28common_name%3D%27Apalone+ferox%27%29%29
19	pre-trial	Cumberland slider	Trachemys scripta troosti	Testudines	Turtle	USA	America	FB	https://reptile-database.reptarium.cz/species?genus=Trachemys&species=scripta&search_param=%28%28common_name%3D%27Trachemys+scripta+scripta%27%29%29
19	pre-trial	Cumberland slider	Trachemys scripta troosti	Testudines	Turtle	USA	America	FB	https://reptile-database.reptarium.cz/species?genus=Trachemys&species=scripta&search_param=%28%28common_name%3D%27Trachemys+scripta+scripta%27%29%29
19	pre-trial	Cumberland slider	Trachemys scripta troosti	Testudines	Turtle	USA	America	FB	https://reptile-database.reptarium.cz/species?genus=Trachemys&species=scripta&search_param=%28%28common_name%3D%27Trachemys+scripta+scripta%27%29%29
19	pre-trial	Cumberland slider	Trachemys scripta troosti	Testudines	Turtle	USA	America	FB	https://reptile-database.reptarium.cz/species?genus=Trachemys&species=scripta&search_param=%28%28common_name%3D%27Trachemys+scripta+scripta%27%29%29
19	pre-trial	Cumberland slider	Trachemys scripta troosti	Testudines	Turtle	USA	America	FB	https://reptile-database.reptarium.cz/species?genus=Trachemys&species=scripta&search_param=%28%28common_name%3D%27Trachemys+scripta+scripta%27%29%29
19	pre-trial	Cumberland slider	Trachemys scripta troosti	Testudines	Turtle	USA	America	FB	https://reptile-database.reptarium.cz/species?genus=Trachemys&species=scripta&search_param=%28%28common_name%3D%27Trachemys+scripta+scripta%27%29%29
19	pre-trial	Cumberland slider	Trachemys scripta troosti	Testudines	Turtle	USA	America	FB	https://reptile-database.reptarium.cz/species?genus=Trachemys&species=scripta&search_param=%28%28common_name%3D%27Trachemys+scripta+scripta%27%29%29
20	26	Diploglossus monotropis	Diploglossus monotropis	Squamata	Lizard	Panama	America	WC/CB	https://reptile-database.reptarium.cz/species?genus=Diploglossus&species=monotropis&search_param=%28%28common_name%3D%27Diploglossus+monotropis%27%29%29
20	26	Diploglossus monotropis	Diploglossus monotropis	Squamata	Lizard	Panama	America	WC/CB	https://reptile-database.reptarium.cz/species?genus=Diploglossus&species=monotropis&search_param=%28%28common_name%3D%27Diploglossus+monotropis%27%29%29

Table S1: Animal species sampled in this study - country origin and categorization as captive bred (CB), farm bred (FB) and wild-caught (WC)

Shipment No.	Sample batch	Animal species	Scientific name of animal/s	Order	Suborder	Country origin	Continent	Captive bred (CB), farm bred (FB), wild-caught (WC)	Web URL used for assigning animal species to categories CB, FB and WC
20	26	Diploglossus monotropis	Diploglossus monotropis	Squamata	Lizard	Panama	America	WC/CB	https://reptile-database.reptarium.cz/species?genus=Diploglossus&species=monotropis&search_param=%28%28common_name%3D%27Diploglossus+monotropis%27%29%29
20	26	Diploglossus monotropis	Diploglossus monotropis	Squamata	Lizard	Panama	America	WC/CB	https://reptile-database.reptarium.cz/species?genus=Diploglossus&species=monotropis&search_param=%28%28common_name%3D%27Diploglossus+monotropis%27%29%29
20	26	Diploglossus monotropis	Diploglossus monotropis	Squamata	Lizard	Panama	America	WC/CB	https://reptile-database.reptarium.cz/species?genus=Diploglossus&species=monotropis&search_param=%28%28common_name%3D%27Diploglossus+monotropis%27%29%29
20	26	Diploglossus monotropis	Diploglossus monotropis	Squamata	Lizard	Panama	America	WC/CB	https://reptile-database.reptarium.cz/species?genus=Diploglossus&species=monotropis&search_param=%28%28common_name%3D%27Diploglossus+monotropis%27%29%29
20	26	Diploglossus monotropis	Diploglossus monotropis	Squamata	Lizard	Panama	America	WC/CB	https://reptile-database.reptarium.cz/species?genus=Diploglossus&species=monotropis&search_param=%28%28common_name%3D%27Diploglossus+monotropis%27%29%29
20	26	Diploglossus monotropis	Diploglossus monotropis	Squamata	Lizard	Panama	America	WC/CB	https://reptile-database.reptarium.cz/species?genus=Diploglossus&species=monotropis&search_param=%28%28common_name%3D%27Diploglossus+monotropis%27%29%29
20	26	Diploglossus monotropis	Diploglossus monotropis	Squamata	Lizard	Panama	America	WC/CB	https://reptile-database.reptarium.cz/species?genus=Diploglossus&species=monotropis&search_param=%28%28common_name%3D%27Diploglossus+monotropis%27%29%29
20	26	Diploglossus monotropis	Diploglossus monotropis	Squamata	Lizard	Panama	America	WC/CB	https://reptile-database.reptarium.cz/species?genus=Diploglossus&species=monotropis&search_param=%28%28common_name%3D%27Diploglossus+monotropis%27%29%29
20	26	Diploglossus monotropis	Diploglossus monotropis	Squamata	Lizard	Panama	America	WC/CB	https://reptile-database.reptarium.cz/species?genus=Diploglossus&species=monotropis&search_param=%28%28common_name%3D%27Diploglossus+monotropis%27%29%29
20	26	Diploglossus monotropis	Diploglossus monotropis	Squamata	Lizard	Panama	America	WC/CB	https://reptile-database.reptarium.cz/species?genus=Diploglossus&species=monotropis&search_param=%28%28common_name%3D%27Diploglossus+monotropis%27%29%29
20	26	Diploglossus monotropis	Diploglossus monotropis	Squamata	Lizard	Panama	America	WC/CB	https://reptile-database.reptarium.cz/species?genus=Diploglossus&species=monotropis&search_param=%28%28common_name%3D%27Diploglossus+monotropis%27%29%29
20	26	Diploglossus monotropis	Diploglossus monotropis	Squamata	Lizard	Panama	America	WC/CB	https://reptile-database.reptarium.cz/species?genus=Diploglossus&species=monotropis&search_param=%28%28common_name%3D%27Diploglossus+monotropis%27%29%29

Table S1: Animal species sampled in this study - country origin and categorization as captive bred (CB), farm bred (FB) and wild-caught (WC)

Shipment No.	Sample batch	Animal species	Scientific name of animal/s	Order	Suborder	Country origin	Continent	Captive bred (CB), farm bred (FB), wild-caught (WC)	Web URL used for assigning animal species to categories CB, FB and WC
20	26	Diploglossus monotropis	Diploglossus monotropis	Squamata	Lizard	Panama	America	WC/CB	https://reptile-database.reptarium.cz/species?genus=Diploglossus&species=monotropis&search_param=%28%28common_name%3D%27Diploglossus+monotropis%27%29%29
21	27	Common leopard gecko	Eublepharis macularius	Squamata	Lizard	Canada	America	CB	https://reptile-database.reptarium.cz/species?genus=Eublepharis&species=macularius&search_param=%28%28common_name%3D%27Eublepharis+macularius%27%29%29
21	27	Common leopard gecko	Eublepharis macularius	Squamata	Lizard	Canada	America	CB	https://reptile-database.reptarium.cz/species?genus=Eublepharis&species=macularius&search_param=%28%28common_name%3D%27Eublepharis+macularius%27%29%29
21	27	Common leopard gecko	Eublepharis macularius	Squamata	Lizard	Canada	America	CB	https://reptile-database.reptarium.cz/species?genus=Eublepharis&species=macularius&search_param=%28%28common_name%3D%27Eublepharis+macularius%27%29%29
21	27	Common leopard gecko	Eublepharis macularius	Squamata	Lizard	Canada	America	CB	https://reptile-database.reptarium.cz/species?genus=Eublepharis&species=macularius&search_param=%28%28common_name%3D%27Eublepharis+macularius%27%29%29
21	27	Common leopard gecko	Eublepharis macularius	Squamata	Lizard	Canada	America	CB	https://reptile-database.reptarium.cz/species?genus=Eublepharis&species=macularius&search_param=%28%28common_name%3D%27Eublepharis+macularius%27%29%29
21	27	Common leopard gecko	Eublepharis macularius	Squamata	Lizard	Canada	America	CB	https://reptile-database.reptarium.cz/species?genus=Eublepharis&species=macularius&search_param=%28%28common_name%3D%27Eublepharis+macularius%27%29%29
21	27	Common leopard gecko	Eublepharis macularius	Squamata	Lizard	Canada	America	CB	https://reptile-database.reptarium.cz/species?genus=Eublepharis&species=macularius&search_param=%28%28common_name%3D%27Eublepharis+macularius%27%29%29
21	28	Crested gecko	Correlophus ciliatus	Squamata	Lizard	Canada	America	CB	https://reptile-database.reptarium.cz/species?genus=Correlophus&species=ciliatus&search_param=%28%28common_name%3D%27Correlophus+ciliatus%27%29%29
21	28	Crested gecko	Correlophus ciliatus	Squamata	Lizard	Canada	America	CB	https://reptile-database.reptarium.cz/species?genus=Correlophus&species=ciliatus&search_param=%28%28common_name%3D%27Correlophus+ciliatus%27%29%29
21	28	Crested gecko	Correlophus ciliatus	Squamata	Lizard	Canada	America	CB	https://reptile-database.reptarium.cz/species?genus=Correlophus&species=ciliatus&search_param=%28%28common_name%3D%27Correlophus+ciliatus%27%29%29
21	28	Crested gecko	Correlophus ciliatus	Squamata	Lizard	Canada	America	CB	https://reptile-database.reptarium.cz/species?genus=Correlophus&species=ciliatus&search_param=%28%28common_name%3D%27Correlophus+ciliatus%27%29%29

Table S1: Animal species sampled in this study - country origin and categorization as captive bred (CB), farm bred (FB) and wild-caught (WC)

Shipment No.	Sample batch	Animal species	Scientific name of animal/s	Order	Suborder	Country origin	Continent	Captive bred (CB), farm bred (FB), wild-caught (WC)	Web URL used for assigning animal species to categories CB, FB and WC
21	28	Crested gecko	Correlophus ciliatus	Squamata	Lizard	Canada	America	CB	https://reptile-database.reptarium.cz/species?genus=Correlophus&species=ciliatus&search_param=%28%28common_name%3D%27Correlophus+ciliatus%27%29%29
21	28	Crested gecko	Correlophus ciliatus	Squamata	Lizard	Canada	America	CB	https://reptile-database.reptarium.cz/species?genus=Correlophus&species=ciliatus&search_param=%28%28common_name%3D%27Correlophus+ciliatus%27%29%29
21	28	Crested gecko	Correlophus ciliatus	Squamata	Lizard	Canada	America	CB	https://reptile-database.reptarium.cz/species?genus=Correlophus&species=ciliatus&search_param=%28%28common_name%3D%27Correlophus+ciliatus%27%29%29
21	28	Crested gecko	Correlophus ciliatus	Squamata	Lizard	Canada	America	CB	https://reptile-database.reptarium.cz/species?genus=Correlophus&species=ciliatus&search_param=%28%28common_name%3D%27Correlophus+ciliatus%27%29%29
21	28	Crested gecko	Correlophus ciliatus	Squamata	Lizard	Canada	America	CB	https://reptile-database.reptarium.cz/species?genus=Correlophus&species=ciliatus&search_param=%28%28common_name%3D%27Correlophus+ciliatus%27%29%29
21	28	Crested gecko	Correlophus ciliatus	Squamata	Lizard	Canada	America	CB	https://reptile-database.reptarium.cz/species?genus=Correlophus&species=ciliatus&search_param=%28%28common_name%3D%27Correlophus+ciliatus%27%29%29
21	28	Crested gecko	Correlophus ciliatus	Squamata	Lizard	Canada	America	CB	https://reptile-database.reptarium.cz/species?genus=Correlophus&species=ciliatus&search_param=%28%28common_name%3D%27Correlophus+ciliatus%27%29%29
21	28	Crested gecko	Correlophus ciliatus	Squamata	Lizard	Canada	America	CB	https://reptile-database.reptarium.cz/species?genus=Correlophus&species=ciliatus&search_param=%28%28common_name%3D%27Correlophus+ciliatus%27%29%29
21	28	Crested gecko	Correlophus ciliatus	Squamata	Lizard	Canada	America	CB	https://reptile-database.reptarium.cz/species?genus=Correlophus&species=ciliatus&search_param=%28%28common_name%3D%27Correlophus+ciliatus%27%29%29
21	27	Common leopard gecko	Eublepharis macularius	Squamata	Lizard	Canada	America	CB	https://reptile-database.reptarium.cz/species?genus=Eublepharis&species=macularius&search_param=%28%28common_name%3D%27Eublepharis+macularius%27%29%29
21	27	Common leopard gecko	Eublepharis macularius	Squamata	Lizard	Canada	America	CB	https://reptile-database.reptarium.cz/species?genus=Eublepharis&species=macularius&search_param=%28%28common_name%3D%27Eublepharis+macularius%27%29%29
21	27	Common leopard gecko	Eublepharis macularius	Squamata	Lizard	Canada	America	CB	https://reptile-database.reptarium.cz/species?genus=Eublepharis&species=macularius&search_param=%28%28common_name%3D%27Eublepharis+macularius%27%29%29

Table S1: Animal species sampled in this study - country origin and categorization as captive bred (CB), farm bred (FB) and wild-caught (WC)

Shipment No.	Sample batch	Animal species	Scientific name of animal/s	Order	Suborder	Country origin	Continent	Captive bred (CB), farm bred (FB), wild-caught (WC)	Web URL used for assigning animal species to categories CB, FB and WC
21	27	Common leopard gecko	Eublepharis macularius	Squamata	Lizard	Canada	America	CB	https://reptile-database.reptarium.cz/species?genus=Eublepharis&species=macularius&search_param=%28%28common_name%3D%27Eublepharis+macularius%27%29%29
21	27	Common leopard gecko	Eublepharis macularius	Squamata	Lizard	Canada	America	CB	https://reptile-database.reptarium.cz/species?genus=Eublepharis&species=macularius&search_param=%28%28common_name%3D%27Eublepharis+macularius%27%29%29
21	27	Common leopard gecko	Eublepharis macularius	Squamata	Lizard	Canada	America	CB	https://reptile-database.reptarium.cz/species?genus=Eublepharis&species=macularius&search_param=%28%28common_name%3D%27Eublepharis+macularius%27%29%29
21	27	Common leopard gecko	Eublepharis macularius	Squamata	Lizard	Canada	America	CB	https://reptile-database.reptarium.cz/species?genus=Eublepharis&species=macularius&search_param=%28%28common_name%3D%27Eublepharis+macularius%27%29%29
21	27	Common leopard gecko	Eublepharis macularius	Squamata	Lizard	Canada	America	CB	https://reptile-database.reptarium.cz/species?genus=Eublepharis&species=macularius&search_param=%28%28common_name%3D%27Eublepharis+macularius%27%29%29
21	27	Common leopard gecko	Eublepharis macularius	Squamata	Lizard	Canada	America	CB	https://reptile-database.reptarium.cz/species?genus=Eublepharis&species=macularius&search_param=%28%28common_name%3D%27Eublepharis+macularius%27%29%29
21	27	Common leopard gecko	Eublepharis macularius	Squamata	Lizard	Canada	America	CB	https://reptile-database.reptarium.cz/species?genus=Eublepharis&species=macularius&search_param=%28%28common_name%3D%27Eublepharis+macularius%27%29%29
21	27	Common leopard gecko	Eublepharis macularius	Squamata	Lizard	Canada	America	CB	https://reptile-database.reptarium.cz/species?genus=Eublepharis&species=macularius&search_param=%28%28common_name%3D%27Eublepharis+macularius%27%29%29
22	29	Boa constrictor	Boa constrictor	Squamata	Snake	USA	America	CB	https://reptile-database.reptarium.cz/species?genus=Boa&species=constrictor&search_param=%28%28common_name%3D%27Boa+constrictor%27%29%29
22	29	Boa constrictor	Boa constrictor	Squamata	Snake	USA	America	CB	https://reptile-database.reptarium.cz/species?genus=Boa&species=constrictor&search_param=%28%28common_name%3D%27Boa+constrictor%27%29%29
22	29	Boa constrictor	Boa constrictor	Squamata	Snake	USA	America	CB	https://reptile-database.reptarium.cz/species?genus=Boa&species=constrictor&search_param=%28%28common_name%3D%27Boa+constrictor%27%29%29
22	29	Boa constrictor	Boa constrictor	Squamata	Snake	USA	America	CB	https://reptile-database.reptarium.cz/species?genus=Boa&species=constrictor&search_param=%28%28common_name%3D%27Boa+constrictor%27%29%29

Table S1: Animal species sampled in this study - country origin and categorization as captive bred (CB), farm bred (FB) and wild-caught (WC)

Shipment No.	Sample batch	Animal species	Scientific name of animal/s	Order	Suborder	Country origin	Continent	Captive bred (CB), farm bred (FB), wild-caught (WC)	Web URL used for assigning animal species to categories CB, FB and WC
22	29	Boa constrictor	Boa constrictor	Squamata	Snake	USA	America	CB	https://reptile-database.reptarium.cz/species?genus=Boa&species=constrictor&search_param=%28%28common_name%3D%27Boa+constrictor%27%29%29
22	29	Boa constrictor	Boa constrictor	Squamata	Snake	USA	America	CB	https://reptile-database.reptarium.cz/species?genus=Boa&species=constrictor&search_param=%28%28common_name%3D%27Boa+constrictor%27%29%29
22	29	Boa constrictor	Boa constrictor	Squamata	Snake	USA	America	CB	https://reptile-database.reptarium.cz/species?genus=Boa&species=constrictor&search_param=%28%28common_name%3D%27Boa+constrictor%27%29%29
22	29	Boa constrictor	Boa constrictor	Squamata	Snake	USA	America	CB	https://reptile-database.reptarium.cz/species?genus=Boa&species=constrictor&search_param=%28%28common_name%3D%27Boa+constrictor%27%29%29
22	29	Boa constrictor	Boa constrictor	Squamata	Snake	USA	America	CB	https://reptile-database.reptarium.cz/species?genus=Boa&species=constrictor&search_param=%28%28common_name%3D%27Boa+constrictor%27%29%29
22	29	Boa constrictor	Boa constrictor	Squamata	Snake	USA	America	CB	https://reptile-database.reptarium.cz/species?genus=Boa&species=constrictor&search_param=%28%28common_name%3D%27Boa+constrictor%27%29%29
23	30	Eastern collared lizard	Crotaphytus insularis	Squamata	Lizard	USA	America	WC	https://reptile-database.reptarium.cz/species?genus=Crotaphytus&species=insularis&search_param=%28%28common_name%3D%27Crotaphytus+collaris%27%29%29
23	30	Eastern collared lizard	Crotaphytus insularis	Squamata	Lizard	USA	America	WC	https://reptile-database.reptarium.cz/species?genus=Crotaphytus&species=insularis&search_param=%28%28common_name%3D%27Crotaphytus+collaris%27%29%29
23	30	Eastern collared lizard	Crotaphytus insularis	Squamata	Lizard	USA	America	WC	https://reptile-database.reptarium.cz/species?genus=Crotaphytus&species=insularis&search_param=%28%28common_name%3D%27Crotaphytus+collaris%27%29%29
23	30	Eastern collared lizard	Crotaphytus insularis	Squamata	Lizard	USA	America	WC	https://reptile-database.reptarium.cz/species?genus=Crotaphytus&species=insularis&search_param=%28%28common_name%3D%27Crotaphytus+collaris%27%29%29
23	30	Eastern collared lizard	Crotaphytus insularis	Squamata	Lizard	USA	America	WC	https://reptile-database.reptarium.cz/species?genus=Crotaphytus&species=insularis&search_param=%28%28common_name%3D%27Crotaphytus+collaris%27%29%29
23	30	Eastern collared lizard	Crotaphytus insularis	Squamata	Lizard	USA	America	WC	https://reptile-database.reptarium.cz/species?genus=Crotaphytus&species=insularis&search_param=%28%28common_name%3D%27Crotaphytus+collaris%27%29%29

Table S1: Animal species sampled in this study - country origin and categorization as captive bred (CB), farm bred (FB) and wild-caught (WC)

Shipment No.	Sample batch	Animal species	Scientific name of animal/s	Order	Suborder	Country origin	Continent	Captive bred (CB), farm bred (FB), wild-caught (WC)	Web URL used for assigning animal species to categories CB, FB and WC
23	30	Eastern collared lizard	Crotaphytus insularis	Squamata	Lizard	USA	America	WC	https://reptile-database.reptarium.cz/species?genus=Crotaphytus&species=insularis&search_param=%28%28common_name%3D%27Crotaphytus+collaris%27%29%29
23	30	Eastern collared lizard	Crotaphytus insularis	Squamata	Lizard	USA	America	WC	https://reptile-database.reptarium.cz/species?genus=Crotaphytus&species=insularis&search_param=%28%28common_name%3D%27Crotaphytus+collaris%27%29%29
23	30	Eastern collared lizard	Crotaphytus insularis	Squamata	Lizard	USA	America	WC	https://reptile-database.reptarium.cz/species?genus=Crotaphytus&species=insularis&search_param=%28%28common_name%3D%27Crotaphytus+collaris%27%29%29
23	30	Eastern collared lizard	Crotaphytus insularis	Squamata	Lizard	USA	America	WC	https://reptile-database.reptarium.cz/species?genus=Crotaphytus&species=insularis&search_param=%28%28common_name%3D%27Crotaphytus+collaris%27%29%29
23	31	Green anole	Anolis carolinensis	Squamata	Lizard	USA	America	WC/FB	https://reptile-database.reptarium.cz/species?genus=Anolis&species=carolinensis&search_param=%28%28common_name%3D%27Anolis+carolinensis%27%29%29
23	31	Green anole	Anolis carolinensis	Squamata	Lizard	USA	America	WC/FB	https://reptile-database.reptarium.cz/species?genus=Anolis&species=carolinensis&search_param=%28%28common_name%3D%27Anolis+carolinensis%27%29%29
23	31	Green anole	Anolis carolinensis	Squamata	Lizard	USA	America	WC/FB	https://reptile-database.reptarium.cz/species?genus=Anolis&species=carolinensis&search_param=%28%28common_name%3D%27Anolis+carolinensis%27%29%29
23	31	Green anole	Anolis carolinensis	Squamata	Lizard	USA	America	WC/FB	https://reptile-database.reptarium.cz/species?genus=Anolis&species=carolinensis&search_param=%28%28common_name%3D%27Anolis+carolinensis%27%29%29
24	32	New Caledonia giant gecko	Rhacodactylus leachianus	Squamata	Lizard	USA	America	CB	https://reptile-database.reptarium.cz/species?genus=Rhacodactylus&species=leachianus&search_param=%28%28common_name%3D%27Rhacodactylus+leachianus%27%29%29
24	32	New Caledonia giant gecko	Rhacodactylus leachianus	Squamata	Lizard	USA	America	CB	https://reptile-database.reptarium.cz/species?genus=Rhacodactylus&species=leachianus&search_param=%28%28common_name%3D%27Rhacodactylus+leachianus%27%29%29
24	32	New Caledonia giant gecko	Rhacodactylus leachianus	Squamata	Lizard	USA	America	CB	https://reptile-database.reptarium.cz/species?genus=Rhacodactylus&species=leachianus&search_param=%28%28common_name%3D%27Rhacodactylus+leachianus%27%29%29
24	32	New Caledonia giant gecko	Rhacodactylus leachianus	Squamata	Lizard	USA	America	CB	https://reptile-database.reptarium.cz/species?genus=Rhacodactylus&species=leachianus&search_param=%28%28common_name%3D%27Rhacodactylus+leachianus%27%29%29

Table S1: Animal species sampled in this study - country origin and categorization as captive bred (CB), farm bred (FB) and wild-caught (WC)

Shipment No.	Sample batch	Animal species	Scientific name of animal/s	Order	Suborder	Country origin	Continent	Captive bred (CB), farm bred (FB), wild-caught (WC)	Web URL used for assigning animal species to categories CB, FB and WC
24	32	New Caledonia giant gecko	Rhacodactylus leachianus	Squamata	Lizard	USA	America	CB	https://reptile-database.reptarium.cz/species?genus=Rhacodactylus&species=leachianus&search_param=%28%28common_name%3D%27Rhacodactylus+leachianus%27%29%29
24	32	New Caledonia giant gecko	Rhacodactylus leachianus	Squamata	Lizard	USA	America	CB	https://reptile-database.reptarium.cz/species?genus=Rhacodactylus&species=leachianus&search_param=%28%28common_name%3D%27Rhacodactylus+leachianus%27%29%29
24	32	New Caledonia giant gecko	Rhacodactylus leachianus	Squamata	Lizard	USA	America	CB	https://reptile-database.reptarium.cz/species?genus=Rhacodactylus&species=leachianus&search_param=%28%28common_name%3D%27Rhacodactylus+leachianus%27%29%29
24	32	New Caledonia giant gecko	Rhacodactylus leachianus	Squamata	Lizard	USA	America	CB	https://reptile-database.reptarium.cz/species?genus=Rhacodactylus&species=leachianus&search_param=%28%28common_name%3D%27Rhacodactylus+leachianus%27%29%29
24	32	New Caledonia giant gecko	Rhacodactylus leachianus	Squamata	Lizard	USA	America	CB	https://reptile-database.reptarium.cz/species?genus=Rhacodactylus&species=leachianus&search_param=%28%28common_name%3D%27Rhacodactylus+leachianus%27%29%29
24	32	New Caledonia giant gecko	Rhacodactylus leachianus	Squamata	Lizard	USA	America	CB	https://reptile-database.reptarium.cz/species?genus=Rhacodactylus&species=leachianus&search_param=%28%28common_name%3D%27Rhacodactylus+leachianus%27%29%29
24	32	New Caledonia giant gecko	Rhacodactylus leachianus	Squamata	Lizard	USA	America	CB	https://reptile-database.reptarium.cz/species?genus=Rhacodactylus&species=leachianus&search_param=%28%28common_name%3D%27Rhacodactylus+leachianus%27%29%29
24	33	Gopher snake	Pituophis catenifer	Squamata	Snake	USA	America	CB	https://reptile-database.reptarium.cz/species?genus=Pituophis&species=catenifer&search_param=%28%28common_name%3D%27Pituophis+catenifer%27%29%29
24	33	Gopher snake	Pituophis catenifer	Squamata	Snake	USA	America	CB	https://reptile-database.reptarium.cz/species?genus=Pituophis&species=catenifer&search_param=%28%28common_name%3D%27Pituophis+catenifer%27%29%29
24	33	Gopher snake	Pituophis catenifer	Squamata	Snake	USA	America	CB	https://reptile-database.reptarium.cz/species?genus=Pituophis&species=catenifer&search_param=%28%28common_name%3D%27Pituophis+catenifer%27%29%29
24	33	Gopher snake	Pituophis catenifer	Squamata	Snake	USA	America	CB	https://reptile-database.reptarium.cz/species?genus=Pituophis&species=catenifer&search_param=%28%28common_name%3D%27Pituophis+catenifer%27%29%29
24	33	Gopher snake	Pituophis catenifer	Squamata	Snake	USA	America	CB	https://reptile-database.reptarium.cz/species?genus=Pituophis&species=catenifer&search_param=%28%28common_name%3D%27Pituophis+catenifer%27%29%29

Table S1: Animal species sampled in this study - country origin and categorization as captive bred (CB), farm bred (FB) and wild-caught (WC)

Shipment No.	Sample batch	Animal species	Scientific name of animal/s	Order	Suborder	Country origin	Continent	Captive bred (CB), farm bred (FB), wild-caught (WC)	Web URL used for assigning animal species to categories CB, FB and WC
24	33	Gopher snake	Pituophis catenifer	Squamata	Snake	USA	America	CB	https://reptile-database.reptarium.cz/species?genus=Pituophis&species=catenifer&search_param=%28%28common_name%3D%27Pituophis+catenifer%27%29%29
24	33	Gopher snake	Pituophis catenifer	Squamata	Snake	USA	America	CB	https://reptile-database.reptarium.cz/species?genus=Pituophis&species=catenifer&search_param=%28%28common_name%3D%27Pituophis+catenifer%27%29%29
25	34	Common musk turtle	Sternotherus odoratus	Testudines	Turtle	USA	America	FB	https://reptile-database.reptarium.cz/species?genus=Sternotherus&species=odoratus&search_param=%28%28common_name%3D%27Sternotherus+odoratus%27%29%29
25	34	Common musk turtle	Sternotherus odoratus	Testudines	Turtle	USA	America	FB	https://reptile-database.reptarium.cz/species?genus=Sternotherus&species=odoratus&search_param=%28%28common_name%3D%27Sternotherus+odoratus%27%29%29
25	34	Common musk turtle	Sternotherus odoratus	Testudines	Turtle	USA	America	FB	https://reptile-database.reptarium.cz/species?genus=Sternotherus&species=odoratus&search_param=%28%28common_name%3D%27Sternotherus+odoratus%27%29%29
25	34	Common musk turtle	Sternotherus odoratus	Testudines	Turtle	USA	America	FB	https://reptile-database.reptarium.cz/species?genus=Sternotherus&species=odoratus&search_param=%28%28common_name%3D%27Sternotherus+odoratus%27%29%29
25	34	Common musk turtle	Sternotherus odoratus	Testudines	Turtle	USA	America	FB	https://reptile-database.reptarium.cz/species?genus=Sternotherus&species=odoratus&search_param=%28%28common_name%3D%27Sternotherus+odoratus%27%29%29
25	34	Common musk turtle	Sternotherus odoratus	Testudines	Turtle	USA	America	FB	https://reptile-database.reptarium.cz/species?genus=Sternotherus&species=odoratus&search_param=%28%28common_name%3D%27Sternotherus+odoratus%27%29%29
25	34	Common musk turtle	Sternotherus odoratus	Testudines	Turtle	USA	America	FB	https://reptile-database.reptarium.cz/species?genus=Sternotherus&species=odoratus&search_param=%28%28common_name%3D%27Sternotherus+odoratus%27%29%29
25	35	Common green iguana	Iguana iguana	Squamata	Lizard	USA	America	WC/FB	https://reptile-database.reptarium.cz/species?genus=Iguana&species=iguana&search_param=%28%28common_name%3D%27Iguana+iguana%27%29%29
25	35	Common green iguana	Iguana iguana	Squamata	Lizard	USA	America	WC/FB	https://reptile-database.reptarium.cz/species?genus=Iguana&species=iguana&search_param=%28%28common_name%3D%27Iguana+iguana%27%29%29
25	35	Common green iguana	Iguana iguana	Squamata	Lizard	USA	America	WC/FB	https://reptile-database.reptarium.cz/species?genus=Iguana&species=iguana&search_param=%28%28common_name%3D%27Iguana+iguana%27%29%29

Table S1: Animal species sampled in this study - country origin and categorization as captive bred (CB), farm bred (FB) and wild-caught (WC)

Shipment No.	Sample batch	Animal species	Scientific name of animal/s	Order	Suborder	Country origin	Continent	Captive bred (CB), farm bred (FB), wild-caught (WC)	Web URL used for assigning animal species to categories CB, FB and WC
25	35	Common green iguana	Iguana iguana	Squamata	Lizard	USA	America	WC/FB	https://reptile-database.reptarium.cz/species?genus=Iguana&species=iguana&search_param=%28%28common_name%3D%27Iguana+iguana%27%29%29
25	35	Common green iguana	Iguana iguana	Squamata	Lizard	USA	America	WC/FB	https://reptile-database.reptarium.cz/species?genus=Iguana&species=iguana&search_param=%28%28common_name%3D%27Iguana+iguana%27%29%29
25	35	Common green iguana	Iguana iguana	Squamata	Lizard	USA	America	WC/FB	https://reptile-database.reptarium.cz/species?genus=Iguana&species=iguana&search_param=%28%28common_name%3D%27Iguana+iguana%27%29%29
25	36	Green anole	Anolis carolinensis	Squamata	Lizard	USA	America	WC/FB	https://reptile-database.reptarium.cz/species?genus=Anolis&species=carolinensis&search_param=%28%28common_name%3D%27Anolis+carolinensis%27%29%29
25	36	Green anole	Anolis carolinensis	Squamata	Lizard	USA	America	WC/FB	https://reptile-database.reptarium.cz/species?genus=Anolis&species=carolinensis&search_param=%28%28common_name%3D%27Anolis+carolinensis%27%29%29
25	37	Cuban giant anole	Anolis equestris	Squamata	Lizard	USA	America	WC/FB	https://reptile-database.reptarium.cz/species?genus=Anolis&species=equestris&search_param=%28%28common_name%3D%27Anolis+equestris%27%29%29
25	37	Cuban giant anole	Anolis equestris	Squamata	Lizard	USA	America	WC/FB	https://reptile-database.reptarium.cz/species?genus=Anolis&species=equestris&search_param=%28%28common_name%3D%27Anolis+equestris%27%29%29
25	37	Cuban giant anole	Anolis equestris	Squamata	Lizard	USA	America	WC/FB	https://reptile-database.reptarium.cz/species?genus=Anolis&species=equestris&search_param=%28%28common_name%3D%27Anolis+equestris%27%29%29
25	37	Cuban giant anole	Anolis equestris	Squamata	Lizard	USA	America	WC/FB	https://reptile-database.reptarium.cz/species?genus=Anolis&species=equestris&search_param=%28%28common_name%3D%27Anolis+equestris%27%29%29
25	37	Cuban giant anole	Anolis equestris	Squamata	Lizard	USA	America	WC/FB	https://reptile-database.reptarium.cz/species?genus=Anolis&species=equestris&search_param=%28%28common_name%3D%27Anolis+equestris%27%29%29
25	37	Cuban giant anole	Anolis equestris	Squamata	Lizard	USA	America	WC/FB	https://reptile-database.reptarium.cz/species?genus=Anolis&species=equestris&search_param=%28%28common_name%3D%27Anolis+equestris%27%29%29
25	37	Cuban giant anole	Anolis equestris	Squamata	Lizard	USA	America	WC/FB	https://reptile-database.reptarium.cz/species?genus=Anolis&species=equestris&search_param=%28%28common_name%3D%27Anolis+equestris%27%29%29

Table S1: Animal species sampled in this study - country origin and categorization as captive bred (CB), farm bred (FB) and wild-caught (WC)

Shipment No.	Sample batch	Animal species	Scientific name of animal/s	Order	Suborder	Country origin	Continent	Captive bred (CB), farm bred (FB), wild-caught (WC)	Web URL used for assigning animal species to categories CB, FB and WC
25	37	Cuban giant anole	Anolis equestris	Squamata	Lizard	USA	America	WC/FB	https://reptile-database.reptarium.cz/species?genus=Anolis&species=equestris&search_param=%28%28common_name%3D%27Anolis+equestris%27%29%29
25	37	Cuban giant anole	Anolis equestris	Squamata	Lizard	USA	America	WC/FB	https://reptile-database.reptarium.cz/species?genus=Anolis&species=equestris&search_param=%28%28common_name%3D%27Anolis+equestris%27%29%29
25	37	Cuban giant anole	Anolis equestris	Squamata	Lizard	USA	America	WC/FB	https://reptile-database.reptarium.cz/species?genus=Anolis&species=equestris&search_param=%28%28common_name%3D%27Anolis+equestris%27%29%29
25	38	Cuban giant anole	Anolis equestris	Squamata	Lizard	USA	America	WC/FB	https://reptile-database.reptarium.cz/species?genus=Anolis&species=equestris&search_param=%28%28common_name%3D%27Anolis+equestris%27%29%29
25	38	Cuban giant anole	Anolis equestris	Squamata	Lizard	USA	America	WC/FB	https://reptile-database.reptarium.cz/species?genus=Anolis&species=equestris&search_param=%28%28common_name%3D%27Anolis+equestris%27%29%29
25	38	Cuban giant anole	Anolis equestris	Squamata	Lizard	USA	America	WC/FB	https://reptile-database.reptarium.cz/species?genus=Anolis&species=equestris&search_param=%28%28common_name%3D%27Anolis+equestris%27%29%29
25	38	Cuban giant anole	Anolis equestris	Squamata	Lizard	USA	America	WC/FB	https://reptile-database.reptarium.cz/species?genus=Anolis&species=equestris&search_param=%28%28common_name%3D%27Anolis+equestris%27%29%29
25	38	Cuban giant anole	Anolis equestris	Squamata	Lizard	USA	America	WC/FB	https://reptile-database.reptarium.cz/species?genus=Anolis&species=equestris&search_param=%28%28common_name%3D%27Anolis+equestris%27%29%29
26	38	Mediterranean spur-thighed tortoise	Testudo graeca	Testudines	Turtle	Turkey	Asia	WC/FB	https://www.academia.edu/4668767/Testudo_trade_in_Turkey
26	38	Mediterranean spur-thighed tortoise	Testudo graeca	Testudines	Turtle	Turkey	Asia	WC/FB	https://www.academia.edu/4668767/Testudo_trade_in_Turkey
26	38	Mediterranean spur-thighed tortoise	Testudo graeca	Testudines	Turtle	Turkey	Asia	WC/FB	https://www.academia.edu/4668767/Testudo_trade_in_Turkey
26	38	Mediterranean spur-thighed tortoise	Testudo graeca	Testudines	Turtle	Turkey	Asia	WC/FB	https://www.academia.edu/4668767/Testudo_trade_in_Turkey
26	38	Mediterranean spur-thighed tortoise	Testudo graeca	Testudines	Turtle	Turkey	Asia	WC/FB	https://www.academia.edu/4668767/Testudo_trade_in_Turkey
26	38	Mediterranean spur-thighed tortoise	Testudo graeca	Testudines	Turtle	Turkey	Asia	WC/FB	https://www.academia.edu/4668767/Testudo_trade_in_Turkey
26	39	Annam leaf turtle	Mauremys annamensis	Testudines	Turtle	Turkey	Asia	WC/FB	https://reptile-database.reptarium.cz/species?genus=Mauremys&species=annamensis&search_param=%28%28common_name%3D%27Mauremys+annamensis%27%29%29

Table S1: Animal species sampled in this study - country origin and categorization as captive bred (CB), farm bred (FB) and wild-caught (WC)

Shipment No.	Sample batch	Animal species	Scientific name of animal/s	Order	Suborder	Country origin	Continent	Captive bred (CB), farm bred (FB), wild-caught (WC)	Web URL used for assigning animal species to categories CB, FB and WC
26	39	Annam leaf turtle	Mauremys annamensis	Testudines	Turtle	Turkey	Asia	WC/FB	https://reptile-database.reptarium.cz/species?genus=Mauremys&species=annamensis&search_param=%28%28common_name%3D%27Mauremys+annamensis%27%29%29
26	39	Annam leaf turtle	Mauremys annamensis	Testudines	Turtle	Turkey	Asia	WC/FB	https://reptile-database.reptarium.cz/species?genus=Mauremys&species=annamensis&search_param=%28%28common_name%3D%27Mauremys+annamensis%27%29%29
27	40	Central bearded dragon	Pogona vitticeps	Squamata	Lizard	USA	America	CB	https://reptile-database.reptarium.cz/species?genus=Pogona&species=vitticeps&search_param=%28%28common_name%3D%27Pogona+vitticeps%27%29%29
27	40	Central bearded dragon	Pogona vitticeps	Squamata	Lizard	USA	America	CB	https://reptile-database.reptarium.cz/species?genus=Pogona&species=vitticeps&search_param=%28%28common_name%3D%27Pogona+vitticeps%27%29%29
27	40	Central bearded dragon	Pogona vitticeps	Squamata	Lizard	USA	America	CB	https://reptile-database.reptarium.cz/species?genus=Pogona&species=vitticeps&search_param=%28%28common_name%3D%27Pogona+vitticeps%27%29%29
27	40	Central bearded dragon	Pogona vitticeps	Squamata	Lizard	USA	America	CB	https://reptile-database.reptarium.cz/species?genus=Pogona&species=vitticeps&search_param=%28%28common_name%3D%27Pogona+vitticeps%27%29%29
27	40	Central bearded dragon	Pogona vitticeps	Squamata	Lizard	USA	America	CB	https://reptile-database.reptarium.cz/species?genus=Pogona&species=vitticeps&search_param=%28%28common_name%3D%27Pogona+vitticeps%27%29%29
27	40	Central bearded dragon	Pogona vitticeps	Squamata	Lizard	USA	America	CB	https://reptile-database.reptarium.cz/species?genus=Pogona&species=vitticeps&search_param=%28%28common_name%3D%27Pogona+vitticeps%27%29%29
27	40	Central bearded dragon	Pogona vitticeps	Squamata	Lizard	USA	America	CB	https://reptile-database.reptarium.cz/species?genus=Pogona&species=vitticeps&search_param=%28%28common_name%3D%27Pogona+vitticeps%27%29%29
27	40	Central bearded dragon	Pogona vitticeps	Squamata	Lizard	USA	America	CB	https://reptile-database.reptarium.cz/species?genus=Pogona&species=vitticeps&search_param=%28%28common_name%3D%27Pogona+vitticeps%27%29%29
27	40	Central bearded dragon	Pogona vitticeps	Squamata	Lizard	USA	America	CB	https://reptile-database.reptarium.cz/species?genus=Pogona&species=vitticeps&search_param=%28%28common_name%3D%27Pogona+vitticeps%27%29%29
27	40	Central bearded dragon	Pogona vitticeps	Squamata	Lizard	USA	America	CB	https://reptile-database.reptarium.cz/species?genus=Pogona&species=vitticeps&search_param=%28%28common_name%3D%27Pogona+vitticeps%27%29%29

Table S1: Animal species sampled in this study - country origin and categorization as captive bred (CB), farm bred (FB) and wild-caught (WC)

Shipment No.	Sample batch	Animal species	Scientific name of animal/s	Order	Suborder	Country origin	Continent	Captive bred (CB), farm bred (FB), wild-caught (WC)	Web URL used for assigning animal species to categories CB, FB and WC
27	40	Central bearded dragon	Pogona vitticeps	Squamata	Lizard	USA	America	CB	https://reptile-database.reptarium.cz/species?genus=Pogona&species=vitticeps&search_param=%28%28common_name%3D%27Pogona+vitticeps%27%29%29
27	40	Central bearded dragon	Pogona vitticeps	Squamata	Lizard	USA	America	CB	https://reptile-database.reptarium.cz/species?genus=Pogona&species=vitticeps&search_param=%28%28common_name%3D%27Pogona+vitticeps%27%29%29
27	40	Central bearded dragon	Pogona vitticeps	Squamata	Lizard	USA	America	CB	https://reptile-database.reptarium.cz/species?genus=Pogona&species=vitticeps&search_param=%28%28common_name%3D%27Pogona+vitticeps%27%29%29
27	40	Central bearded dragon	Pogona vitticeps	Squamata	Lizard	USA	America	CB	https://reptile-database.reptarium.cz/species?genus=Pogona&species=vitticeps&search_param=%28%28common_name%3D%27Pogona+vitticeps%27%29%29
27	41	New Caledonia giant gecko	Rhacodactylus leachianus	Squamata	Lizard	USA	America	CB	https://reptile-database.reptarium.cz/species?genus=Rhacodactylus&species=leachianus&search_param=%28%28common_name%3D%27Rhacodactylus+leachianus%27%29%29
27	41	New Caledonia giant gecko	Rhacodactylus leachianus	Squamata	Lizard	USA	America	CB	https://reptile-database.reptarium.cz/species?genus=Rhacodactylus&species=leachianus&search_param=%28%28common_name%3D%27Rhacodactylus+leachianus%27%29%29
27	41	New Caledonia giant gecko	Rhacodactylus leachianus	Squamata	Lizard	USA	America	CB	https://reptile-database.reptarium.cz/species?genus=Rhacodactylus&species=leachianus&search_param=%28%28common_name%3D%27Rhacodactylus+leachianus%27%29%29
27	41	New Caledonia giant gecko	Rhacodactylus leachianus	Squamata	Lizard	USA	America	CB	https://reptile-database.reptarium.cz/species?genus=Rhacodactylus&species=leachianus&search_param=%28%28common_name%3D%27Rhacodactylus+leachianus%27%29%29
27	41	New Caledonia giant gecko	Rhacodactylus leachianus	Squamata	Lizard	USA	America	CB	https://reptile-database.reptarium.cz/species?genus=Rhacodactylus&species=leachianus&search_param=%28%28common_name%3D%27Rhacodactylus+leachianus%27%29%29
27	42	Common leopard gecko	Eublepharis macularius	Squamata	Lizard	USA	America	CB	https://reptile-database.reptarium.cz/species?genus=Eublepharis&species=macularius&search_param=%28%28common_name%3D%27Eublepharis+macularius%27%29%29
27	42	Common leopard gecko	Eublepharis macularius	Squamata	Lizard	USA	America	CB	https://reptile-database.reptarium.cz/species?genus=Eublepharis&species=macularius&search_param=%28%28common_name%3D%27Eublepharis+macularius%27%29%29
27	42	Common leopard gecko	Eublepharis macularius	Squamata	Lizard	USA	America	CB	https://reptile-database.reptarium.cz/species?genus=Eublepharis&species=macularius&search_param=%28%28common_name%3D%27Eublepharis+macularius%27%29%29

Table S1: Animal species sampled in this study - country origin and categorization as captive bred (CB), farm bred (FB) and wild-caught (WC)

Shipment No.	Sample batch	Animal species	Scientific name of animal/s	Order	Suborder	Country origin	Continent	Captive bred (CB), farm bred (FB), wild-caught (WC)	Web URL used for assigning animal species to categories CB, FB and WC
27	42	Common leopard gecko	Eublepharis macularius	Squamata	Lizard	USA	America	CB	https://reptile-database.reptarium.cz/species?genus=Eublepharis&species=macularius&search_param=%28%28common_name%3D%27Eublepharis+macularius%27%29%29
27	42	Common leopard gecko	Eublepharis macularius	Squamata	Lizard	USA	America	CB	https://reptile-database.reptarium.cz/species?genus=Eublepharis&species=macularius&search_param=%28%28common_name%3D%27Eublepharis+macularius%27%29%29
27	42	Common leopard gecko	Eublepharis macularius	Squamata	Lizard	USA	America	CB	https://reptile-database.reptarium.cz/species?genus=Eublepharis&species=macularius&search_param=%28%28common_name%3D%27Eublepharis+macularius%27%29%29
27	42	Common leopard gecko	Eublepharis macularius	Squamata	Lizard	USA	America	CB	https://reptile-database.reptarium.cz/species?genus=Eublepharis&species=macularius&search_param=%28%28common_name%3D%27Eublepharis+macularius%27%29%29
27	42	Common leopard gecko	Eublepharis macularius	Squamata	Lizard	USA	America	CB	https://reptile-database.reptarium.cz/species?genus=Eublepharis&species=macularius&search_param=%28%28common_name%3D%27Eublepharis+macularius%27%29%29
27	42	Common leopard gecko	Eublepharis macularius	Squamata	Lizard	USA	America	CB	https://reptile-database.reptarium.cz/species?genus=Eublepharis&species=macularius&search_param=%28%28common_name%3D%27Eublepharis+macularius%27%29%29
27	42	Common leopard gecko	Eublepharis macularius	Squamata	Lizard	USA	America	CB	https://reptile-database.reptarium.cz/species?genus=Eublepharis&species=macularius&search_param=%28%28common_name%3D%27Eublepharis+macularius%27%29%29
27	42	Common leopard gecko	Eublepharis macularius	Squamata	Lizard	USA	America	CB	https://reptile-database.reptarium.cz/species?genus=Eublepharis&species=macularius&search_param=%28%28common_name%3D%27Eublepharis+macularius%27%29%29
27	42	Common leopard gecko	Eublepharis macularius	Squamata	Lizard	USA	America	CB	https://reptile-database.reptarium.cz/species?genus=Eublepharis&species=macularius&search_param=%28%28common_name%3D%27Eublepharis+macularius%27%29%29
27	42	Common leopard gecko	Eublepharis macularius	Squamata	Lizard	USA	America	CB	https://reptile-database.reptarium.cz/species?genus=Eublepharis&species=macularius&search_param=%28%28common_name%3D%27Eublepharis+macularius%27%29%29
28	43	Fat-tail gecko	Hemitheconyx caudicinctus	Squamata	Lizard	USA	America	CB	https://reptile-database.reptarium.cz/species?genus=Hemitheconyx&species=caudicinctus&search_param=%28%28common_name%3D%27Hemitheconyx+caudicinctus%27%29%29
28	43	Fat-tail gecko	Hemitheconyx caudicinctus	Squamata	Lizard	USA	America	CB	https://reptile-database.reptarium.cz/species?genus=Hemitheconyx&species=caudicinctus&search_param=%28%28common_name%3D%27Hemitheconyx+caudicinctus%27%29%29

Table S1: Animal species sampled in this study - country origin and categorization as captive bred (CB), farm bred (FB) and wild-caught (WC)

Shipment No.	Sample batch	Animal species	Scientific name of animal/s	Order	Suborder	Country origin	Continent	Captive bred (CB), farm bred (FB), wild-caught (WC)	Web URL used for assigning animal species to categories CB, FB and WC
28	43	Fat-tail gecko	Hemitheconyx caudicinctus	Squamata	Lizard	USA	America	CB	https://reptile-database.reptarium.cz/species?genus=Hemitheconyx&species=caudicinctus&search_param=%28%28common_name%3D%27Hemitheconyx+caudicinctus%27%29%29
28	43	Fat-tail gecko	Hemitheconyx caudicinctus	Squamata	Lizard	USA	America	CB	https://reptile-database.reptarium.cz/species?genus=Hemitheconyx&species=caudicinctus&search_param=%28%28common_name%3D%27Hemitheconyx+caudicinctus%27%29%29
28	43	Fat-tail gecko	Hemitheconyx caudicinctus	Squamata	Lizard	USA	America	CB	https://reptile-database.reptarium.cz/species?genus=Hemitheconyx&species=caudicinctus&search_param=%28%28common_name%3D%27Hemitheconyx+caudicinctus%27%29%29
28	43	Fat-tail gecko	Hemitheconyx caudicinctus	Squamata	Lizard	USA	America	CB	https://reptile-database.reptarium.cz/species?genus=Hemitheconyx&species=caudicinctus&search_param=%28%28common_name%3D%27Hemitheconyx+caudicinctus%27%29%29
28	43	Fat-tail gecko	Hemitheconyx caudicinctus	Squamata	Lizard	USA	America	CB	https://reptile-database.reptarium.cz/species?genus=Hemitheconyx&species=caudicinctus&search_param=%28%28common_name%3D%27Hemitheconyx+caudicinctus%27%29%29
28	44	Crested gecko	Correlophus ciliatus	Squamata	Lizard	USA	America	CB	https://reptile-database.reptarium.cz/species?genus=Correlophus&species=ciliatus&search_param=%28%28common_name%3D%27Correlophus+ciliatus%27%29%29
28	44	Crested gecko	Correlophus ciliatus	Squamata	Lizard	USA	America	CB	https://reptile-database.reptarium.cz/species?genus=Correlophus&species=ciliatus&search_param=%28%28common_name%3D%27Correlophus+ciliatus%27%29%29
28	44	Crested gecko	Correlophus ciliatus	Squamata	Lizard	USA	America	CB	https://reptile-database.reptarium.cz/species?genus=Correlophus&species=ciliatus&search_param=%28%28common_name%3D%27Correlophus+ciliatus%27%29%29
28	44	Crested gecko	Correlophus ciliatus	Squamata	Lizard	USA	America	CB	https://reptile-database.reptarium.cz/species?genus=Correlophus&species=ciliatus&search_param=%28%28common_name%3D%27Correlophus+ciliatus%27%29%29
28	44	Crested gecko	Correlophus ciliatus	Squamata	Lizard	USA	America	CB	https://reptile-database.reptarium.cz/species?genus=Correlophus&species=ciliatus&search_param=%28%28common_name%3D%27Correlophus+ciliatus%27%29%29
28	43	Fat-tail gecko	Hemitheconyx caudicinctus	Squamata	Lizard	USA	America	CB	https://reptile-database.reptarium.cz/species?genus=Hemitheconyx&species=caudicinctus&search_param=%28%28common_name%3D%27Hemitheconyx+caudicinctus%27%29%29
28	43	Fat-tail gecko	Hemitheconyx caudicinctus	Squamata	Lizard	USA	America	CB	https://reptile-database.reptarium.cz/species?genus=Hemitheconyx&species=caudicinctus&search_param=%28%28common_name%3D%27Hemitheconyx+caudicinctus%27%29%29

Table S1: Animal species sampled in this study - country origin and categorization as captive bred (CB), farm bred (FB) and wild-caught (WC)

Shipment No.	Sample batch	Animal species	Scientific name of animal/s	Order	Suborder	Country origin	Continent	Captive bred (CB), farm bred (FB), wild-caught (WC)	Web URL used for assigning animal species to categories CB, FB and WC
28	43	Fat-tail gecko	Hemitheconyx caudicinctus	Squamata	Lizard	USA	America	CB	https://reptile-database.reptarium.cz/species?genus=Hemitheconyx&species=caudicinctus&search_param=%28%28common_name%3D%27Hemitheconyx+caudicinctus%27%29%29
28	43	Fat-tail gecko	Hemitheconyx caudicinctus	Squamata	Lizard	USA	America	CB	https://reptile-database.reptarium.cz/species?genus=Hemitheconyx&species=caudicinctus&search_param=%28%28common_name%3D%27Hemitheconyx+caudicinctus%27%29%29
28	43	Fat-tail gecko	Hemitheconyx caudicinctus	Squamata	Lizard	USA	America	CB	https://reptile-database.reptarium.cz/species?genus=Hemitheconyx&species=caudicinctus&search_param=%28%28common_name%3D%27Hemitheconyx+caudicinctus%27%29%29
28	43	Fat-tail gecko	Hemitheconyx caudicinctus	Squamata	Lizard	USA	America	CB	https://reptile-database.reptarium.cz/species?genus=Hemitheconyx&species=caudicinctus&search_param=%28%28common_name%3D%27Hemitheconyx+caudicinctus%27%29%29
28	44	Crested gecko	Correlophus ciliatus	Squamata	Lizard	USA	America	CB	https://reptile-database.reptarium.cz/species?genus=Correlophus&species=ciliatus&search_param=%28%28common_name%3D%27Correlophus+ciliatus%27%29%29
28	44	Crested gecko	Correlophus ciliatus	Squamata	Lizard	USA	America	CB	https://reptile-database.reptarium.cz/species?genus=Correlophus&species=ciliatus&search_param=%28%28common_name%3D%27Correlophus+ciliatus%27%29%29
28	44	Crested gecko	Correlophus ciliatus	Squamata	Lizard	USA	America	CB	https://reptile-database.reptarium.cz/species?genus=Correlophus&species=ciliatus&search_param=%28%28common_name%3D%27Correlophus+ciliatus%27%29%29
28	44	Crested gecko	Correlophus ciliatus	Squamata	Lizard	USA	America	CB	https://reptile-database.reptarium.cz/species?genus=Correlophus&species=ciliatus&search_param=%28%28common_name%3D%27Correlophus+ciliatus%27%29%29
28	44	Crested gecko	Correlophus ciliatus	Squamata	Lizard	USA	America	CB	https://reptile-database.reptarium.cz/species?genus=Correlophus&species=ciliatus&search_param=%28%28common_name%3D%27Correlophus+ciliatus%27%29%29
28	44	Crested gecko	Correlophus ciliatus	Squamata	Lizard	USA	America	CB	https://reptile-database.reptarium.cz/species?genus=Correlophus&species=ciliatus&search_param=%28%28common_name%3D%27Correlophus+ciliatus%27%29%29
28	44	Crested gecko	Correlophus ciliatus	Squamata	Lizard	USA	America	CB	https://reptile-database.reptarium.cz/species?genus=Correlophus&species=ciliatus&search_param=%28%28common_name%3D%27Correlophus+ciliatus%27%29%29
28	45	Western hognose snake	Heterodon nasicus	Squamata	Snake	USA	America	CB	https://reptile-database.reptarium.cz/species?genus=Heterodon&species=nasicus&search_param=%28%28common_name%3D%27Heterodon+nasicus%27%29%29

Table S1: Animal species sampled in this study - country origin and categorization as captive bred (CB), farm bred (FB) and wild-caught (WC)

Shipment No.	Sample batch	Animal species	Scientific name of animal/s	Order	Suborder	Country origin	Continent	Captive bred (CB), farm bred (FB), wild-caught (WC)	Web URL used for assigning animal species to categories CB, FB and WC
28	45	Western hognose snake	Heterodon nasicus	Squamata	Snake	USA	America	CB	https://reptile-database.reptarium.cz/species?genus=Heterodon&species=nasicus&search_param=%28%28common_name%3D%27Heterodon+nasicus%27%29%29
28	45	Western hognose snake	Heterodon nasicus	Squamata	Snake	USA	America	CB	https://reptile-database.reptarium.cz/species?genus=Heterodon&species=nasicus&search_param=%28%28common_name%3D%27Heterodon+nasicus%27%29%29
28	45	Western hognose snake	Heterodon nasicus	Squamata	Snake	USA	America	CB	https://reptile-database.reptarium.cz/species?genus=Heterodon&species=nasicus&search_param=%28%28common_name%3D%27Heterodon+nasicus%27%29%29
28	45	Western hognose snake	Heterodon nasicus	Squamata	Snake	USA	America	CB	https://reptile-database.reptarium.cz/species?genus=Heterodon&species=nasicus&search_param=%28%28common_name%3D%27Heterodon+nasicus%27%29%29
28	45	Western hognose snake	Heterodon nasicus	Squamata	Snake	USA	America	CB	https://reptile-database.reptarium.cz/species?genus=Heterodon&species=nasicus&search_param=%28%28common_name%3D%27Heterodon+nasicus%27%29%29
29	46	Red cornsnake	Pantherophis guttatus	Squamata	Snake	USA	America	CB	https://reptile-database.reptarium.cz/species?genus=Pantherophis&species=guttatus&search_param=%28%28common_name%3D%27Pantherophis+guttatus%27%29%29
29	46	Red cornsnake	Pantherophis guttatus	Squamata	Snake	USA	America	CB	https://reptile-database.reptarium.cz/species?genus=Pantherophis&species=guttatus&search_param=%28%28common_name%3D%27Pantherophis+guttatus%27%29%29
29	46	Red cornsnake	Pantherophis guttatus	Squamata	Snake	USA	America	CB	https://reptile-database.reptarium.cz/species?genus=Pantherophis&species=guttatus&search_param=%28%28common_name%3D%27Pantherophis+guttatus%27%29%29
29	46	Red cornsnake	Pantherophis guttatus	Squamata	Snake	USA	America	CB	https://reptile-database.reptarium.cz/species?genus=Pantherophis&species=guttatus&search_param=%28%28common_name%3D%27Pantherophis+guttatus%27%29%29
29	46	Red cornsnake	Pantherophis guttatus	Squamata	Snake	USA	America	CB	https://reptile-database.reptarium.cz/species?genus=Pantherophis&species=guttatus&search_param=%28%28common_name%3D%27Pantherophis+guttatus%27%29%29
29	46	Red cornsnake	Pantherophis guttatus	Squamata	Snake	USA	America	CB	https://reptile-database.reptarium.cz/species?genus=Pantherophis&species=guttatus&search_param=%28%28common_name%3D%27Pantherophis+guttatus%27%29%29
29	46	Red cornsnake	Pantherophis guttatus	Squamata	Snake	USA	America	CB	https://reptile-database.reptarium.cz/species?genus=Pantherophis&species=guttatus&search_param=%28%28common_name%3D%27Pantherophis+guttatus%27%29%29

Table S1: Animal species sampled in this study - country origin and categorization as captive bred (CB), farm bred (FB) and wild-caught (WC)

Shipment No.	Sample batch	Animal species	Scientific name of animal/s	Order	Suborder	Country origin	Continent	Captive bred (CB), farm bred (FB), wild-caught (WC)	Web URL used for assigning animal species to categories CB, FB and WC
29	47	Ball python	Python regius	Squamata	Snake	USA	America	CB	https://reptile-database.reptarium.cz/species?genus=Python&species=regius&search_param=%28%28common_name%3D%27Python+regius%27%29%29
29	47	Ball python	Python regius	Squamata	Snake	USA	America	CB	https://reptile-database.reptarium.cz/species?genus=Python&species=regius&search_param=%28%28common_name%3D%27Python+regius%27%29%29
29	47	Ball python	Python regius	Squamata	Snake	USA	America	CB	https://reptile-database.reptarium.cz/species?genus=Python&species=regius&search_param=%28%28common_name%3D%27Python+regius%27%29%29
29	47	Ball python	Python regius	Squamata	Snake	USA	America	CB	https://reptile-database.reptarium.cz/species?genus=Python&species=regius&search_param=%28%28common_name%3D%27Python+regius%27%29%29
29	47	Ball python	Python regius	Squamata	Snake	USA	America	CB	https://reptile-database.reptarium.cz/species?genus=Python&species=regius&search_param=%28%28common_name%3D%27Python+regius%27%29%29
29	47	Ball python	Python regius	Squamata	Snake	USA	America	CB	https://reptile-database.reptarium.cz/species?genus=Python&species=regius&search_param=%28%28common_name%3D%27Python+regius%27%29%29
29	47	Ball python	Python regius	Squamata	Snake	USA	America	CB	https://reptile-database.reptarium.cz/species?genus=Python&species=regius&search_param=%28%28common_name%3D%27Python+regius%27%29%29
29	47	Ball python	Python regius	Squamata	Snake	USA	America	CB	https://reptile-database.reptarium.cz/species?genus=Python&species=regius&search_param=%28%28common_name%3D%27Python+regius%27%29%29
29	47	Ball python	Python regius	Squamata	Snake	USA	America	CB	https://reptile-database.reptarium.cz/species?genus=Python&species=regius&search_param=%28%28common_name%3D%27Python+regius%27%29%29
29	47	Ball python	Python regius	Squamata	Snake	USA	America	CB	https://reptile-database.reptarium.cz/species?genus=Python&species=regius&search_param=%28%28common_name%3D%27Python+regius%27%29%29
30	pre-trial	chameleon	Chamealeo	Squamata	Lizard	Mozambique	Africa	WC	
31	48	Stripeneck musk turtle	Sternotherus peltifer	Testudines	Turtle	USA	America	FB	https://reptile-database.reptarium.cz/species?genus=Sternotherus&species=peltifer&search_param=%28%28common_name%3D%27Sternotherus+peltifer%27%29%29
31	48	Stripeneck musk turtle	Sternotherus peltifer	Testudines	Turtle	USA	America	FB	https://reptile-database.reptarium.cz/species?genus=Sternotherus&species=peltifer&search_param=%28%28common_name%3D%27Sternotherus+peltifer%27%29%29

Table S1: Animal species sampled in this study - country origin and categorization as captive bred (CB), farm bred (FB) and wild-caught (WC)

Shipment No.	Sample batch	Animal species	Scientific name of animal/s	Order	Suborder	Country origin	Continent	Captive bred (CB), farm bred (FB), wild-caught (WC)	Web URL used for assigning animal species to categories CB, FB and WC
31	48	Stripeneck musk turtle	Sternotherus peltifer	Testudines	Turtle	USA	America	FB	https://reptile-database.reptarium.cz/species?genus=Sternotherus&species=peltifer&search_param=%28%28common_name%3D%27Sternotherus+peltifer%27%29%29
31	48	Stripeneck musk turtle	Sternotherus peltifer	Testudines	Turtle	USA	America	FB	https://reptile-database.reptarium.cz/species?genus=Sternotherus&species=peltifer&search_param=%28%28common_name%3D%27Sternotherus+peltifer%27%29%29
31	48	Stripeneck musk turtle	Sternotherus peltifer	Testudines	Turtle	USA	America	FB	https://reptile-database.reptarium.cz/species?genus=Sternotherus&species=peltifer&search_param=%28%28common_name%3D%27Sternotherus+peltifer%27%29%29
31	48	Stripeneck musk turtle	Sternotherus peltifer	Testudines	Turtle	USA	America	FB	https://reptile-database.reptarium.cz/species?genus=Sternotherus&species=peltifer&search_param=%28%28common_name%3D%27Sternotherus+peltifer%27%29%29
31	48	Stripeneck musk turtle	Sternotherus peltifer	Testudines	Turtle	USA	America	FB	https://reptile-database.reptarium.cz/species?genus=Sternotherus&species=peltifer&search_param=%28%28common_name%3D%27Sternotherus+peltifer%27%29%29
31	48	Stripeneck musk turtle	Sternotherus peltifer	Testudines	Turtle	USA	America	FB	https://reptile-database.reptarium.cz/species?genus=Sternotherus&species=peltifer&search_param=%28%28common_name%3D%27Sternotherus+peltifer%27%29%29
32	50	Horsefield's tortoise	Testudo horsfieldii	Testudines	Turtle	Uzbekistan	Asia	FB	https://reptile-database.reptarium.cz/species?genus=Testudo&species=horsfieldii&search_param=%28%28common_name%3D%27Testudo+horsfieldii%27%29%29
32	50	Horsefield's tortoise	Testudo horsfieldii	Testudines	Turtle	Uzbekistan	Asia	FB	https://reptile-database.reptarium.cz/species?genus=Testudo&species=horsfieldii&search_param=%28%28common_name%3D%27Testudo+horsfieldii%27%29%29
32	50	Horsefield's tortoise	Testudo horsfieldii	Testudines	Turtle	Uzbekistan	Asia	FB	https://reptile-database.reptarium.cz/species?genus=Testudo&species=horsfieldii&search_param=%28%28common_name%3D%27Testudo+horsfieldii%27%29%29
32	50	Horsefield's tortoise	Testudo horsfieldii	Testudines	Turtle	Uzbekistan	Asia	FB	https://reptile-database.reptarium.cz/species?genus=Testudo&species=horsfieldii&search_param=%28%28common_name%3D%27Testudo+horsfieldii%27%29%29
32	50	Horsefield's tortoise	Testudo horsfieldii	Testudines	Turtle	Uzbekistan	Asia	FB	https://reptile-database.reptarium.cz/species?genus=Testudo&species=horsfieldii&search_param=%28%28common_name%3D%27Testudo+horsfieldii%27%29%29
32	50	Horsefield's tortoise	Testudo horsfieldii	Testudines	Turtle	Uzbekistan	Asia	FB	https://reptile-database.reptarium.cz/species?genus=Testudo&species=horsfieldii&search_param=%28%28common_name%3D%27Testudo+horsfieldii%27%29%29

Table S1: Animal species sampled in this study - country origin and categorization as captive bred (CB), farm bred (FB) and wild-caught (WC)

Shipment No.	Sample batch	Animal species	Scientific name of animal/s	Order	Suborder	Country origin	Continent	Captive bred (CB), farm bred (FB), wild-caught (WC)	Web URL used for assigning animal species to categories CB, FB and WC
32	50	Horsefield's tortoise	Testudo horsfieldii	Testudines	Turtle	Uzbekistan	Asia	FB	https://reptile-database.reptarium.cz/species?genus=Testudo&species=horsfieldii&search_param=%28%28common_name%3D%27Testudo+horsfieldii%27%29%29
32	50	Horsefield's tortoise	Testudo horsfieldii	Testudines	Turtle	Uzbekistan	Asia	FB	https://reptile-database.reptarium.cz/species?genus=Testudo&species=horsfieldii&search_param=%28%28common_name%3D%27Testudo+horsfieldii%27%29%29
32	50	Horsefield's tortoise	Testudo horsfieldii	Testudines	Turtle	Uzbekistan	Asia	FB	https://reptile-database.reptarium.cz/species?genus=Testudo&species=horsfieldii&search_param=%28%28common_name%3D%27Testudo+horsfieldii%27%29%29
32	50	Horsefield's tortoise	Testudo horsfieldii	Testudines	Turtle	Uzbekistan	Asia	FB	https://reptile-database.reptarium.cz/species?genus=Testudo&species=horsfieldii&search_param=%28%28common_name%3D%27Testudo+horsfieldii%27%29%29
32	50	Horsefield's tortoise	Testudo horsfieldii	Testudines	Turtle	Uzbekistan	Asia	FB	https://reptile-database.reptarium.cz/species?genus=Testudo&species=horsfieldii&search_param=%28%28common_name%3D%27Testudo+horsfieldii%27%29%29
32	49	Horsefield's tortoise	Testudo horsfieldii	Testudines	Turtle	Uzbekistan	Asia	FB	https://reptile-database.reptarium.cz/species?genus=Testudo&species=horsfieldii&search_param=%28%28common_name%3D%27Testudo+horsfieldii%27%29%29
32	49	Horsefield's tortoise	Testudo horsfieldii	Testudines	Turtle	Uzbekistan	Asia	FB	https://reptile-database.reptarium.cz/species?genus=Testudo&species=horsfieldii&search_param=%28%28common_name%3D%27Testudo+horsfieldii%27%29%29
32	49	Horsefield's tortoise	Testudo horsfieldii	Testudines	Turtle	Uzbekistan	Asia	FB	https://reptile-database.reptarium.cz/species?genus=Testudo&species=horsfieldii&search_param=%28%28common_name%3D%27Testudo+horsfieldii%27%29%29
32	49	Horsefield's tortoise	Testudo horsfieldii	Testudines	Turtle	Uzbekistan	Asia	FB	https://reptile-database.reptarium.cz/species?genus=Testudo&species=horsfieldii&search_param=%28%28common_name%3D%27Testudo+horsfieldii%27%29%29
32	49	Horsefield's tortoise	Testudo horsfieldii	Testudines	Turtle	Uzbekistan	Asia	FB	https://reptile-database.reptarium.cz/species?genus=Testudo&species=horsfieldii&search_param=%28%28common_name%3D%27Testudo+horsfieldii%27%29%29
32	49	Horsefield's tortoise	Testudo horsfieldii	Testudines	Turtle	Uzbekistan	Asia	FB	https://reptile-database.reptarium.cz/species?genus=Testudo&species=horsfieldii&search_param=%28%28common_name%3D%27Testudo+horsfieldii%27%29%29
32	49	Horsefield's tortoise	Testudo horsfieldii	Testudines	Turtle	Uzbekistan	Asia	FB	https://reptile-database.reptarium.cz/species?genus=Testudo&species=horsfieldii&search_param=%28%28common_name%3D%27Testudo+horsfieldii%27%29%29

Table S1: Animal species sampled in this study - country origin and categorization as captive bred (CB), farm bred (FB) and wild-caught (WC)

Shipment No.	Sample batch	Animal species	Scientific name of animal/s	Order	Suborder	Country origin	Continent	Captive bred (CB), farm bred (FB), wild-caught (WC)	Web URL used for assigning animal species to categories CB, FB and WC
32	49	Horsefield's tortoise	Testudo horsfieldii	Testudines	Turtle	Uzbekistan	Asia	FB	https://reptile-database.reptarium.cz/species?genus=Testudo&species=horsfieldii&search_param=%28%28common_name%3D%27Testudo+horsfieldii%27%29%29
33	50	Ball python	Python regius	Squamata	Snake	Canada	America	CB	https://reptile-database.reptarium.cz/species?genus=Python&species=regius&search_param=%28%28common_name%3D%27Python+regius%27%29%29
33	50	Ball python	Python regius	Squamata	Snake	Canada	America	CB	https://reptile-database.reptarium.cz/species?genus=Python&species=regius&search_param=%28%28common_name%3D%27Python+regius%27%29%29
33	50	Ball python	Python regius	Squamata	Snake	Canada	America	CB	https://reptile-database.reptarium.cz/species?genus=Python&species=regius&search_param=%28%28common_name%3D%27Python+regius%27%29%29
33	50	Ball python	Python regius	Squamata	Snake	Canada	America	CB	https://reptile-database.reptarium.cz/species?genus=Python&species=regius&search_param=%28%28common_name%3D%27Python+regius%27%29%29
33	50	Ball python	Python regius	Squamata	Snake	Canada	America	CB	https://reptile-database.reptarium.cz/species?genus=Python&species=regius&search_param=%28%28common_name%3D%27Python+regius%27%29%29
33	50	Ball python	Python regius	Squamata	Snake	Canada	America	CB	https://reptile-database.reptarium.cz/species?genus=Python&species=regius&search_param=%28%28common_name%3D%27Python+regius%27%29%29
33	50	Ball python	Python regius	Squamata	Snake	Canada	America	CB	https://reptile-database.reptarium.cz/species?genus=Python&species=regius&search_param=%28%28common_name%3D%27Python+regius%27%29%29
33	50	Ball python	Python regius	Squamata	Snake	Canada	America	CB	https://reptile-database.reptarium.cz/species?genus=Python&species=regius&search_param=%28%28common_name%3D%27Python+regius%27%29%29
33	50	Ball python	Python regius	Squamata	Snake	Canada	America	CB	https://reptile-database.reptarium.cz/species?genus=Python&species=regius&search_param=%28%28common_name%3D%27Python+regius%27%29%29
33	50	Ball python	Python regius	Squamata	Snake	Canada	America	CB	https://reptile-database.reptarium.cz/species?genus=Python&species=regius&search_param=%28%28common_name%3D%27Python+regius%27%29%29
33	50	Ball python	Python regius	Squamata	Snake	Canada	America	CB	https://reptile-database.reptarium.cz/species?genus=Python&species=regius&search_param=%28%28common_name%3D%27Python+regius%27%29%29

Table S1: Animal species sampled in this study - country origin and categorization as captive bred (CB), farm bred (FB) and wild-caught (WC)

Shipment No.	Sample batch	Animal species	Scientific name of animal/s	Order	Suborder	Country origin	Continent	Captive bred (CB), farm bred (FB), wild-caught (WC)	Web URL used for assigning animal species to categories CB, FB and WC
33	50	Ball python	Python regius	Squamata	Snake	Canada	America	CB	https://reptile-database.reptarium.cz/species?genus=Python&species=regius&search_param=%28%28common_name%3D%27Python+regius%27%29%29
33	50	Ball python	Python regius	Squamata	Snake	Canada	America	CB	https://reptile-database.reptarium.cz/species?genus=Python&species=regius&search_param=%28%28common_name%3D%27Python+regius%27%29%29
33	50	Ball python	Python regius	Squamata	Snake	Canada	America	CB	https://reptile-database.reptarium.cz/species?genus=Python&species=regius&search_param=%28%28common_name%3D%27Python+regius%27%29%29
33	50	Ball python	Python regius	Squamata	Snake	Canada	America	CB	https://reptile-database.reptarium.cz/species?genus=Python&species=regius&search_param=%28%28common_name%3D%27Python+regius%27%29%29
33	50	Ball python	Python regius	Squamata	Snake	Canada	America	CB	https://reptile-database.reptarium.cz/species?genus=Python&species=regius&search_param=%28%28common_name%3D%27Python+regius%27%29%29
33	50	Ball python	Python regius	Squamata	Snake	Canada	America	CB	https://reptile-database.reptarium.cz/species?genus=Python&species=regius&search_param=%28%28common_name%3D%27Python+regius%27%29%29
33	50	Ball python	Python regius	Squamata	Snake	Canada	America	CB	https://reptile-database.reptarium.cz/species?genus=Python&species=regius&search_param=%28%28common_name%3D%27Python+regius%27%29%29
34	51	Ameiva	Ameiva sp.	Squamata	Lizard	Guyana	America	WC	https://reptile-database.reptarium.cz/advanced_search?common_name=ameiva&submit=Search
34	51	Ameiva	Ameiva sp.	Squamata	Lizard	Guyana	America	WC	https://reptile-database.reptarium.cz/advanced_search?common_name=ameiva&submit=Search
34	51	Ameiva	Ameiva sp.	Squamata	Lizard	Guyana	America	WC	https://reptile-database.reptarium.cz/advanced_search?common_name=ameiva&submit=Search
34	51	Ameiva	Ameiva sp.	Squamata	Lizard	Guyana	America	WC	https://reptile-database.reptarium.cz/advanced_search?common_name=ameiva&submit=Search
34	51	Ameiva	Ameiva sp.	Squamata	Lizard	Guyana	America	WC	https://reptile-database.reptarium.cz/advanced_search?common_name=ameiva&submit=Search
34	51	Ameiva	Ameiva sp.	Squamata	Lizard	Guyana	America	WC	https://reptile-database.reptarium.cz/advanced_search?common_name=ameiva&submit=Search
34	51	Ameiva	Ameiva sp.	Squamata	Lizard	Guyana	America	WC	https://reptile-database.reptarium.cz/advanced_search?common_name=ameiva&submit=Search

Table S1: Animal species sampled in this study - country origin and categorization as captive bred (CB), farm bred (FB) and wild-caught (WC)

Shipment No.	Sample batch	Animal species	Scientific name of animal/s	Order	Suborder	Country origin	Continent	Captive bred (CB), farm bred (FB), wild-caught (WC)	Web URL used for assigning animal species to categories CB, FB and WC
34	51	Ameiva	Ameiva sp.	Squamata	Lizard	Guyana	America	WC	https://reptile-database.reptarium.cz/advanced_search?common_name=ameiva&submit=Search
35	52	Red-footed tortoise	Chelonoidis carbonarius	Testudines	Turtle	Colombia	America	WC/FB	https://reptile-database.reptarium.cz/species?genus=Chelonoidis&species=carbonarius&search_param=%28%28common_name%3D%27Chelonoidis+carbonarius%27%29%29
35	52	Red-footed tortoise	Chelonoidis carbonarius	Testudines	Turtle	Colombia	America	WC/FB	https://reptile-database.reptarium.cz/species?genus=Chelonoidis&species=carbonarius&search_param=%28%28common_name%3D%27Chelonoidis+carbonarius%27%29%29
35	52	Red-footed tortoise	Chelonoidis carbonarius	Testudines	Turtle	Colombia	America	WC/FB	https://reptile-database.reptarium.cz/species?genus=Chelonoidis&species=carbonarius&search_param=%28%28common_name%3D%27Chelonoidis+carbonarius%27%29%29
35	52	Red-footed tortoise	Chelonoidis carbonarius	Testudines	Turtle	Colombia	America	WC/FB	https://reptile-database.reptarium.cz/species?genus=Chelonoidis&species=carbonarius&search_param=%28%28common_name%3D%27Chelonoidis+carbonarius%27%29%29
35	52	Red-footed tortoise	Chelonoidis carbonarius	Testudines	Turtle	Colombia	America	WC/FB	https://reptile-database.reptarium.cz/species?genus=Chelonoidis&species=carbonarius&search_param=%28%28common_name%3D%27Chelonoidis+carbonarius%27%29%29
35	52	Red-footed tortoise	Chelonoidis carbonarius	Testudines	Turtle	Colombia	America	WC/FB	https://reptile-database.reptarium.cz/species?genus=Chelonoidis&species=carbonarius&search_param=%28%28common_name%3D%27Chelonoidis+carbonarius%27%29%29
35	53	Red-footed tortoise	Chelonoidis carbonarius	Testudines	Turtle	Colombia	America	WC/FB	https://reptile-database.reptarium.cz/species?genus=Chelonoidis&species=carbonarius&search_param=%28%28common_name%3D%27Chelonoidis+carbonarius%27%29%29
35	53	Red-footed tortoise	Chelonoidis carbonarius	Testudines	Turtle	Colombia	America	WC/FB	https://reptile-database.reptarium.cz/species?genus=Chelonoidis&species=carbonarius&search_param=%28%28common_name%3D%27Chelonoidis+carbonarius%27%29%29
35	53	Red-footed tortoise	Chelonoidis carbonarius	Testudines	Turtle	Colombia	America	WC/FB	https://reptile-database.reptarium.cz/species?genus=Chelonoidis&species=carbonarius&search_param=%28%28common_name%3D%27Chelonoidis+carbonarius%27%29%29
35	53	Red-footed tortoise	Chelonoidis carbonarius	Testudines	Turtle	Colombia	America	WC/FB	https://reptile-database.reptarium.cz/species?genus=Chelonoidis&species=carbonarius&search_param=%28%28common_name%3D%27Chelonoidis+carbonarius%27%29%29
35	53	Red-footed tortoise	Chelonoidis carbonarius	Testudines	Turtle	Colombia	America	WC/FB	https://reptile-database.reptarium.cz/species?genus=Chelonoidis&species=carbonarius&search_param=%28%28common_name%3D%27Chelonoidis+carbonarius%27%29%29

Table S1: Animal species sampled in this study - country origin and categorization as captive bred (CB), farm bred (FB) and wild-caught (WC)

Shipment No.	Sample batch	Animal species	Scientific name of animal/s	Order	Suborder	Country origin	Continent	Captive bred (CB), farm bred (FB), wild-caught (WC)	Web URL used for assigning animal species to categories CB, FB and WC
35	52	Red-footed tortoise	Chelonoidis carbonarius	Testudines	Turtle	Colombia	America	WC/FB	https://reptile-database.reptarium.cz/species?genus=Chelonoidis&species=carbonarius&search_param=%28%28common_name%3D%27Chelonoidis+carbonarius%27%29%29
35	52	Red-footed tortoise	Chelonoidis carbonarius	Testudines	Turtle	Colombia	America	WC/FB	https://reptile-database.reptarium.cz/species?genus=Chelonoidis&species=carbonarius&search_param=%28%28common_name%3D%27Chelonoidis+carbonarius%27%29%29
35	52	Red-footed tortoise	Chelonoidis carbonarius	Testudines	Turtle	Colombia	America	WC/FB	https://reptile-database.reptarium.cz/species?genus=Chelonoidis&species=carbonarius&search_param=%28%28common_name%3D%27Chelonoidis+carbonarius%27%29%29
35	52	Red-footed tortoise	Chelonoidis carbonarius	Testudines	Turtle	Colombia	America	WC/FB	https://reptile-database.reptarium.cz/species?genus=Chelonoidis&species=carbonarius&search_param=%28%28common_name%3D%27Chelonoidis+carbonarius%27%29%29
35	52	Red-footed tortoise	Chelonoidis carbonarius	Testudines	Turtle	Colombia	America	WC/FB	https://reptile-database.reptarium.cz/species?genus=Chelonoidis&species=carbonarius&search_param=%28%28common_name%3D%27Chelonoidis+carbonarius%27%29%29
35	52	Red-footed tortoise	Chelonoidis carbonarius	Testudines	Turtle	Colombia	America	WC/FB	https://reptile-database.reptarium.cz/species?genus=Chelonoidis&species=carbonarius&search_param=%28%28common_name%3D%27Chelonoidis+carbonarius%27%29%29
35	52	Red-footed tortoise	Chelonoidis carbonarius	Testudines	Turtle	Colombia	America	WC/FB	https://reptile-database.reptarium.cz/species?genus=Chelonoidis&species=carbonarius&search_param=%28%28common_name%3D%27Chelonoidis+carbonarius%27%29%29
35	52	Red-footed tortoise	Chelonoidis carbonarius	Testudines	Turtle	Colombia	America	WC/FB	https://reptile-database.reptarium.cz/species?genus=Chelonoidis&species=carbonarius&search_param=%28%28common_name%3D%27Chelonoidis+carbonarius%27%29%29
35	52	Red-footed tortoise	Chelonoidis carbonarius	Testudines	Turtle	Colombia	America	WC/FB	https://reptile-database.reptarium.cz/species?genus=Chelonoidis&species=carbonarius&search_param=%28%28common_name%3D%27Chelonoidis+carbonarius%27%29%29
35	53	Red-footed tortoise	Chelonoidis carbonarius	Testudines	Turtle	Colombia	America	WC/FB	https://reptile-database.reptarium.cz/species?genus=Chelonoidis&species=carbonarius&search_param=%28%28common_name%3D%27Chelonoidis+carbonarius%27%29%29
35	53	Red-footed tortoise	Chelonoidis carbonarius	Testudines	Turtle	Colombia	America	WC/FB	https://reptile-database.reptarium.cz/species?genus=Chelonoidis&species=carbonarius&search_param=%28%28common_name%3D%27Chelonoidis+carbonarius%27%29%29
35	53	Red-footed tortoise	Chelonoidis carbonarius	Testudines	Turtle	Colombia	America	WC/FB	https://reptile-database.reptarium.cz/species?genus=Chelonoidis&species=carbonarius&search_param=%28%28common_name%3D%27Chelonoidis+carbonarius%27%29%29

Table S1: Animal species sampled in this study - country origin and categorization as captive bred (CB), farm bred (FB) and wild-caught (WC)

Shipment No.	Sample batch	Animal species	Scientific name of animal/s	Order	Suborder	Country origin	Continent	Captive bred (CB), farm bred (FB), wild-caught (WC)	Web URL used for assigning animal species to categories CB, FB and WC
35	53	Red-footed tortoise	Chelonoidis carbonarius	Testudines	Turtle	Colombia	America	WC/FB	https://reptile-database.reptarium.cz/species?genus=Chelonoidis&species=carbonarius&search_param=%28%28common_name%3D%27Chelonoidis+carbonarius%27%29%29
35	53	Red-footed tortoise	Chelonoidis carbonarius	Testudines	Turtle	Colombia	America	WC/FB	https://reptile-database.reptarium.cz/species?genus=Chelonoidis&species=carbonarius&search_param=%28%28common_name%3D%27Chelonoidis+carbonarius%27%29%29
35	53	Red-footed tortoise	Chelonoidis carbonarius	Testudines	Turtle	Colombia	America	WC/FB	https://reptile-database.reptarium.cz/species?genus=Chelonoidis&species=carbonarius&search_param=%28%28common_name%3D%27Chelonoidis+carbonarius%27%29%29
35	53	Red-footed tortoise	Chelonoidis carbonarius	Testudines	Turtle	Colombia	America	WC/FB	https://reptile-database.reptarium.cz/species?genus=Chelonoidis&species=carbonarius&search_param=%28%28common_name%3D%27Chelonoidis+carbonarius%27%29%29
35	52	Red-footed tortoise	Chelonoidis carbonarius	Testudines	Turtle	Colombia	America	WC/FB	https://reptile-database.reptarium.cz/species?genus=Chelonoidis&species=carbonarius&search_param=%28%28common_name%3D%27Chelonoidis+carbonarius%27%29%29
35	52	Red-footed tortoise	Chelonoidis carbonarius	Testudines	Turtle	Colombia	America	WC/FB	https://reptile-database.reptarium.cz/species?genus=Chelonoidis&species=carbonarius&search_param=%28%28common_name%3D%27Chelonoidis+carbonarius%27%29%29
35	52	Red-footed tortoise	Chelonoidis carbonarius	Testudines	Turtle	Colombia	America	WC/FB	https://reptile-database.reptarium.cz/species?genus=Chelonoidis&species=carbonarius&search_param=%28%28common_name%3D%27Chelonoidis+carbonarius%27%29%29
35	52	Red-footed tortoise	Chelonoidis carbonarius	Testudines	Turtle	Colombia	America	WC/FB	https://reptile-database.reptarium.cz/species?genus=Chelonoidis&species=carbonarius&search_param=%28%28common_name%3D%27Chelonoidis+carbonarius%27%29%29
35	52	Red-footed tortoise	Chelonoidis carbonarius	Testudines	Turtle	Colombia	America	WC/FB	https://reptile-database.reptarium.cz/species?genus=Chelonoidis&species=carbonarius&search_param=%28%28common_name%3D%27Chelonoidis+carbonarius%27%29%29
35	52	Red-footed tortoise	Chelonoidis carbonarius	Testudines	Turtle	Colombia	America	WC/FB	https://reptile-database.reptarium.cz/species?genus=Chelonoidis&species=carbonarius&search_param=%28%28common_name%3D%27Chelonoidis+carbonarius%27%29%29
35	52	Red-footed tortoise	Chelonoidis carbonarius	Testudines	Turtle	Colombia	America	WC/FB	https://reptile-database.reptarium.cz/species?genus=Chelonoidis&species=carbonarius&search_param=%28%28common_name%3D%27Chelonoidis+carbonarius%27%29%29
35	52	Red-footed tortoise	Chelonoidis carbonarius	Testudines	Turtle	Colombia	America	WC/FB	https://reptile-database.reptarium.cz/species?genus=Chelonoidis&species=carbonarius&search_param=%28%28common_name%3D%27Chelonoidis+carbonarius%27%29%29

Table S1: Animal species sampled in this study - country origin and categorization as captive bred (CB), farm bred (FB) and wild-caught (WC)

Shipment No.	Sample batch	Animal species	Scientific name of animal/s	Order	Suborder	Country origin	Continent	Captive bred (CB), farm bred (FB), wild-caught (WC)	Web URL used for assigning animal species to categories CB, FB and WC
35	52	Red-footed tortoise	Chelonoidis carbonarius	Testudines	Turtle	Colombia	America	WC/FB	https://reptile-database.reptarium.cz/species?genus=Chelonoidis&species=carbonarius&search_param=%28%28common_name%3D%27Chelonoidis+carbonarius%27%29%29
35	52	Red-footed tortoise	Chelonoidis carbonarius	Testudines	Turtle	Colombia	America	WC/FB	https://reptile-database.reptarium.cz/species?genus=Chelonoidis&species=carbonarius&search_param=%28%28common_name%3D%27Chelonoidis+carbonarius%27%29%29
36	53	Horsefield's tortoise	Testudo horsfieldii	Testudines	Turtle	Uzbekistan	Asia	FB	https://reptile-database.reptarium.cz/species?genus=Testudo&species=horsfieldii&search_param=%28%28common_name%3D%27Testudo+horsfieldii%27%29%29
36	53	Horsefield's tortoise	Testudo horsfieldii	Testudines	Turtle	Uzbekistan	Asia	FB	https://reptile-database.reptarium.cz/species?genus=Testudo&species=horsfieldii&search_param=%28%28common_name%3D%27Testudo+horsfieldii%27%29%29
36	53	Horsefield's tortoise	Testudo horsfieldii	Testudines	Turtle	Uzbekistan	Asia	FB	https://reptile-database.reptarium.cz/species?genus=Testudo&species=horsfieldii&search_param=%28%28common_name%3D%27Testudo+horsfieldii%27%29%29
36	53	Horsefield's tortoise	Testudo horsfieldii	Testudines	Turtle	Uzbekistan	Asia	FB	https://reptile-database.reptarium.cz/species?genus=Testudo&species=horsfieldii&search_param=%28%28common_name%3D%27Testudo+horsfieldii%27%29%29
36	53	Horsefield's tortoise	Testudo horsfieldii	Testudines	Turtle	Uzbekistan	Asia	FB	https://reptile-database.reptarium.cz/species?genus=Testudo&species=horsfieldii&search_param=%28%28common_name%3D%27Testudo+horsfieldii%27%29%29
36	53	Horsefield's tortoise	Testudo horsfieldii	Testudines	Turtle	Uzbekistan	Asia	FB	https://reptile-database.reptarium.cz/species?genus=Testudo&species=horsfieldii&search_param=%28%28common_name%3D%27Testudo+horsfieldii%27%29%29
36	53	Horsefield's tortoise	Testudo horsfieldii	Testudines	Turtle	Uzbekistan	Asia	FB	https://reptile-database.reptarium.cz/species?genus=Testudo&species=horsfieldii&search_param=%28%28common_name%3D%27Testudo+horsfieldii%27%29%29
36	53	Horsefield's tortoise	Testudo horsfieldii	Testudines	Turtle	Uzbekistan	Asia	FB	https://reptile-database.reptarium.cz/species?genus=Testudo&species=horsfieldii&search_param=%28%28common_name%3D%27Testudo+horsfieldii%27%29%29
36	53	Horsefield's tortoise	Testudo horsfieldii	Testudines	Turtle	Uzbekistan	Asia	FB	https://reptile-database.reptarium.cz/species?genus=Testudo&species=horsfieldii&search_param=%28%28common_name%3D%27Testudo+horsfieldii%27%29%29
36	53	Horsefield's tortoise	Testudo horsfieldii	Testudines	Turtle	Uzbekistan	Asia	FB	https://reptile-database.reptarium.cz/species?genus=Testudo&species=horsfieldii&search_param=%28%28common_name%3D%27Testudo+horsfieldii%27%29%29

Table S1: Animal species sampled in this study - country origin and categorization as captive bred (CB), farm bred (FB) and wild-caught (WC)

Shipment No.	Sample batch	Animal species	Scientific name of animal/s	Order	Suborder	Country origin	Continent	Captive bred (CB), farm bred (FB), wild-caught (WC)	Web URL used for assigning animal species to categories CB, FB and WC
36	53	Horsefield's tortoise	Testudo horsfieldii	Testudines	Turtle	Uzbekistan	Asia	FB	https://reptile-database.reptarium.cz/species?genus=Testudo&species=horsfieldii&search_param=%28%28common_name%3D%27Testudo+horsfieldii%27%29%29
36	53	Horsefield's tortoise	Testudo horsfieldii	Testudines	Turtle	Uzbekistan	Asia	FB	https://reptile-database.reptarium.cz/species?genus=Testudo&species=horsfieldii&search_param=%28%28common_name%3D%27Testudo+horsfieldii%27%29%29
36	53	Horsefield's tortoise	Testudo horsfieldii	Testudines	Turtle	Uzbekistan	Asia	FB	https://reptile-database.reptarium.cz/species?genus=Testudo&species=horsfieldii&search_param=%28%28common_name%3D%27Testudo+horsfieldii%27%29%29
36	53	Horsefield's tortoise	Testudo horsfieldii	Testudines	Turtle	Uzbekistan	Asia	FB	https://reptile-database.reptarium.cz/species?genus=Testudo&species=horsfieldii&search_param=%28%28common_name%3D%27Testudo+horsfieldii%27%29%29
36	53	Horsefield's tortoise	Testudo horsfieldii	Testudines	Turtle	Uzbekistan	Asia	FB	https://reptile-database.reptarium.cz/species?genus=Testudo&species=horsfieldii&search_param=%28%28common_name%3D%27Testudo+horsfieldii%27%29%29
36	53	Horsefield's tortoise	Testudo horsfieldii	Testudines	Turtle	Uzbekistan	Asia	FB	https://reptile-database.reptarium.cz/species?genus=Testudo&species=horsfieldii&search_param=%28%28common_name%3D%27Testudo+horsfieldii%27%29%29
36	53	Horsefield's tortoise	Testudo horsfieldii	Testudines	Turtle	Uzbekistan	Asia	FB	https://reptile-database.reptarium.cz/species?genus=Testudo&species=horsfieldii&search_param=%28%28common_name%3D%27Testudo+horsfieldii%27%29%29
36	53	Horsefield's tortoise	Testudo horsfieldii	Testudines	Turtle	Uzbekistan	Asia	FB	https://reptile-database.reptarium.cz/species?genus=Testudo&species=horsfieldii&search_param=%28%28common_name%3D%27Testudo+horsfieldii%27%29%29
36	53	Horsefield's tortoise	Testudo horsfieldii	Testudines	Turtle	Uzbekistan	Asia	FB	https://reptile-database.reptarium.cz/species?genus=Testudo&species=horsfieldii&search_param=%28%28common_name%3D%27Testudo+horsfieldii%27%29%29
36	53	Horsefield's tortoise	Testudo horsfieldii	Testudines	Turtle	Uzbekistan	Asia	FB	https://reptile-database.reptarium.cz/species?genus=Testudo&species=horsfieldii&search_param=%28%28common_name%3D%27Testudo+horsfieldii%27%29%29
36	53	Horsefield's tortoise	Testudo horsfieldii	Testudines	Turtle	Uzbekistan	Asia	FB	https://reptile-database.reptarium.cz/species?genus=Testudo&species=horsfieldii&search_param=%28%28common_name%3D%27Testudo+horsfieldii%27%29%29
36	53	Horsefield's tortoise	Testudo horsfieldii	Testudines	Turtle	Uzbekistan	Asia	FB	https://reptile-database.reptarium.cz/species?genus=Testudo&species=horsfieldii&search_param=%28%28common_name%3D%27Testudo+horsfieldii%27%29%29

Table S1: Animal species sampled in this study - country origin and categorization as captive bred (CB), farm bred (FB) and wild-caught (WC)

Shipment No.	Sample batch	Animal species	Scientific name of animal/s	Order	Suborder	Country origin	Continent	Captive bred (CB), farm bred (FB), wild-caught (WC)	Web URL used for assigning animal species to categories CB, FB and WC
37	54	Rough greensnake	Opheodrys aestivus	Squamata	Snake	USA	America	WC	https://reptile-database.reptarium.cz/species?genus=Opheodrys&species=aestivus&search_param=%28%28common_name%3D%27Opheodrys+aestivus%27%29%29
37	54	Rough greensnake	Opheodrys aestivus	Squamata	Snake	USA	America	WC	https://reptile-database.reptarium.cz/species?genus=Opheodrys&species=aestivus&search_param=%28%28common_name%3D%27Opheodrys+aestivus%27%29%29
37	54	Rough greensnake	Opheodrys aestivus	Squamata	Snake	USA	America	WC	https://reptile-database.reptarium.cz/species?genus=Opheodrys&species=aestivus&search_param=%28%28common_name%3D%27Opheodrys+aestivus%27%29%29
37	54	Rough greensnake	Opheodrys aestivus	Squamata	Snake	USA	America	WC	https://reptile-database.reptarium.cz/species?genus=Opheodrys&species=aestivus&search_param=%28%28common_name%3D%27Opheodrys+aestivus%27%29%29
37	54	Rough greensnake	Opheodrys aestivus	Squamata	Snake	USA	America	WC	https://reptile-database.reptarium.cz/species?genus=Opheodrys&species=aestivus&search_param=%28%28common_name%3D%27Opheodrys+aestivus%27%29%29
37	54	Rough greensnake	Opheodrys aestivus	Squamata	Snake	USA	America	WC	https://reptile-database.reptarium.cz/species?genus=Opheodrys&species=aestivus&search_param=%28%28common_name%3D%27Opheodrys+aestivus%27%29%29
37	54	Rough greensnake	Opheodrys aestivus	Squamata	Snake	USA	America	WC	https://reptile-database.reptarium.cz/species?genus=Opheodrys&species=aestivus&search_param=%28%28common_name%3D%27Opheodrys+aestivus%27%29%29
37	54	Rough greensnake	Opheodrys aestivus	Squamata	Snake	USA	America	WC	https://reptile-database.reptarium.cz/species?genus=Opheodrys&species=aestivus&search_param=%28%28common_name%3D%27Opheodrys+aestivus%27%29%29
38	55	Armored pricklenape	Acanthosaura armata	Squamata	Lizard	Vietnam	Asia	WC	https://reptile-database.reptarium.cz/species?genus=Acanthosaura&species=armata&search_param=%28%28common_name%3D%27Acanthosaura+armata%27%29%29
38	55	Armored pricklenape	Acanthosaura armata	Squamata	Lizard	Vietnam	Asia	WC	https://reptile-database.reptarium.cz/species?genus=Acanthosaura&species=armata&search_param=%28%28common_name%3D%27Acanthosaura+armata%27%29%29
38	55	Armored pricklenape	Acanthosaura armata	Squamata	Lizard	Vietnam	Asia	WC	https://reptile-database.reptarium.cz/species?genus=Acanthosaura&species=armata&search_param=%28%28common_name%3D%27Acanthosaura+armata%27%29%29
38	55	Armored pricklenape	Acanthosaura armata	Squamata	Lizard	Vietnam	Asia	WC	https://reptile-database.reptarium.cz/species?genus=Acanthosaura&species=armata&search_param=%28%28common_name%3D%27Acanthosaura+armata%27%29%29

Table S1: Animal species sampled in this study - country origin and categorization as captive bred (CB), farm bred (FB) and wild-caught (WC)

Shipment No.	Sample batch	Animal species	Scientific name of animal/s	Order	Suborder	Country origin	Continent	Captive bred (CB), farm bred (FB), wild-caught (WC)	Web URL used for assigning animal species to categories CB, FB and WC
38	55	Armored pricklenape	Acanthosaura armata	Squamata	Lizard	Vietnam	Asia	WC	https://reptile-database.reptarium.cz/species?genus=Acanthosaura&species=armata&search_param=%28%28common_name%3D%27Acanthosaura+armata%27%29%29
38	55	Armored pricklenape	Acanthosaura armata	Squamata	Lizard	Vietnam	Asia	WC	https://reptile-database.reptarium.cz/species?genus=Acanthosaura&species=armata&search_param=%28%28common_name%3D%27Acanthosaura+armata%27%29%29
38	56	Golden gecko / Grossmann's gecko	Gekko badenii/Gekko grossmanni	Squamata	Lizard	Vietnam	Asia	WC	https://reptile-database.reptarium.cz/species?genus=Gekko&species=badenii&search_param=%28%28common_name%3D%27golden+gecko%27%29%29 , https://reptile-database.reptarium.cz/species?genus=Gekko&species=grossmanni&search_param=%28%28common_name%3D%27gekko+grossmanni%27%29%29
38	56	Golden gecko / Grossmann's gecko	Gekko badenii/Gekko grossmanni	Squamata	Lizard	Vietnam	Asia	WC	https://reptile-database.reptarium.cz/species?genus=Gekko&species=badenii&search_param=%28%28common_name%3D%27golden+gecko%27%29%29 , https://reptile-database.reptarium.cz/species?genus=Gekko&species=grossmanni&search_param=%28%28common_name%3D%27gekko+grossmanni%27%29%29
38	56	Golden gecko / Grossmann's gecko	Gekko badenii/Gekko grossmanni	Squamata	Lizard	Vietnam	Asia	WC	https://reptile-database.reptarium.cz/species?genus=Gekko&species=badenii&search_param=%28%28common_name%3D%27golden+gecko%27%29%29 , https://reptile-database.reptarium.cz/species?genus=Gekko&species=grossmanni&search_param=%28%28common_name%3D%27gekko+grossmanni%27%29%29
38	56	Golden gecko / Grossmann's gecko	Gekko badenii/Gekko grossmanni	Squamata	Lizard	Vietnam	Asia	WC	https://reptile-database.reptarium.cz/species?genus=Gekko&species=badenii&search_param=%28%28common_name%3D%27golden+gecko%27%29%29 , https://reptile-database.reptarium.cz/species?genus=Gekko&species=grossmanni&search_param=%28%28common_name%3D%27gekko+grossmanni%27%29%29
38	56	Golden gecko / Grossmann's gecko	Gekko badenii/Gekko grossmanni	Squamata	Lizard	Vietnam	Asia	WC	https://reptile-database.reptarium.cz/species?genus=Gekko&species=badenii&search_param=%28%28common_name%3D%27golden+gecko%27%29%29 , https://reptile-database.reptarium.cz/species?genus=Gekko&species=grossmanni&search_param=%28%28common_name%3D%27gekko+grossmanni%27%29%29

Table S1: Animal species sampled in this study - country origin and categorization as captive bred (CB), farm bred (FB) and wild-caught (WC)

Shipment No.	Sample batch	Animal species	Scientific name of animal/s	Order	Suborder	Country origin	Continent	Captive bred (CB), farm bred (FB), wild-caught (WC)	Web URL used for assigning animal species to categories CB, FB and WC
38	56	Golden gecko / Grossmann's gecko	Gekko badenii/Gekko grossmanni	Squamata	Lizard	Vietnam	Asia	WC	https://reptile-database.reptarium.cz/species?genus=Gekko&species=badenii&search_param=%28%28common_name%3D%27golden+gecko%27%29%29 , https://reptile-database.reptarium.cz/species?genus=Gekko&species=grossmanni&search_param=%28%28common_name%3D%27gekko+grossmanni%27%29%29
38	56	Golden gecko / Grossmann's gecko	Gekko badenii/Gekko grossmanni	Squamata	Lizard	Vietnam	Asia	WC	https://reptile-database.reptarium.cz/species?genus=Gekko&species=badenii&search_param=%28%28common_name%3D%27golden+gecko%27%29%29 , https://reptile-database.reptarium.cz/species?genus=Gekko&species=grossmanni&search_param=%28%28common_name%3D%27gekko+grossmanni%27%29%29
38	56	Golden gecko / Grossmann's gecko	Gekko badenii/Gekko grossmanni	Squamata	Lizard	Vietnam	Asia	WC	https://reptile-database.reptarium.cz/species?genus=Gekko&species=badenii&search_param=%28%28common_name%3D%27golden+gecko%27%29%29 , https://reptile-database.reptarium.cz/species?genus=Gekko&species=grossmanni&search_param=%28%28common_name%3D%27gekko+grossmanni%27%29%29
38	56	Golden gecko / Grossmann's gecko	Gekko badenii/Gekko grossmanni	Squamata	Lizard	Vietnam	Asia	WC	https://reptile-database.reptarium.cz/species?genus=Gekko&species=badenii&search_param=%28%28common_name%3D%27golden+gecko%27%29%29 , https://reptile-database.reptarium.cz/species?genus=Gekko&species=grossmanni&search_param=%28%28common_name%3D%27gekko+grossmanni%27%29%29
39	57	Blue crested lizard / Chinese water dragon	Calotes mystaceus/Physignathus cocincinus	Squamata	Lizard	Vietnam	Asia	WC	https://reptile-database.reptarium.cz/species?genus=Calotes&species=mystaceus&search_param=%28%28common_name%3D%27Calotes+mystaceus%27%29%29 , https://reptile-database.reptarium.cz/species?genus=Physignathus&species=cocincinus&search_param=%28%28common_name%3D%27Physignatus+cocincinus%27%29%29
39	57	Blue crested lizard / Chinese water dragon	Calotes mystaceus/Physignathus cocincinus	Squamata	Lizard	Vietnam	Asia	WC	https://reptile-database.reptarium.cz/species?genus=Calotes&species=mystaceus&search_param=%28%28common_name%3D%27Calotes+mystaceus%27%29%29 , https://reptile-database.reptarium.cz/species?genus=Physignathus&species=cocincinus&search_param=%28%28common_name%3D%27Physignatus+cocincinus%27%29%29
39	57	Blue crested lizard / Chinese water dragon	Calotes mystaceus/Physignathus cocincinus	Squamata	Lizard	Vietnam	Asia	WC	https://reptile-database.reptarium.cz/species?genus=Calotes&species=mystaceus&search_param=%28%28common_name%3D%27Calotes+mystaceus%27%29%29 , https://reptile-database.reptarium.cz/species?genus=Physignathus&species=cocincinus&search_param=%28%28common_name%3D%27Physignatus+cocincinus%27%29%29

Table S1: Animal species sampled in this study - country origin and categorization as captive bred (CB), farm bred (FB) and wild-caught (WC)

Shipment No.	Sample batch	Animal species	Scientific name of animal/s	Order	Suborder	Country origin	Continent	Captive bred (CB), farm bred (FB), wild-caught (WC)	Web URL used for assigning animal species to categories CB, FB and WC
39	57	Blue crested lizard / Chinese water dragon	Calotes mystaceus/Physignathus cocincinus	Squamata	Lizard	Vietnam	Asia	WC	https://reptile-database.reptarium.cz/species?genus=Calotes&species=mystaceus&search_param=%28%28common_name%3D%27Calotes+mystaceus%27%29%29 , https://reptile-database.reptarium.cz/species?genus=Physignathus&species=cocincinus&search_param=%28%28common_name%3D%27Physignatus+cocincinus%27%29%29
39	57	Blue crested lizard / Chinese water dragon	Calotes mystaceus/Physignathus cocincinus	Squamata	Lizard	Vietnam	Asia	WC	https://reptile-database.reptarium.cz/species?genus=Calotes&species=mystaceus&search_param=%28%28common_name%3D%27Calotes+mystaceus%27%29%29 , https://reptile-database.reptarium.cz/species?genus=Physignathus&species=cocincinus&search_param=%28%28common_name%3D%27Physignatus+cocincinus%27%29%29
39	57	Blue crested lizard / Chinese water dragon	Calotes mystaceus/Physignathus cocincinus	Squamata	Lizard	Vietnam	Asia	WC	https://reptile-database.reptarium.cz/species?genus=Calotes&species=mystaceus&search_param=%28%28common_name%3D%27Calotes+mystaceus%27%29%29 , https://reptile-database.reptarium.cz/species?genus=Physignathus&species=cocincinus&search_param=%28%28common_name%3D%27Physignatus+cocincinus%27%29%29
39	57	Blue crested lizard / Chinese water dragon	Calotes mystaceus/Physignathus cocincinus	Squamata	Lizard	Vietnam	Asia	WC	https://reptile-database.reptarium.cz/species?genus=Calotes&species=mystaceus&search_param=%28%28common_name%3D%27Calotes+mystaceus%27%29%29 , https://reptile-database.reptarium.cz/species?genus=Physignathus&species=cocincinus&search_param=%28%28common_name%3D%27Physignatus+cocincinus%27%29%29
39	58	Asian grass lizard	Takydromus sexlineatus	Squamata	Lizard	Vietnam	Asia	WC	https://reptile-database.reptarium.cz/species?genus=Takydromus&species=sexlineatus&search_param=%28%28common_name%3D%27Takydromus+sexlineatus%27%29%29
39	58	Asian grass lizard	Takydromus sexlineatus	Squamata	Lizard	Vietnam	Asia	WC	https://reptile-database.reptarium.cz/species?genus=Takydromus&species=sexlineatus&search_param=%28%28common_name%3D%27Takydromus+sexlineatus%27%29%29
39	58	Asian grass lizard	Takydromus sexlineatus	Squamata	Lizard	Vietnam	Asia	WC	https://reptile-database.reptarium.cz/species?genus=Takydromus&species=sexlineatus&search_param=%28%28common_name%3D%27Takydromus+sexlineatus%27%29%29
39	58	Asian grass lizard	Takydromus sexlineatus	Squamata	Lizard	Vietnam	Asia	WC	https://reptile-database.reptarium.cz/species?genus=Takydromus&species=sexlineatus&search_param=%28%28common_name%3D%27Takydromus+sexlineatus%27%29%29
39	58	Asian grass lizard	Takydromus sexlineatus	Squamata	Lizard	Vietnam	Asia	WC	https://reptile-database.reptarium.cz/species?genus=Takydromus&species=sexlineatus&search_param=%28%28common_name%3D%27Takydromus+sexlineatus%27%29%29

Table S1: Animal species sampled in this study - country origin and categorization as captive bred (CB), farm bred (FB) and wild-caught (WC)

Shipment No.	Sample batch	Animal species	Scientific name of animal/s	Order	Suborder	Country origin	Continent	Captive bred (CB), farm bred (FB), wild-caught (WC)	Web URL used for assigning animal species to categories CB, FB and WC
39	58	Asian grass lizard	Takydromus sexlineatus	Squamata	Lizard	Vietnam	Asia	WC	https://reptile-database.reptarium.cz/species?genus=Takydromus&species=sexlineatus&search_param=%28%28common_name%3D%27Takydromus+sexlineatus%27%29%29
39	58	Asian grass lizard	Takydromus sexlineatus	Squamata	Lizard	Vietnam	Asia	WC	https://reptile-database.reptarium.cz/species?genus=Takydromus&species=sexlineatus&search_param=%28%28common_name%3D%27Takydromus+sexlineatus%27%29%29
39	58	Asian grass lizard	Takydromus sexlineatus	Squamata	Lizard	Vietnam	Asia	WC	https://reptile-database.reptarium.cz/species?genus=Takydromus&species=sexlineatus&search_param=%28%28common_name%3D%27Takydromus+sexlineatus%27%29%29
39	58	Asian grass lizard	Takydromus sexlineatus	Squamata	Lizard	Vietnam	Asia	WC	https://reptile-database.reptarium.cz/species?genus=Takydromus&species=sexlineatus&search_param=%28%28common_name%3D%27Takydromus+sexlineatus%27%29%29
39	58	Asian grass lizard	Takydromus sexlineatus	Squamata	Lizard	Vietnam	Asia	WC	https://reptile-database.reptarium.cz/species?genus=Takydromus&species=sexlineatus&search_param=%28%28common_name%3D%27Takydromus+sexlineatus%27%29%29
39	58	Asian grass lizard	Takydromus sexlineatus	Squamata	Lizard	Vietnam	Asia	WC	https://reptile-database.reptarium.cz/species?genus=Takydromus&species=sexlineatus&search_param=%28%28common_name%3D%27Takydromus+sexlineatus%27%29%29
39	59	brown pricklenape	Acanthosaura lepidogaster	Squamata	Lizard	Vietnam	Asia	WC	https://reptile-database.reptarium.cz/species?genus=Acanthosaura&species=lepidogaster&search_param=%28%28common_name%3D%27Acanthosaura+lepidogaster%27%29%29
39	59	brown pricklenape	Acanthosaura lepidogaster	Squamata	Lizard	Vietnam	Asia	WC	https://reptile-database.reptarium.cz/species?genus=Acanthosaura&species=lepidogaster&search_param=%28%28common_name%3D%27Acanthosaura+lepidogaster%27%29%29
39	59	brown pricklenape	Acanthosaura lepidogaster	Squamata	Lizard	Vietnam	Asia	WC	https://reptile-database.reptarium.cz/species?genus=Acanthosaura&species=lepidogaster&search_param=%28%28common_name%3D%27Acanthosaura+lepidogaster%27%29%29
39	59	Brown pricklenape	Acanthosaura lepidogaster	Squamata	Lizard	Vietnam	Asia	WC	https://reptile-database.reptarium.cz/species?genus=Acanthosaura&species=lepidogaster&search_param=%28%28common_name%3D%27Acanthosaura+lepidogaster%27%29%29
39	59	Brown pricklenape	Acanthosaura lepidogaster	Squamata	Lizard	Vietnam	Asia	WC	https://reptile-database.reptarium.cz/species?genus=Acanthosaura&species=lepidogaster&search_param=%28%28common_name%3D%27Acanthosaura+lepidogaster%27%29%29
39	59	Brown pricklenape	Acanthosaura lepidogaster	Squamata	Lizard	Vietnam	Asia	WC	https://reptile-database.reptarium.cz/species?genus=Acanthosaura&species=lepidogaster&search_param=%28%28common_name%3D%27Acanthosaura+lepidogaster%27%29%29

Table S1: Animal species sampled in this study - country origin and categorization as captive bred (CB), farm bred (FB) and wild-caught (WC)

Shipment No.	Sample batch	Animal species	Scientific name of animal/s	Order	Suborder	Country origin	Continent	Captive bred (CB), farm bred (FB), wild-caught (WC)	Web URL used for assigning animal species to categories CB, FB and WC
39	59	Brown pricklenape	Acanthosaura lepidogaster	Squamata	Lizard	Vietnam	Asia	WC	https://reptile-database.reptarium.cz/species?genus=Acanthosaura&species=lepidogaster&search_param=%28%28common_name%3D%27Acanthosaura+lepidogaster%27%29%29
39	59	Brown pricklenape	Acanthosaura lepidogaster	Squamata	Lizard	Vietnam	Asia	WC	https://reptile-database.reptarium.cz/species?genus=Acanthosaura&species=lepidogaster&search_param=%28%28common_name%3D%27Acanthosaura+lepidogaster%27%29%29
39	59	Brown pricklenape	Acanthosaura lepidogaster	Squamata	Lizard	Vietnam	Asia	WC	https://reptile-database.reptarium.cz/species?genus=Acanthosaura&species=lepidogaster&search_param=%28%28common_name%3D%27Acanthosaura+lepidogaster%27%29%29
39	59	Brown pricklenape	Acanthosaura lepidogaster	Squamata	Lizard	Vietnam	Asia	WC	https://reptile-database.reptarium.cz/species?genus=Acanthosaura&species=lepidogaster&search_param=%28%28common_name%3D%27Acanthosaura+lepidogaster%27%29%29
39	60	Eastern garden lizard	Calotes versicolor	Squamata	Lizard	Vietnam	Asia	WC	https://reptile-database.reptarium.cz/species?genus=Calotes&species=versicolor&search_param=%28%28common_name%3D%27Calotes+versicolor%27%29%29
39	60	Eastern garden lizard	Calotes versicolor	Squamata	Lizard	Vietnam	Asia	WC	https://reptile-database.reptarium.cz/species?genus=Calotes&species=versicolor&search_param=%28%28common_name%3D%27Calotes+versicolor%27%29%29
39	60	Eastern garden lizard	Calotes versicolor	Squamata	Lizard	Vietnam	Asia	WC	https://reptile-database.reptarium.cz/species?genus=Calotes&species=versicolor&search_param=%28%28common_name%3D%27Calotes+versicolor%27%29%29
39	60	Eastern garden lizard	Calotes versicolor	Squamata	Lizard	Vietnam	Asia	WC	https://reptile-database.reptarium.cz/species?genus=Calotes&species=versicolor&search_param=%28%28common_name%3D%27Calotes+versicolor%27%29%29
39	60	Eastern garden lizard	Calotes versicolor	Squamata	Lizard	Vietnam	Asia	WC	https://reptile-database.reptarium.cz/species?genus=Calotes&species=versicolor&search_param=%28%28common_name%3D%27Calotes+versicolor%27%29%29
39	60	Eastern garden lizard	Calotes versicolor	Squamata	Lizard	Vietnam	Asia	WC	https://reptile-database.reptarium.cz/species?genus=Calotes&species=versicolor&search_param=%28%28common_name%3D%27Calotes+versicolor%27%29%29
39	60	Eastern garden lizard	Calotes versicolor	Squamata	Lizard	Vietnam	Asia	WC	https://reptile-database.reptarium.cz/species?genus=Calotes&species=versicolor&search_param=%28%28common_name%3D%27Calotes+versicolor%27%29%29
39	60	Eastern garden lizard	Calotes versicolor	Squamata	Lizard	Vietnam	Asia	WC	https://reptile-database.reptarium.cz/species?genus=Calotes&species=versicolor&search_param=%28%28common_name%3D%27Calotes+versicolor%27%29%29

Table S1: Animal species sampled in this study - country origin and categorization as captive bred (CB), farm bred (FB) and wild-caught (WC)

Shipment No.	Sample batch	Animal species	Scientific name of animal/s	Order	Suborder	Country origin	Continent	Captive bred (CB), farm bred (FB), wild-caught (WC)	Web URL used for assigning animal species to categories CB, FB and WC
39	60	Eastern garden lizard	Calotes versicolor	Squamata	Lizard	Vietnam	Asia	WC	https://reptile-database.reptarium.cz/species?genus=Calotes&species=versicolor&search_param=%28%28common_name%3D%27Calotes+versicolor%27%29%29
39	61	Green pricklenape / Brown pricklenape	Acanthosaura capra/lepidogaster	Squamata	Lizard	Vietnam	Asia	WC	https://reptile-database.reptarium.cz/species?genus=Acanthosaura&species=capra&search_param=%28%28common_name%3D%27Acanthosaura+capra%27%29%29 , https://reptile-database.reptarium.cz/species?genus=Acanthosaura&species=lepidogaster&search_param=%28%28common_name%3D%27Acanthosaura+lepidogaster%27%29%29
39	61	Green pricklenape / Brown pricklenape	Acanthosaura capra/lepidogaster	Squamata	Lizard	Vietnam	Asia	WC	https://reptile-database.reptarium.cz/species?genus=Acanthosaura&species=capra&search_param=%28%28common_name%3D%27Acanthosaura+capra%27%29%29 , https://reptile-database.reptarium.cz/species?genus=Acanthosaura&species=lepidogaster&search_param=%28%28common_name%3D%27Acanthosaura+lepidogaster%27%29%29
39	61	Green pricklenape / Brown pricklenape	Acanthosaura capra/lepidogaster	Squamata	Lizard	Vietnam	Asia	WC	https://reptile-database.reptarium.cz/species?genus=Acanthosaura&species=capra&search_param=%28%28common_name%3D%27Acanthosaura+capra%27%29%29 , https://reptile-database.reptarium.cz/species?genus=Acanthosaura&species=lepidogaster&search_param=%28%28common_name%3D%27Acanthosaura+lepidogaster%27%29%29
39	61	Green pricklenape / Brown pricklenape	Acanthosaura capra/lepidogaster	Squamata	Lizard	Vietnam	Asia	WC	https://reptile-database.reptarium.cz/species?genus=Acanthosaura&species=capra&search_param=%28%28common_name%3D%27Acanthosaura+capra%27%29%29 , https://reptile-database.reptarium.cz/species?genus=Acanthosaura&species=lepidogaster&search_param=%28%28common_name%3D%27Acanthosaura+lepidogaster%27%29%29
39	61	Green pricklenape / Brown pricklenape	Acanthosaura capra/lepidogaster	Squamata	Lizard	Vietnam	Asia	WC	https://reptile-database.reptarium.cz/species?genus=Acanthosaura&species=capra&search_param=%28%28common_name%3D%27Acanthosaura+capra%27%29%29 , https://reptile-database.reptarium.cz/species?genus=Acanthosaura&species=lepidogaster&search_param=%28%28common_name%3D%27Acanthosaura+lepidogaster%27%29%29
39	61	Green pricklenape / Brown pricklenape	Acanthosaura capra/lepidogaster	Squamata	Lizard	Vietnam	Asia	WC	https://reptile-database.reptarium.cz/species?genus=Acanthosaura&species=capra&search_param=%28%28common_name%3D%27Acanthosaura+capra%27%29%29 , https://reptile-database.reptarium.cz/species?genus=Acanthosaura&species=lepidogaster&search_param=%28%28common_name%3D%27Acanthosaura+lepidogaster%27%29%29

Table S1: Animal species sampled in this study - country origin and categorization as captive bred (CB), farm bred (FB) and wild-caught (WC)

Shipment No.	Sample batch	Animal species	Scientific name of animal/s	Order	Suborder	Country origin	Continent	Captive bred (CB), farm bred (FB), wild-caught (WC)	Web URL used for assigning animal species to categories CB, FB and WC
39	61	Green pricklenape / Brown pricklenape	Acanthosaura capra/lepidogaster	Squamata	Lizard	Vietnam	Asia	WC	https://reptile-database.reptarium.cz/species?genus=Acanthosaura&species=capra&search_param=%28%28common_name%3D%27Acanthosaura+capra%27%29%29 , https://reptile-database.reptarium.cz/species?genus=Acanthosaura&species=lepidogaster&search_param=%28%28common_name%3D%27Acanthosaura+lepidogaster%27%29%29
39	62	Chinese water dragon	Physignatus cocincinus	Squamata	Lizard	Vietnam	Asia	WC	https://reptile-database.reptarium.cz/species?genus=Physignathus&species=cocincinus&search_param=%28%28common_name%3D%27gr%C3%BCne+wasseragame%27%29%29
39	62	Chinese water dragon	Physignatus cocincinus	Squamata	Lizard	Vietnam	Asia	WC	https://reptile-database.reptarium.cz/species?genus=Physignathus&species=cocincinus&search_param=%28%28common_name%3D%27gr%C3%BCne+wasseragame%27%29%29
39	62	Chinese water dragon	Physignatus cocincinus	Squamata	Lizard	Vietnam	Asia	WC	https://reptile-database.reptarium.cz/species?genus=Physignathus&species=cocincinus&search_param=%28%28common_name%3D%27gr%C3%BCne+wasseragame%27%29%29
39	62	Chinese water dragon	Physignatus cocincinus	Squamata	Lizard	Vietnam	Asia	WC	https://reptile-database.reptarium.cz/species?genus=Physignathus&species=cocincinus&search_param=%28%28common_name%3D%27gr%C3%BCne+wasseragame%27%29%29
39	62	Chinese water dragon	Physignatus cocincinus	Squamata	Lizard	Vietnam	Asia	WC	https://reptile-database.reptarium.cz/species?genus=Physignathus&species=cocincinus&search_param=%28%28common_name%3D%27gr%C3%BCne+wasseragame%27%29%29
39	62	Chinese water dragon	Physignatus cocincinus	Squamata	Lizard	Vietnam	Asia	WC	https://reptile-database.reptarium.cz/species?genus=Physignathus&species=cocincinus&search_param=%28%28common_name%3D%27gr%C3%BCne+wasseragame%27%29%29
39	62	Chinese water dragon	Physignatus cocincinus	Squamata	Lizard	Vietnam	Asia	WC	https://reptile-database.reptarium.cz/species?genus=Physignathus&species=cocincinus&search_param=%28%28common_name%3D%27gr%C3%BCne+wasseragame%27%29%29
39	62	Chinese water dragon	Physignatus cocincinus	Squamata	Lizard	Vietnam	Asia	WC	https://reptile-database.reptarium.cz/species?genus=Physignathus&species=cocincinus&search_param=%28%28common_name%3D%27gr%C3%BCne+wasseragame%27%29%29
39	62	Chinese water dragon	Physignatus cocincinus	Squamata	Lizard	Vietnam	Asia	WC	https://reptile-database.reptarium.cz/species?genus=Physignathus&species=cocincinus&search_param=%28%28common_name%3D%27gr%C3%BCne+wasseragame%27%29%29
39	62	Chinese water dragon	Physignatus cocincinus	Squamata	Lizard	Vietnam	Asia	WC	https://reptile-database.reptarium.cz/species?genus=Physignathus&species=cocincinus&search_param=%28%28common_name%3D%27gr%C3%BCne+wasseragame%27%29%29

Table S1: Animal species sampled in this study - country origin and categorization as captive bred (CB), farm bred (FB) and wild-caught (WC)

Shipment No.	Sample batch	Animal species	Scientific name of animal/s	Order	Suborder	Country origin	Continent	Captive bred (CB), farm bred (FB), wild-caught (WC)	Web URL used for assigning animal species to categories CB, FB and WC
39	62	Chinese water dragon	Physignatus cocincinus	Squamata	Lizard	Vietnam	Asia	WC	https://reptile-database.reptarium.cz/species?genus=Physignathus&species=cocincinus&search_param=%28%28common_name%3D%27gr%C3%BCne+wasseragame%27%29%29
39	62	Chinese water dragon	Physignatus cocincinus	Squamata	Lizard	Vietnam	Asia	WC	https://reptile-database.reptarium.cz/species?genus=Physignathus&species=cocincinus&search_param=%28%28common_name%3D%27gr%C3%BCne+wasseragame%27%29%29
39	62	Chinese water dragon	Physignatus cocincinus	Squamata	Lizard	Vietnam	Asia	WC	https://reptile-database.reptarium.cz/species?genus=Physignathus&species=cocincinus&search_param=%28%28common_name%3D%27gr%C3%BCne+wasseragame%27%29%29
40	63	Common leopard gecko	Eublepharis macularius	Squamata	Lizard	China	Asia	CB	https://reptile-database.reptarium.cz/species?genus=Eublepharis&species=macularius&search_param=%28%28common_name%3D%27Eublepharis+macularius%27%29%29
40	63	Common leopard gecko	Eublepharis macularius	Squamata	Lizard	China	Asia	CB	https://reptile-database.reptarium.cz/species?genus=Eublepharis&species=macularius&search_param=%28%28common_name%3D%27Eublepharis+macularius%27%29%29
40	63	Common leopard gecko	Eublepharis macularius	Squamata	Lizard	China	Asia	CB	https://reptile-database.reptarium.cz/species?genus=Eublepharis&species=macularius&search_param=%28%28common_name%3D%27Eublepharis+macularius%27%29%29
40	63	Common leopard gecko	Eublepharis macularius	Squamata	Lizard	China	Asia	CB	https://reptile-database.reptarium.cz/species?genus=Eublepharis&species=macularius&search_param=%28%28common_name%3D%27Eublepharis+macularius%27%29%29
40	63	Common leopard gecko	Eublepharis macularius	Squamata	Lizard	China	Asia	CB	https://reptile-database.reptarium.cz/species?genus=Eublepharis&species=macularius&search_param=%28%28common_name%3D%27Eublepharis+macularius%27%29%29
40	63	Common leopard gecko	Eublepharis macularius	Squamata	Lizard	China	Asia	CB	https://reptile-database.reptarium.cz/species?genus=Eublepharis&species=macularius&search_param=%28%28common_name%3D%27Eublepharis+macularius%27%29%29
40	63	Common leopard gecko	Eublepharis macularius	Squamata	Lizard	China	Asia	CB	https://reptile-database.reptarium.cz/species?genus=Eublepharis&species=macularius&search_param=%28%28common_name%3D%27Eublepharis+macularius%27%29%29
41	pre-trial	Tokay gecko	Gekko gecko	Squamata	Lizard	Vietnam	Asia	WC	https://reptile-database.reptarium.cz/species?genus=Gekko&species=gecko&search_param=%28%28common_name%3D%27Gekko+gecko%27%29%29
42	65	Rainbow boa	Epicrates maurus	Squamata	Snake	USA	America	CB	https://reptile-database.reptarium.cz/species?genus=Epicrates&species=maurus&search_param=%28%28common_name%3D%27Epicrates+cenchria+maurus%27%29%29

Table S1: Animal species sampled in this study - country origin and categorization as captive bred (CB), farm bred (FB) and wild-caught (WC)

Shipment No.	Sample batch	Animal species	Scientific name of animal/s	Order	Suborder	Country origin	Continent	Captive bred (CB), farm bred (FB), wild-caught (WC)	Web URL used for assigning animal species to categories CB, FB and WC
42	65	Rainbow boa	Epicrates maurus	Squamata	Snake	USA	America	CB	https://reptile-database.reptarium.cz/species?genus=Epicrates&species=maurus&search_param=%28%28common_name%3D%27Epicrates+cenchria+maurus%27%29%29
42	65	Rainbow boa	Epicrates maurus	Squamata	Snake	USA	America	CB	https://reptile-database.reptarium.cz/species?genus=Epicrates&species=maurus&search_param=%28%28common_name%3D%27Epicrates+cenchria+maurus%27%29%29
42	65	Rainbow boa	Epicrates maurus	Squamata	Snake	USA	America	CB	https://reptile-database.reptarium.cz/species?genus=Epicrates&species=maurus&search_param=%28%28common_name%3D%27Epicrates+cenchria+maurus%27%29%29
42	64	Rainbow boa	Epicrates maurus	Squamata	Snake	USA	America	CB	https://reptile-database.reptarium.cz/species?genus=Epicrates&species=maurus&search_param=%28%28common_name%3D%27Epicrates+cenchria+maurus%27%29%29
42	64	Rainbow boa	Epicrates maurus	Squamata	Snake	USA	America	CB	https://reptile-database.reptarium.cz/species?genus=Epicrates&species=maurus&search_param=%28%28common_name%3D%27Epicrates+cenchria+maurus%27%29%29
42	64	Rainbow boa	Epicrates maurus	Squamata	Snake	USA	America	CB	https://reptile-database.reptarium.cz/species?genus=Epicrates&species=maurus&search_param=%28%28common_name%3D%27Epicrates+cenchria+maurus%27%29%29
42	64	Rainbow boa	Epicrates maurus	Squamata	Snake	USA	America	CB	https://reptile-database.reptarium.cz/species?genus=Epicrates&species=maurus&search_param=%28%28common_name%3D%27Epicrates+cenchria+maurus%27%29%29
42	64	Rainbow boa	Epicrates maurus	Squamata	Snake	USA	America	CB	https://reptile-database.reptarium.cz/species?genus=Epicrates&species=maurus&search_param=%28%28common_name%3D%27Epicrates+cenchria+maurus%27%29%29
42	64	Rainbow boa	Epicrates maurus	Squamata	Snake	USA	America	CB	https://reptile-database.reptarium.cz/species?genus=Epicrates&species=maurus&search_param=%28%28common_name%3D%27Epicrates+cenchria+maurus%27%29%29
42	64	Rainbow boa	Epicrates maurus	Squamata	Snake	USA	America	CB	https://reptile-database.reptarium.cz/species?genus=Epicrates&species=maurus&search_param=%28%28common_name%3D%27Epicrates+cenchria+maurus%27%29%29
42	64	Rainbow boa	Epicrates maurus	Squamata	Snake	USA	America	CB	https://reptile-database.reptarium.cz/species?genus=Epicrates&species=maurus&search_param=%28%28common_name%3D%27Epicrates+cenchria+maurus%27%29%29
42	64	Rainbow boa	Epicrates maurus	Squamata	Snake	USA	America	CB	https://reptile-database.reptarium.cz/species?genus=Epicrates&species=maurus&search_param=%28%28common_name%3D%27Epicrates+cenchria+maurus%27%29%29

Table S1: Animal species sampled in this study - country origin and categorization as captive bred (CB), farm bred (FB) and wild-caught (WC)

Shipment No.	Sample batch	Animal species	Scientific name of animal/s	Order	Suborder	Country origin	Continent	Captive bred (CB), farm bred (FB), wild-caught (WC)	Web URL used for assigning animal species to categories CB, FB and WC
42	64	Rainbow boa	Epicrates maurus	Squamata	Snake	USA	America	CB	https://reptile-database.reptarium.cz/species?genus=Epicrates&species=maurus&search_param=%28%28common_name%3D%27Epicrates+cenchria+maurus%27%29%29
42	64	Rainbow boa	Epicrates maurus	Squamata	Snake	USA	America	CB	https://reptile-database.reptarium.cz/species?genus=Epicrates&species=maurus&search_param=%28%28common_name%3D%27Epicrates+cenchria+maurus%27%29%29
42	64	Rainbow boa	Epicrates maurus	Squamata	Snake	USA	America	CB	https://reptile-database.reptarium.cz/species?genus=Epicrates&species=maurus&search_param=%28%28common_name%3D%27Epicrates+cenchria+maurus%27%29%29
42	64	Rainbow boa	Epicrates maurus	Squamata	Snake	USA	America	CB	https://reptile-database.reptarium.cz/species?genus=Epicrates&species=maurus&search_param=%28%28common_name%3D%27Epicrates+cenchria+maurus%27%29%29
42	64	Rainbow boa	Epicrates maurus	Squamata	Snake	USA	America	CB	https://reptile-database.reptarium.cz/species?genus=Epicrates&species=maurus&search_param=%28%28common_name%3D%27Epicrates+cenchria+maurus%27%29%29
42	64	Rainbow boa	Epicrates maurus	Squamata	Snake	USA	America	CB	https://reptile-database.reptarium.cz/species?genus=Epicrates&species=maurus&search_param=%28%28common_name%3D%27Epicrates+cenchria+maurus%27%29%29
42	64	Rainbow boa	Epicrates maurus	Squamata	Snake	USA	America	CB	https://reptile-database.reptarium.cz/species?genus=Epicrates&species=maurus&search_param=%28%28common_name%3D%27Epicrates+cenchria+maurus%27%29%29
42	64	Rainbow boa	Epicrates maurus	Squamata	Snake	USA	America	CB	https://reptile-database.reptarium.cz/species?genus=Epicrates&species=maurus&search_param=%28%28common_name%3D%27Epicrates+cenchria+maurus%27%29%29
42	64	Rainbow boa	Epicrates maurus	Squamata	Snake	USA	America	CB	https://reptile-database.reptarium.cz/species?genus=Epicrates&species=maurus&search_param=%28%28common_name%3D%27Epicrates+cenchria+maurus%27%29%29
42	64	Rainbow boa	Epicrates maurus	Squamata	Snake	USA	America	CB	https://reptile-database.reptarium.cz/species?genus=Epicrates&species=maurus&search_param=%28%28common_name%3D%27Epicrates+cenchria+maurus%27%29%29
42	64	Rainbow boa	Epicrates maurus	Squamata	Snake	USA	America	CB	https://reptile-database.reptarium.cz/species?genus=Epicrates&species=maurus&search_param=%28%28common_name%3D%27Epicrates+cenchria+maurus%27%29%29
42	64	Rainbow boa	Epicrates maurus	Squamata	Snake	USA	America	CB	https://reptile-database.reptarium.cz/species?genus=Epicrates&species=maurus&search_param=%28%28common_name%3D%27Epicrates+cenchria+maurus%27%29%29

Table S1: Animal species sampled in this study - country origin and categorization as captive bred (CB), farm bred (FB) and wild-caught (WC)

Shipment No.	Sample batch	Animal species	Scientific name of animal/s	Order	Suborder	Country origin	Continent	Captive bred (CB), farm bred (FB), wild-caught (WC)	Web URL used for assigning animal species to categories CB, FB and WC
42	64	Rainbow boa	Epicrates maurus	Squamata	Snake	USA	America	CB	https://reptile-database.reptarium.cz/species?genus=Epicrates&species=maurus&search_param=%28%28common_name%3D%27Epicrates+cenchria+maurus%27%29%29
42	64	Rainbow boa	Epicrates maurus	Squamata	Snake	USA	America	CB	https://reptile-database.reptarium.cz/species?genus=Epicrates&species=maurus&search_param=%28%28common_name%3D%27Epicrates+cenchria+maurus%27%29%29
42	64	Rainbow boa	Epicrates maurus	Squamata	Snake	USA	America	CB	https://reptile-database.reptarium.cz/species?genus=Epicrates&species=maurus&search_param=%28%28common_name%3D%27Epicrates+cenchria+maurus%27%29%29
42	64	Rainbow boa	Epicrates maurus	Squamata	Snake	USA	America	CB	https://reptile-database.reptarium.cz/species?genus=Epicrates&species=maurus&search_param=%28%28common_name%3D%27Epicrates+cenchria+maurus%27%29%29
42	64	Rainbow boa	Epicrates maurus	Squamata	Snake	USA	America	CB	https://reptile-database.reptarium.cz/species?genus=Epicrates&species=maurus&search_param=%28%28common_name%3D%27Epicrates+cenchria+maurus%27%29%29
42	64	Rainbow boa	Epicrates maurus	Squamata	Snake	USA	America	CB	https://reptile-database.reptarium.cz/species?genus=Epicrates&species=maurus&search_param=%28%28common_name%3D%27Epicrates+cenchria+maurus%27%29%29
42	64	Rainbow boa	Epicrates maurus	Squamata	Snake	USA	America	CB	https://reptile-database.reptarium.cz/species?genus=Epicrates&species=maurus&search_param=%28%28common_name%3D%27Epicrates+cenchria+maurus%27%29%29
43	65	Horsefield's tortoise	Testudo horsfieldii	Testudines	Turtle	Uzbekistan	Asia	FB	https://reptile-database.reptarium.cz/species?genus=Testudo&species=horsfieldii&search_param=%28%28common_name%3D%27Testudo+horsfieldii%27%29%29
43	65	Horsefield's tortoise	Testudo horsfieldii	Testudines	Turtle	Uzbekistan	Asia	FB	https://reptile-database.reptarium.cz/species?genus=Testudo&species=horsfieldii&search_param=%28%28common_name%3D%27Testudo+horsfieldii%27%29%29
43	65	Horsefield's tortoise	Testudo horsfieldii	Testudines	Turtle	Uzbekistan	Asia	FB	https://reptile-database.reptarium.cz/species?genus=Testudo&species=horsfieldii&search_param=%28%28common_name%3D%27Testudo+horsfieldii%27%29%29
43	65	Horsefield's tortoise	Testudo horsfieldii	Testudines	Turtle	Uzbekistan	Asia	FB	https://reptile-database.reptarium.cz/species?genus=Testudo&species=horsfieldii&search_param=%28%28common_name%3D%27Testudo+horsfieldii%27%29%29
43	65	Horsefield's tortoise	Testudo horsfieldii	Testudines	Turtle	Uzbekistan	Asia	FB	https://reptile-database.reptarium.cz/species?genus=Testudo&species=horsfieldii&search_param=%28%28common_name%3D%27Testudo+horsfieldii%27%29%29

Table S1: Animal species sampled in this study - country origin and categorization as captive bred (CB), farm bred (FB) and wild-caught (WC)

Shipment No.	Sample batch	Animal species	Scientific name of animal/s	Order	Suborder	Country origin	Continent	Captive bred (CB), farm bred (FB), wild-caught (WC)	Web URL used for assigning animal species to categories CB, FB and WC
43	65	Horsefield's tortoise	Testudo horsfieldii	Testudines	Turtle	Uzbekistan	Asia	FB	https://reptile-database.reptarium.cz/species?genus=Testudo&species=horsfieldii&search_param=%28%28common_name%3D%27Testudo+horsfieldii%27%29%29
43	65	Horsefield's tortoise	Testudo horsfieldii	Testudines	Turtle	Uzbekistan	Asia	FB	https://reptile-database.reptarium.cz/species?genus=Testudo&species=horsfieldii&search_param=%28%28common_name%3D%27Testudo+horsfieldii%27%29%29
43	65	Horsefield's tortoise	Testudo horsfieldii	Testudines	Turtle	Uzbekistan	Asia	FB	https://reptile-database.reptarium.cz/species?genus=Testudo&species=horsfieldii&search_param=%28%28common_name%3D%27Testudo+horsfieldii%27%29%29
43	65	Horsefield's tortoise	Testudo horsfieldii	Testudines	Turtle	Uzbekistan	Asia	FB	https://reptile-database.reptarium.cz/species?genus=Testudo&species=horsfieldii&search_param=%28%28common_name%3D%27Testudo+horsfieldii%27%29%29
43	65	Horsefield's tortoise	Testudo horsfieldii	Testudines	Turtle	Uzbekistan	Asia	FB	https://reptile-database.reptarium.cz/species?genus=Testudo&species=horsfieldii&search_param=%28%28common_name%3D%27Testudo+horsfieldii%27%29%29
44	66	Central bearded dragon	Pogona vitticeps	Squamata	Lizard	Ukraine	Europe	CB	https://reptile-database.reptarium.cz/species?genus=Pogona&species=vitticeps&search_param=%28%28common_name%3D%27Pogona+vitticeps%27%29%29
44	66	Central bearded dragon	Pogona vitticeps	Squamata	Lizard	Ukraine	Europe	CB	https://reptile-database.reptarium.cz/species?genus=Pogona&species=vitticeps&search_param=%28%28common_name%3D%27Pogona+vitticeps%27%29%29
44	66	Central bearded dragon	Pogona vitticeps	Squamata	Lizard	Ukraine	Europe	CB	https://reptile-database.reptarium.cz/species?genus=Pogona&species=vitticeps&search_param=%28%28common_name%3D%27Pogona+vitticeps%27%29%29
44	66	Central bearded dragon	Pogona vitticeps	Squamata	Lizard	Ukraine	Europe	CB	https://reptile-database.reptarium.cz/species?genus=Pogona&species=vitticeps&search_param=%28%28common_name%3D%27Pogona+vitticeps%27%29%29
44	66	Central bearded dragon	Pogona vitticeps	Squamata	Lizard	Ukraine	Europe	CB	https://reptile-database.reptarium.cz/species?genus=Pogona&species=vitticeps&search_param=%28%28common_name%3D%27Pogona+vitticeps%27%29%29
44	66	Central bearded dragon	Pogona vitticeps	Squamata	Lizard	Ukraine	Europe	CB	https://reptile-database.reptarium.cz/species?genus=Pogona&species=vitticeps&search_param=%28%28common_name%3D%27Pogona+vitticeps%27%29%29
44	66	Central bearded dragon	Pogona vitticeps	Squamata	Lizard	Ukraine	Europe	CB	https://reptile-database.reptarium.cz/species?genus=Pogona&species=vitticeps&search_param=%28%28common_name%3D%27Pogona+vitticeps%27%29%29

Table S1: Animal species sampled in this study - country origin and categorization as captive bred (CB), farm bred (FB) and wild-caught (WC)

Shipment No.	Sample batch	Animal species	Scientific name of animal/s	Order	Suborder	Country origin	Continent	Captive bred (CB), farm bred (FB), wild-caught (WC)	Web URL used for assigning animal species to categories CB, FB and WC
44	66	Central bearded dragon	Pogona vitticeps	Squamata	Lizard	Ukraine	Europe	CB	https://reptile-database.reptarium.cz/species?genus=Pogona&species=vitticeps&search_param=%28%28common_name%3D%27Pogona+vitticeps%27%29%29
44	66	Central bearded dragon	Pogona vitticeps	Squamata	Lizard	Ukraine	Europe	CB	https://reptile-database.reptarium.cz/species?genus=Pogona&species=vitticeps&search_param=%28%28common_name%3D%27Pogona+vitticeps%27%29%29
44	66	Central bearded dragon	Pogona vitticeps	Squamata	Lizard	Ukraine	Europe	CB	https://reptile-database.reptarium.cz/species?genus=Pogona&species=vitticeps&search_param=%28%28common_name%3D%27Pogona+vitticeps%27%29%29
44	66	Central bearded dragon	Pogona vitticeps	Squamata	Lizard	Ukraine	Europe	CB	https://reptile-database.reptarium.cz/species?genus=Pogona&species=vitticeps&search_param=%28%28common_name%3D%27Pogona+vitticeps%27%29%29
44	66	Central bearded dragon	Pogona vitticeps	Squamata	Lizard	Ukraine	Europe	CB	https://reptile-database.reptarium.cz/species?genus=Pogona&species=vitticeps&search_param=%28%28common_name%3D%27Pogona+vitticeps%27%29%29
44	66	Central bearded dragon	Pogona vitticeps	Squamata	Lizard	Ukraine	Europe	CB	https://reptile-database.reptarium.cz/species?genus=Pogona&species=vitticeps&search_param=%28%28common_name%3D%27Pogona+vitticeps%27%29%29
44	66	Central bearded dragon	Pogona vitticeps	Squamata	Lizard	Ukraine	Europe	CB	https://reptile-database.reptarium.cz/species?genus=Pogona&species=vitticeps&search_param=%28%28common_name%3D%27Pogona+vitticeps%27%29%29
44	66	Central bearded dragon	Pogona vitticeps	Squamata	Lizard	Ukraine	Europe	CB	https://reptile-database.reptarium.cz/species?genus=Pogona&species=vitticeps&search_param=%28%28common_name%3D%27Pogona+vitticeps%27%29%29
44	67	Madagascar day gecko	Phelsuma madagascariensis	Squamata	Lizard	Ukraine	Europe	CB	https://reptile-database.reptarium.cz/species?genus=Phelsuma&species=madagascariensis&search_param=%28%28common_name%3D%27Phelsuma+madagascariensis%27%29%29
44	67	Madagascar day gecko	Phelsuma madagascariensis	Squamata	Lizard	Ukraine	Europe	CB	https://reptile-database.reptarium.cz/species?genus=Phelsuma&species=madagascariensis&search_param=%28%28common_name%3D%27Phelsuma+madagascariensis%27%29%29
44	67	Madagascar day gecko	Phelsuma madagascariensis	Squamata	Lizard	Ukraine	Europe	CB	https://reptile-database.reptarium.cz/species?genus=Phelsuma&species=madagascariensis&search_param=%28%28common_name%3D%27Phelsuma+madagascariensis%27%29%29
44	67	Madagascar day gecko	Phelsuma madagascariensis	Squamata	Lizard	Ukraine	Europe	CB	https://reptile-database.reptarium.cz/species?genus=Phelsuma&species=madagascariensis&search_param=%28%28common_name%3D%27Phelsuma+madagascariensis%27%29%29

Table S1: Animal species sampled in this study - country origin and categorization as captive bred (CB), farm bred (FB) and wild-caught (WC)

Shipment No.	Sample batch	Animal species	Scientific name of animal/s	Order	Suborder	Country origin	Continent	Captive bred (CB), farm bred (FB), wild-caught (WC)	Web URL used for assigning animal species to categories CB, FB and WC
44	67	Madagascar day gecko	Phelsuma madagascariensis	Squamata	Lizard	Ukraine	Europe	CB	https://reptile-database.reptarium.cz/species?genus=Phelsuma&species=madagascariensis&search_param=%28%28common_name%3D%27Phelsuma+madagascariensis%27%29%29
44	67	Madagascar day gecko	Phelsuma madagascariensis	Squamata	Lizard	Ukraine	Europe	CB	https://reptile-database.reptarium.cz/species?genus=Phelsuma&species=madagascariensis&search_param=%28%28common_name%3D%27Phelsuma+madagascariensis%27%29%29
44	67	Madagascar day gecko	Phelsuma madagascariensis	Squamata	Lizard	Ukraine	Europe	CB	https://reptile-database.reptarium.cz/species?genus=Phelsuma&species=madagascariensis&search_param=%28%28common_name%3D%27Phelsuma+madagascariensis%27%29%29
44	67	Madagascar day gecko	Phelsuma madagascariensis	Squamata	Lizard	Ukraine	Europe	CB	https://reptile-database.reptarium.cz/species?genus=Phelsuma&species=madagascariensis&search_param=%28%28common_name%3D%27Phelsuma+madagascariensis%27%29%29
44	67	Madagascar day gecko	Phelsuma madagascariensis	Squamata	Lizard	Ukraine	Europe	CB	https://reptile-database.reptarium.cz/species?genus=Phelsuma&species=madagascariensis&search_param=%28%28common_name%3D%27Phelsuma+madagascariensis%27%29%29
45	68	Ball python	Python regius	Squamata	Snake	USA	America	CB	https://reptile-database.reptarium.cz/species?genus=Python&species=regius&search_param=%28%28common_name%3D%27Python+regius%27%29%29
45	68	Ball python	Python regius	Squamata	Snake	USA	America	CB	https://reptile-database.reptarium.cz/species?genus=Python&species=regius&search_param=%28%28common_name%3D%27Python+regius%27%29%29
45	68	Ball python	Python regius	Squamata	Snake	USA	America	CB	https://reptile-database.reptarium.cz/species?genus=Python&species=regius&search_param=%28%28common_name%3D%27Python+regius%27%29%29
45	68	Ball python	Python regius	Squamata	Snake	USA	America	CB	https://reptile-database.reptarium.cz/species?genus=Python&species=regius&search_param=%28%28common_name%3D%27Python+regius%27%29%29
46	69	Horsefield's tortoise	Testudo horsfieldii	Testudines	Turtle	Uzbekistan	Asia	FB	https://reptile-database.reptarium.cz/species?genus=Testudo&species=horsfieldii&search_param=%28%28common_name%3D%27Testudo+horsfieldii%27%29%29
46	69	Horsefield's tortoise	Testudo horsfieldii	Testudines	Turtle	Uzbekistan	Asia	FB	https://reptile-database.reptarium.cz/species?genus=Testudo&species=horsfieldii&search_param=%28%28common_name%3D%27Testudo+horsfieldii%27%29%29
46	69	Horsefield's tortoise	Testudo horsfieldii	Testudines	Turtle	Uzbekistan	Asia	FB	https://reptile-database.reptarium.cz/species?genus=Testudo&species=horsfieldii&search_param=%28%28common_name%3D%27Testudo+horsfieldii%27%29%29

Table S1: Animal species sampled in this study - country origin and categorization as captive bred (CB), farm bred (FB) and wild-caught (WC)

Shipment No.	Sample batch	Animal species	Scientific name of animal/s	Order	Suborder	Country origin	Continent	Captive bred (CB), farm bred (FB), wild-caught (WC)	Web URL used for assigning animal species to categories CB, FB and WC
46	69	Horsefield's tortoise	Testudo horsfieldii	Testudines	Turtle	Uzbekistan	Asia	FB	https://reptile-database.reptarium.cz/species?genus=Testudo&species=horsfieldii&search_param=%28%28common_name%3D%27Testudo+horsfieldii%27%29%29
46	69	Horsefield's tortoise	Testudo horsfieldii	Testudines	Turtle	Uzbekistan	Asia	FB	https://reptile-database.reptarium.cz/species?genus=Testudo&species=horsfieldii&search_param=%28%28common_name%3D%27Testudo+horsfieldii%27%29%29
46	69	Horsefield's tortoise	Testudo horsfieldii	Testudines	Turtle	Uzbekistan	Asia	FB	https://reptile-database.reptarium.cz/species?genus=Testudo&species=horsfieldii&search_param=%28%28common_name%3D%27Testudo+horsfieldii%27%29%29
46	69	Horsefield's tortoise	Testudo horsfieldii	Testudines	Turtle	Uzbekistan	Asia	FB	https://reptile-database.reptarium.cz/species?genus=Testudo&species=horsfieldii&search_param=%28%28common_name%3D%27Testudo+horsfieldii%27%29%29
46	69	Horsefield's tortoise	Testudo horsfieldii	Testudines	Turtle	Uzbekistan	Asia	FB	https://reptile-database.reptarium.cz/species?genus=Testudo&species=horsfieldii&search_param=%28%28common_name%3D%27Testudo+horsfieldii%27%29%29
46	69	Horsefield's tortoise	Testudo horsfieldii	Testudines	Turtle	Uzbekistan	Asia	FB	https://reptile-database.reptarium.cz/species?genus=Testudo&species=horsfieldii&search_param=%28%28common_name%3D%27Testudo+horsfieldii%27%29%29
46	69	Horsefield's tortoise	Testudo horsfieldii	Testudines	Turtle	Uzbekistan	Asia	FB	https://reptile-database.reptarium.cz/species?genus=Testudo&species=horsfieldii&search_param=%28%28common_name%3D%27Testudo+horsfieldii%27%29%29
46	69	Horsefield's tortoise	Testudo horsfieldii	Testudines	Turtle	Uzbekistan	Asia	FB	https://reptile-database.reptarium.cz/species?genus=Testudo&species=horsfieldii&search_param=%28%28common_name%3D%27Testudo+horsfieldii%27%29%29
46	69	Horsefield's tortoise	Testudo horsfieldii	Testudines	Turtle	Uzbekistan	Asia	FB	https://reptile-database.reptarium.cz/species?genus=Testudo&species=horsfieldii&search_param=%28%28common_name%3D%27Testudo+horsfieldii%27%29%29
47	70	Garter snake	Thamnophis sp.	Squamata	Snake	USA	America	WC/FB	https://reptile-database.reptarium.cz/advanced_search?common_name=Thamnophis&submit=Search
47	70	Garter snake	Thamnophis sp.	Squamata	Snake	USA	America	WC/FB	https://reptile-database.reptarium.cz/advanced_search?common_name=Thamnophis&submit=Search
47	70	Garter snake	Thamnophis sp.	Squamata	Snake	USA	America	WC/FB	https://reptile-database.reptarium.cz/advanced_search?common_name=Thamnophis&submit=Search
47	70	Garter snake	Thamnophis sp.	Squamata	Snake	USA	America	WC/FB	https://reptile-database.reptarium.cz/advanced_search?common_name=Thamnophis&submit=Search

Table S1: Animal species sampled in this study - country origin and categorization as captive bred (CB), farm bred (FB) and wild-caught (WC)

Shipment No.	Sample batch	Animal species	Scientific name of animal/s	Order	Suborder	Country origin	Continent	Captive bred (CB), farm bred (FB), wild-caught (WC)	Web URL used for assigning animal species to categories CB, FB and WC
47	70	Garter snake	Thamnophis sp.	Squamata	Snake	USA	America	WC/FB	https://reptile-database.reptarium.cz/advanced_search?common_name=Thamnophis&submit=Search
47	70	Garter snake	Thamnophis sp.	Squamata	Snake	USA	America	WC/FB	https://reptile-database.reptarium.cz/advanced_search?common_name=Thamnophis&submit=Search
47	70	Garter snake	Thamnophis sp.	Squamata	Snake	USA	America	WC/FB	https://reptile-database.reptarium.cz/advanced_search?common_name=Thamnophis&submit=Search
47	71	Green anole	Anolis carolinensis	Squamata	Lizard	USA	America	WC/FB	https://reptile-database.reptarium.cz/species?genus=Anolis&species=carolinensis&search_param=%28%28common_name%3D%27Anolis+carolinensis%27%29%29
47	71	Green anole	Anolis carolinensis	Squamata	Lizard	USA	America	WC/FB	https://reptile-database.reptarium.cz/species?genus=Anolis&species=carolinensis&search_param=%28%28common_name%3D%27Anolis+carolinensis%27%29%29
47	71	Green anole	Anolis carolinensis	Squamata	Lizard	USA	America	WC/FB	https://reptile-database.reptarium.cz/species?genus=Anolis&species=carolinensis&search_param=%28%28common_name%3D%27Anolis+carolinensis%27%29%29
47	71	Green anole	Anolis carolinensis	Squamata	Lizard	USA	America	WC/FB	https://reptile-database.reptarium.cz/species?genus=Anolis&species=carolinensis&search_param=%28%28common_name%3D%27Anolis+carolinensis%27%29%29
47	71	Green anole	Anolis carolinensis	Squamata	Lizard	USA	America	WC/FB	https://reptile-database.reptarium.cz/species?genus=Anolis&species=carolinensis&search_param=%28%28common_name%3D%27Anolis+carolinensis%27%29%29
47	71	Green anole	Anolis carolinensis	Squamata	Lizard	USA	America	WC/FB	https://reptile-database.reptarium.cz/species?genus=Anolis&species=carolinensis&search_param=%28%28common_name%3D%27Anolis+carolinensis%27%29%29
47	71	Green anole	Anolis carolinensis	Squamata	Lizard	USA	America	WC/FB	https://reptile-database.reptarium.cz/species?genus=Anolis&species=carolinensis&search_param=%28%28common_name%3D%27Anolis+carolinensis%27%29%29
47	71	Green anole	Anolis carolinensis	Squamata	Lizard	USA	America	WC/FB	https://reptile-database.reptarium.cz/species?genus=Anolis&species=carolinensis&search_param=%28%28common_name%3D%27Anolis+carolinensis%27%29%29
47	71	Green anole	Anolis carolinensis	Squamata	Lizard	USA	America	WC/FB	https://reptile-database.reptarium.cz/species?genus=Anolis&species=carolinensis&search_param=%28%28common_name%3D%27Anolis+carolinensis%27%29%29
47	71	Green anole	Anolis carolinensis	Squamata	Lizard	USA	America	WC/FB	https://reptile-database.reptarium.cz/species?genus=Anolis&species=carolinensis&search_param=%28%28common_name%3D%27Anolis+carolinensis%27%29%29

Table S1: Animal species sampled in this study - country origin and categorization as captive bred (CB), farm bred (FB) and wild-caught (WC)

Shipment No.	Sample batch	Animal species	Scientific name of animal/s	Order	Suborder	Country origin	Continent	Captive bred (CB), farm bred (FB), wild-caught (WC)	Web URL used for assigning animal species to categories CB, FB and WC
48	72	Yellow mud turtle	Kinosternon flavescens	Testudines	Turtle	USA	America	FB	https://reptile-database.reptarium.cz/species?genus=Kinosternon&species=flavescens&search_param=%28%28common_name%3D%27Kinosternon+flavescens%27%29%29
48	72	Yellow mud turtle	Kinosternon flavescens	Testudines	Turtle	USA	America	FB	https://reptile-database.reptarium.cz/species?genus=Kinosternon&species=flavescens&search_param=%28%28common_name%3D%27Kinosternon+flavescens%27%29%29
48	72	Yellow mud turtle	Kinosternon flavescens	Testudines	Turtle	USA	America	FB	https://reptile-database.reptarium.cz/species?genus=Kinosternon&species=flavescens&search_param=%28%28common_name%3D%27Kinosternon+flavescens%27%29%29
48	72	Yellow mud turtle	Kinosternon flavescens	Testudines	Turtle	USA	America	FB	https://reptile-database.reptarium.cz/species?genus=Kinosternon&species=flavescens&search_param=%28%28common_name%3D%27Kinosternon+flavescens%27%29%29
48	72	Yellow mud turtle	Kinosternon flavescens	Testudines	Turtle	USA	America	FB	https://reptile-database.reptarium.cz/species?genus=Kinosternon&species=flavescens&search_param=%28%28common_name%3D%27Kinosternon+flavescens%27%29%29
48	72	Yellow mud turtle	Kinosternon flavescens	Testudines	Turtle	USA	America	FB	https://reptile-database.reptarium.cz/species?genus=Kinosternon&species=flavescens&search_param=%28%28common_name%3D%27Kinosternon+flavescens%27%29%29
48	72	Yellow mud turtle	Kinosternon flavescens	Testudines	Turtle	USA	America	FB	https://reptile-database.reptarium.cz/species?genus=Kinosternon&species=flavescens&search_param=%28%28common_name%3D%27Kinosternon+flavescens%27%29%29
48	72	Yellow mud turtle	Kinosternon flavescens	Testudines	Turtle	USA	America	FB	https://reptile-database.reptarium.cz/species?genus=Kinosternon&species=flavescens&search_param=%28%28common_name%3D%27Kinosternon+flavescens%27%29%29
48	72	Yellow mud turtle	Kinosternon flavescens	Testudines	Turtle	USA	America	FB	https://reptile-database.reptarium.cz/species?genus=Kinosternon&species=flavescens&search_param=%28%28common_name%3D%27Kinosternon+flavescens%27%29%29
48	72	Yellow mud turtle	Kinosternon flavescens	Testudines	Turtle	USA	America	FB	https://reptile-database.reptarium.cz/species?genus=Kinosternon&species=flavescens&search_param=%28%28common_name%3D%27Kinosternon+flavescens%27%29%29
48	73	Yellow-bellied slider	Trachemys scripta scripta	Testudines	Turtle	USA	America	FB	https://reptile-database.reptarium.cz/species?genus=Trachemys&species=scripta&search_param=%28%28common_name%3D%27Trachemys+scripta+scripta%27%29%29
48	73	Yellow-bellied slider	Trachemys scripta scripta	Testudines	Turtle	USA	America	FB	https://reptile-database.reptarium.cz/species?genus=Trachemys&species=scripta&search_param=%28%28common_name%3D%27Trachemys+scripta+scripta%27%29%29

Table S1: Animal species sampled in this study - country origin and categorization as captive bred (CB), farm bred (FB) and wild-caught (WC)

Shipment No.	Sample batch	Animal species	Scientific name of animal/s	Order	Suborder	Country origin	Continent	Captive bred (CB), farm bred (FB), wild-caught (WC)	Web URL used for assigning animal species to categories CB, FB and WC
48	73	Yellow-bellied slider	Trachemys scripta scripta	Testudines	Turtle	USA	America	FB	https://reptile-database.reptarium.cz/species?genus=Trachemys&species=scripta&search_param=%28%28common_name%3D%27Trachemys+scripta+scripta%27%29%29
48	73	Yellow-bellied slider	Trachemys scripta scripta	Testudines	Turtle	USA	America	FB	https://reptile-database.reptarium.cz/species?genus=Trachemys&species=scripta&search_param=%28%28common_name%3D%27Trachemys+scripta+scripta%27%29%29
48	73	Yellow-bellied slider	Trachemys scripta scripta	Testudines	Turtle	USA	America	FB	https://reptile-database.reptarium.cz/species?genus=Trachemys&species=scripta&search_param=%28%28common_name%3D%27Trachemys+scripta+scripta%27%29%29
48	73	Yellow-bellied slider	Trachemys scripta scripta	Testudines	Turtle	USA	America	FB	https://reptile-database.reptarium.cz/species?genus=Trachemys&species=scripta&search_param=%28%28common_name%3D%27Trachemys+scripta+scripta%27%29%29
48	73	Yellow-bellied slider	Trachemys scripta scripta	Testudines	Turtle	USA	America	FB	https://reptile-database.reptarium.cz/species?genus=Trachemys&species=scripta&search_param=%28%28common_name%3D%27Trachemys+scripta+scripta%27%29%29
49	74	Parson's chameleon	Calumma parsonii	Squamata	Lizard	USA	America	CB	https://reptile-database.reptarium.cz/species?genus=Calumma&species=parsonii&search_param=%28%28common_name%3D%27Calumma+parsonii%27%29%29
49	74	Parson's chameleon	Calumma parsonii	Squamata	Lizard	USA	America	CB	https://reptile-database.reptarium.cz/species?genus=Calumma&species=parsonii&search_param=%28%28common_name%3D%27Calumma+parsonii%27%29%29
49	74	Parson's chameleon	Calumma parsonii	Squamata	Lizard	USA	America	CB	https://reptile-database.reptarium.cz/species?genus=Calumma&species=parsonii&search_param=%28%28common_name%3D%27Calumma+parsonii%27%29%29
49	74	Parson's chameleon	Calumma parsonii	Squamata	Lizard	USA	America	CB	https://reptile-database.reptarium.cz/species?genus=Calumma&species=parsonii&search_param=%28%28common_name%3D%27Calumma+parsonii%27%29%29
49	74	Parson's chameleon	Calumma parsonii	Squamata	Lizard	USA	America	CB	https://reptile-database.reptarium.cz/species?genus=Calumma&species=parsonii&search_param=%28%28common_name%3D%27Calumma+parsonii%27%29%29
49	74	Parson's chameleon	Calumma parsonii	Squamata	Lizard	USA	America	CB	https://reptile-database.reptarium.cz/species?genus=Calumma&species=parsonii&search_param=%28%28common_name%3D%27Calumma+parsonii%27%29%29
49	74	Parson's chameleon	Calumma parsonii	Squamata	Lizard	USA	America	CB	https://reptile-database.reptarium.cz/species?genus=Calumma&species=parsonii&search_param=%28%28common_name%3D%27Calumma+parsonii%27%29%29

Table S1: Animal species sampled in this study - country origin and categorization as captive bred (CB), farm bred (FB) and wild-caught (WC)

Shipment No.	Sample batch	Animal species	Scientific name of animal/s	Order	Suborder	Country origin	Continent	Captive bred (CB), farm bred (FB), wild-caught (WC)	Web URL used for assigning animal species to categories CB, FB and WC
49	74	Parson's chameleon	Calumma parsonii	Squamata	Lizard	USA	America	CB	https://reptile-database.reptarium.cz/species?genus=Calumma&species=parsonii&search_param=%28%28common_name%3D%27Calumma+parsonii%27%29%29
49	74	Parson's chameleon	Calumma parsonii	Squamata	Lizard	USA	America	CB	https://reptile-database.reptarium.cz/species?genus=Calumma&species=parsonii&search_param=%28%28common_name%3D%27Calumma+parsonii%27%29%29
49	74	Parson's chameleon	Calumma parsonii	Squamata	Lizard	USA	America	CB	https://reptile-database.reptarium.cz/species?genus=Calumma&species=parsonii&search_param=%28%28common_name%3D%27Calumma+parsonii%27%29%29
49	74	Parson's chameleon	Calumma parsonii	Squamata	Lizard	USA	America	CB	https://reptile-database.reptarium.cz/species?genus=Calumma&species=parsonii&search_param=%28%28common_name%3D%27Calumma+parsonii%27%29%29
49	74	Parson's chameleon	Calumma parsonii	Squamata	Lizard	USA	America	CB	https://reptile-database.reptarium.cz/species?genus=Calumma&species=parsonii&search_param=%28%28common_name%3D%27Calumma+parsonii%27%29%29
49	74	Parson's chameleon	Calumma parsonii	Squamata	Lizard	USA	America	CB	https://reptile-database.reptarium.cz/species?genus=Calumma&species=parsonii&search_param=%28%28common_name%3D%27Calumma+parsonii%27%29%29
49	74	Parson's chameleon	Calumma parsonii	Squamata	Lizard	USA	America	CB	https://reptile-database.reptarium.cz/species?genus=Calumma&species=parsonii&search_param=%28%28common_name%3D%27Calumma+parsonii%27%29%29
50	75	Sand boa	Eryx colubrinus	Squamata	Snake	USA	America	CB	https://reptile-database.reptarium.cz/species?genus=Eryx&species=colubrinus&search_param=%28%28common_name%3D%27Eryx+colubrinus%27%29%29
50	75	Sand boa	Eryx colubrinus	Squamata	Snake	USA	America	CB	https://reptile-database.reptarium.cz/species?genus=Eryx&species=colubrinus&search_param=%28%28common_name%3D%27Eryx+colubrinus%27%29%29
50	75	Sand boa	Eryx colubrinus	Squamata	Snake	USA	America	CB	https://reptile-database.reptarium.cz/species?genus=Eryx&species=colubrinus&search_param=%28%28common_name%3D%27Eryx+colubrinus%27%29%29
50	75	Sand boa	Eryx colubrinus	Squamata	Snake	USA	America	CB	https://reptile-database.reptarium.cz/species?genus=Eryx&species=colubrinus&search_param=%28%28common_name%3D%27Eryx+colubrinus%27%29%29
50	75	Sand boa	Eryx colubrinus	Squamata	Snake	USA	America	CB	https://reptile-database.reptarium.cz/species?genus=Eryx&species=colubrinus&search_param=%28%28common_name%3D%27Eryx+colubrinus%27%29%29

Table S1: Animal species sampled in this study - country origin and categorization as captive bred (CB), farm bred (FB) and wild-caught (WC)

Shipment No.	Sample batch	Animal species	Scientific name of animal/s	Order	Suborder	Country origin	Continent	Captive bred (CB), farm bred (FB), wild-caught (WC)	Web URL used for assigning animal species to categories CB, FB and WC
50	75	Sand boa	Eryx colubrinus	Squamata	Snake	USA	America	CB	https://reptile-database.reptarium.cz/species?genus=Eryx&species=colubrinus&search_param=%28%28common_name%3D%27Eryx+colubrinus%27%29%29
50	75	Sand boa	Eryx colubrinus	Squamata	Snake	USA	America	CB	https://reptile-database.reptarium.cz/species?genus=Eryx&species=colubrinus&search_param=%28%28common_name%3D%27Eryx+colubrinus%27%29%29
50	75	Sand boa	Eryx colubrinus	Squamata	Snake	USA	America	CB	https://reptile-database.reptarium.cz/species?genus=Eryx&species=colubrinus&search_param=%28%28common_name%3D%27Eryx+colubrinus%27%29%29
50	75	Sand boa	Eryx colubrinus	Squamata	Snake	USA	America	CB	https://reptile-database.reptarium.cz/species?genus=Eryx&species=colubrinus&search_param=%28%28common_name%3D%27Eryx+colubrinus%27%29%29
50	76	Indigo snake	Drymarchon corais (D.couperi?)	Squamata	Snake	USA	America	CB	https://reptile-database.reptarium.cz/species?genus=Drymarchon&species=corais&search_param=%28%28common_name%3D%27Drymarchon+corais%27%29%29
50	76	Indigo snake	Drymarchon corais (D.couperi?)	Squamata	Snake	USA	America	CB	https://reptile-database.reptarium.cz/species?genus=Drymarchon&species=corais&search_param=%28%28common_name%3D%27Drymarchon+corais%27%29%29
50	76	Indigo snake	Drymarchon corais (D.couperi?)	Squamata	Snake	USA	America	CB	https://reptile-database.reptarium.cz/species?genus=Drymarchon&species=corais&search_param=%28%28common_name%3D%27Drymarchon+corais%27%29%29
50	76	Indigo snake	Drymarchon corais (D.couperi?)	Squamata	Snake	USA	America	CB	https://reptile-database.reptarium.cz/species?genus=Drymarchon&species=corais&search_param=%28%28common_name%3D%27Drymarchon+corais%27%29%29
50	76	Indigo snake	Drymarchon corais (D.couperi?)	Squamata	Snake	USA	America	CB	https://reptile-database.reptarium.cz/species?genus=Drymarchon&species=corais&search_param=%28%28common_name%3D%27Drymarchon+corais%27%29%29
50	76	Indigo snake	Drymarchon corais (D.couperi?)	Squamata	Snake	USA	America	CB	https://reptile-database.reptarium.cz/species?genus=Drymarchon&species=corais&search_param=%28%28common_name%3D%27Drymarchon+corais%27%29%29
50	76	Indigo snake	Drymarchon corais (D.couperi?)	Squamata	Snake	USA	America	CB	https://reptile-database.reptarium.cz/species?genus=Drymarchon&species=corais&search_param=%28%28common_name%3D%27Drymarchon+corais%27%29%29
50	76	Indigo snake	Drymarchon corais (D.couperi?)	Squamata	Snake	USA	America	CB	https://reptile-database.reptarium.cz/species?genus=Drymarchon&species=corais&search_param=%28%28common_name%3D%27Drymarchon+corais%27%29%29

Table S1: Animal species sampled in this study - country origin and categorization as captive bred (CB), farm bred (FB) and wild-caught (WC)

Shipment No.	Sample batch	Animal species	Scientific name of animal/s	Order	Suborder	Country origin	Continent	Captive bred (CB), farm bred (FB), wild-caught (WC)	Web URL used for assigning animal species to categories CB, FB and WC
50	76	Indigo snake	Drymarchon corais (D.couperi?)	Squamata	Snake	USA	America	CB	https://reptile-database.reptarium.cz/species?genus=Drymarchon&species=corais&search_param=%28%28common_name%3D%27Drymarchon+corais%27%29%29
50	76	Indigo snake	Drymarchon corais (D.couperi?)	Squamata	Snake	USA	America	CB	https://reptile-database.reptarium.cz/species?genus=Drymarchon&species=corais&search_param=%28%28common_name%3D%27Drymarchon+corais%27%29%29
50	76	Indigo snake	Drymarchon corais (D.couperi?)	Squamata	Snake	USA	America	CB	https://reptile-database.reptarium.cz/species?genus=Drymarchon&species=corais&search_param=%28%28common_name%3D%27Drymarchon+corais%27%29%29
50	76	Indigo snake	Drymarchon corais (D.couperi?)	Squamata	Snake	USA	America	CB	https://reptile-database.reptarium.cz/species?genus=Drymarchon&species=corais&search_param=%28%28common_name%3D%27Drymarchon+corais%27%29%29
50	76	Indigo snake	Drymarchon corais (D.couperi?)	Squamata	Snake	USA	America	CB	https://reptile-database.reptarium.cz/species?genus=Drymarchon&species=corais&search_param=%28%28common_name%3D%27Drymarchon+corais%27%29%29
50	76	Indigo snake	Drymarchon corais (D.couperi?)	Squamata	Snake	USA	America	CB	https://reptile-database.reptarium.cz/species?genus=Drymarchon&species=corais&search_param=%28%28common_name%3D%27Drymarchon+corais%27%29%29
50	77	Fat-tail gecko	Hemitheconyx caudicinctus	Squamata	Lizard	USA	America	CB	https://reptile-database.reptarium.cz/species?genus=Hemitheconyx&species=caudicinctus&search_param=%28%28common_name%3D%27Hemitheconyx+caudicinctus%27%29%29
50	77	Fat-tail gecko	Hemitheconyx caudicinctus	Squamata	Lizard	USA	America	CB	https://reptile-database.reptarium.cz/species?genus=Hemitheconyx&species=caudicinctus&search_param=%28%28common_name%3D%27Hemitheconyx+caudicinctus%27%29%29
50	77	Fat-tail gecko	Hemitheconyx caudicinctus	Squamata	Lizard	USA	America	CB	https://reptile-database.reptarium.cz/species?genus=Hemitheconyx&species=caudicinctus&search_param=%28%28common_name%3D%27Hemitheconyx+caudicinctus%27%29%29
50	77	Fat-tail gecko	Hemitheconyx caudicinctus	Squamata	Lizard	USA	America	CB	https://reptile-database.reptarium.cz/species?genus=Hemitheconyx&species=caudicinctus&search_param=%28%28common_name%3D%27Hemitheconyx+caudicinctus%27%29%29
50	77	Fat-tail gecko	Hemitheconyx caudicinctus	Squamata	Lizard	USA	America	CB	https://reptile-database.reptarium.cz/species?genus=Hemitheconyx&species=caudicinctus&search_param=%28%28common_name%3D%27Hemitheconyx+caudicinctus%27%29%29
50	77	Fat-tail gecko	Hemitheconyx caudicinctus	Squamata	Lizard	USA	America	CB	https://reptile-database.reptarium.cz/species?genus=Hemitheconyx&species=caudicinctus&search_param=%28%28common_name%3D%27Hemitheconyx+caudicinctus%27%29%29

Table S1: Animal species sampled in this study - country origin and categorization as captive bred (CB), farm bred (FB) and wild-caught (WC)

Shipment No.	Sample batch	Animal species	Scientific name of animal/s	Order	Suborder	Country origin	Continent	Captive bred (CB), farm bred (FB), wild-caught (WC)	Web URL used for assigning animal species to categories CB, FB and WC
50	77	Fat-tail gecko	Hemitheconyx caudicinctus	Squamata	Lizard	USA	America	CB	https://reptile-database.reptarium.cz/species?genus=Hemitheconyx&species=caudicinctus&search_param=%28%28common_name%3D%27Hemitheconyx+caudicinctus%27%29%29
50	77	Fat-tail gecko	Hemitheconyx caudicinctus	Squamata	Lizard	USA	America	CB	https://reptile-database.reptarium.cz/species?genus=Hemitheconyx&species=caudicinctus&search_param=%28%28common_name%3D%27Hemitheconyx+caudicinctus%27%29%29
50	77	Fat-tail gecko	Hemitheconyx caudicinctus	Squamata	Lizard	USA	America	CB	https://reptile-database.reptarium.cz/species?genus=Hemitheconyx&species=caudicinctus&search_param=%28%28common_name%3D%27Hemitheconyx+caudicinctus%27%29%29
50	77	Fat-tail gecko	Hemitheconyx caudicinctus	Squamata	Lizard	USA	America	CB	https://reptile-database.reptarium.cz/species?genus=Hemitheconyx&species=caudicinctus&search_param=%28%28common_name%3D%27Hemitheconyx+caudicinctus%27%29%29
50	77	Fat-tail gecko	Hemitheconyx caudicinctus	Squamata	Lizard	USA	America	CB	https://reptile-database.reptarium.cz/species?genus=Hemitheconyx&species=caudicinctus&search_param=%28%28common_name%3D%27Hemitheconyx+caudicinctus%27%29%29
50	77	Fat-tail gecko	Hemitheconyx caudicinctus	Squamata	Lizard	USA	America	CB	https://reptile-database.reptarium.cz/species?genus=Hemitheconyx&species=caudicinctus&search_param=%28%28common_name%3D%27Hemitheconyx+caudicinctus%27%29%29
50	77	Fat-tail gecko	Hemitheconyx caudicinctus	Squamata	Lizard	USA	America	CB	https://reptile-database.reptarium.cz/species?genus=Hemitheconyx&species=caudicinctus&search_param=%28%28common_name%3D%27Hemitheconyx+caudicinctus%27%29%29
50	78	Chicken snake	Spilotes pullatus	Squamata	Snake	USA	America	CB	https://reptile-database.reptarium.cz/species?genus=Spilotes&species=pullatus&search_param=%28%28common_name%3D%27Spilotes+pullatus%27%29%29
50	78	Chicken snake	Spilotes pullatus	Squamata	Snake	USA	America	CB	https://reptile-database.reptarium.cz/species?genus=Spilotes&species=pullatus&search_param=%28%28common_name%3D%27Spilotes+pullatus%27%29%29
50	78	Chicken snake	Spilotes pullatus	Squamata	Snake	USA	America	CB	https://reptile-database.reptarium.cz/species?genus=Spilotes&species=pullatus&search_param=%28%28common_name%3D%27Spilotes+pullatus%27%29%29
50	78	Chicken snake	Spilotes pullatus	Squamata	Snake	USA	America	CB	https://reptile-database.reptarium.cz/species?genus=Spilotes&species=pullatus&search_param=%28%28common_name%3D%27Spilotes+pullatus%27%29%29
50	78	Chicken snake	Spilotes pullatus	Squamata	Snake	USA	America	CB	https://reptile-database.reptarium.cz/species?genus=Spilotes&species=pullatus&search_param=%28%28common_name%3D%27Spilotes+pullatus%27%29%29

Table S1: Animal species sampled in this study - country origin and categorization as captive bred (CB), farm bred (FB) and wild-caught (WC)

Shipment No.	Sample batch	Animal species	Scientific name of animal/s	Order	Suborder	Country origin	Continent	Captive bred (CB), farm bred (FB), wild-caught (WC)	Web URL used for assigning animal species to categories CB, FB and WC
50	78	Chicken snake	Spilotes pullatus	Squamata	Snake	USA	America	CB	https://reptile-database.reptarium.cz/species?genus=Spilotes&species=pullatus&search_param=%28%28common_name%3D%27Spilotes+pullatus%27%29%29
50	78	Chicken snake	Spilotes pullatus	Squamata	Snake	USA	America	CB	https://reptile-database.reptarium.cz/species?genus=Spilotes&species=pullatus&search_param=%28%28common_name%3D%27Spilotes+pullatus%27%29%29
50	78	Chicken snake	Spilotes pullatus	Squamata	Snake	USA	America	CB	https://reptile-database.reptarium.cz/species?genus=Spilotes&species=pullatus&search_param=%28%28common_name%3D%27Spilotes+pullatus%27%29%29
50	78	Chicken snake	Spilotes pullatus	Squamata	Snake	USA	America	CB	https://reptile-database.reptarium.cz/species?genus=Spilotes&species=pullatus&search_param=%28%28common_name%3D%27Spilotes+pullatus%27%29%29
50	78	Chicken snake	Spilotes pullatus	Squamata	Snake	USA	America	CB	https://reptile-database.reptarium.cz/species?genus=Spilotes&species=pullatus&search_param=%28%28common_name%3D%27Spilotes+pullatus%27%29%29
50	78	Chicken snake	Spilotes pullatus	Squamata	Snake	USA	America	CB	https://reptile-database.reptarium.cz/species?genus=Spilotes&species=pullatus&search_param=%28%28common_name%3D%27Spilotes+pullatus%27%29%29
50	78	Chicken snake	Spilotes pullatus	Squamata	Snake	USA	America	CB	https://reptile-database.reptarium.cz/species?genus=Spilotes&species=pullatus&search_param=%28%28common_name%3D%27Spilotes+pullatus%27%29%29
50	78	Chicken snake	Spilotes pullatus	Squamata	Snake	USA	America	CB	https://reptile-database.reptarium.cz/species?genus=Spilotes&species=pullatus&search_param=%28%28common_name%3D%27Spilotes+pullatus%27%29%29
51	79	Green spiny lizard	Sceloporus malachiticus	Squamata	Lizard	USA	America	presumably CB	https://reptile-database.reptarium.cz/species?genus=Sceloporus&species=malachiticus&search_param=%28%28common_name%3D%27Sceloporus+malachiticus%27%29%29
51	79	Green spiny lizard	Sceloporus malachiticus	Squamata	Lizard	USA	America	presumably CB	https://reptile-database.reptarium.cz/species?genus=Sceloporus&species=malachiticus&search_param=%28%28common_name%3D%27Sceloporus+malachiticus%27%29%29
51	79	Green spiny lizard	Sceloporus malachiticus	Squamata	Lizard	USA	America	presumably CB	https://reptile-database.reptarium.cz/species?genus=Sceloporus&species=malachiticus&search_param=%28%28common_name%3D%27Sceloporus+malachiticus%27%29%29
51	79	Green spiny lizard	Sceloporus malachiticus	Squamata	Lizard	USA	America	presumably CB	https://reptile-database.reptarium.cz/species?genus=Sceloporus&species=malachiticus&search_param=%28%28common_name%3D%27Sceloporus+malachiticus%27%29%29

Table S1: Animal species sampled in this study - country origin and categorization as captive bred (CB), farm bred (FB) and wild-caught (WC)

Shipment No.	Sample batch	Animal species	Scientific name of animal/s	Order	Suborder	Country origin	Continent	Captive bred (CB), farm bred (FB), wild-caught (WC)	Web URL used for assigning animal species to categories CB, FB and WC
51	79	Green spiny lizard	Sceloporus malachiticus	Squamata	Lizard	USA	America	presumably CB	https://reptile-database.reptarium.cz/species?genus=Sceloporus&species=malachiticus&search_param=%28%28common_name%3D%27Sceloporus+malachiticus%27%29%29
51	79	Green spiny lizard	Sceloporus malachiticus	Squamata	Lizard	USA	America	presumably CB	https://reptile-database.reptarium.cz/species?genus=Sceloporus&species=malachiticus&search_param=%28%28common_name%3D%27Sceloporus+malachiticus%27%29%29
51	79	Green spiny lizard	Sceloporus malachiticus	Squamata	Lizard	USA	America	presumably CB	https://reptile-database.reptarium.cz/species?genus=Sceloporus&species=malachiticus&search_param=%28%28common_name%3D%27Sceloporus+malachiticus%27%29%29
51	80	Rough greensnake	Opheodrys aestivus	Squamata	Snake	USA	America	WC	https://reptile-database.reptarium.cz/species?genus=Opheodrys&species=aestivus&search_param=%28%28common_name%3D%27Opheodrys+aestivus%27%29%29
51	80	Rough greensnake	Opheodrys aestivus	Squamata	Snake	USA	America	WC	https://reptile-database.reptarium.cz/species?genus=Opheodrys&species=aestivus&search_param=%28%28common_name%3D%27Opheodrys+aestivus%27%29%29
51	80	Rough greensnake	Opheodrys aestivus	Squamata	Snake	USA	America	WC	https://reptile-database.reptarium.cz/species?genus=Opheodrys&species=aestivus&search_param=%28%28common_name%3D%27Opheodrys+aestivus%27%29%29
51	80	Rough greensnake	Opheodrys aestivus	Squamata	Snake	USA	America	WC	https://reptile-database.reptarium.cz/species?genus=Opheodrys&species=aestivus&search_param=%28%28common_name%3D%27Opheodrys+aestivus%27%29%29
51	80	Rough greensnake	Opheodrys aestivus	Squamata	Snake	USA	America	WC	https://reptile-database.reptarium.cz/species?genus=Opheodrys&species=aestivus&search_param=%28%28common_name%3D%27Opheodrys+aestivus%27%29%29
51	80	Rough greensnake	Opheodrys aestivus	Squamata	Snake	USA	America	WC	https://reptile-database.reptarium.cz/species?genus=Opheodrys&species=aestivus&search_param=%28%28common_name%3D%27Opheodrys+aestivus%27%29%29
51	80	Rough greensnake	Opheodrys aestivus	Squamata	Snake	USA	America	WC	https://reptile-database.reptarium.cz/species?genus=Opheodrys&species=aestivus&search_param=%28%28common_name%3D%27Opheodrys+aestivus%27%29%29
51	80	Rough greensnake	Opheodrys aestivus	Squamata	Snake	USA	America	WC	https://reptile-database.reptarium.cz/species?genus=Opheodrys&species=aestivus&search_param=%28%28common_name%3D%27Opheodrys+aestivus%27%29%29
51	80	Rough greensnake	Opheodrys aestivus	Squamata	Snake	USA	America	WC	https://reptile-database.reptarium.cz/species?genus=Opheodrys&species=aestivus&search_param=%28%28common_name%3D%27Opheodrys+aestivus%27%29%29

Table S1: Animal species sampled in this study - country origin and categorization as captive bred (CB), farm bred (FB) and wild-caught (WC)

Shipment No.	Sample batch	Animal species	Scientific name of animal/s	Order	Suborder	Country origin	Continent	Captive bred (CB), farm bred (FB), wild-caught (WC)	Web URL used for assigning animal species to categories CB, FB and WC
51	80	Rough greensnake	Opheodrys aestivus	Squamata	Snake	USA	America	WC	https://reptile-database.reptarium.cz/species?genus=Opheodrys&species=aestivus&search_param=%28%28common_name%3D%27Opheodrys+aestivus%27%29%29
51	80	Rough greensnake	Opheodrys aestivus	Squamata	Snake	USA	America	WC	https://reptile-database.reptarium.cz/species?genus=Opheodrys&species=aestivus&search_param=%28%28common_name%3D%27Opheodrys+aestivus%27%29%29
52	81	Common green iguana	Iguana iguana	Squamata	Lizard	El Salvador	America	FB	https://reptile-database.reptarium.cz/species?genus=Iguana&species=iguana&search_param=%28%28common_name%3D%27Iguana+iguana%27%29%29
52	81	Common green iguana	Iguana iguana	Squamata	Lizard	El Salvador	America	FB	https://reptile-database.reptarium.cz/species?genus=Iguana&species=iguana&search_param=%28%28common_name%3D%27Iguana+iguana%27%29%29
52	81	Common green iguana	Iguana iguana	Squamata	Lizard	El Salvador	America	FB	https://reptile-database.reptarium.cz/species?genus=Iguana&species=iguana&search_param=%28%28common_name%3D%27Iguana+iguana%27%29%29
52	81	Common green iguana	Iguana iguana	Squamata	Lizard	El Salvador	America	FB	https://reptile-database.reptarium.cz/species?genus=Iguana&species=iguana&search_param=%28%28common_name%3D%27Iguana+iguana%27%29%29
52	81	Common green iguana	Iguana iguana	Squamata	Lizard	El Salvador	America	FB	https://reptile-database.reptarium.cz/species?genus=Iguana&species=iguana&search_param=%28%28common_name%3D%27Iguana+iguana%27%29%29
52	81	Common green iguana	Iguana iguana	Squamata	Lizard	El Salvador	America	FB	https://reptile-database.reptarium.cz/species?genus=Iguana&species=iguana&search_param=%28%28common_name%3D%27Iguana+iguana%27%29%29
52	81	Common green iguana	Iguana iguana	Squamata	Lizard	El Salvador	America	FB	https://reptile-database.reptarium.cz/species?genus=Iguana&species=iguana&search_param=%28%28common_name%3D%27Iguana+iguana%27%29%29
52	81	Common green iguana	Iguana iguana	Squamata	Lizard	El Salvador	America	FB	https://reptile-database.reptarium.cz/species?genus=Iguana&species=iguana&search_param=%28%28common_name%3D%27Iguana+iguana%27%29%29
52	81	Common green iguana	Iguana iguana	Squamata	Lizard	El Salvador	America	FB	https://reptile-database.reptarium.cz/species?genus=Iguana&species=iguana&search_param=%28%28common_name%3D%27Iguana+iguana%27%29%29
52	81	Common green iguana	Iguana iguana	Squamata	Lizard	El Salvador	America	FB	https://reptile-database.reptarium.cz/species?genus=Iguana&species=iguana&search_param=%28%28common_name%3D%27Iguana+iguana%27%29%29

Table S1: Animal species sampled in this study - country origin and categorization as captive bred (CB), farm bred (FB) and wild-caught (WC)

Shipment No.	Sample batch	Animal species	Scientific name of animal/s	Order	Suborder	Country origin	Continent	Captive bred (CB), farm bred (FB), wild-caught (WC)	Web URL used for assigning animal species to categories CB, FB and WC
52	81	Common green iguana	Iguana iguana	Squamata	Lizard	El Salvador	America	FB	https://reptile-database.reptarium.cz/species?genus=Iguana&species=iguana&search_param=%28%28common_name%3D%27Iguana+iguana%27%29%29
53	82	Red-footed tortoise	Chelonoidis carbonarius	Testudines	Turtle	Brazil	America	WC/FB	https://reptile-database.reptarium.cz/species?genus=Chelonoidis&species=carbonarius&search_param=%28%28common_name%3D%27Chelonoidis+carbonarius%27%29%29
53	82	Red-footed tortoise	Chelonoidis carbonarius	Testudines	Turtle	Brazil	America	WC/FB	https://reptile-database.reptarium.cz/species?genus=Chelonoidis&species=carbonarius&search_param=%28%28common_name%3D%27Chelonoidis+carbonarius%27%29%29
53	82	Red-footed tortoise	Chelonoidis carbonarius	Testudines	Turtle	Brazil	America	WC/FB	https://reptile-database.reptarium.cz/species?genus=Chelonoidis&species=carbonarius&search_param=%28%28common_name%3D%27Chelonoidis+carbonarius%27%29%29
53	82	Red-footed tortoise	Chelonoidis carbonarius	Testudines	Turtle	Brazil	America	WC/FB	https://reptile-database.reptarium.cz/species?genus=Chelonoidis&species=carbonarius&search_param=%28%28common_name%3D%27Chelonoidis+carbonarius%27%29%29
53	82	Red-footed tortoise	Chelonoidis carbonarius	Testudines	Turtle	Brazil	America	WC/FB	https://reptile-database.reptarium.cz/species?genus=Chelonoidis&species=carbonarius&search_param=%28%28common_name%3D%27Chelonoidis+carbonarius%27%29%29
53	82	Red-footed tortoise	Chelonoidis carbonarius	Testudines	Turtle	Brazil	America	WC/FB	https://reptile-database.reptarium.cz/species?genus=Chelonoidis&species=carbonarius&search_param=%28%28common_name%3D%27Chelonoidis+carbonarius%27%29%29
53	82	Red-footed tortoise	Chelonoidis carbonarius	Testudines	Turtle	Brazil	America	WC/FB	https://reptile-database.reptarium.cz/species?genus=Chelonoidis&species=carbonarius&search_param=%28%28common_name%3D%27Chelonoidis+carbonarius%27%29%29
53	82	Red-footed tortoise	Chelonoidis carbonarius	Testudines	Turtle	Brazil	America	WC/FB	https://reptile-database.reptarium.cz/species?genus=Chelonoidis&species=carbonarius&search_param=%28%28common_name%3D%27Chelonoidis+carbonarius%27%29%29
53	82	Red-footed tortoise	Chelonoidis carbonarius	Testudines	Turtle	Brazil	America	WC/FB	https://reptile-database.reptarium.cz/species?genus=Chelonoidis&species=carbonarius&search_param=%28%28common_name%3D%27Chelonoidis+carbonarius%27%29%29
53	82	Red-footed tortoise	Chelonoidis carbonarius	Testudines	Turtle	Brazil	America	WC/FB	https://reptile-database.reptarium.cz/species?genus=Chelonoidis&species=carbonarius&search_param=%28%28common_name%3D%27Chelonoidis+carbonarius%27%29%29
54	83	Red-footed tortoise	Chelonoidis carbonarius	Testudines	Turtle	Guatemala	America	WC/FB	https://reptile-database.reptarium.cz/species?genus=Chelonoidis&species=carbonarius&search_param=%28%28common_name%3D%27Chelonoidis+carbonarius%27%29%29

Table S1: Animal species sampled in this study - country origin and categorization as captive bred (CB), farm bred (FB) and wild-caught (WC)

Shipment No.	Sample batch	Animal species	Scientific name of animal/s	Order	Suborder	Country origin	Continent	Captive bred (CB), farm bred (FB), wild-caught (WC)	Web URL used for assigning animal species to categories CB, FB and WC
54	83	Red-footed tortoise	Chelonoidis carbonarius	Testudines	Turtle	Guatemala	America	WC/FB	https://reptile-database.reptarium.cz/species?genus=Chelonoidis&species=carbonarius&search_param=%28%28common_name%3D%27Chelonoidis+carbonarius%27%29%29
54	83	Red-footed tortoise	Chelonoidis carbonarius	Testudines	Turtle	Guatemala	America	WC/FB	https://reptile-database.reptarium.cz/species?genus=Chelonoidis&species=carbonarius&search_param=%28%28common_name%3D%27Chelonoidis+carbonarius%27%29%29
54	83	Red-footed tortoise	Chelonoidis carbonarius	Testudines	Turtle	Guatemala	America	WC/FB	https://reptile-database.reptarium.cz/species?genus=Chelonoidis&species=carbonarius&search_param=%28%28common_name%3D%27Chelonoidis+carbonarius%27%29%29
54	83	Red-footed tortoise	Chelonoidis carbonarius	Testudines	Turtle	Guatemala	America	WC/FB	https://reptile-database.reptarium.cz/species?genus=Chelonoidis&species=carbonarius&search_param=%28%28common_name%3D%27Chelonoidis+carbonarius%27%29%29
54	83	Red-footed tortoise	Chelonoidis carbonarius	Testudines	Turtle	Guatemala	America	WC/FB	https://reptile-database.reptarium.cz/species?genus=Chelonoidis&species=carbonarius&search_param=%28%28common_name%3D%27Chelonoidis+carbonarius%27%29%29
54	83	Red-footed tortoise	Chelonoidis carbonarius	Testudines	Turtle	Guatemala	America	WC/FB	https://reptile-database.reptarium.cz/species?genus=Chelonoidis&species=carbonarius&search_param=%28%28common_name%3D%27Chelonoidis+carbonarius%27%29%29
54	83	Red-footed tortoise	Chelonoidis carbonarius	Testudines	Turtle	Guatemala	America	WC/FB	https://reptile-database.reptarium.cz/species?genus=Chelonoidis&species=carbonarius&search_param=%28%28common_name%3D%27Chelonoidis+carbonarius%27%29%29
54	83	Red-footed tortoise	Chelonoidis carbonarius	Testudines	Turtle	Guatemala	America	WC/FB	https://reptile-database.reptarium.cz/species?genus=Chelonoidis&species=carbonarius&search_param=%28%28common_name%3D%27Chelonoidis+carbonarius%27%29%29
54	83	Red-footed tortoise	Chelonoidis carbonarius	Testudines	Turtle	Guatemala	America	WC/FB	https://reptile-database.reptarium.cz/species?genus=Chelonoidis&species=carbonarius&search_param=%28%28common_name%3D%27Chelonoidis+carbonarius%27%29%29
54	83	Red-footed tortoise	Chelonoidis carbonarius	Testudines	Turtle	Guatemala	America	WC/FB	https://reptile-database.reptarium.cz/species?genus=Chelonoidis&species=carbonarius&search_param=%28%28common_name%3D%27Chelonoidis+carbonarius%27%29%29
55	84	Eastern collared lizard	Crotaphytus insularis	Squamata	Lizard	USA	America	WC	https://reptile-database.reptarium.cz/species?genus=Crotaphytus&species=insularis&search_param=%28%28common_name%3D%27Crotaphytus+collaris%27%29%29
55	84	Eastern collared lizard	Crotaphytus insularis	Squamata	Lizard	USA	America	WC	https://reptile-database.reptarium.cz/species?genus=Crotaphytus&species=insularis&search_param=%28%28common_name%3D%27Crotaphytus+collaris%27%29%29

Table S1: Animal species sampled in this study - country origin and categorization as captive bred (CB), farm bred (FB) and wild-caught (WC)

Shipment No.	Sample batch	Animal species	Scientific name of animal/s	Order	Suborder	Country origin	Continent	Captive bred (CB), farm bred (FB), wild-caught (WC)	Web URL used for assigning animal species to categories CB, FB and WC
55	85	Trinket snake	Coelognathus helena	Testudines	Snake	USA	America	CB	https://reptile-database.reptarium.cz/species?genus=Coelognathus&species=helena&search_param=%28%28common_name%3D%27Coelognathus+helena%27%29%29
55	86	Eastern milksnake	Lampropeltis triangulum	Squamata	Snake	USA	America	CB	https://reptile-database.reptarium.cz/species?genus=Lampropeltis&species=triangulum&search_param=%28%28common_name%3D%27Lampropeltis+triangulum%27%29%29
55	86	Eastern milksnake	Lampropeltis triangulum	Squamata	Snake	USA	America	CB	https://reptile-database.reptarium.cz/species?genus=Lampropeltis&species=triangulum&search_param=%28%28common_name%3D%27Lampropeltis+triangulum%27%29%29
55	86	Eastern milksnake	Lampropeltis triangulum	Squamata	Snake	USA	America	CB	https://reptile-database.reptarium.cz/species?genus=Lampropeltis&species=triangulum&search_param=%28%28common_name%3D%27Lampropeltis+triangulum%27%29%29
55	86	Eastern milksnake	Lampropeltis triangulum	Squamata	Snake	USA	America	CB	https://reptile-database.reptarium.cz/species?genus=Lampropeltis&species=triangulum&search_param=%28%28common_name%3D%27Lampropeltis+triangulum%27%29%29
55	86	Eastern milksnake	Lampropeltis triangulum	Squamata	Snake	USA	America	CB	https://reptile-database.reptarium.cz/species?genus=Lampropeltis&species=triangulum&search_param=%28%28common_name%3D%27Lampropeltis+triangulum%27%29%29
55	86	Eastern milksnake	Lampropeltis triangulum	Squamata	Snake	USA	America	CB	https://reptile-database.reptarium.cz/species?genus=Lampropeltis&species=triangulum&search_param=%28%28common_name%3D%27Lampropeltis+triangulum%27%29%29
55	86	Eastern milksnake	Lampropeltis triangulum	Squamata	Snake	USA	America	CB	https://reptile-database.reptarium.cz/species?genus=Lampropeltis&species=triangulum&search_param=%28%28common_name%3D%27Lampropeltis+triangulum%27%29%29
55	87	Common garter snake	Thamnophis sirtalis	Squamata	Snake	USA	America	WC/FB	https://reptile-database.reptarium.cz/species?genus=Thamnophis&species=sirtalis&search_param=%28%28common_name%3D%27Thamnophis+sirtalis%27%29%29
55	87	Common garter snake	Thamnophis sirtalis	Squamata	Snake	USA	America	WC/FB	https://reptile-database.reptarium.cz/species?genus=Thamnophis&species=sirtalis&search_param=%28%28common_name%3D%27Thamnophis+sirtalis%27%29%29
55	87	Common garter snake	Thamnophis sirtalis	Squamata	Snake	USA	America	WC/FB	https://reptile-database.reptarium.cz/species?genus=Thamnophis&species=sirtalis&search_param=%28%28common_name%3D%27Thamnophis+sirtalis%27%29%29
55	87	Common garter snake	Thamnophis sirtalis	Squamata	Snake	USA	America	WC/FB	https://reptile-database.reptarium.cz/species?genus=Thamnophis&species=sirtalis&search_param=%28%28common_name%3D%27Thamnophis+sirtalis%27%29%29

Table S1: Animal species sampled in this study - country origin and categorization as captive bred (CB), farm bred (FB) and wild-caught (WC)

Shipment No.	Sample batch	Animal species	Scientific name of animal/s	Order	Suborder	Country origin	Continent	Captive bred (CB), farm bred (FB), wild-caught (WC)	Web URL used for assigning animal species to categories CB, FB and WC
55	87	Common garter snake	Thamnophis sirtalis	Squamata	Snake	USA	America	WC/FB	https://reptile-database.reptarium.cz/species?genus=Thamnophis&species=sirtalis&search_param=%28%28common_name%3D%27Thamnophis+sirtalis%27%29%29
55	87	Common garter snake	Thamnophis sirtalis	Squamata	Snake	USA	America	WC/FB	https://reptile-database.reptarium.cz/species?genus=Thamnophis&species=sirtalis&search_param=%28%28common_name%3D%27Thamnophis+sirtalis%27%29%29
55	87	Common garter snake	Thamnophis sirtalis	Squamata	Snake	USA	America	WC/FB	https://reptile-database.reptarium.cz/species?genus=Thamnophis&species=sirtalis&search_param=%28%28common_name%3D%27Thamnophis+sirtalis%27%29%29
55	87	Common garter snake	Thamnophis sirtalis	Squamata	Snake	USA	America	WC/FB	https://reptile-database.reptarium.cz/species?genus=Thamnophis&species=sirtalis&search_param=%28%28common_name%3D%27Thamnophis+sirtalis%27%29%29
55	87	Common garter snake	Thamnophis sirtalis	Squamata	Snake	USA	America	WC/FB	https://reptile-database.reptarium.cz/species?genus=Thamnophis&species=sirtalis&search_param=%28%28common_name%3D%27Thamnophis+sirtalis%27%29%29
56	88	Bell's hingeback tortoise	Kinixys belliana	Testudines	Turtle	Egypt	Africa	WC	https://reptile-database.reptarium.cz/species?genus=Kinixys&species=belliana&search_param=%28%28common_name%3D%27Kinixys+belliana%27%29%29
56	88	Bell's hingeback tortoise	Kinixys belliana	Testudines	Turtle	Egypt	Africa	WC	https://reptile-database.reptarium.cz/species?genus=Kinixys&species=belliana&search_param=%28%28common_name%3D%27Kinixys+belliana%27%29%29
56	88	Bell's hingeback tortoise	Kinixys belliana	Testudines	Turtle	Egypt	Africa	WC	https://reptile-database.reptarium.cz/species?genus=Kinixys&species=belliana&search_param=%28%28common_name%3D%27Kinixys+belliana%27%29%29
56	88	Bell's hingeback tortoise	Kinixys belliana	Testudines	Turtle	Egypt	Africa	WC	https://reptile-database.reptarium.cz/species?genus=Kinixys&species=belliana&search_param=%28%28common_name%3D%27Kinixys+belliana%27%29%29
56	88	Bell's hingeback tortoise	Kinixys belliana	Testudines	Turtle	Egypt	Africa	WC	https://reptile-database.reptarium.cz/species?genus=Kinixys&species=belliana&search_param=%28%28common_name%3D%27Kinixys+belliana%27%29%29
56	88	Bell's hingeback tortoise	Kinixys belliana	Testudines	Turtle	Egypt	Africa	WC	https://reptile-database.reptarium.cz/species?genus=Kinixys&species=belliana&search_param=%28%28common_name%3D%27Kinixys+belliana%27%29%29
56	89	Common agama	Agama agama	Squamata	Lizard	Egypt	Africa	WC	https://reptile-database.reptarium.cz/species?genus=Agama&species=agama&search_param=%28%28common_name%3D%27agam+agama%27%29%29

Table S1: Animal species sampled in this study - country origin and categorization as captive bred (CB), farm bred (FB) and wild-caught (WC)

Shipment No.	Sample batch	Animal species	Scientific name of animal/s	Order	Suborder	Country origin	Continent	Captive bred (CB), farm bred (FB), wild-caught (WC)	Web URL used for assigning animal species to categories CB, FB and WC
56	89	Common agama	Agama agama	Squamata	Lizard	Egypt	Africa	WC	https://reptile-database.reptarium.cz/species?genus=Agama&species=agama&search_param=%28%28common_name%3D%27agam+agama%27%29%29
56	89	Common agama	Agama agama	Squamata	Lizard	Egypt	Africa	WC	https://reptile-database.reptarium.cz/species?genus=Agama&species=agama&search_param=%28%28common_name%3D%27agam+agama%27%29%29
56	89	Common agama	Agama agama	Squamata	Lizard	Egypt	Africa	WC	https://reptile-database.reptarium.cz/species?genus=Agama&species=agama&search_param=%28%28common_name%3D%27agam+agama%27%29%29
56	89	Common agama	Agama agama	Squamata	Lizard	Egypt	Africa	WC	https://reptile-database.reptarium.cz/species?genus=Agama&species=agama&search_param=%28%28common_name%3D%27agam+agama%27%29%29
56	89	Common agama	Agama agama	Squamata	Lizard	Egypt	Africa	WC	https://reptile-database.reptarium.cz/species?genus=Agama&species=agama&search_param=%28%28common_name%3D%27agam+agama%27%29%29
56	89	Common agama	Agama agama	Squamata	Lizard	Egypt	Africa	WC	https://reptile-database.reptarium.cz/species?genus=Agama&species=agama&search_param=%28%28common_name%3D%27agam+agama%27%29%29
56	89	Common agama	Agama agama	Squamata	Lizard	Egypt	Africa	WC	https://reptile-database.reptarium.cz/species?genus=Agama&species=agama&search_param=%28%28common_name%3D%27agam+agama%27%29%29
57	90	Asian grass lizard	Takydromus sexlineatus	Squamata	Lizard	Vietnam	Asia	WC	https://reptile-database.reptarium.cz/species?genus=Takydromus&species=sexlineatus&search_param=%28%28common_name%3D%27Takydromus+sexlineatus%27%29%29
57	90	Asian grass lizard	Takydromus sexlineatus	Squamata	Lizard	Vietnam	Asia	WC	https://reptile-database.reptarium.cz/species?genus=Takydromus&species=sexlineatus&search_param=%28%28common_name%3D%27Takydromus+sexlineatus%27%29%29
57	90	Asian grass lizard	Takydromus sexlineatus	Squamata	Lizard	Vietnam	Asia	WC	https://reptile-database.reptarium.cz/species?genus=Takydromus&species=sexlineatus&search_param=%28%28common_name%3D%27Takydromus+sexlineatus%27%29%29
57	90	Asian grass lizard	Takydromus sexlineatus	Squamata	Lizard	Vietnam	Asia	WC	https://reptile-database.reptarium.cz/species?genus=Takydromus&species=sexlineatus&search_param=%28%28common_name%3D%27Takydromus+sexlineatus%27%29%29
57	90	Asian grass lizard	Takydromus sexlineatus	Squamata	Lizard	Vietnam	Asia	WC	https://reptile-database.reptarium.cz/species?genus=Takydromus&species=sexlineatus&search_param=%28%28common_name%3D%27Takydromus+sexlineatus%27%29%29

Table S1: Animal species sampled in this study - country origin and categorization as captive bred (CB), farm bred (FB) and wild-caught (WC)

Shipment No.	Sample batch	Animal species	Scientific name of animal/s	Order	Suborder	Country origin	Continent	Captive bred (CB), farm bred (FB), wild-caught (WC)	Web URL used for assigning animal species to categories CB, FB and WC
57	91	Eastern garden lizard / Chinese water dragon	Calotes versicolor / Physignatus cocincinus	Squamata	Lizard	Vietnam	Asia	WC	https://reptile-database.reptarium.cz/species?genus=Calotes&species=versicolor&search_param=%28%28common_name%3D%27Calotes+versicolor%27%29%29 , https://reptile-database.reptarium.cz/species?genus=Physignathus&species=cocincinus&search_param=%28%28common_name%3D%27Physignatus+cocincinus%27%29%29
57	91	Eastern garden lizard / Chinese water dragon	Calotes versicolor / Physignatus cocincinus	Squamata	Lizard	Vietnam	Asia	WC	https://reptile-database.reptarium.cz/species?genus=Calotes&species=versicolor&search_param=%28%28common_name%3D%27Calotes+versicolor%27%29%29 , https://reptile-database.reptarium.cz/species?genus=Physignathus&species=cocincinus&search_param=%28%28common_name%3D%27Physignatus+cocincinus%27%29%29
57	91	Eastern garden lizard / Chinese water dragon	Calotes versicolor / Physignatus cocincinus	Squamata	Lizard	Vietnam	Asia	WC	https://reptile-database.reptarium.cz/species?genus=Calotes&species=versicolor&search_param=%28%28common_name%3D%27Calotes+versicolor%27%29%29 , https://reptile-database.reptarium.cz/species?genus=Physignathus&species=cocincinus&search_param=%28%28common_name%3D%27Physignatus+cocincinus%27%29%29
57	91	Eastern garden lizard / Chinese water dragon	Calotes versicolor / Physignatus cocincinus	Squamata	Lizard	Vietnam	Asia	WC	https://reptile-database.reptarium.cz/species?genus=Calotes&species=versicolor&search_param=%28%28common_name%3D%27Calotes+versicolor%27%29%29 , https://reptile-database.reptarium.cz/species?genus=Physignathus&species=cocincinus&search_param=%28%28common_name%3D%27Physignatus+cocincinus%27%29%29
57	91	Eastern garden lizard / Chinese water dragon	Calotes versicolor / Physignatus cocincinus	Squamata	Lizard	Vietnam	Asia	WC	https://reptile-database.reptarium.cz/species?genus=Calotes&species=versicolor&search_param=%28%28common_name%3D%27Calotes+versicolor%27%29%29 , https://reptile-database.reptarium.cz/species?genus=Physignathus&species=cocincinus&search_param=%28%28common_name%3D%27Physignatus+cocincinus%27%29%29
57	91	Eastern garden lizard / Chinese water dragon	Calotes versicolor / Physignatus cocincinus	Squamata	Lizard	Vietnam	Asia	WC	https://reptile-database.reptarium.cz/species?genus=Calotes&species=versicolor&search_param=%28%28common_name%3D%27Calotes+versicolor%27%29%29 , https://reptile-database.reptarium.cz/species?genus=Physignathus&species=cocincinus&search_param=%28%28common_name%3D%27Physignatus+cocincinus%27%29%29
57	91	Eastern garden lizard / Chinese water dragon	Calotes versicolor / Physignatus cocincinus	Squamata	Lizard	Vietnam	Asia	WC	https://reptile-database.reptarium.cz/species?genus=Calotes&species=versicolor&search_param=%28%28common_name%3D%27Calotes+versicolor%27%29%29 , https://reptile-database.reptarium.cz/species?genus=Physignathus&species=cocincinus&search_param=%28%28common_name%3D%27Physignatus+cocincinus%27%29%29

Table S1: Animal species sampled in this study - country origin and categorization as captive bred (CB), farm bred (FB) and wild-caught (WC)

Shipment No.	Sample batch	Animal species	Scientific name of animal/s	Order	Suborder	Country origin	Continent	Captive bred (CB), farm bred (FB), wild-caught (WC)	Web URL used for assigning animal species to categories CB, FB and WC
57	91	Eastern garden lizard / Chinese water dragon	Calotes versicolor / Physignatus cocincinus	Squamata	Lizard	Vietnam	Asia	WC	https://reptile-database.reptarium.cz/species?genus=Calotes&species=versicolor&search_param=%28%28common_name%3D%27Calotes+versicolor%27%29%29 , https://reptile-database.reptarium.cz/species?genus=Physignathus&species=cocincinus&search_param=%28%28common_name%3D%27Physignatus+cocincinus%27%29%29
57	91	Eastern garden lizard / Chinese water dragon	Calotes versicolor / Physignatus cocincinus	Squamata	Lizard	Vietnam	Asia	WC	https://reptile-database.reptarium.cz/species?genus=Calotes&species=versicolor&search_param=%28%28common_name%3D%27Calotes+versicolor%27%29%29 , https://reptile-database.reptarium.cz/species?genus=Physignathus&species=cocincinus&search_param=%28%28common_name%3D%27Physignatus+cocincinus%27%29%29
57	91	Eastern garden lizard / Chinese water dragon	Calotes versicolor / Physignatus cocincinus	Squamata	Lizard	Vietnam	Asia	WC	https://reptile-database.reptarium.cz/species?genus=Calotes&species=versicolor&search_param=%28%28common_name%3D%27Calotes+versicolor%27%29%29 , https://reptile-database.reptarium.cz/species?genus=Physignathus&species=cocincinus&search_param=%28%28common_name%3D%27Physignatus+cocincinus%27%29%29
57	91	Eastern garden lizard / Chinese water dragon	Calotes versicolor / Physignatus cocincinus	Squamata	Lizard	Vietnam	Asia	WC	https://reptile-database.reptarium.cz/species?genus=Calotes&species=versicolor&search_param=%28%28common_name%3D%27Calotes+versicolor%27%29%29 , https://reptile-database.reptarium.cz/species?genus=Physignathus&species=cocincinus&search_param=%28%28common_name%3D%27Physignatus+cocincinus%27%29%29
57	91	Eastern garden lizard / Chinese water dragon	Calotes versicolor / Physignatus cocincinus	Squamata	Lizard	Vietnam	Asia	WC	https://reptile-database.reptarium.cz/species?genus=Calotes&species=versicolor&search_param=%28%28common_name%3D%27Calotes+versicolor%27%29%29 , https://reptile-database.reptarium.cz/species?genus=Physignathus&species=cocincinus&search_param=%28%28common_name%3D%27Physignatus+cocincinus%27%29%29
57	91	Eastern garden lizard / Chinese water dragon	Calotes versicolor / Physignatus cocincinus	Squamata	Lizard	Vietnam	Asia	WC	https://reptile-database.reptarium.cz/species?genus=Calotes&species=versicolor&search_param=%28%28common_name%3D%27Calotes+versicolor%27%29%29 , https://reptile-database.reptarium.cz/species?genus=Physignathus&species=cocincinus&search_param=%28%28common_name%3D%27Physignatus+cocincinus%27%29%29
57	91	Eastern garden lizard / Chinese water dragon	Calotes versicolor / Physignatus cocincinus	Squamata	Lizard	Vietnam	Asia	WC	https://reptile-database.reptarium.cz/species?genus=Calotes&species=versicolor&search_param=%28%28common_name%3D%27Calotes+versicolor%27%29%29 , https://reptile-database.reptarium.cz/species?genus=Physignathus&species=cocincinus&search_param=%28%28common_name%3D%27Physignatus+cocincinus%27%29%29

Table S1: Animal species sampled in this study - country origin and categorization as captive bred (CB), farm bred (FB) and wild-caught (WC)

Shipment No.	Sample batch	Animal species	Scientific name of animal/s	Order	Suborder	Country origin	Continent	Captive bred (CB), farm bred (FB), wild-caught (WC)	Web URL used for assigning animal species to categories CB, FB and WC
57	91	Eastern garden lizard / Chinese water dragon	Calotes versicolor / Physignatus cocincinus	Squamata	Lizard	Vietnam	Asia	WC	https://reptile-database.reptarium.cz/species?genus=Calotes&species=versicolor&search_param=%28%28common_name%3D%27Calotes+versicolor%27%29%29 , https://reptile-database.reptarium.cz/species?genus=Physignathus&species=cocincinus&search_param=%28%28common_name%3D%27Physignatus+cocincinus%27%29%29
57	92	Common house gecko	Hemidactylus frenatus	Squamata	Lizard	Vietnam	Asia	WC	https://reptile-database.reptarium.cz/species?genus=Hemidactylus&species=frenatus&search_param=%28%28common_name%3D%27Hemidactylus+frenatus%27%29%29
57	92	Common house gecko	Hemidactylus frenatus	Squamata	Lizard	Vietnam	Asia	WC	https://reptile-database.reptarium.cz/species?genus=Hemidactylus&species=frenatus&search_param=%28%28common_name%3D%27Hemidactylus+frenatus%27%29%29
57	92	Common house gecko	Hemidactylus frenatus	Squamata	Lizard	Vietnam	Asia	WC	https://reptile-database.reptarium.cz/species?genus=Hemidactylus&species=frenatus&search_param=%28%28common_name%3D%27Hemidactylus+frenatus%27%29%29
57	92	Common house gecko	Hemidactylus frenatus	Squamata	Lizard	Vietnam	Asia	WC	https://reptile-database.reptarium.cz/species?genus=Hemidactylus&species=frenatus&search_param=%28%28common_name%3D%27Hemidactylus+frenatus%27%29%29
58	93	Hispaniolan masked curlytail	Leiocephalus personatus	Squamata	Lizard	USA	America	WC/FB	https://reptile-database.reptarium.cz/species?genus=Leiocephalus&species=personatus&search_param=%28%28common_name%3D%27maskenleguan%27%29%29
58	93	Hispaniolan masked curlytail	Leiocephalus personatus	Squamata	Lizard	USA	America	WC/FB	https://reptile-database.reptarium.cz/species?genus=Leiocephalus&species=personatus&search_param=%28%28common_name%3D%27maskenleguan%27%29%29
58	93	Hispaniolan masked curlytail	Leiocephalus personatus	Squamata	Lizard	USA	America	WC/FB	https://reptile-database.reptarium.cz/species?genus=Leiocephalus&species=personatus&search_param=%28%28common_name%3D%27maskenleguan%27%29%29
58	93	Hispaniolan masked curlytail	Leiocephalus personatus	Squamata	Lizard	USA	America	WC/FB	https://reptile-database.reptarium.cz/species?genus=Leiocephalus&species=personatus&search_param=%28%28common_name%3D%27maskenleguan%27%29%29
58	93	Hispaniolan masked curlytail	Leiocephalus personatus	Squamata	Lizard	USA	America	WC/FB	https://reptile-database.reptarium.cz/species?genus=Leiocephalus&species=personatus&search_param=%28%28common_name%3D%27maskenleguan%27%29%29
58	94	Striped skink	Trachylepis striata	Squamata	Lizard	USA	America	WC/FB	https://reptile-database.reptarium.cz/species?genus=Trachylepis&species=striata&search_param=%28%28common_name%3D%27Trachylepis+striata%27%29%29

Table S1: Animal species sampled in this study - country origin and categorization as captive bred (CB), farm bred (FB) and wild-caught (WC)

Shipment No.	Sample batch	Animal species	Scientific name of animal/s	Order	Suborder	Country origin	Continent	Captive bred (CB), farm bred (FB), wild-caught (WC)	Web URL used for assigning animal species to categories CB, FB and WC
58	94	Striped skink	Trachylepis striata	Squamata	Lizard	USA	America	WC/FB	https://reptile-database.reptarium.cz/species?genus=Trachylepis&species=striata&search_param=%28%28common_name%3D%27Trachylepis+striata%27%29%29
58	94	Striped skink	Trachylepis striata	Squamata	Lizard	USA	America	WC/FB	https://reptile-database.reptarium.cz/species?genus=Trachylepis&species=striata&search_param=%28%28common_name%3D%27Trachylepis+striata%27%29%29
58	94	Striped skink	Trachylepis striata	Squamata	Lizard	USA	America	WC/FB	https://reptile-database.reptarium.cz/species?genus=Trachylepis&species=striata&search_param=%28%28common_name%3D%27Trachylepis+striata%27%29%29
58	94	Striped skink	Trachylepis striata	Squamata	Lizard	USA	America	WC/FB	https://reptile-database.reptarium.cz/species?genus=Trachylepis&species=striata&search_param=%28%28common_name%3D%27Trachylepis+striata%27%29%29
58	95	Saw-scaled curlytail	Leiocephalus carinatus	Squamata	Lizard	USA	America	WC/FB	https://reptile-database.reptarium.cz/species?genus=Leiocephalus&species=carinatus&search_param=%28%28common_name%3D%27Leiocephalus+carinatus%27%29%29
58	95	Saw-scaled curlytail	Leiocephalus carinatus	Squamata	Lizard	USA	America	WC/FB	https://reptile-database.reptarium.cz/species?genus=Leiocephalus&species=carinatus&search_param=%28%28common_name%3D%27Leiocephalus+carinatus%27%29%29
58	95	Saw-scaled curlytail	Leiocephalus carinatus	Squamata	Lizard	USA	America	WC/FB	https://reptile-database.reptarium.cz/species?genus=Leiocephalus&species=carinatus&search_param=%28%28common_name%3D%27Leiocephalus+carinatus%27%29%29
58	95	Saw-scaled curlytail	Leiocephalus carinatus	Squamata	Lizard	USA	America	WC/FB	https://reptile-database.reptarium.cz/species?genus=Leiocephalus&species=carinatus&search_param=%28%28common_name%3D%27Leiocephalus+carinatus%27%29%29
58	95	Saw-scaled curlytail	Leiocephalus carinatus	Squamata	Lizard	USA	America	WC/FB	https://reptile-database.reptarium.cz/species?genus=Leiocephalus&species=carinatus&search_param=%28%28common_name%3D%27Leiocephalus+carinatus%27%29%29
58	95	Saw-scaled curlytail	Leiocephalus carinatus	Squamata	Lizard	USA	America	WC/FB	https://reptile-database.reptarium.cz/species?genus=Leiocephalus&species=carinatus&search_param=%28%28common_name%3D%27Leiocephalus+carinatus%27%29%29
58	96	Cuban giant anole	Anolis equestris	Squamata	Lizard	USA	America	WC/FB	https://reptile-database.reptarium.cz/species?genus=Anolis&species=equestris&search_param=%28%28common_name%3D%27Anolis+equestris%27%29%29
58	96	Cuban giant anole	Anolis equestris	Squamata	Lizard	USA	America	WC/FB	https://reptile-database.reptarium.cz/species?genus=Anolis&species=equestris&search_param=%28%28common_name%3D%27Anolis+equestris%27%29%29

Table S1: Animal species sampled in this study - country origin and categorization as captive bred (CB), farm bred (FB) and wild-caught (WC)

Shipment No.	Sample batch	Animal species	Scientific name of animal/s	Order	Suborder	Country origin	Continent	Captive bred (CB), farm bred (FB), wild-caught (WC)	Web URL used for assigning animal species to categories CB, FB and WC
58	96	Cuban giant anole	Anolis equestris	Squamata	Lizard	USA	America	WC/FB	https://reptile-database.reptarium.cz/species?genus=Anolis&species=equestris&search_param=%28%28common_name%3D%27Anolis+equestris%27%29%29
58	96	Cuban giant anole	Anolis equestris	Squamata	Lizard	USA	America	WC/FB	https://reptile-database.reptarium.cz/species?genus=Anolis&species=equestris&search_param=%28%28common_name%3D%27Anolis+equestris%27%29%29
58	96	Cuban giant anole	Anolis equestris	Squamata	Lizard	USA	America	WC/FB	https://reptile-database.reptarium.cz/species?genus=Anolis&species=equestris&search_param=%28%28common_name%3D%27Anolis+equestris%27%29%29
58	96	Cuban giant anole	Anolis equestris	Squamata	Lizard	USA	America	WC/FB	https://reptile-database.reptarium.cz/species?genus=Anolis&species=equestris&search_param=%28%28common_name%3D%27Anolis+equestris%27%29%29
58	96	Cuban giant anole	Anolis equestris	Squamata	Lizard	USA	America	WC/FB	https://reptile-database.reptarium.cz/species?genus=Anolis&species=equestris&search_param=%28%28common_name%3D%27Anolis+equestris%27%29%29
58	96	Cuban giant anole	Anolis equestris	Squamata	Lizard	USA	America	WC/FB	https://reptile-database.reptarium.cz/species?genus=Anolis&species=equestris&search_param=%28%28common_name%3D%27Anolis+equestris%27%29%29
58	96	Cuban giant anole	Anolis equestris	Squamata	Lizard	USA	America	WC/FB	https://reptile-database.reptarium.cz/species?genus=Anolis&species=equestris&search_param=%28%28common_name%3D%27Anolis+equestris%27%29%29
58	96	Cuban giant anole	Anolis equestris	Squamata	Lizard	USA	America	WC/FB	https://reptile-database.reptarium.cz/species?genus=Anolis&species=equestris&search_param=%28%28common_name%3D%27Anolis+equestris%27%29%29
58	97	Striped mud turtle	Kinosternon baurii	Testudines	Turtle	USA	America	FB	https://reptile-database.reptarium.cz/species?genus=Kinosternon&species=baurii&search_param=%28%28common_name%3D%27Kinosternon+baurii%27%29%29
58	97	Striped mud turtle	Kinosternon baurii	Testudines	Turtle	USA	America	FB	https://reptile-database.reptarium.cz/species?genus=Kinosternon&species=baurii&search_param=%28%28common_name%3D%27Kinosternon+baurii%27%29%29
58	97	Striped mud turtle	Kinosternon baurii	Testudines	Turtle	USA	America	FB	https://reptile-database.reptarium.cz/species?genus=Kinosternon&species=baurii&search_param=%28%28common_name%3D%27Kinosternon+baurii%27%29%29
58	97	Striped mud turtle	Kinosternon baurii	Testudines	Turtle	USA	America	FB	https://reptile-database.reptarium.cz/species?genus=Kinosternon&species=baurii&search_param=%28%28common_name%3D%27Kinosternon+baurii%27%29%29

Table S1: Animal species sampled in this study - country origin and categorization as captive bred (CB), farm bred (FB) and wild-caught (WC)

Shipment No.	Sample batch	Animal species	Scientific name of animal/s	Order	Suborder	Country origin	Continent	Captive bred (CB), farm bred (FB), wild-caught (WC)	Web URL used for assigning animal species to categories CB, FB and WC
58	97	Striped mud turtle	Kinosternon baurii	Testudines	Turtle	USA	America	FB	https://reptile-database.reptarium.cz/species?genus=Kinosternon&species=baurii&search_param=%28%28common_name%3D%27Kinosternon+baurii%27%29%29
58	97	Striped mud turtle	Kinosternon baurii	Testudines	Turtle	USA	America	FB	https://reptile-database.reptarium.cz/species?genus=Kinosternon&species=baurii&search_param=%28%28common_name%3D%27Kinosternon+baurii%27%29%29
58	97	Striped mud turtle	Kinosternon baurii	Testudines	Turtle	USA	America	FB	https://reptile-database.reptarium.cz/species?genus=Kinosternon&species=baurii&search_param=%28%28common_name%3D%27Kinosternon+baurii%27%29%29
58	97	Striped mud turtle	Kinosternon baurii	Testudines	Turtle	USA	America	FB	https://reptile-database.reptarium.cz/species?genus=Kinosternon&species=baurii&search_param=%28%28common_name%3D%27Kinosternon+baurii%27%29%29
58	97	Striped mud turtle	Kinosternon baurii	Testudines	Turtle	USA	America	FB	https://reptile-database.reptarium.cz/species?genus=Kinosternon&species=baurii&search_param=%28%28common_name%3D%27Kinosternon+baurii%27%29%29
58	97	Striped mud turtle	Kinosternon baurii	Testudines	Turtle	USA	America	FB	https://reptile-database.reptarium.cz/species?genus=Kinosternon&species=baurii&search_param=%28%28common_name%3D%27Kinosternon+baurii%27%29%29
58	97	Striped mud turtle	Kinosternon baurii	Testudines	Turtle	USA	America	FB	https://reptile-database.reptarium.cz/species?genus=Kinosternon&species=baurii&search_param=%28%28common_name%3D%27Kinosternon+baurii%27%29%29
58	98	Razorback musk turtle	Sternotherus carinatus	Testudines	Turtle	USA	America	FB	https://reptile-database.reptarium.cz/species?genus=Sternotherus&species=carinatus&search_param=%28%28common_name%3D%27Sternotherus+carinatus%27%29%29
58	98	Razorback musk turtle	Sternotherus carinatus	Testudines	Turtle	USA	America	FB	https://reptile-database.reptarium.cz/species?genus=Sternotherus&species=carinatus&search_param=%28%28common_name%3D%27Sternotherus+carinatus%27%29%29
58	98	Razorback musk turtle	Sternotherus carinatus	Testudines	Turtle	USA	America	FB	https://reptile-database.reptarium.cz/species?genus=Sternotherus&species=carinatus&search_param=%28%28common_name%3D%27Sternotherus+carinatus%27%29%29
58	98	Razorback musk turtle	Sternotherus carinatus	Testudines	Turtle	USA	America	FB	https://reptile-database.reptarium.cz/species?genus=Sternotherus&species=carinatus&search_param=%28%28common_name%3D%27Sternotherus+carinatus%27%29%29
59	99	Ball python	Python regius	Squamata	Snake	USA	America	CB	https://reptile-database.reptarium.cz/species?genus=Python&species=regius&search_param=%28%28common_name%3D%27Python+regius%27%29%29

Table S1: Animal species sampled in this study - country origin and categorization as captive bred (CB), farm bred (FB) and wild-caught (WC)

Shipment No.	Sample batch	Animal species	Scientific name of animal/s	Order	Suborder	Country origin	Continent	Captive bred (CB), farm bred (FB), wild-caught (WC)	Web URL used for assigning animal species to categories CB, FB and WC
59	99	Ball python	Python regius	Squamata	Snake	USA	America	CB	https://reptile-database.reptarium.cz/species?genus=Python&species=regius&search_param=%28%28common_name%3D%27Python+regius%27%29%29
59	99	Ball python	Python regius	Squamata	Snake	USA	America	CB	https://reptile-database.reptarium.cz/species?genus=Python&species=regius&search_param=%28%28common_name%3D%27Python+regius%27%29%29
59	99	Ball python	Python regius	Squamata	Snake	USA	America	CB	https://reptile-database.reptarium.cz/species?genus=Python&species=regius&search_param=%28%28common_name%3D%27Python+regius%27%29%29
59	99	Ball python	Python regius	Squamata	Snake	USA	America	CB	https://reptile-database.reptarium.cz/species?genus=Python&species=regius&search_param=%28%28common_name%3D%27Python+regius%27%29%29
59	99	Ball python	Python regius	Squamata	Snake	USA	America	CB	https://reptile-database.reptarium.cz/species?genus=Python&species=regius&search_param=%28%28common_name%3D%27Python+regius%27%29%29
59	99	Ball python	Python regius	Squamata	Snake	USA	America	CB	https://reptile-database.reptarium.cz/species?genus=Python&species=regius&search_param=%28%28common_name%3D%27Python+regius%27%29%29
59	99	Ball python	Python regius	Squamata	Snake	USA	America	CB	https://reptile-database.reptarium.cz/species?genus=Python&species=regius&search_param=%28%28common_name%3D%27Python+regius%27%29%29
59	99	Ball python	Python regius	Squamata	Snake	USA	America	CB	https://reptile-database.reptarium.cz/species?genus=Python&species=regius&search_param=%28%28common_name%3D%27Python+regius%27%29%29
59	99	Ball python	Python regius	Squamata	Snake	USA	America	CB	https://reptile-database.reptarium.cz/species?genus=Python&species=regius&search_param=%28%28common_name%3D%27Python+regius%27%29%29
59	99	Ball python	Python regius	Squamata	Snake	USA	America	CB	https://reptile-database.reptarium.cz/species?genus=Python&species=regius&search_param=%28%28common_name%3D%27Python+regius%27%29%29
60	100	Leopard tortoise	Stigmochelys pardalis	Testudines	Turtle	Tanzania	Africa	FB	https://reptile-database.reptarium.cz/species?genus=Stigmochelys&species=pardalis&search_param=%28%28common_name%3D%27Stigmochelys+pardalis%27%29%29
60	100	Leopard tortoise	Stigmochelys pardalis	Testudines	Turtle	Tanzania	Africa	FB	https://reptile-database.reptarium.cz/species?genus=Stigmochelys&species=pardalis&search_param=%28%28common_name%3D%27Stigmochelys+pardalis%27%29%29

Table S1: Animal species sampled in this study - country origin and categorization as captive bred (CB), farm bred (FB) and wild-caught (WC)

Shipment No.	Sample batch	Animal species	Scientific name of animal/s	Order	Suborder	Country origin	Continent	Captive bred (CB), farm bred (FB), wild-caught (WC)	Web URL used for assigning animal species to categories CB, FB and WC
60	100	Leopard tortoise	Stigmochelys pardalis	Testudines	Turtle	Tanzania	Africa	FB	https://reptile-database.reptarium.cz/species?genus=Stigmochelys&species=pardalis&search_param=%28%28common_name%3D%27Stigmochelys+pardalis%27%29%29
61	101	Asian grass lizard	Takydromus sexlineatus	Squamata	Lizard	Vietnam	Asia	WC	https://reptile-database.reptarium.cz/species?genus=Takydromus&species=sexlineatus&search_param=%28%28common_name%3D%27Takydromus+sexlineatus%27%29%29
61	101	Asian grass lizard	Takydromus sexlineatus	Squamata	Lizard	Vietnam	Asia	WC	https://reptile-database.reptarium.cz/species?genus=Takydromus&species=sexlineatus&search_param=%28%28common_name%3D%27Takydromus+sexlineatus%27%29%29
61	101	Asian grass lizard	Takydromus sexlineatus	Squamata	Lizard	Vietnam	Asia	WC	https://reptile-database.reptarium.cz/species?genus=Takydromus&species=sexlineatus&search_param=%28%28common_name%3D%27Takydromus+sexlineatus%27%29%29
61	101	Asian grass lizard	Takydromus sexlineatus	Squamata	Lizard	Vietnam	Asia	WC	https://reptile-database.reptarium.cz/species?genus=Takydromus&species=sexlineatus&search_param=%28%28common_name%3D%27Takydromus+sexlineatus%27%29%29
61	101	Asian grass lizard	Takydromus sexlineatus	Squamata	Lizard	Vietnam	Asia	WC	https://reptile-database.reptarium.cz/species?genus=Takydromus&species=sexlineatus&search_param=%28%28common_name%3D%27Takydromus+sexlineatus%27%29%29
61	101	Asian grass lizard	Takydromus sexlineatus	Squamata	Lizard	Vietnam	Asia	WC	https://reptile-database.reptarium.cz/species?genus=Takydromus&species=sexlineatus&search_param=%28%28common_name%3D%27Takydromus+sexlineatus%27%29%29
61	101	Asian grass lizard	Takydromus sexlineatus	Squamata	Lizard	Vietnam	Asia	WC	https://reptile-database.reptarium.cz/species?genus=Takydromus&species=sexlineatus&search_param=%28%28common_name%3D%27Takydromus+sexlineatus%27%29%29
61	101	Asian grass lizard	Takydromus sexlineatus	Squamata	Lizard	Vietnam	Asia	WC	https://reptile-database.reptarium.cz/species?genus=Takydromus&species=sexlineatus&search_param=%28%28common_name%3D%27Takydromus+sexlineatus%27%29%29
61	101	Asian grass lizard	Takydromus sexlineatus	Squamata	Lizard	Vietnam	Asia	WC	https://reptile-database.reptarium.cz/species?genus=Takydromus&species=sexlineatus&search_param=%28%28common_name%3D%27Takydromus+sexlineatus%27%29%29
61	101	Asian grass lizard	Takydromus sexlineatus	Squamata	Lizard	Vietnam	Asia	WC	https://reptile-database.reptarium.cz/species?genus=Takydromus&species=sexlineatus&search_param=%28%28common_name%3D%27Takydromus+sexlineatus%27%29%29
61	101	Asian grass lizard	Takydromus sexlineatus	Squamata	Lizard	Vietnam	Asia	WC	https://reptile-database.reptarium.cz/species?genus=Takydromus&species=sexlineatus&search_param=%28%28common_name%3D%27Takydromus+sexlineatus%27%29%29

Table S1: Animal species sampled in this study - country origin and categorization as captive bred (CB), farm bred (FB) and wild-caught (WC)

Shipment No.	Sample batch	Animal species	Scientific name of animal/s	Order	Suborder	Country origin	Continent	Captive bred (CB), farm bred (FB), wild-caught (WC)	Web URL used for assigning animal species to categories CB, FB and WC
61	101	Asian grass lizard	Takydromus sexlineatus	Squamata	Lizard	Vietnam	Asia	WC	https://reptile-database.reptarium.cz/species?genus=Takydromus&species=sexlineatus&search_param=%28%28common_name%3D%27Takydromus+sexlineatus%27%29%29
61	102	Chinese water dragon	Physignatus cocincinus	Squamata	Lizard	Vietnam	Asia	WC	https://reptile-database.reptarium.cz/species?genus=Physignathus&species=cocincinus&search_param=%28%28common_name%3D%27gr%C3%BCne+wasseragame%27%29%29
61	102	Chinese water dragon	Physignatus cocincinus	Squamata	Lizard	Vietnam	Asia	WC	https://reptile-database.reptarium.cz/species?genus=Physignathus&species=cocincinus&search_param=%28%28common_name%3D%27gr%C3%BCne+wasseragame%27%29%29
61	102	Chinese water dragon	Physignatus cocincinus	Squamata	Lizard	Vietnam	Asia	WC	https://reptile-database.reptarium.cz/species?genus=Physignathus&species=cocincinus&search_param=%28%28common_name%3D%27gr%C3%BCne+wasseragame%27%29%29
61	102	Chinese water dragon	Physignatus cocincinus	Squamata	Lizard	Vietnam	Asia	WC	https://reptile-database.reptarium.cz/species?genus=Physignathus&species=cocincinus&search_param=%28%28common_name%3D%27gr%C3%BCne+wasseragame%27%29%29
61	102	Chinese water dragon	Physignatus cocincinus	Squamata	Lizard	Vietnam	Asia	WC	https://reptile-database.reptarium.cz/species?genus=Physignathus&species=cocincinus&search_param=%28%28common_name%3D%27gr%C3%BCne+wasseragame%27%29%29
61	102	Chinese water dragon	Physignatus cocincinus	Squamata	Lizard	Vietnam	Asia	WC	https://reptile-database.reptarium.cz/species?genus=Physignathus&species=cocincinus&search_param=%28%28common_name%3D%27gr%C3%BCne+wasseragame%27%29%29
61	102	Chinese water dragon	Physignatus cocincinus	Squamata	Lizard	Vietnam	Asia	WC	https://reptile-database.reptarium.cz/species?genus=Physignathus&species=cocincinus&search_param=%28%28common_name%3D%27gr%C3%BCne+wasseragame%27%29%29
61	102	Chinese water dragon	Physignatus cocincinus	Squamata	Lizard	Vietnam	Asia	WC	https://reptile-database.reptarium.cz/species?genus=Physignathus&species=cocincinus&search_param=%28%28common_name%3D%27gr%C3%BCne+wasseragame%27%29%29
61	102	Chinese water dragon	Physignatus cocincinus	Squamata	Lizard	Vietnam	Asia	WC	https://reptile-database.reptarium.cz/species?genus=Physignathus&species=cocincinus&search_param=%28%28common_name%3D%27gr%C3%BCne+wasseragame%27%29%29
61	102	Chinese water dragon	Physignatus cocincinus	Squamata	Lizard	Vietnam	Asia	WC	https://reptile-database.reptarium.cz/species?genus=Physignathus&species=cocincinus&search_param=%28%28common_name%3D%27gr%C3%BCne+wasseragame%27%29%29
61	102	Chinese water dragon	Physignatus cocincinus	Squamata	Lizard	Vietnam	Asia	WC	https://reptile-database.reptarium.cz/species?genus=Physignathus&species=cocincinus&search_param=%28%28common_name%3D%27gr%C3%BCne+wasseragame%27%29%29

Table S1: Animal species sampled in this study - country origin and categorization as captive bred (CB), farm bred (FB) and wild-caught (WC)

Shipment No.	Sample batch	Animal species	Scientific name of animal/s	Order	Suborder	Country origin	Continent	Captive bred (CB), farm bred (FB), wild-caught (WC)	Web URL used for assigning animal species to categories CB, FB and WC
61	103	Tokay gecko	Gekko gecko	Squamata	Lizard	Vietnam	Asia	WC	https://reptile-database.reptarium.cz/species?genus=Gekko&species=gecko&search_param=%28%28common_name%3D%27Gekko+gecko%27%29%29
61	103	Tokay gecko	Gekko gecko	Squamata	Lizard	Vietnam	Asia	WC	https://reptile-database.reptarium.cz/species?genus=Gekko&species=gecko&search_param=%28%28common_name%3D%27Gekko+gecko%27%29%29
61	103	Tokay gecko	Gekko gecko	Squamata	Lizard	Vietnam	Asia	WC	https://reptile-database.reptarium.cz/species?genus=Gekko&species=gecko&search_param=%28%28common_name%3D%27Gekko+gecko%27%29%29
61	103	Tokay gecko	Gekko gecko	Squamata	Lizard	Vietnam	Asia	WC	https://reptile-database.reptarium.cz/species?genus=Gekko&species=gecko&search_param=%28%28common_name%3D%27Gekko+gecko%27%29%29
61	103	Tokay gecko	Gekko gecko	Squamata	Lizard	Vietnam	Asia	WC	https://reptile-database.reptarium.cz/species?genus=Gekko&species=gecko&search_param=%28%28common_name%3D%27Gekko+gecko%27%29%29
62	104	Skink	Trachylepis	Squamata	Lizard	Vietnam	Asia	WC	
62	104	Skink	Trachylepis	Squamata	Lizard	Vietnam	Asia	WC	
62	104	Skink	Trachylepis	Squamata	Lizard	Vietnam	Asia	WC	
62	104	Skink	Trachylepis	Squamata	Lizard	Vietnam	Asia	WC	
62	104	Skink	Trachylepis	Squamata	Lizard	Vietnam	Asia	WC	
62	105	Chinese water dragon	Physignatus cocincinus	Squamata	Lizard	Vietnam	Asia	WC	https://reptile-database.reptarium.cz/species?genus=Physignathus&species=cocincinus&search_param=%28%28common_name%3D%27gr%C3%BCne+wasseragame%27%29%29
62	105	Chinese water dragon	Physignatus cocincinus	Squamata	Lizard	Vietnam	Asia	WC	https://reptile-database.reptarium.cz/species?genus=Physignathus&species=cocincinus&search_param=%28%28common_name%3D%27gr%C3%BCne+wasseragame%27%29%29
62	105	Chinese water dragon	Physignatus cocincinus	Squamata	Lizard	Vietnam	Asia	WC	https://reptile-database.reptarium.cz/species?genus=Physignathus&species=cocincinus&search_param=%28%28common_name%3D%27gr%C3%BCne+wasseragame%27%29%29
62	105	Chinese water dragon	Physignatus cocincinus	Squamata	Lizard	Vietnam	Asia	WC	https://reptile-database.reptarium.cz/species?genus=Physignathus&species=cocincinus&search_param=%28%28common_name%3D%27gr%C3%BCne+wasseragame%27%29%29
62	105	Chinese water dragon	Physignatus cocincinus	Squamata	Lizard	Vietnam	Asia	WC	https://reptile-database.reptarium.cz/species?genus=Physignathus&species=cocincinus&search_param=%28%28common_name%3D%27gr%C3%BCne+wasseragame%27%29%29
62	105	Chinese water dragon	Physignatus cocincinus	Squamata	Lizard	Vietnam	Asia	WC	https://reptile-database.reptarium.cz/species?genus=Physignathus&species=cocincinus&search_param=%28%28common_name%3D%27gr%C3%BCne+wasseragame%27%29%29
62	105	Chinese water dragon	Physignatus cocincinus	Squamata	Lizard	Vietnam	Asia	WC	https://reptile-database.reptarium.cz/species?genus=Physignathus&species=cocincinus&search_param=%28%28common_name%3D%27gr%C3%BCne+wasseragame%27%29%29

Table S1: Animal species sampled in this study - country origin and categorization as captive bred (CB), farm bred (FB) and wild-caught (WC)

Shipment No.	Sample batch	Animal species	Scientific name of animal/s	Order	Suborder	Country origin	Continent	Captive bred (CB), farm bred (FB), wild-caught (WC)	Web URL used for assigning animal species to categories CB, FB and WC
62	105	Chinese water dragon	Physignatus cocincinus	Squamata	Lizard	Vietnam	Asia	WC	https://reptile-database.reptarium.cz/species?genus=Physignathus&species=cocincinus&search_param=%28%28common_name%3D%27gr%C3%BCne+wasseragame%27%29%29
62	105	Chinese water dragon	Physignatus cocincinus	Squamata	Lizard	Vietnam	Asia	WC	https://reptile-database.reptarium.cz/species?genus=Physignathus&species=cocincinus&search_param=%28%28common_name%3D%27gr%C3%BCne+wasseragame%27%29%29
62	105	Chinese water dragon	Physignatus cocincinus	Squamata	Lizard	Vietnam	Asia	WC	https://reptile-database.reptarium.cz/species?genus=Physignathus&species=cocincinus&search_param=%28%28common_name%3D%27gr%C3%BCne+wasseragame%27%29%29
62	105	Chinese water dragon	Physignatus cocincinus	Squamata	Lizard	Vietnam	Asia	WC	https://reptile-database.reptarium.cz/species?genus=Physignathus&species=cocincinus&search_param=%28%28common_name%3D%27gr%C3%BCne+wasseragame%27%29%29
62	106	Gecko	(Gekkonidae)	Squamata	Lizard	Vietnam	Asia	WC	
62	106	Gecko	(Gekkonidae)	Squamata	Lizard	Vietnam	Asia	WC	
62	106	Gecko	(Gekkonidae)	Squamata	Lizard	Vietnam	Asia	WC	
62	106	Gecko	(Gekkonidae)	Squamata	Lizard	Vietnam	Asia	WC	
62	106	Gecko	(Gekkonidae)	Squamata	Lizard	Vietnam	Asia	WC	
62	106	Gecko	(Gekkonidae)	Squamata	Lizard	Vietnam	Asia	WC	
62	106	Gecko	(Gekkonidae)	Squamata	Lizard	Vietnam	Asia	WC	
62	106	Gecko	(Gekkonidae)	Squamata	Lizard	Vietnam	Asia	WC	
62	106	Gecko	(Gekkonidae)	Squamata	Lizard	Vietnam	Asia	WC	
62	106	Gecko	(Gekkonidae)	Squamata	Lizard	Vietnam	Asia	WC	
62	106	Gecko	(Gekkonidae)	Squamata	Lizard	Vietnam	Asia	WC	
62	106	Gecko	(Gekkonidae)	Squamata	Lizard	Vietnam	Asia	WC	
62	106	Gecko	(Gekkonidae)	Squamata	Lizard	Vietnam	Asia	WC	
62	107	Pricklenape	Acanthosaura sp.	Squamata	Lizard	Vietnam	Asia	WC	https://reptile-database.reptarium.cz/advanced_search?common_name=Acanthosaura&submit=Search
62	107	Pricklenape	Acanthosaura sp.	Squamata	Lizard	Vietnam	Asia	WC	https://reptile-database.reptarium.cz/advanced_search?common_name=Acanthosaura&submit=Search
62	107	Pricklenape	Acanthosaura sp.	Squamata	Lizard	Vietnam	Asia	WC	https://reptile-database.reptarium.cz/advanced_search?common_name=Acanthosaura&submit=Search
62	107	Pricklenape	Acanthosaura sp.	Squamata	Lizard	Vietnam	Asia	WC	https://reptile-database.reptarium.cz/advanced_search?common_name=Acanthosaura&submit=Search
62	107	Pricklenape	Acanthosaura sp.	Squamata	Lizard	Vietnam	Asia	WC	https://reptile-database.reptarium.cz/advanced_search?common_name=Acanthosaura&submit=Search
62	107	Pricklenape	Acanthosaura sp.	Squamata	Lizard	Vietnam	Asia	WC	https://reptile-database.reptarium.cz/advanced_search?common_name=Acanthosaura&submit=Search

Table S1: Animal species sampled in this study - country origin and categorization as captive bred (CB), farm bred (FB) and wild-caught (WC)

Shipment No.	Sample batch	Animal species	Scientific name of animal/s	Order	Suborder	Country origin	Continent	Captive bred (CB), farm bred (FB), wild-caught (WC)	Web URL used for assigning animal species to categories CB, FB and WC
62	108	Asian grass lizard	Takydromus sexlineatus	Squamata	Lizard	Vietnam	Asia	WC	https://reptile-database.reptarium.cz/species?genus=Takydromus&species=sexlineatus&search_param=%28%28common_name%3D%27Takydromus+sexlineatus%27%29%29
62	108	Asian grass lizard	Takydromus sexlineatus	Squamata	Lizard	Vietnam	Asia	WC	https://reptile-database.reptarium.cz/species?genus=Takydromus&species=sexlineatus&search_param=%28%28common_name%3D%27Takydromus+sexlineatus%27%29%29
62	108	Asian grass lizard	Takydromus sexlineatus	Squamata	Lizard	Vietnam	Asia	WC	https://reptile-database.reptarium.cz/species?genus=Takydromus&species=sexlineatus&search_param=%28%28common_name%3D%27Takydromus+sexlineatus%27%29%29
62	108	Asian grass lizard	Takydromus sexlineatus	Squamata	Lizard	Vietnam	Asia	WC	https://reptile-database.reptarium.cz/species?genus=Takydromus&species=sexlineatus&search_param=%28%28common_name%3D%27Takydromus+sexlineatus%27%29%29
62	108	Asian grass lizard	Takydromus sexlineatus	Squamata	Lizard	Vietnam	Asia	WC	https://reptile-database.reptarium.cz/species?genus=Takydromus&species=sexlineatus&search_param=%28%28common_name%3D%27Takydromus+sexlineatus%27%29%29
62	108	Asian grass lizard	Takydromus sexlineatus	Squamata	Lizard	Vietnam	Asia	WC	https://reptile-database.reptarium.cz/species?genus=Takydromus&species=sexlineatus&search_param=%28%28common_name%3D%27Takydromus+sexlineatus%27%29%29
62	108	Asian grass lizard	Takydromus sexlineatus	Squamata	Lizard	Vietnam	Asia	WC	https://reptile-database.reptarium.cz/species?genus=Takydromus&species=sexlineatus&search_param=%28%28common_name%3D%27Takydromus+sexlineatus%27%29%29
62	108	Asian grass lizard	Takydromus sexlineatus	Squamata	Lizard	Vietnam	Asia	WC	https://reptile-database.reptarium.cz/species?genus=Takydromus&species=sexlineatus&search_param=%28%28common_name%3D%27Takydromus+sexlineatus%27%29%29
63	109	Mediterranean spur-thighed tortoise & Marginated tortoise	Testudo graeca & T.marginata	Testudines	Turtle	Turkey	Asia	WC/FB	https://www.academia.edu/4668767/Testudo_trade_in_Turkey
63	109	Mediterranean spur-thighed tortoise & Marginated tortoise	Testudo graeca & T.marginata	Testudines	Turtle	Turkey	Asia	WC/FB	https://www.academia.edu/4668767/Testudo_trade_in_Turkey
63	109	Mediterranean spur-thighed tortoise & Marginated tortoise	Testudo graeca & T.marginata	Testudines	Turtle	Turkey	Asia	WC/FB	https://www.academia.edu/4668767/Testudo_trade_in_Turkey
63	109	Mediterranean spur-thighed tortoise & Marginated tortoise	Testudo graeca & T.marginata	Testudines	Turtle	Turkey	Asia	WC/FB	https://www.academia.edu/4668767/Testudo_trade_in_Turkey
63	109	Mediterranean spur-thighed tortoise & Marginated tortoise	Testudo graeca & T.marginata	Testudines	Turtle	Turkey	Asia	WC/FB	https://www.academia.edu/4668767/Testudo_trade_in_Turkey

Table S1: Animal species sampled in this study - country origin and categorization as captive bred (CB), farm bred (FB) and wild-caught (WC)

Shipment No.	Sample batch	Animal species	Scientific name of animal/s	Order	Suborder	Country origin	Continent	Captive bred (CB), farm bred (FB), wild-caught (WC)	Web URL used for assigning animal species to categories CB, FB and WC
64	110	East African black mud turtle	Pelusios subniger	Testudines	Turtle	Mozambique	Africa	WC	https://reptile-database.reptarium.cz/species?genus=Pelusios&species=subniger&search_param=%28%28common_name%3D%27Pelusios+subniger%27%29%29
64	110	East African black mud turtle	Pelusios subniger	Testudines	Turtle	Mozambique	Africa	WC	https://reptile-database.reptarium.cz/species?genus=Pelusios&species=subniger&search_param=%28%28common_name%3D%27Pelusios+subniger%27%29%29
64	110	East African black mud turtle	Pelusios subniger	Testudines	Turtle	Mozambique	Africa	WC	https://reptile-database.reptarium.cz/species?genus=Pelusios&species=subniger&search_param=%28%28common_name%3D%27Pelusios+subniger%27%29%29
64	110	East African black mud turtle	Pelusios subniger	Testudines	Turtle	Mozambique	Africa	WC	https://reptile-database.reptarium.cz/species?genus=Pelusios&species=subniger&search_param=%28%28common_name%3D%27Pelusios+subniger%27%29%29
64	110	East African black mud turtle	Pelusios subniger	Testudines	Turtle	Mozambique	Africa	WC	https://reptile-database.reptarium.cz/species?genus=Pelusios&species=subniger&search_param=%28%28common_name%3D%27Pelusios+subniger%27%29%29
64	110	East African black mud turtle	Pelusios subniger	Testudines	Turtle	Mozambique	Africa	WC	https://reptile-database.reptarium.cz/species?genus=Pelusios&species=subniger&search_param=%28%28common_name%3D%27Pelusios+subniger%27%29%29
65	111	Leopard tortoise	Stigmochelys pardalis	Testudines	Turtle	Ecuador	America	FB	https://reptile-database.reptarium.cz/species?genus=Stigmochelys&species=pardalis&search_param=%28%28common_name%3D%27Stigmochelys+pardalis%27%29%29
65	111	Leopard tortoise	Stigmochelys pardalis	Testudines	Turtle	Ecuador	America	FB	https://reptile-database.reptarium.cz/species?genus=Stigmochelys&species=pardalis&search_param=%28%28common_name%3D%27Stigmochelys+pardalis%27%29%29
65	111	Leopard tortoise	Stigmochelys pardalis	Testudines	Turtle	Ecuador	America	FB	https://reptile-database.reptarium.cz/species?genus=Stigmochelys&species=pardalis&search_param=%28%28common_name%3D%27Stigmochelys+pardalis%27%29%29
65	111	Leopard tortoise	Stigmochelys pardalis	Testudines	Turtle	Ecuador	America	FB	https://reptile-database.reptarium.cz/species?genus=Stigmochelys&species=pardalis&search_param=%28%28common_name%3D%27Stigmochelys+pardalis%27%29%29
65	111	Leopard tortoise	Stigmochelys pardalis	Testudines	Turtle	Ecuador	America	FB	https://reptile-database.reptarium.cz/species?genus=Stigmochelys&species=pardalis&search_param=%28%28common_name%3D%27Stigmochelys+pardalis%27%29%29
65	111	Leopard tortoise	Stigmochelys pardalis	Testudines	Turtle	Ecuador	America	FB	https://reptile-database.reptarium.cz/species?genus=Stigmochelys&species=pardalis&search_param=%28%28common_name%3D%27Stigmochelys+pardalis%27%29%29

Table S1: Animal species sampled in this study - country origin and categorization as captive bred (CB), farm bred (FB) and wild-caught (WC)

Shipment No.	Sample batch	Animal species	Scientific name of animal/s	Order	Suborder	Country origin	Continent	Captive bred (CB), farm bred (FB), wild-caught (WC)	Web URL used for assigning animal species to categories CB, FB and WC
65	111	Leopard tortoise	Stigmochelys pardalis	Testudines	Turtle	Ecuador	America	FB	https://reptile-database.reptarium.cz/species?genus=Stigmochelys&species=pardalis&search_param=%28%28common_name%3D%27Stigmochelys+pardalis%27%29%29
65	111	Leopard tortoise	Stigmochelys pardalis	Testudines	Turtle	Ecuador	America	FB	https://reptile-database.reptarium.cz/species?genus=Stigmochelys&species=pardalis&search_param=%28%28common_name%3D%27Stigmochelys+pardalis%27%29%29
65	111	Leopard tortoise	Stigmochelys pardalis	Testudines	Turtle	Ecuador	America	FB	https://reptile-database.reptarium.cz/species?genus=Stigmochelys&species=pardalis&search_param=%28%28common_name%3D%27Stigmochelys+pardalis%27%29%29
65	111	Leopard tortoise	Stigmochelys pardalis	Testudines	Turtle	Ecuador	America	FB	https://reptile-database.reptarium.cz/species?genus=Stigmochelys&species=pardalis&search_param=%28%28common_name%3D%27Stigmochelys+pardalis%27%29%29
65	112	Central bearded dragon	Pogona vitticeps	Squamata	Lizard	Ecuador	America	CB	https://reptile-database.reptarium.cz/species?genus=Pogona&species=vitticeps&search_param=%28%28common_name%3D%27Pogona+vitticeps%27%29%29
65	112	Central bearded dragon	Pogona vitticeps	Squamata	Lizard	Ecuador	America	CB	https://reptile-database.reptarium.cz/species?genus=Pogona&species=vitticeps&search_param=%28%28common_name%3D%27Pogona+vitticeps%27%29%29
65	112	Central bearded dragon	Pogona vitticeps	Squamata	Lizard	Ecuador	America	CB	https://reptile-database.reptarium.cz/species?genus=Pogona&species=vitticeps&search_param=%28%28common_name%3D%27Pogona+vitticeps%27%29%29
65	112	Central bearded dragon	Pogona vitticeps	Squamata	Lizard	Ecuador	America	CB	https://reptile-database.reptarium.cz/species?genus=Pogona&species=vitticeps&search_param=%28%28common_name%3D%27Pogona+vitticeps%27%29%29
65	112	Central bearded dragon	Pogona vitticeps	Squamata	Lizard	Ecuador	America	CB	https://reptile-database.reptarium.cz/species?genus=Pogona&species=vitticeps&search_param=%28%28common_name%3D%27Pogona+vitticeps%27%29%29
65	112	Central bearded dragon	Pogona vitticeps	Squamata	Lizard	Ecuador	America	CB	https://reptile-database.reptarium.cz/species?genus=Pogona&species=vitticeps&search_param=%28%28common_name%3D%27Pogona+vitticeps%27%29%29
65	112	Central bearded dragon	Pogona vitticeps	Squamata	Lizard	Ecuador	America	CB	https://reptile-database.reptarium.cz/species?genus=Pogona&species=vitticeps&search_param=%28%28common_name%3D%27Pogona+vitticeps%27%29%29
65	112	Central bearded dragon	Pogona vitticeps	Squamata	Lizard	Ecuador	America	CB	https://reptile-database.reptarium.cz/species?genus=Pogona&species=vitticeps&search_param=%28%28common_name%3D%27Pogona+vitticeps%27%29%29

Table S1: Animal species sampled in this study - country origin and categorization as captive bred (CB), farm bred (FB) and wild-caught (WC)

Shipment No.	Sample batch	Animal species	Scientific name of animal/s	Order	Suborder	Country origin	Continent	Captive bred (CB), farm bred (FB), wild-caught (WC)	Web URL used for assigning animal species to categories CB, FB and WC
65	112	Central bearded dragon	Pogona vitticeps	Squamata	Lizard	Ecuador	America	CB	https://reptile-database.reptarium.cz/species?genus=Pogona&species=vitticeps&search_param=%28%28common_name%3D%27Pogona+vitticeps%27%29%29
66	113	Yellow-headed gecko	Gonatodes albogularis	Squamata	Lizard	Nicaragua	America	WC	https://reptile-database.reptarium.cz/species?genus=Gonatodes&species=albogularis&search_param=%28%28common_name%3D%27Gonatodes+albogularis%27%29%29
66	113	Yellow-headed gecko	Gonatodes albogularis	Squamata	Lizard	Nicaragua	America	WC	https://reptile-database.reptarium.cz/species?genus=Gonatodes&species=albogularis&search_param=%28%28common_name%3D%27Gonatodes+albogularis%27%29%29
66	113	Yellow-headed gecko	Gonatodes albogularis	Squamata	Lizard	Nicaragua	America	WC	https://reptile-database.reptarium.cz/species?genus=Gonatodes&species=albogularis&search_param=%28%28common_name%3D%27Gonatodes+albogularis%27%29%29
66	113	Yellow-headed gecko	Gonatodes albogularis	Squamata	Lizard	Nicaragua	America	WC	https://reptile-database.reptarium.cz/species?genus=Gonatodes&species=albogularis&search_param=%28%28common_name%3D%27Gonatodes+albogularis%27%29%29
66	113	Yellow-headed gecko	Gonatodes albogularis	Squamata	Lizard	Nicaragua	America	WC	https://reptile-database.reptarium.cz/species?genus=Gonatodes&species=albogularis&search_param=%28%28common_name%3D%27Gonatodes+albogularis%27%29%29
66	113	Yellow-headed gecko	Gonatodes albogularis	Squamata	Lizard	Nicaragua	America	WC	https://reptile-database.reptarium.cz/species?genus=Gonatodes&species=albogularis&search_param=%28%28common_name%3D%27Gonatodes+albogularis%27%29%29
67	114	Horsefield's tortoise	Testudo horsfieldii	Testudines	Turtle	Uzbekistan	Asia	FB	https://reptile-database.reptarium.cz/species?genus=Testudo&species=horsfieldii&search_param=%28%28common_name%3D%27Testudo+horsfieldii%27%29%29
67	114	Horsefield's tortoise	Testudo horsfieldii	Testudines	Turtle	Uzbekistan	Asia	FB	https://reptile-database.reptarium.cz/species?genus=Testudo&species=horsfieldii&search_param=%28%28common_name%3D%27Testudo+horsfieldii%27%29%29
67	114	Horsefield's tortoise	Testudo horsfieldii	Testudines	Turtle	Uzbekistan	Asia	FB	https://reptile-database.reptarium.cz/species?genus=Testudo&species=horsfieldii&search_param=%28%28common_name%3D%27Testudo+horsfieldii%27%29%29
67	114	Horsefield's tortoise	Testudo horsfieldii	Testudines	Turtle	Uzbekistan	Asia	FB	https://reptile-database.reptarium.cz/species?genus=Testudo&species=horsfieldii&search_param=%28%28common_name%3D%27Testudo+horsfieldii%27%29%29
67	114	Horsefield's tortoise	Testudo horsfieldii	Testudines	Turtle	Uzbekistan	Asia	FB	https://reptile-database.reptarium.cz/species?genus=Testudo&species=horsfieldii&search_param=%28%28common_name%3D%27Testudo+horsfieldii%27%29%29

Table S1: Animal species sampled in this study - country origin and categorization as captive bred (CB), farm bred (FB) and wild-caught (WC)

Shipment No.	Sample batch	Animal species	Scientific name of animal/s	Order	Suborder	Country origin	Continent	Captive bred (CB), farm bred (FB), wild-caught (WC)	Web URL used for assigning animal species to categories CB, FB and WC
67	114	Horsefield's tortoise	Testudo horsfieldii	Testudines	Turtle	Uzbekistan	Asia	FB	https://reptile-database.reptarium.cz/species?genus=Testudo&species=horsfieldii&search_param=%28%28common_name%3D%27Testudo+horsfieldii%27%29%29
67	114	Horsefield's tortoise	Testudo horsfieldii	Testudines	Turtle	Uzbekistan	Asia	FB	https://reptile-database.reptarium.cz/species?genus=Testudo&species=horsfieldii&search_param=%28%28common_name%3D%27Testudo+horsfieldii%27%29%29
67	114	Horsefield's tortoise	Testudo horsfieldii	Testudines	Turtle	Uzbekistan	Asia	FB	https://reptile-database.reptarium.cz/species?genus=Testudo&species=horsfieldii&search_param=%28%28common_name%3D%27Testudo+horsfieldii%27%29%29
68	115	Jackson's chameleon	Trioceros jacksonii	Squamata	Lizard	Uganda	Africa	CB	https://reptile-database.reptarium.cz/species?genus=Trioceros&species=jacksonii&search_param=%28%28common_name%3D%27Trioceros+jacksonii%27%29%29
68	115	Jackson's chameleon	Trioceros jacksonii	Squamata	Lizard	Uganda	Africa	CB	https://reptile-database.reptarium.cz/species?genus=Trioceros&species=jacksonii&search_param=%28%28common_name%3D%27Trioceros+jacksonii%27%29%29
68	115	Jackson's chameleon	Trioceros jacksonii	Squamata	Lizard	Uganda	Africa	CB	https://reptile-database.reptarium.cz/species?genus=Trioceros&species=jacksonii&search_param=%28%28common_name%3D%27Trioceros+jacksonii%27%29%29
68	115	Jackson's chameleon	Trioceros jacksonii	Squamata	Lizard	Uganda	Africa	CB	https://reptile-database.reptarium.cz/species?genus=Trioceros&species=jacksonii&search_param=%28%28common_name%3D%27Trioceros+jacksonii%27%29%29
68	115	Jackson's chameleon	Trioceros jacksonii	Squamata	Lizard	Uganda	Africa	CB	https://reptile-database.reptarium.cz/species?genus=Trioceros&species=jacksonii&search_param=%28%28common_name%3D%27Trioceros+jacksonii%27%29%29
68	115	Jackson's chameleon	Trioceros jacksonii	Squamata	Lizard	Uganda	Africa	CB	https://reptile-database.reptarium.cz/species?genus=Trioceros&species=jacksonii&search_param=%28%28common_name%3D%27Trioceros+jacksonii%27%29%29
69	116	Cuvier's Madagascar swift	Oplurus cuvieri	Squamata	Lizard	Madagascar	Africa	WC	https://reptile-database.reptarium.cz/species?genus=Oplurus&species=cuvieri&search_param=%28%28common_name%3D%27Oplurus+cuvieri%27%29%29
69	116	Cuvier's Madagascar swift	Oplurus cuvieri	Squamata	Lizard	Madagascar	Africa	WC	https://reptile-database.reptarium.cz/species?genus=Oplurus&species=cuvieri&search_param=%28%28common_name%3D%27Oplurus+cuvieri%27%29%29
69	116	Cuvier's Madagascar swift	Oplurus cuvieri	Squamata	Lizard	Madagascar	Africa	WC	https://reptile-database.reptarium.cz/species?genus=Oplurus&species=cuvieri&search_param=%28%28common_name%3D%27Oplurus+cuvieri%27%29%29

Table S1: Animal species sampled in this study - country origin and categorization as captive bred (CB), farm bred (FB) and wild-caught (WC)

Shipment No.	Sample batch	Animal species	Scientific name of animal/s	Order	Suborder	Country origin	Continent	Captive bred (CB), farm bred (FB), wild-caught (WC)	Web URL used for assigning animal species to categories CB, FB and WC
69	116	Cuvier's Madagascar swift	Oplurus cuvieri	Squamata	Lizard	Madagascar	Africa	WC	https://reptile-database.reptarium.cz/species?genus=Oplurus&species=cuvieri&search_param=%28%28common_name%3D%27Oplurus+cuvieri%27%29%29
69	116	Cuvier's Madagascar swift	Oplurus cuvieri	Squamata	Lizard	Madagascar	Africa	WC	https://reptile-database.reptarium.cz/species?genus=Oplurus&species=cuvieri&search_param=%28%28common_name%3D%27Oplurus+cuvieri%27%29%29
69	116	Cuvier's Madagascar swift	Oplurus cuvieri	Squamata	Lizard	Madagascar	Africa	WC	https://reptile-database.reptarium.cz/species?genus=Oplurus&species=cuvieri&search_param=%28%28common_name%3D%27Oplurus+cuvieri%27%29%29
69	116	Cuvier's Madagascar swift	Oplurus cuvieri	Squamata	Lizard	Madagascar	Africa	WC	https://reptile-database.reptarium.cz/species?genus=Oplurus&species=cuvieri&search_param=%28%28common_name%3D%27Oplurus+cuvieri%27%29%29
69	116	Cuvier's Madagascar swift	Oplurus cuvieri	Squamata	Lizard	Madagascar	Africa	WC	https://reptile-database.reptarium.cz/species?genus=Oplurus&species=cuvieri&search_param=%28%28common_name%3D%27Oplurus+cuvieri%27%29%29
69	116	Cuvier's Madagascar swift	Oplurus cuvieri	Squamata	Lizard	Madagascar	Africa	WC	https://reptile-database.reptarium.cz/species?genus=Oplurus&species=cuvieri&search_param=%28%28common_name%3D%27Oplurus+cuvieri%27%29%29
69	117	Southeastern girdled lizard	Zonosaurus maximus	Squamata	Lizard	Madagascar	Africa	WC	https://reptile-database.reptarium.cz/species?genus=Zonosaurus&species=maximus&search_param=%28%28common_name%3D%27Zonosaurus+maximus%27%29%29
69	117	Southeastern girdled lizard	Zonosaurus maximus	Squamata	Lizard	Madagascar	Africa	WC	https://reptile-database.reptarium.cz/species?genus=Zonosaurus&species=maximus&search_param=%28%28common_name%3D%27Zonosaurus+maximus%27%29%29
69	117	Southeastern girdled lizard	Zonosaurus maximus	Squamata	Lizard	Madagascar	Africa	WC	https://reptile-database.reptarium.cz/species?genus=Zonosaurus&species=maximus&search_param=%28%28common_name%3D%27Zonosaurus+maximus%27%29%29
69	117	Southeastern girdled lizard	Zonosaurus maximus	Squamata	Lizard	Madagascar	Africa	WC	https://reptile-database.reptarium.cz/species?genus=Zonosaurus&species=maximus&search_param=%28%28common_name%3D%27Zonosaurus+maximus%27%29%29
69	117	Southeastern girdled lizard	Zonosaurus maximus	Squamata	Lizard	Madagascar	Africa	WC	https://reptile-database.reptarium.cz/species?genus=Zonosaurus&species=maximus&search_param=%28%28common_name%3D%27Zonosaurus+maximus%27%29%29
69	117	Southeastern girdled lizard	Zonosaurus maximus	Squamata	Lizard	Madagascar	Africa	WC	https://reptile-database.reptarium.cz/species?genus=Zonosaurus&species=maximus&search_param=%28%28common_name%3D%27Zonosaurus+maximus%27%29%29

Table S1: Animal species sampled in this study - country origin and categorization as captive bred (CB), farm bred (FB) and wild-caught (WC)

Shipment No.	Sample batch	Animal species	Scientific name of animal/s	Order	Suborder	Country origin	Continent	Captive bred (CB), farm bred (FB), wild-caught (WC)	Web URL used for assigning animal species to categories CB, FB and WC
69	117	Southeastern girdled lizard	Zonosaurus maximus	Squamata	Lizard	Madagascar	Africa	WC	https://reptile-database.reptarium.cz/species?genus=Zonosaurus&species=maximus&search_param=%28%28common_name%3D%27Zonosaurus+maximus%27%29%29
70	118	Boa constrictor	Boa constrictor	Squamata	Snake	USA	America	CB	https://reptile-database.reptarium.cz/species?genus=Boa&species=constrictor&search_param=%28%28common_name%3D%27Boa+constrictor%27%29%29
70	118	Boa constrictor	Boa constrictor	Squamata	Snake	USA	America	CB	https://reptile-database.reptarium.cz/species?genus=Boa&species=constrictor&search_param=%28%28common_name%3D%27Boa+constrictor%27%29%29
70	118	Boa constrictor	Boa constrictor	Squamata	Snake	USA	America	CB	https://reptile-database.reptarium.cz/species?genus=Boa&species=constrictor&search_param=%28%28common_name%3D%27Boa+constrictor%27%29%29
70	118	Boa constrictor	Boa constrictor	Squamata	Snake	USA	America	CB	https://reptile-database.reptarium.cz/species?genus=Boa&species=constrictor&search_param=%28%28common_name%3D%27Boa+constrictor%27%29%29
70	118	Boa constrictor	Boa constrictor	Squamata	Snake	USA	America	CB	https://reptile-database.reptarium.cz/species?genus=Boa&species=constrictor&search_param=%28%28common_name%3D%27Boa+constrictor%27%29%29
71	119	Rough greensnake	Opheodrys aestivus	Squamata	Snake	USA	America	WC	https://reptile-database.reptarium.cz/species?genus=Opheodrys&species=aestivus&search_param=%28%28common_name%3D%27Opheodrys+aestivus%27%29%29
71	119	Rough greensnake	Opheodrys aestivus	Squamata	Snake	USA	America	WC	https://reptile-database.reptarium.cz/species?genus=Opheodrys&species=aestivus&search_param=%28%28common_name%3D%27Opheodrys+aestivus%27%29%29
71	119	Rough greensnake	Opheodrys aestivus	Squamata	Snake	USA	America	WC	https://reptile-database.reptarium.cz/species?genus=Opheodrys&species=aestivus&search_param=%28%28common_name%3D%27Opheodrys+aestivus%27%29%29
71	119	Rough greensnake	Opheodrys aestivus	Squamata	Snake	USA	America	WC	https://reptile-database.reptarium.cz/species?genus=Opheodrys&species=aestivus&search_param=%28%28common_name%3D%27Opheodrys+aestivus%27%29%29
71	119	Rough greensnake	Opheodrys aestivus	Squamata	Snake	USA	America	WC	https://reptile-database.reptarium.cz/species?genus=Opheodrys&species=aestivus&search_param=%28%28common_name%3D%27Opheodrys+aestivus%27%29%29
71	120	Painted wood turtle	Rhinoclemmys pulcherrima	Testudines	Turtle	USA	America	presumably CB	https://reptile-database.reptarium.cz/species?genus=Rhinoclemmys&species=pulcherrima&search_param=%28%28common_name%3D%27Rhinoclemmys+pulcherrima%27%29%29

Table S1: Animal species sampled in this study - country origin and categorization as captive bred (CB), farm bred (FB) and wild-caught (WC)

Shipment No.	Sample batch	Animal species	Scientific name of animal/s	Order	Suborder	Country origin	Continent	Captive bred (CB), farm bred (FB), wild-caught (WC)	Web URL used for assigning animal species to categories CB, FB and WC
71	120	Painted wood turtle	Rhinoclemmys pulcherrima	Testudines	Turtle	USA	America	presumably CB	https://reptile-database.reptarium.cz/species?genus=Rhinoclemmys&species=pulcherrima&search_param=%28%28common_name%3D%27Rhinoclemmys+pulcherrima%27%29%29
71	120	Painted wood turtle	Rhinoclemmys pulcherrima	Testudines	Turtle	USA	America	presumably CB	https://reptile-database.reptarium.cz/species?genus=Rhinoclemmys&species=pulcherrima&search_param=%28%28common_name%3D%27Rhinoclemmys+pulcherrima%27%29%29
71	120	Painted wood turtle	Rhinoclemmys pulcherrima	Testudines	Turtle	USA	America	presumably CB	https://reptile-database.reptarium.cz/species?genus=Rhinoclemmys&species=pulcherrima&search_param=%28%28common_name%3D%27Rhinoclemmys+pulcherrima%27%29%29
71	120	Painted wood turtle	Rhinoclemmys pulcherrima	Testudines	Turtle	USA	America	presumably CB	https://reptile-database.reptarium.cz/species?genus=Rhinoclemmys&species=pulcherrima&search_param=%28%28common_name%3D%27Rhinoclemmys+pulcherrima%27%29%29
71	120	Painted wood turtle	Rhinoclemmys pulcherrima	Testudines	Turtle	USA	America	presumably CB	https://reptile-database.reptarium.cz/species?genus=Rhinoclemmys&species=pulcherrima&search_param=%28%28common_name%3D%27Rhinoclemmys+pulcherrima%27%29%29
71	120	Painted wood turtle	Rhinoclemmys pulcherrima	Testudines	Turtle	USA	America	presumably CB	https://reptile-database.reptarium.cz/species?genus=Rhinoclemmys&species=pulcherrima&search_param=%28%28common_name%3D%27Rhinoclemmys+pulcherrima%27%29%29
71	120	Painted wood turtle	Rhinoclemmys pulcherrima	Testudines	Turtle	USA	America	presumably CB	https://reptile-database.reptarium.cz/species?genus=Rhinoclemmys&species=pulcherrima&search_param=%28%28common_name%3D%27Rhinoclemmys+pulcherrima%27%29%29
71	121	Yellow-headed gecko	Gonatodes albogularis	Squamata	Lizard	USA	America	WC	https://reptile-database.reptarium.cz/species?genus=Gonatodes&species=albogularis&search_param=%28%28common_name%3D%27Gonatodes+albogularis%27%29%29
71	121	Yellow-headed gecko	Gonatodes albogularis	Squamata	Lizard	USA	America	WC	https://reptile-database.reptarium.cz/species?genus=Gonatodes&species=albogularis&search_param=%28%28common_name%3D%27Gonatodes+albogularis%27%29%29
71	121	Yellow-headed gecko	Gonatodes albogularis	Squamata	Lizard	USA	America	WC	https://reptile-database.reptarium.cz/species?genus=Gonatodes&species=albogularis&search_param=%28%28common_name%3D%27Gonatodes+albogularis%27%29%29
71	121	Yellow-headed gecko	Gonatodes albogularis	Squamata	Lizard	USA	America	WC	https://reptile-database.reptarium.cz/species?genus=Gonatodes&species=albogularis&search_param=%28%28common_name%3D%27Gonatodes+albogularis%27%29%29
72	122	Common green iguana	Iguana iguana	Squamata	Lizard	El Salvador	America	FB	https://reptile-database.reptarium.cz/species?genus=Iguana&species=iguana&search_param=%28%28common_name%3D%27Iguana+iguana%27%29%29

Table S1: Animal species sampled in this study - country origin and categorization as captive bred (CB), farm bred (FB) and wild-caught (WC)

Shipment No.	Sample batch	Animal species	Scientific name of animal/s	Order	Suborder	Country origin	Continent	Captive bred (CB), farm bred (FB), wild-caught (WC)	Web URL used for assigning animal species to categories CB, FB and WC
72	122	Common green iguana	Iguana iguana	Squamata	Lizard	El Salvador	America	FB	https://reptile-database.reptarium.cz/species?genus=Iguana&species=iguana&search_param=%28%28common_name%3D%27Iguana+iguana%27%29%29
72	122	Common green iguana	Iguana iguana	Squamata	Lizard	El Salvador	America	FB	https://reptile-database.reptarium.cz/species?genus=Iguana&species=iguana&search_param=%28%28common_name%3D%27Iguana+iguana%27%29%29
72	122	Common green iguana	Iguana iguana	Squamata	Lizard	El Salvador	America	FB	https://reptile-database.reptarium.cz/species?genus=Iguana&species=iguana&search_param=%28%28common_name%3D%27Iguana+iguana%27%29%29
72	122	Common green iguana	Iguana iguana	Squamata	Lizard	El Salvador	America	FB	https://reptile-database.reptarium.cz/species?genus=Iguana&species=iguana&search_param=%28%28common_name%3D%27Iguana+iguana%27%29%29
72	122	Common green iguana	Iguana iguana	Squamata	Lizard	El Salvador	America	FB	https://reptile-database.reptarium.cz/species?genus=Iguana&species=iguana&search_param=%28%28common_name%3D%27Iguana+iguana%27%29%29
72	122	Common green iguana	Iguana iguana	Squamata	Lizard	El Salvador	America	FB	https://reptile-database.reptarium.cz/species?genus=Iguana&species=iguana&search_param=%28%28common_name%3D%27Iguana+iguana%27%29%29
72	122	Common green iguana	Iguana iguana	Squamata	Lizard	El Salvador	America	FB	https://reptile-database.reptarium.cz/species?genus=Iguana&species=iguana&search_param=%28%28common_name%3D%27Iguana+iguana%27%29%29
73	123	Central bearded dragon	Pogona vitticeps	Squamata	Lizard	USA	America	CB	https://reptile-database.reptarium.cz/species?genus=Pogona&species=vitticeps&search_param=%28%28common_name%3D%27Pogona+vitticeps%27%29%29
73	123	Central bearded dragon	Pogona vitticeps	Squamata	Lizard	USA	America	CB	https://reptile-database.reptarium.cz/species?genus=Pogona&species=vitticeps&search_param=%28%28common_name%3D%27Pogona+vitticeps%27%29%29
73	123	Central bearded dragon	Pogona vitticeps	Squamata	Lizard	USA	America	CB	https://reptile-database.reptarium.cz/species?genus=Pogona&species=vitticeps&search_param=%28%28common_name%3D%27Pogona+vitticeps%27%29%29
73	123	Central bearded dragon	Pogona vitticeps	Squamata	Lizard	USA	America	CB	https://reptile-database.reptarium.cz/species?genus=Pogona&species=vitticeps&search_param=%28%28common_name%3D%27Pogona+vitticeps%27%29%29
73	123	Central bearded dragon	Pogona vitticeps	Squamata	Lizard	USA	America	CB	https://reptile-database.reptarium.cz/species?genus=Pogona&species=vitticeps&search_param=%28%28common_name%3D%27Pogona+vitticeps%27%29%29

Table S1: Animal species sampled in this study - country origin and categorization as captive bred (CB), farm bred (FB) and wild-caught (WC)

Shipment No.	Sample batch	Animal species	Scientific name of animal/s	Order	Suborder	Country origin	Continent	Captive bred (CB), farm bred (FB), wild-caught (WC)	Web URL used for assigning animal species to categories CB, FB and WC
73	123	Central bearded dragon	Pogona vitticeps	Squamata	Lizard	USA	America	CB	https://reptile-database.reptarium.cz/species?genus=Pogona&species=vitticeps&search_param=%28%28common_name%3D%27Pogona+vitticeps%27%29%29
73	123	Central bearded dragon	Pogona vitticeps	Squamata	Lizard	USA	America	CB	https://reptile-database.reptarium.cz/species?genus=Pogona&species=vitticeps&search_param=%28%28common_name%3D%27Pogona+vitticeps%27%29%29
73	124	Red cornsnake	Pantherophis guttatus	Squamata	Snake	USA	America	CB	https://reptile-database.reptarium.cz/species?genus=Pantherophis&species=guttatus&search_param=%28%28common_name%3D%27Pantherophis+guttatus%27%29%29
73	124	Red cornsnake	Pantherophis guttatus	Squamata	Snake	USA	America	CB	https://reptile-database.reptarium.cz/species?genus=Pantherophis&species=guttatus&search_param=%28%28common_name%3D%27Pantherophis+guttatus%27%29%29
73	124	Red cornsnake	Pantherophis guttatus	Squamata	Snake	USA	America	CB	https://reptile-database.reptarium.cz/species?genus=Pantherophis&species=guttatus&search_param=%28%28common_name%3D%27Pantherophis+guttatus%27%29%29
73	124	Red cornsnake	Pantherophis guttatus	Squamata	Snake	USA	America	CB	https://reptile-database.reptarium.cz/species?genus=Pantherophis&species=guttatus&search_param=%28%28common_name%3D%27Pantherophis+guttatus%27%29%29
73	124	Red cornsnake	Pantherophis guttatus	Squamata	Snake	USA	America	CB	https://reptile-database.reptarium.cz/species?genus=Pantherophis&species=guttatus&search_param=%28%28common_name%3D%27Pantherophis+guttatus%27%29%29
73	124	Red cornsnake	Pantherophis guttatus	Squamata	Snake	USA	America	CB	https://reptile-database.reptarium.cz/species?genus=Pantherophis&species=guttatus&search_param=%28%28common_name%3D%27Pantherophis+guttatus%27%29%29
73	124	Red cornsnake	Pantherophis guttatus	Squamata	Snake	USA	America	CB	https://reptile-database.reptarium.cz/species?genus=Pantherophis&species=guttatus&search_param=%28%28common_name%3D%27Pantherophis+guttatus%27%29%29
73	124	Red cornsnake	Pantherophis guttatus	Squamata	Snake	USA	America	CB	https://reptile-database.reptarium.cz/species?genus=Pantherophis&species=guttatus&search_param=%28%28common_name%3D%27Pantherophis+guttatus%27%29%29
73	125	Fat-tail gecko	Hemitheconyx caudicinctus	Squamata	Lizard	USA	America	CB	https://reptile-database.reptarium.cz/species?genus=Hemitheconyx&species=caudicinctus&search_param=%28%28common_name%3D%27Hemitheconyx+caudicinctus%27%29%29
73	125	Fat-tail gecko	Hemitheconyx caudicinctus	Squamata	Lizard	USA	America	CB	https://reptile-database.reptarium.cz/species?genus=Hemitheconyx&species=caudicinctus&search_param=%28%28common_name%3D%27Hemitheconyx+caudicinctus%27%29%29

Table S1: Animal species sampled in this study - country origin and categorization as captive bred (CB), farm bred (FB) and wild-caught (WC)

Shipment No.	Sample batch	Animal species	Scientific name of animal/s	Order	Suborder	Country origin	Continent	Captive bred (CB), farm bred (FB), wild-caught (WC)	Web URL used for assigning animal species to categories CB, FB and WC
73	125	Fat-tail gecko	Hemitheconyx caudicinctus	Squamata	Lizard	USA	America	CB	https://reptile-database.reptarium.cz/species?genus=Hemitheconyx&species=caudicinctus&search_param=%28%28common_name%3D%27Hemitheconyx+caudicinctus%27%29%29
73	125	Fat-tail gecko	Hemitheconyx caudicinctus	Squamata	Lizard	USA	America	CB	https://reptile-database.reptarium.cz/species?genus=Hemitheconyx&species=caudicinctus&search_param=%28%28common_name%3D%27Hemitheconyx+caudicinctus%27%29%29
73	125	Fat-tail gecko	Hemitheconyx caudicinctus	Squamata	Lizard	USA	America	CB	https://reptile-database.reptarium.cz/species?genus=Hemitheconyx&species=caudicinctus&search_param=%28%28common_name%3D%27Hemitheconyx+caudicinctus%27%29%29
73	125	Fat-tail gecko	Hemitheconyx caudicinctus	Squamata	Lizard	USA	America	CB	https://reptile-database.reptarium.cz/species?genus=Hemitheconyx&species=caudicinctus&search_param=%28%28common_name%3D%27Hemitheconyx+caudicinctus%27%29%29
73	125	Fat-tail gecko	Hemitheconyx caudicinctus	Squamata	Lizard	USA	America	CB	https://reptile-database.reptarium.cz/species?genus=Hemitheconyx&species=caudicinctus&search_param=%28%28common_name%3D%27Hemitheconyx+caudicinctus%27%29%29
73	126	New Caledonia giant gecko	Rhacodactylus leachianus	Squamata	Lizard	USA	America	CB	https://reptile-database.reptarium.cz/species?genus=Rhacodactylus&species=leachianus&search_param=%28%28common_name%3D%27Rhacodactylus+leachianus%27%29%29
73	126	New Caledonia giant gecko	Rhacodactylus leachianus	Squamata	Lizard	USA	America	CB	https://reptile-database.reptarium.cz/species?genus=Rhacodactylus&species=leachianus&search_param=%28%28common_name%3D%27Rhacodactylus+leachianus%27%29%29
73	126	New Caledonia giant gecko	Rhacodactylus leachianus	Squamata	Lizard	USA	America	CB	https://reptile-database.reptarium.cz/species?genus=Rhacodactylus&species=leachianus&search_param=%28%28common_name%3D%27Rhacodactylus+leachianus%27%29%29
73	126	New Caledonia giant gecko	Rhacodactylus leachianus	Squamata	Lizard	USA	America	CB	https://reptile-database.reptarium.cz/species?genus=Rhacodactylus&species=leachianus&search_param=%28%28common_name%3D%27Rhacodactylus+leachianus%27%29%29
73	126	New Caledonia giant gecko	Rhacodactylus leachianus	Squamata	Lizard	USA	America	CB	https://reptile-database.reptarium.cz/species?genus=Rhacodactylus&species=leachianus&search_param=%28%28common_name%3D%27Rhacodactylus+leachianus%27%29%29
73	126	New Caledonia giant gecko	Rhacodactylus leachianus	Squamata	Lizard	USA	America	CB	https://reptile-database.reptarium.cz/species?genus=Rhacodactylus&species=leachianus&search_param=%28%28common_name%3D%27Rhacodactylus+leachianus%27%29%29
73	127	Western hognose snake	Heterodon nasicus	Squamata	Snake	USA	America	CB	https://reptile-database.reptarium.cz/species?genus=Heterodon&species=nasicus&search_param=%28%28common_name%3D%27Heterodon+nasicus%27%29%29

Table S1: Animal species sampled in this study - country origin and categorization as captive bred (CB), farm bred (FB) and wild-caught (WC)

Shipment No.	Sample batch	Animal species	Scientific name of animal/s	Order	Suborder	Country origin	Continent	Captive bred (CB), farm bred (FB), wild-caught (WC)	Web URL used for assigning animal species to categories CB, FB and WC
73	127	Western hognose snake	Heterodon nasicus	Squamata	Snake	USA	America	CB	https://reptile-database.reptarium.cz/species?genus=Heterodon&species=nasicus&search_param=%28%28common_name%3D%27Heterodon+nasicus%27%29%29
73	127	Western hognose snake	Heterodon nasicus	Squamata	Snake	USA	America	CB	https://reptile-database.reptarium.cz/species?genus=Heterodon&species=nasicus&search_param=%28%28common_name%3D%27Heterodon+nasicus%27%29%29
74	128	Leopard tortoise	Stigmochelys pardalis	Testudines	Turtle	South-Africa	Africa	FB	https://reptile-database.reptarium.cz/species?genus=Stigmochelys&species=pardalis&search_param=%28%28common_name%3D%27Stigmochelys+pardalis%27%29%29
74	128	Leopard tortoise	Stigmochelys pardalis	Testudines	Turtle	South-Africa	Africa	FB	https://reptile-database.reptarium.cz/species?genus=Stigmochelys&species=pardalis&search_param=%28%28common_name%3D%27Stigmochelys+pardalis%27%29%29
74	128	Leopard tortoise	Stigmochelys pardalis	Testudines	Turtle	South-Africa	Africa	FB	https://reptile-database.reptarium.cz/species?genus=Stigmochelys&species=pardalis&search_param=%28%28common_name%3D%27Stigmochelys+pardalis%27%29%29
74	128	Leopard tortoise	Stigmochelys pardalis	Testudines	Turtle	South-Africa	Africa	FB	https://reptile-database.reptarium.cz/species?genus=Stigmochelys&species=pardalis&search_param=%28%28common_name%3D%27Stigmochelys+pardalis%27%29%29
74	128	Leopard tortoise	Stigmochelys pardalis	Testudines	Turtle	South-Africa	Africa	FB	https://reptile-database.reptarium.cz/species?genus=Stigmochelys&species=pardalis&search_param=%28%28common_name%3D%27Stigmochelys+pardalis%27%29%29
74	128	Leopard tortoise	Stigmochelys pardalis	Testudines	Turtle	South-Africa	Africa	FB	https://reptile-database.reptarium.cz/species?genus=Stigmochelys&species=pardalis&search_param=%28%28common_name%3D%27Stigmochelys+pardalis%27%29%29
74	128	Leopard tortoise	Stigmochelys pardalis	Testudines	Turtle	South-Africa	Africa	FB	https://reptile-database.reptarium.cz/species?genus=Stigmochelys&species=pardalis&search_param=%28%28common_name%3D%27Stigmochelys+pardalis%27%29%29
74	128	Leopard tortoise	Stigmochelys pardalis	Testudines	Turtle	South-Africa	Africa	FB	https://reptile-database.reptarium.cz/species?genus=Stigmochelys&species=pardalis&search_param=%28%28common_name%3D%27Stigmochelys+pardalis%27%29%29
74	128	Leopard tortoise	Stigmochelys pardalis	Testudines	Turtle	South-Africa	Africa	FB	https://reptile-database.reptarium.cz/species?genus=Stigmochelys&species=pardalis&search_param=%28%28common_name%3D%27Stigmochelys+pardalis%27%29%29
74	128	Leopard tortoise	Stigmochelys pardalis	Testudines	Turtle	South-Africa	Africa	FB	https://reptile-database.reptarium.cz/species?genus=Stigmochelys&species=pardalis&search_param=%28%28common_name%3D%27Stigmochelys+pardalis%27%29%29

Table S1: Animal species sampled in this study - country origin and categorization as captive bred (CB), farm bred (FB) and wild-caught (WC)

Shipment No.	Sample batch	Animal species	Scientific name of animal/s	Order	Suborder	Country origin	Continent	Captive bred (CB), farm bred (FB), wild-caught (WC)	Web URL used for assigning animal species to categories CB, FB and WC
75	129	Cuban giant anole	Anolis equestris	Squamata	Lizard	USA	America	WC/FB	https://reptile-database.reptarium.cz/species?genus=Anolis&species=equestris&search_param=%28%28common_name%3D%27Anolis+equestris%27%29%29
75	129	Cuban giant anole	Anolis equestris	Squamata	Lizard	USA	America	WC/FB	https://reptile-database.reptarium.cz/species?genus=Anolis&species=equestris&search_param=%28%28common_name%3D%27Anolis+equestris%27%29%29
75	129	Cuban giant anole	Anolis equestris	Squamata	Lizard	USA	America	WC/FB	https://reptile-database.reptarium.cz/species?genus=Anolis&species=equestris&search_param=%28%28common_name%3D%27Anolis+equestris%27%29%29
75	129	Cuban giant anole	Anolis equestris	Squamata	Lizard	USA	America	WC/FB	https://reptile-database.reptarium.cz/species?genus=Anolis&species=equestris&search_param=%28%28common_name%3D%27Anolis+equestris%27%29%29
75	129	Cuban giant anole	Anolis equestris	Squamata	Lizard	USA	America	WC/FB	https://reptile-database.reptarium.cz/species?genus=Anolis&species=equestris&search_param=%28%28common_name%3D%27Anolis+equestris%27%29%29
75	129	Cuban giant anole	Anolis equestris	Squamata	Lizard	USA	America	WC/FB	https://reptile-database.reptarium.cz/species?genus=Anolis&species=equestris&search_param=%28%28common_name%3D%27Anolis+equestris%27%29%29
75	129	Cuban giant anole	Anolis equestris	Squamata	Lizard	USA	America	WC/FB	https://reptile-database.reptarium.cz/species?genus=Anolis&species=equestris&search_param=%28%28common_name%3D%27Anolis+equestris%27%29%29
75	129	Cuban giant anole	Anolis equestris	Squamata	Lizard	USA	America	WC/FB	https://reptile-database.reptarium.cz/species?genus=Anolis&species=equestris&search_param=%28%28common_name%3D%27Anolis+equestris%27%29%29
75	130	Argentine black and white tegu	Salvator merianae	Squamata	Lizard	USA	America	CB	https://reptile-database.reptarium.cz/species?genus=Salvator&species=merianae&search_param=%28%28common_name%3D%27Salvator+merianae%27%29%29
75	130	Argentine black and white tegu	Salvator merianae	Squamata	Lizard	USA	America	CB	https://reptile-database.reptarium.cz/species?genus=Salvator&species=merianae&search_param=%28%28common_name%3D%27Salvator+merianae%27%29%29
75	130	Argentine black and white tegu	Salvator merianae	Squamata	Lizard	USA	America	CB	https://reptile-database.reptarium.cz/species?genus=Salvator&species=merianae&search_param=%28%28common_name%3D%27Salvator+merianae%27%29%29
75	130	Argentine black and white tegu	Salvator merianae	Squamata	Lizard	USA	America	CB	https://reptile-database.reptarium.cz/species?genus=Salvator&species=merianae&search_param=%28%28common_name%3D%27Salvator+merianae%27%29%29

Table S1: Animal species sampled in this study - country origin and categorization as captive bred (CB), farm bred (FB) and wild-caught (WC)

Shipment No.	Sample batch	Animal species	Scientific name of animal/s	Order	Suborder	Country origin	Continent	Captive bred (CB), farm bred (FB), wild-caught (WC)	Web URL used for assigning animal species to categories CB, FB and WC
75	130	Argentine black and white tegu	Salvator merianae	Squamata	Lizard	USA	America	CB	https://reptile-database.reptarium.cz/species?genus=Salvator&species=merianae&search_param=%28%28common_name%3D%27Salvator+merianae%27%29%29
75	130	Argentine black and white tegu	Salvator merianae	Squamata	Lizard	USA	America	CB	https://reptile-database.reptarium.cz/species?genus=Salvator&species=merianae&search_param=%28%28common_name%3D%27Salvator+merianae%27%29%29
75	130	Argentine black and white tegu	Salvator merianae	Squamata	Lizard	USA	America	CB	https://reptile-database.reptarium.cz/species?genus=Salvator&species=merianae&search_param=%28%28common_name%3D%27Salvator+merianae%27%29%29
75	130	Argentine black and white tegu	Salvator merianae	Squamata	Lizard	USA	America	CB	https://reptile-database.reptarium.cz/species?genus=Salvator&species=merianae&search_param=%28%28common_name%3D%27Salvator+merianae%27%29%29
75	130	Argentine black and white tegu	Salvator merianae	Squamata	Lizard	USA	America	CB	https://reptile-database.reptarium.cz/species?genus=Salvator&species=merianae&search_param=%28%28common_name%3D%27Salvator+merianae%27%29%29
75	130	Argentine black and white tegu	Salvator merianae	Squamata	Lizard	USA	America	CB	https://reptile-database.reptarium.cz/species?genus=Salvator&species=merianae&search_param=%28%28common_name%3D%27Salvator+merianae%27%29%29
75	131	Green spiny lizard	Sceloporus malachiticus	Squamata	Lizard	USA	America	presumably CB	https://reptile-database.reptarium.cz/species?genus=Sceloporus&species=malachiticus&search_param=%28%28common_name%3D%27Sceloporus+malachiticus%27%29%29
75	131	Green spiny lizard	Sceloporus malachiticus	Squamata	Lizard	USA	America	presumably CB	https://reptile-database.reptarium.cz/species?genus=Sceloporus&species=malachiticus&search_param=%28%28common_name%3D%27Sceloporus+malachiticus%27%29%29
75	131	Green spiny lizard	Sceloporus malachiticus	Squamata	Lizard	USA	America	presumably CB	https://reptile-database.reptarium.cz/species?genus=Sceloporus&species=malachiticus&search_param=%28%28common_name%3D%27Sceloporus+malachiticus%27%29%29
75	131	Green spiny lizard	Sceloporus malachiticus	Squamata	Lizard	USA	America	presumably CB	https://reptile-database.reptarium.cz/species?genus=Sceloporus&species=malachiticus&search_param=%28%28common_name%3D%27Sceloporus+malachiticus%27%29%29
75	131	Green spiny lizard	Sceloporus malachiticus	Squamata	Lizard	USA	America	presumably CB	https://reptile-database.reptarium.cz/species?genus=Sceloporus&species=malachiticus&search_param=%28%28common_name%3D%27Sceloporus+malachiticus%27%29%29
75	132	Lined gecko	Gekko vittatus	Squamata	Lizard	USA	America	WC	https://reptile-database.reptarium.cz/species?genus=Gekko&species=vittatus&search_param=%28%28common_name%3D%27Gekko+gecko%27%29%29

Table S1: Animal species sampled in this study - country origin and categorization as captive bred (CB), farm bred (FB) and wild-caught (WC)

Shipment No.	Sample batch	Animal species	Scientific name of animal/s	Order	Suborder	Country origin	Continent	Captive bred (CB), farm bred (FB), wild-caught (WC)	Web URL used for assigning animal species to categories CB, FB and WC
75	132	Lined gecko	Gekko vittatus	Squamata	Lizard	USA	America	WC	https://reptile-database.reptarium.cz/species?genus=Gekko&species=vittatus&search_param=%28%28common_name%3D%27Gekko+gecko%27%29%29
75	132	Lined gecko	Gekko vittatus	Squamata	Lizard	USA	America	WC	https://reptile-database.reptarium.cz/species?genus=Gekko&species=vittatus&search_param=%28%28common_name%3D%27Gekko+gecko%27%29%29
75	132	Lined gecko	Gekko vittatus	Squamata	Lizard	USA	America	WC	https://reptile-database.reptarium.cz/species?genus=Gekko&species=vittatus&search_param=%28%28common_name%3D%27Gekko+gecko%27%29%29
75	133	Striped mud turtle	Kinosternon baurii	Testudines	Turtle	USA	America	FB	https://reptile-database.reptarium.cz/species?genus=Kinosternon&species=baurii&search_param=%28%28common_name%3D%27Kinosternon+baurii%27%29%29
75	133	Striped mud turtle	Kinosternon baurii	Testudines	Turtle	USA	America	FB	https://reptile-database.reptarium.cz/species?genus=Kinosternon&species=baurii&search_param=%28%28common_name%3D%27Kinosternon+baurii%27%29%29
75	133	Striped mud turtle	Kinosternon baurii	Testudines	Turtle	USA	America	FB	https://reptile-database.reptarium.cz/species?genus=Kinosternon&species=baurii&search_param=%28%28common_name%3D%27Kinosternon+baurii%27%29%29
75	133	Striped mud turtle	Kinosternon baurii	Testudines	Turtle	USA	America	FB	https://reptile-database.reptarium.cz/species?genus=Kinosternon&species=baurii&search_param=%28%28common_name%3D%27Kinosternon+baurii%27%29%29
75	133	Striped mud turtle	Kinosternon baurii	Testudines	Turtle	USA	America	FB	https://reptile-database.reptarium.cz/species?genus=Kinosternon&species=baurii&search_param=%28%28common_name%3D%27Kinosternon+baurii%27%29%29
75	133	Striped mud turtle	Kinosternon baurii	Testudines	Turtle	USA	America	FB	https://reptile-database.reptarium.cz/species?genus=Kinosternon&species=baurii&search_param=%28%28common_name%3D%27Kinosternon+baurii%27%29%29
75	134	Eastern ribbonsnake	Thamnophis sauritus	Squamata	Snake	USA	America	WC	https://reptile-database.reptarium.cz/species?genus=Thamnophis&species=saurita&search_param=%28%28common_name%3D%27Thamnophis+sauritus%27%29%29
75	134	Eastern ribbonsnake	Thamnophis sauritus	Squamata	Snake	USA	America	WC	https://reptile-database.reptarium.cz/species?genus=Thamnophis&species=saurita&search_param=%28%28common_name%3D%27Thamnophis+sauritus%27%29%29
75	134	Eastern ribbonsnake	Thamnophis sauritus	Squamata	Snake	USA	America	WC	https://reptile-database.reptarium.cz/species?genus=Thamnophis&species=saurita&search_param=%28%28common_name%3D%27Thamnophis+sauritus%27%29%29

Table S1: Animal species sampled in this study - country origin and categorization as captive bred (CB), farm bred (FB) and wild-caught (WC)

Shipment No.	Sample batch	Animal species	Scientific name of animal/s	Order	Suborder	Country origin	Continent	Captive bred (CB), farm bred (FB), wild-caught (WC)	Web URL used for assigning animal species to categories CB, FB and WC
75	134	Eastern ribbonsnake	Thamnophis sauritus	Squamata	Snake	USA	America	WC	https://reptile-database.reptarium.cz/species?genus=Thamnophis&species=saurita&search_param=%28%28common_name%3D%27Thamnophis+sauritus%27%29%29
75	134	Eastern ribbonsnake	Thamnophis sauritus	Squamata	Snake	USA	America	WC	https://reptile-database.reptarium.cz/species?genus=Thamnophis&species=saurita&search_param=%28%28common_name%3D%27Thamnophis+sauritus%27%29%29
75	135	Savannah monitor	Varanus exanthematicus	Squamata	Lizard	USA	America	CB	https://reptile-database.reptarium.cz/species?genus=Varanus&species=exanthematicus&search_param=%28%28common_name%3D%27Varanus+exanthematicus%27%29%29
75	135	Savannah monitor	Varanus exanthematicus	Squamata	Lizard	USA	America	CB	https://reptile-database.reptarium.cz/species?genus=Varanus&species=exanthematicus&search_param=%28%28common_name%3D%27Varanus+exanthematicus%27%29%29
75	135	Savannah monitor	Varanus exanthematicus	Squamata	Lizard	USA	America	CB	https://reptile-database.reptarium.cz/species?genus=Varanus&species=exanthematicus&search_param=%28%28common_name%3D%27Varanus+exanthematicus%27%29%29
75	135	Savannah monitor	Varanus exanthematicus	Squamata	Lizard	USA	America	CB	https://reptile-database.reptarium.cz/species?genus=Varanus&species=exanthematicus&search_param=%28%28common_name%3D%27Varanus+exanthematicus%27%29%29
75	135	Savannah monitor	Varanus exanthematicus	Squamata	Lizard	USA	America	CB	https://reptile-database.reptarium.cz/species?genus=Varanus&species=exanthematicus&search_param=%28%28common_name%3D%27Varanus+exanthematicus%27%29%29
75	135	Savannah monitor	Varanus exanthematicus	Squamata	Lizard	USA	America	CB	https://reptile-database.reptarium.cz/species?genus=Varanus&species=exanthematicus&search_param=%28%28common_name%3D%27Varanus+exanthematicus%27%29%29
75	135	Savannah monitor	Varanus exanthematicus	Squamata	Lizard	USA	America	CB	https://reptile-database.reptarium.cz/species?genus=Varanus&species=exanthematicus&search_param=%28%28common_name%3D%27Varanus+exanthematicus%27%29%29
76	136	Shingleback lizard	Tiliqua rugosa	Squamata	Lizard	Japan	Asia	CB	https://reptile-database.reptarium.cz/species?genus=Tiliqua&species=rugosa&search_param=%28%28common_name%3D%27Tiliqua+rugosa%27%29%29
76	136	Shingleback lizard	Tiliqua rugosa	Squamata	Lizard	Japan	Asia	CB	https://reptile-database.reptarium.cz/species?genus=Tiliqua&species=rugosa&search_param=%28%28common_name%3D%27Tiliqua+rugosa%27%29%29
76	136	Shingleback lizard	Tiliqua rugosa	Squamata	Lizard	Japan	Asia	CB	https://reptile-database.reptarium.cz/species?genus=Tiliqua&species=rugosa&search_param=%28%28common_name%3D%27Tiliqua+rugosa%27%29%29

Table S1: Animal species sampled in this study - country origin and categorization as captive bred (CB), farm bred (FB) and wild-caught (WC)

Shipment No.	Sample batch	Animal species	Scientific name of animal/s	Order	Suborder	Country origin	Continent	Captive bred (CB), farm bred (FB), wild-caught (WC)	Web URL used for assigning animal species to categories CB, FB and WC
76	136	Shingleback lizard	Tiliqua rugosa	Squamata	Lizard	Japan	Asia	CB	https://reptile-database.reptarium.cz/species?genus=Tiliqua&species=rugosa&search_param=%28%28common_name%3D%27Tiliqua+rugosa%27%29%29
76	136	Shingleback lizard	Tiliqua rugosa	Squamata	Lizard	Japan	Asia	CB	https://reptile-database.reptarium.cz/species?genus=Tiliqua&species=rugosa&search_param=%28%28common_name%3D%27Tiliqua+rugosa%27%29%29
76	136	Shingleback lizard	Tiliqua rugosa	Squamata	Lizard	Japan	Asia	CB	https://reptile-database.reptarium.cz/species?genus=Tiliqua&species=rugosa&search_param=%28%28common_name%3D%27Tiliqua+rugosa%27%29%29
76	136	Shingleback lizard	Tiliqua rugosa	Squamata	Lizard	Japan	Asia	CB	https://reptile-database.reptarium.cz/species?genus=Tiliqua&species=rugosa&search_param=%28%28common_name%3D%27Tiliqua+rugosa%27%29%29
76	136	Shingleback lizard	Tiliqua rugosa	Squamata	Lizard	Japan	Asia	CB	https://reptile-database.reptarium.cz/species?genus=Tiliqua&species=rugosa&search_param=%28%28common_name%3D%27Tiliqua+rugosa%27%29%29
77	137	Ball python	Python regius	Squamata	Snake	USA	America	CB	https://reptile-database.reptarium.cz/species?genus=Python&species=regius&search_param=%28%28common_name%3D%27Python+regius%27%29%29
77	137	Ball python	Python regius	Squamata	Snake	USA	America	CB	https://reptile-database.reptarium.cz/species?genus=Python&species=regius&search_param=%28%28common_name%3D%27Python+regius%27%29%29
77	137	Ball python	Python regius	Squamata	Snake	USA	America	CB	https://reptile-database.reptarium.cz/species?genus=Python&species=regius&search_param=%28%28common_name%3D%27Python+regius%27%29%29
77	137	Ball python	Python regius	Squamata	Snake	USA	America	CB	https://reptile-database.reptarium.cz/species?genus=Python&species=regius&search_param=%28%28common_name%3D%27Python+regius%27%29%29
77	137	Ball python	Python regius	Squamata	Snake	USA	America	CB	https://reptile-database.reptarium.cz/species?genus=Python&species=regius&search_param=%28%28common_name%3D%27Python+regius%27%29%29
78	138	Chinese water dragon	Physignatus cocincinus	Squamata	Lizard	Vietnam	Asia	WC	https://reptile-database.reptarium.cz/species?genus=Physignathus&species=cocincinus&search_param=%28%28common_name%3D%27gr%C3%BCne+wasseragame%27%29%29
78	138	Chinese water dragon	Physignatus cocincinus	Squamata	Lizard	Vietnam	Asia	WC	https://reptile-database.reptarium.cz/species?genus=Physignathus&species=cocincinus&search_param=%28%28common_name%3D%27gr%C3%BCne+wasseragame%27%29%29

Table S1: Animal species sampled in this study - country origin and categorization as captive bred (CB), farm bred (FB) and wild-caught (WC)

Shipment No.	Sample batch	Animal species	Scientific name of animal/s	Order	Suborder	Country origin	Continent	Captive bred (CB), farm bred (FB), wild-caught (WC)	Web URL used for assigning animal species to categories CB, FB and WC
78	138	Chinese water dragon	Physignatus cocincinus	Squamata	Lizard	Vietnam	Asia	WC	https://reptile-database.reptarium.cz/species?genus=Physignathus&species=cocincinus&search_param=%28%28common_name%3D%27gr%C3%BCne+wasseragame%27%29%29
78	138	Chinese water dragon	Physignatus cocincinus	Squamata	Lizard	Vietnam	Asia	WC	https://reptile-database.reptarium.cz/species?genus=Physignathus&species=cocincinus&search_param=%28%28common_name%3D%27gr%C3%BCne+wasseragame%27%29%29
78	138	Chinese water dragon	Physignatus cocincinus	Squamata	Lizard	Vietnam	Asia	WC	https://reptile-database.reptarium.cz/species?genus=Physignathus&species=cocincinus&search_param=%28%28common_name%3D%27gr%C3%BCne+wasseragame%27%29%29
78	138	Chinese water dragon	Physignatus cocincinus	Squamata	Lizard	Vietnam	Asia	WC	https://reptile-database.reptarium.cz/species?genus=Physignathus&species=cocincinus&search_param=%28%28common_name%3D%27gr%C3%BCne+wasseragame%27%29%29
78	138	Chinese water dragon	Physignatus cocincinus	Squamata	Lizard	Vietnam	Asia	WC	https://reptile-database.reptarium.cz/species?genus=Physignathus&species=cocincinus&search_param=%28%28common_name%3D%27gr%C3%BCne+wasseragame%27%29%29
78	138	Chinese water dragon	Physignatus cocincinus	Squamata	Lizard	Vietnam	Asia	WC	https://reptile-database.reptarium.cz/species?genus=Physignathus&species=cocincinus&search_param=%28%28common_name%3D%27gr%C3%BCne+wasseragame%27%29%29
78	138	Chinese water dragon	Physignatus cocincinus	Squamata	Lizard	Vietnam	Asia	WC	https://reptile-database.reptarium.cz/species?genus=Physignathus&species=cocincinus&search_param=%28%28common_name%3D%27gr%C3%BCne+wasseragame%27%29%29
78	138	Chinese water dragon	Physignatus cocincinus	Squamata	Lizard	Vietnam	Asia	WC	https://reptile-database.reptarium.cz/species?genus=Physignathus&species=cocincinus&search_param=%28%28common_name%3D%27gr%C3%BCne+wasseragame%27%29%29
78	138	Chinese water dragon	Physignatus cocincinus	Squamata	Lizard	Vietnam	Asia	WC	https://reptile-database.reptarium.cz/species?genus=Physignathus&species=cocincinus&search_param=%28%28common_name%3D%27gr%C3%BCne+wasseragame%27%29%29
78	138	Chinese water dragon	Physignatus cocincinus	Squamata	Lizard	Vietnam	Asia	WC	https://reptile-database.reptarium.cz/species?genus=Physignathus&species=cocincinus&search_param=%28%28common_name%3D%27gr%C3%BCne+wasseragame%27%29%29
78	139	Asian grass lizard	Takydromus sexlineatus	Squamata	Lizard	Vietnam	Asia	WC	https://reptile-database.reptarium.cz/species?genus=Takydromus&species=sexlineatus&search_param=%28%28common_name%3D%27Takydromus+sexlineatus%27%29%29
78	139	Asian grass lizard	Takydromus sexlineatus	Squamata	Lizard	Vietnam	Asia	WC	https://reptile-database.reptarium.cz/species?genus=Takydromus&species=sexlineatus&search_param=%28%28common_name%3D%27Takydromus+sexlineatus%27%29%29

Table S1: Animal species sampled in this study - country origin and categorization as captive bred (CB), farm bred (FB) and wild-caught (WC)

Shipment No.	Sample batch	Animal species	Scientific name of animal/s	Order	Suborder	Country origin	Continent	Captive bred (CB), farm bred (FB), wild-caught (WC)	Web URL used for assigning animal species to categories CB, FB and WC
78	139	Asian grass lizard	Takydromus sexlineatus	Squamata	Lizard	Vietnam	Asia	WC	https://reptile-database.reptarium.cz/species?genus=Takydromus&species=sexlineatus&search_param=%28%28common_name%3D%27Takydromus+sexlineatus%27%29%29
78	139	Asian grass lizard	Takydromus sexlineatus	Squamata	Lizard	Vietnam	Asia	WC	https://reptile-database.reptarium.cz/species?genus=Takydromus&species=sexlineatus&search_param=%28%28common_name%3D%27Takydromus+sexlineatus%27%29%29
78	139	Asian grass lizard	Takydromus sexlineatus	Squamata	Lizard	Vietnam	Asia	WC	https://reptile-database.reptarium.cz/species?genus=Takydromus&species=sexlineatus&search_param=%28%28common_name%3D%27Takydromus+sexlineatus%27%29%29
78	139	Asian grass lizard	Takydromus sexlineatus	Squamata	Lizard	Vietnam	Asia	WC	https://reptile-database.reptarium.cz/species?genus=Takydromus&species=sexlineatus&search_param=%28%28common_name%3D%27Takydromus+sexlineatus%27%29%29
78	139	Asian grass lizard	Takydromus sexlineatus	Squamata	Lizard	Vietnam	Asia	WC	https://reptile-database.reptarium.cz/species?genus=Takydromus&species=sexlineatus&search_param=%28%28common_name%3D%27Takydromus+sexlineatus%27%29%29
78	140	Pricklenape	Acanthosaura sp.	Squamata	Lizard	Vietnam	Asia	WC	https://reptile-database.reptarium.cz/advanced_search?common_name=Acanthosaura&submit=Search
78	140	Pricklenape	Acanthosaura sp.	Squamata	Lizard	Vietnam	Asia	WC	https://reptile-database.reptarium.cz/advanced_search?common_name=Acanthosaura&submit=Search
78	140	Pricklenape	Acanthosaura sp.	Squamata	Lizard	Vietnam	Asia	WC	https://reptile-database.reptarium.cz/advanced_search?common_name=Acanthosaura&submit=Search
78	140	Pricklenape	Acanthosaura sp.	Squamata	Lizard	Vietnam	Asia	WC	https://reptile-database.reptarium.cz/advanced_search?common_name=Acanthosaura&submit=Search
79	141	Green basilisk	Basiliscus plumifrons	Squamata	Lizard	Nicaragua	America	WC	https://reptile-database.reptarium.cz/species?genus=Basiliscus&species=plumifrons&search_param=%28%28common_name%3D%27Basiliscus+plumifrons%27%29%29
79	141	Green basilisk	Basiliscus plumifrons	Squamata	Lizard	Nicaragua	America	WC	https://reptile-database.reptarium.cz/species?genus=Basiliscus&species=plumifrons&search_param=%28%28common_name%3D%27Basiliscus+plumifrons%27%29%29
79	141	Green basilisk	Basiliscus plumifrons	Squamata	Lizard	Nicaragua	America	WC	https://reptile-database.reptarium.cz/species?genus=Basiliscus&species=plumifrons&search_param=%28%28common_name%3D%27Basiliscus+plumifrons%27%29%29
79	141	Green basilisk	Basiliscus plumifrons	Squamata	Lizard	Nicaragua	America	WC	https://reptile-database.reptarium.cz/species?genus=Basiliscus&species=plumifrons&search_param=%28%28common_name%3D%27Basiliscus+plumifrons%27%29%29

Table S1: Animal species sampled in this study - country origin and categorization as captive bred (CB), farm bred (FB) and wild-caught (WC)

Shipment No.	Sample batch	Animal species	Scientific name of animal/s	Order	Suborder	Country origin	Continent	Captive bred (CB), farm bred (FB), wild-caught (WC)	Web URL used for assigning animal species to categories CB, FB and WC
79	141	Green basilisk	Basiliscus plumifrons	Squamata	Lizard	Nicaragua	America	WC	https://reptile-database.reptarium.cz/species?genus=Basiliscus&species=plumifrons&search_param=%28%28common_name%3D%27Basiliscus+plumifrons%27%29%29
79	142	Painted wood turtle	Rhinoclemmys pulcherrima	Testudines	Turtle	Nicaragua	America	WC	https://reptile-database.reptarium.cz/species?genus=Rhinoclemmys&species=pulcherrima&search_param=%28%28common_name%3D%27Rhinoclemmys+pulcherrima%27%29%29
79	142	Painted wood turtle	Rhinoclemmys pulcherrima	Testudines	Turtle	Nicaragua	America	WC	https://reptile-database.reptarium.cz/species?genus=Rhinoclemmys&species=pulcherrima&search_param=%28%28common_name%3D%27Rhinoclemmys+pulcherrima%27%29%29
79	142	Painted wood turtle	Rhinoclemmys pulcherrima	Testudines	Turtle	Nicaragua	America	WC	https://reptile-database.reptarium.cz/species?genus=Rhinoclemmys&species=pulcherrima&search_param=%28%28common_name%3D%27Rhinoclemmys+pulcherrima%27%29%29
79	142	Painted wood turtle	Rhinoclemmys pulcherrima	Testudines	Turtle	Nicaragua	America	WC	https://reptile-database.reptarium.cz/species?genus=Rhinoclemmys&species=pulcherrima&search_param=%28%28common_name%3D%27Rhinoclemmys+pulcherrima%27%29%29
79	142	Painted wood turtle	Rhinoclemmys pulcherrima	Testudines	Turtle	Nicaragua	America	WC	https://reptile-database.reptarium.cz/species?genus=Rhinoclemmys&species=pulcherrima&search_param=%28%28common_name%3D%27Rhinoclemmys+pulcherrima%27%29%29
79	142	Painted wood turtle	Rhinoclemmys pulcherrima	Testudines	Turtle	Nicaragua	America	WC	https://reptile-database.reptarium.cz/species?genus=Rhinoclemmys&species=pulcherrima&search_param=%28%28common_name%3D%27Rhinoclemmys+pulcherrima%27%29%29
79	142	Painted wood turtle	Rhinoclemmys pulcherrima	Testudines	Turtle	Nicaragua	America	WC	https://reptile-database.reptarium.cz/species?genus=Rhinoclemmys&species=pulcherrima&search_param=%28%28common_name%3D%27Rhinoclemmys+pulcherrima%27%29%29
79	142	Painted wood turtle	Rhinoclemmys pulcherrima	Testudines	Turtle	Nicaragua	America	WC	https://reptile-database.reptarium.cz/species?genus=Rhinoclemmys&species=pulcherrima&search_param=%28%28common_name%3D%27Rhinoclemmys+pulcherrima%27%29%29
79	142	Painted wood turtle	Rhinoclemmys pulcherrima	Testudines	Turtle	Nicaragua	America	WC	https://reptile-database.reptarium.cz/species?genus=Rhinoclemmys&species=pulcherrima&search_param=%28%28common_name%3D%27Rhinoclemmys+pulcherrima%27%29%29
79	142	Painted wood turtle	Rhinoclemmys pulcherrima	Testudines	Turtle	Nicaragua	America	WC	https://reptile-database.reptarium.cz/species?genus=Rhinoclemmys&species=pulcherrima&search_param=%28%28common_name%3D%27Rhinoclemmys+pulcherrima%27%29%29
80	143	Painted wood turtle	Rhinoclemmys pulcherrima	Testudines	Turtle	USA	America	presumably CB	https://reptile-database.reptarium.cz/species?genus=Rhinoclemmys&species=pulcherrima&search_param=%28%28common_name%3D%27Rhinoclemmys+pulcherrima%27%29%29

Table S1: Animal species sampled in this study - country origin and categorization as captive bred (CB), farm bred (FB) and wild-caught (WC)

Shipment No.	Sample batch	Animal species	Scientific name of animal/s	Order	Suborder	Country origin	Continent	Captive bred (CB), farm bred (FB), wild-caught (WC)	Web URL used for assigning animal species to categories CB, FB and WC
80	143	Painted wood turtle	Rhinoclemmys pulcherrima	Testudines	Turtle	USA	America	presumably CB	https://reptile-database.reptarium.cz/species?genus=Rhinoclemmys&species=pulcherrima&search_param=%28%28common_name%3D%27Rhinoclemmys+pulcherrima%27%29%29
80	143	Painted wood turtle	Rhinoclemmys pulcherrima	Testudines	Turtle	USA	America	presumably CB	https://reptile-database.reptarium.cz/species?genus=Rhinoclemmys&species=pulcherrima&search_param=%28%28common_name%3D%27Rhinoclemmys+pulcherrima%27%29%29
80	143	Painted wood turtle	Rhinoclemmys pulcherrima	Testudines	Turtle	USA	America	presumably CB	https://reptile-database.reptarium.cz/species?genus=Rhinoclemmys&species=pulcherrima&search_param=%28%28common_name%3D%27Rhinoclemmys+pulcherrima%27%29%29
80	143	Painted wood turtle	Rhinoclemmys pulcherrima	Testudines	Turtle	USA	America	presumably CB	https://reptile-database.reptarium.cz/species?genus=Rhinoclemmys&species=pulcherrima&search_param=%28%28common_name%3D%27Rhinoclemmys+pulcherrima%27%29%29
80	143	Painted wood turtle	Rhinoclemmys pulcherrima	Testudines	Turtle	USA	America	presumably CB	https://reptile-database.reptarium.cz/species?genus=Rhinoclemmys&species=pulcherrima&search_param=%28%28common_name%3D%27Rhinoclemmys+pulcherrima%27%29%29
80	143	Painted wood turtle	Rhinoclemmys pulcherrima	Testudines	Turtle	USA	America	presumably CB	https://reptile-database.reptarium.cz/species?genus=Rhinoclemmys&species=pulcherrima&search_param=%28%28common_name%3D%27Rhinoclemmys+pulcherrima%27%29%29
80	143	Painted wood turtle	Rhinoclemmys pulcherrima	Testudines	Turtle	USA	America	presumably CB	https://reptile-database.reptarium.cz/species?genus=Rhinoclemmys&species=pulcherrima&search_param=%28%28common_name%3D%27Rhinoclemmys+pulcherrima%27%29%29
80	143	Painted wood turtle	Rhinoclemmys pulcherrima	Testudines	Turtle	USA	America	presumably CB	https://reptile-database.reptarium.cz/species?genus=Rhinoclemmys&species=pulcherrima&search_param=%28%28common_name%3D%27Rhinoclemmys+pulcherrima%27%29%29
80	143	Painted wood turtle	Rhinoclemmys pulcherrima	Testudines	Turtle	USA	America	presumably CB	https://reptile-database.reptarium.cz/species?genus=Rhinoclemmys&species=pulcherrima&search_param=%28%28common_name%3D%27Rhinoclemmys+pulcherrima%27%29%29
80	143	Painted wood turtle	Rhinoclemmys pulcherrima	Testudines	Turtle	USA	America	presumably CB	https://reptile-database.reptarium.cz/species?genus=Rhinoclemmys&species=pulcherrima&search_param=%28%28common_name%3D%27Rhinoclemmys+pulcherrima%27%29%29
80	143	Painted wood turtle	Rhinoclemmys pulcherrima	Testudines	Turtle	USA	America	presumably CB	https://reptile-database.reptarium.cz/species?genus=Rhinoclemmys&species=pulcherrima&search_param=%28%28common_name%3D%27Rhinoclemmys+pulcherrima%27%29%29
80	143	Painted wood turtle	Rhinoclemmys pulcherrima	Testudines	Turtle	USA	America	presumably CB	https://reptile-database.reptarium.cz/species?genus=Rhinoclemmys&species=pulcherrima&search_param=%28%28common_name%3D%27Rhinoclemmys+pulcherrima%27%29%29

Table S1: Animal species sampled in this study - country origin and categorization as captive bred (CB), farm bred (FB) and wild-caught (WC)

Shipment No.	Sample batch	Animal species	Scientific name of animal/s	Order	Suborder	Country origin	Continent	Captive bred (CB), farm bred (FB), wild-caught (WC)	Web URL used for assigning animal species to categories CB, FB and WC
80	143	Painted wood turtle	Rhinoclemmys pulcherrima	Testudines	Turtle	USA	America	presumably CB	https://reptile-database.reptarium.cz/species?genus=Rhinoclemmys&species=pulcherrima&search_param=%28%28common_name%3D%27Rhinoclemmys+pulcherrima%27%29%29
81	144	Horsefield's tortoise	Testudo horsfieldii	Testudines	Turtle	Uzbekistan	Asia	FB	https://reptile-database.reptarium.cz/species?genus=Testudo&species=horsfieldii&search_param=%28%28common_name%3D%27Testudo+horsfieldii%27%29%29
81	144	Horsefield's tortoise	Testudo horsfieldii	Testudines	Turtle	Uzbekistan	Asia	FB	https://reptile-database.reptarium.cz/species?genus=Testudo&species=horsfieldii&search_param=%28%28common_name%3D%27Testudo+horsfieldii%27%29%29
81	144	Horsefield's tortoise	Testudo horsfieldii	Testudines	Turtle	Uzbekistan	Asia	FB	https://reptile-database.reptarium.cz/species?genus=Testudo&species=horsfieldii&search_param=%28%28common_name%3D%27Testudo+horsfieldii%27%29%29
81	144	Horsefield's tortoise	Testudo horsfieldii	Testudines	Turtle	Uzbekistan	Asia	FB	https://reptile-database.reptarium.cz/species?genus=Testudo&species=horsfieldii&search_param=%28%28common_name%3D%27Testudo+horsfieldii%27%29%29
81	144	Horsefield's tortoise	Testudo horsfieldii	Testudines	Turtle	Uzbekistan	Asia	FB	https://reptile-database.reptarium.cz/species?genus=Testudo&species=horsfieldii&search_param=%28%28common_name%3D%27Testudo+horsfieldii%27%29%29
82	145	Sand monitor	Varanus gouldii	Squamata	Lizard	Canada	America	CB	https://reptile-database.reptarium.cz/species?genus=Varanus&species=gouldii&search_param=%28%28common_name%3D%27Varanus+gouldii%27%29%29
82	145	Sand monitor	Varanus gouldii	Squamata	Lizard	Canada	America	CB	https://reptile-database.reptarium.cz/species?genus=Varanus&species=gouldii&search_param=%28%28common_name%3D%27Varanus+gouldii%27%29%29
82	145	Sand monitor	Varanus gouldii	Squamata	Lizard	Canada	America	CB	https://reptile-database.reptarium.cz/species?genus=Varanus&species=gouldii&search_param=%28%28common_name%3D%27Varanus+gouldii%27%29%29
82	145	Sand monitor	Varanus gouldii	Squamata	Lizard	Canada	America	CB	https://reptile-database.reptarium.cz/species?genus=Varanus&species=gouldii&search_param=%28%28common_name%3D%27Varanus+gouldii%27%29%29
82	145	Sand monitor	Varanus gouldii	Squamata	Lizard	Canada	America	CB	https://reptile-database.reptarium.cz/species?genus=Varanus&species=gouldii&search_param=%28%28common_name%3D%27Varanus+gouldii%27%29%29
82	145	Sand monitor	Varanus gouldii	Squamata	Lizard	Canada	America	CB	https://reptile-database.reptarium.cz/species?genus=Varanus&species=gouldii&search_param=%28%28common_name%3D%27Varanus+gouldii%27%29%29

Table S1: Animal species sampled in this study - country origin and categorization as captive bred (CB), farm bred (FB) and wild-caught (WC)

Shipment No.	Sample batch	Animal species	Scientific name of animal/s	Order	Suborder	Country origin	Continent	Captive bred (CB), farm bred (FB), wild-caught (WC)	Web URL used for assigning animal species to categories CB, FB and WC
82	145	Sand monitor	Varanus gouldii	Squamata	Lizard	Canada	America	CB	https://reptile-database.reptarium.cz/species?genus=Varanus&species=gouldii&search_param=%28%28common_name%3D%27Varanus+gouldii%27%29%29
82	145	Sand monitor	Varanus gouldii	Squamata	Lizard	Canada	America	CB	https://reptile-database.reptarium.cz/species?genus=Varanus&species=gouldii&search_param=%28%28common_name%3D%27Varanus+gouldii%27%29%29
82	145	Sand monitor	Varanus gouldii	Squamata	Lizard	Canada	America	CB	https://reptile-database.reptarium.cz/species?genus=Varanus&species=gouldii&search_param=%28%28common_name%3D%27Varanus+gouldii%27%29%29
82	145	Sand monitor	Varanus gouldii	Squamata	Lizard	Canada	America	CB	https://reptile-database.reptarium.cz/species?genus=Varanus&species=gouldii&search_param=%28%28common_name%3D%27Varanus+gouldii%27%29%29
83	146	Common leopard gecko	Eublepharis macularius	Squamata	Lizard	Canada	America	CB	https://reptile-database.reptarium.cz/species?genus=Eublepharis&species=macularius&search_param=%28%28common_name%3D%27Eublepharis+macularius%27%29%29
83	146	Common leopard gecko	Eublepharis macularius	Squamata	Lizard	Canada	America	CB	https://reptile-database.reptarium.cz/species?genus=Eublepharis&species=macularius&search_param=%28%28common_name%3D%27Eublepharis+macularius%27%29%29
83	146	Common leopard gecko	Eublepharis macularius	Squamata	Lizard	Canada	America	CB	https://reptile-database.reptarium.cz/species?genus=Eublepharis&species=macularius&search_param=%28%28common_name%3D%27Eublepharis+macularius%27%29%29
83	146	Common leopard gecko	Eublepharis macularius	Squamata	Lizard	Canada	America	CB	https://reptile-database.reptarium.cz/species?genus=Eublepharis&species=macularius&search_param=%28%28common_name%3D%27Eublepharis+macularius%27%29%29
83	146	Common leopard gecko	Eublepharis macularius	Squamata	Lizard	Canada	America	CB	https://reptile-database.reptarium.cz/species?genus=Eublepharis&species=macularius&search_param=%28%28common_name%3D%27Eublepharis+macularius%27%29%29
83	146	Common leopard gecko	Eublepharis macularius	Squamata	Lizard	Canada	America	CB	https://reptile-database.reptarium.cz/species?genus=Eublepharis&species=macularius&search_param=%28%28common_name%3D%27Eublepharis+macularius%27%29%29
83	146	Common leopard gecko	Eublepharis macularius	Squamata	Lizard	Canada	America	CB	https://reptile-database.reptarium.cz/species?genus=Eublepharis&species=macularius&search_param=%28%28common_name%3D%27Eublepharis+macularius%27%29%29
83	147	Crested gecko	Correlophus ciliatus	Squamata	Lizard	Canada	America	CB	https://reptile-database.reptarium.cz/species?genus=Correlophus&species=ciliatus&search_param=%28%28common_name%3D%27Correlophus+ciliatus%27%29%29

Table S1: Animal species sampled in this study - country origin and categorization as captive bred (CB), farm bred (FB) and wild-caught (WC)

Shipment No.	Sample batch	Animal species	Scientific name of animal/s	Order	Suborder	Country origin	Continent	Captive bred (CB), farm bred (FB), wild-caught (WC)	Web URL used for assigning animal species to categories CB, FB and WC
83	147	Crested gecko	Correlophus ciliatus	Squamata	Lizard	Canada	America	CB	https://reptile-database.reptarium.cz/species?genus=Correlophus&species=ciliatus&search_param=%28%28common_name%3D%27Correlophus+ciliatus%27%29%29
83	147	Crested gecko	Correlophus ciliatus	Squamata	Lizard	Canada	America	CB	https://reptile-database.reptarium.cz/species?genus=Correlophus&species=ciliatus&search_param=%28%28common_name%3D%27Correlophus+ciliatus%27%29%29
83	147	Crested gecko	Correlophus ciliatus	Squamata	Lizard	Canada	America	CB	https://reptile-database.reptarium.cz/species?genus=Correlophus&species=ciliatus&search_param=%28%28common_name%3D%27Correlophus+ciliatus%27%29%29
83	147	Crested gecko	Correlophus ciliatus	Squamata	Lizard	Canada	America	CB	https://reptile-database.reptarium.cz/species?genus=Correlophus&species=ciliatus&search_param=%28%28common_name%3D%27Correlophus+ciliatus%27%29%29
84	148	Common leopard gecko	Eublepharis macularius	Squamata	Lizard	USA	America	CB	https://reptile-database.reptarium.cz/species?genus=Eublepharis&species=macularius&search_param=%28%28common_name%3D%27Eublepharis+macularius%27%29%29
84	148	Common leopard gecko	Eublepharis macularius	Squamata	Lizard	USA	America	CB	https://reptile-database.reptarium.cz/species?genus=Eublepharis&species=macularius&search_param=%28%28common_name%3D%27Eublepharis+macularius%27%29%29
84	148	Common leopard gecko	Eublepharis macularius	Squamata	Lizard	USA	America	CB	https://reptile-database.reptarium.cz/species?genus=Eublepharis&species=macularius&search_param=%28%28common_name%3D%27Eublepharis+macularius%27%29%29
84	148	Common leopard gecko	Eublepharis macularius	Squamata	Lizard	USA	America	CB	https://reptile-database.reptarium.cz/species?genus=Eublepharis&species=macularius&search_param=%28%28common_name%3D%27Eublepharis+macularius%27%29%29
84	148	Common leopard gecko	Eublepharis macularius	Squamata	Lizard	USA	America	CB	https://reptile-database.reptarium.cz/species?genus=Eublepharis&species=macularius&search_param=%28%28common_name%3D%27Eublepharis+macularius%27%29%29
84	148	Common leopard gecko	Eublepharis macularius	Squamata	Lizard	USA	America	CB	https://reptile-database.reptarium.cz/species?genus=Eublepharis&species=macularius&search_param=%28%28common_name%3D%27Eublepharis+macularius%27%29%29
84	148	Common leopard gecko	Eublepharis macularius	Squamata	Lizard	USA	America	CB	https://reptile-database.reptarium.cz/species?genus=Eublepharis&species=macularius&search_param=%28%28common_name%3D%27Eublepharis+macularius%27%29%29
84	148	Common leopard gecko	Eublepharis macularius	Squamata	Lizard	USA	America	CB	https://reptile-database.reptarium.cz/species?genus=Eublepharis&species=macularius&search_param=%28%28common_name%3D%27Eublepharis+macularius%27%29%29

Table S1: Animal species sampled in this study - country origin and categorization as captive bred (CB), farm bred (FB) and wild-caught (WC)

Shipment No.	Sample batch	Animal species	Scientific name of animal/s	Order	Suborder	Country origin	Continent	Captive bred (CB), farm bred (FB), wild-caught (WC)	Web URL used for assigning animal species to categories CB, FB and WC
84	149	Yarrow's spiny lizard	Sceloporus jarrovii	Squamata	Lizard	USA	America	WC	https://reptile-database.reptarium.cz/species?genus=Sceloporus&species=jarrovii&search_param=%28%28common_name%3D%27Sceloporus+jarrovii%27%29%29
84	149	Yarrow's spiny lizard	Sceloporus jarrovii	Squamata	Lizard	USA	America	WC	https://reptile-database.reptarium.cz/species?genus=Sceloporus&species=jarrovii&search_param=%28%28common_name%3D%27Sceloporus+jarrovii%27%29%29
84	149	Yarrow's spiny lizard	Sceloporus jarrovii	Squamata	Lizard	USA	America	WC	https://reptile-database.reptarium.cz/species?genus=Sceloporus&species=jarrovii&search_param=%28%28common_name%3D%27Sceloporus+jarrovii%27%29%29
84	149	Yarrow's spiny lizard	Sceloporus jarrovii	Squamata	Lizard	USA	America	WC	https://reptile-database.reptarium.cz/species?genus=Sceloporus&species=jarrovii&search_param=%28%28common_name%3D%27Sceloporus+jarrovii%27%29%29
84	150	Side-blotched lizard	Uta stansburiana	Squamata	Lizard	USA	America	WC	https://reptile-database.reptarium.cz/species?genus=Uta&species=stansburiana&search_param=%28%28common_name%3D%27Uta+stansburiana%27%29%29
84	150	Side-blotched lizard	Uta stansburiana	Squamata	Lizard	USA	America	WC	https://reptile-database.reptarium.cz/species?genus=Uta&species=stansburiana&search_param=%28%28common_name%3D%27Uta+stansburiana%27%29%29
84	150	Side-blotched lizard	Uta stansburiana	Squamata	Lizard	USA	America	WC	https://reptile-database.reptarium.cz/species?genus=Uta&species=stansburiana&search_param=%28%28common_name%3D%27Uta+stansburiana%27%29%29
84	150	Side-blotched lizard	Uta stansburiana	Squamata	Lizard	USA	America	WC	https://reptile-database.reptarium.cz/species?genus=Uta&species=stansburiana&search_param=%28%28common_name%3D%27Uta+stansburiana%27%29%29
85	151	Horsefield's tortoise	Testudo horsfieldii	Testudines	Turtle	Ukraine	Europe	FB	https://reptile-database.reptarium.cz/species?genus=Testudo&species=horsfieldii&search_param=%28%28common_name%3D%27Testudo+horsfieldii%27%29%29
85	151	Horsefield's tortoise	Testudo horsfieldii	Testudines	Turtle	Ukraine	Europe	FB	https://reptile-database.reptarium.cz/species?genus=Testudo&species=horsfieldii&search_param=%28%28common_name%3D%27Testudo+horsfieldii%27%29%29
85	151	Horsefield's tortoise	Testudo horsfieldii	Testudines	Turtle	Ukraine	Europe	FB	https://reptile-database.reptarium.cz/species?genus=Testudo&species=horsfieldii&search_param=%28%28common_name%3D%27Testudo+horsfieldii%27%29%29
85	151	Horsefield's tortoise	Testudo horsfieldii	Testudines	Turtle	Ukraine	Europe	FB	https://reptile-database.reptarium.cz/species?genus=Testudo&species=horsfieldii&search_param=%28%28common_name%3D%27Testudo+horsfieldii%27%29%29

Table S1: Animal species sampled in this study - country origin and categorization as captive bred (CB), farm bred (FB) and wild-caught (WC)

Shipment No.	Sample batch	Animal species	Scientific name of animal/s	Order	Suborder	Country origin	Continent	Captive bred (CB), farm bred (FB), wild-caught (WC)	Web URL used for assigning animal species to categories CB, FB and WC
85	151	Horsefield's tortoise	Testudo horsfieldii	Testudines	Turtle	Ukraine	Europe	FB	https://reptile-database.reptarium.cz/species?genus=Testudo&species=horsfieldii&search_param=%28%28common_name%3D%27Testudo+horsfieldii%27%29%29
85	151	Horsefield's tortoise	Testudo horsfieldii	Testudines	Turtle	Ukraine	Europe	FB	https://reptile-database.reptarium.cz/species?genus=Testudo&species=horsfieldii&search_param=%28%28common_name%3D%27Testudo+horsfieldii%27%29%29
85	151	Horsefield's tortoise	Testudo horsfieldii	Testudines	Turtle	Ukraine	Europe	FB	https://reptile-database.reptarium.cz/species?genus=Testudo&species=horsfieldii&search_param=%28%28common_name%3D%27Testudo+horsfieldii%27%29%29
85	152	Veiled chameleon	Chamaeleo calyptratus	Squamata	Lizard	Ukraine	Europe	CB	https://reptile-database.reptarium.cz/species?genus=Chamaeleo&species=calyptratus&search_param=%28%28common_name%3D%27Chamaeleo+calyptratus%27%29%29
85	152	Veiled chameleon	Chamaeleo calyptratus	Squamata	Lizard	Ukraine	Europe	CB	https://reptile-database.reptarium.cz/species?genus=Chamaeleo&species=calyptratus&search_param=%28%28common_name%3D%27Chamaeleo+calyptratus%27%29%29
85	152	Veiled chameleon	Chamaeleo calyptratus	Squamata	Lizard	Ukraine	Europe	CB	https://reptile-database.reptarium.cz/species?genus=Chamaeleo&species=calyptratus&search_param=%28%28common_name%3D%27Chamaeleo+calyptratus%27%29%29
85	152	Veiled chameleon	Chamaeleo calyptratus	Squamata	Lizard	Ukraine	Europe	CB	https://reptile-database.reptarium.cz/species?genus=Chamaeleo&species=calyptratus&search_param=%28%28common_name%3D%27Chamaeleo+calyptratus%27%29%29
86	153	Horsefield's tortoise	Testudo horsfieldii	Testudines	Turtle	Uzbekistan	Asia	FB	https://reptile-database.reptarium.cz/species?genus=Testudo&species=horsfieldii&search_param=%28%28common_name%3D%27Testudo+horsfieldii%27%29%29
86	153	Horsefield's tortoise	Testudo horsfieldii	Testudines	Turtle	Uzbekistan	Asia	FB	https://reptile-database.reptarium.cz/species?genus=Testudo&species=horsfieldii&search_param=%28%28common_name%3D%27Testudo+horsfieldii%27%29%29
86	153	Horsefield's tortoise	Testudo horsfieldii	Testudines	Turtle	Uzbekistan	Asia	FB	https://reptile-database.reptarium.cz/species?genus=Testudo&species=horsfieldii&search_param=%28%28common_name%3D%27Testudo+horsfieldii%27%29%29
86	153	Horsefield's tortoise	Testudo horsfieldii	Testudines	Turtle	Uzbekistan	Asia	FB	https://reptile-database.reptarium.cz/species?genus=Testudo&species=horsfieldii&search_param=%28%28common_name%3D%27Testudo+horsfieldii%27%29%29
86	153	Horsefield's tortoise	Testudo horsfieldii	Testudines	Turtle	Uzbekistan	Asia	FB	https://reptile-database.reptarium.cz/species?genus=Testudo&species=horsfieldii&search_param=%28%28common_name%3D%27Testudo+horsfieldii%27%29%29

Table S1: Animal species sampled in this study - country origin and categorization as captive bred (CB), farm bred (FB) and wild-caught (WC)

Shipment No.	Sample batch	Animal species	Scientific name of animal/s	Order	Suborder	Country origin	Continent	Captive bred (CB), farm bred (FB), wild-caught (WC)	Web URL used for assigning animal species to categories CB, FB and WC
87	154	Horsefield's tortoise	Testudo horsfieldii	Testudines	Turtle	Ukraine	Europe	FB	https://reptile-database.reptarium.cz/species?genus=Testudo&species=horsfieldii&search_param=%28%28common_name%3D%27Testudo+horsfieldii%27%29%29
87	154	Horsefield's tortoise	Testudo horsfieldii	Testudines	Turtle	Ukraine	Europe	FB	https://reptile-database.reptarium.cz/species?genus=Testudo&species=horsfieldii&search_param=%28%28common_name%3D%27Testudo+horsfieldii%27%29%29
87	154	Horsefield's tortoise	Testudo horsfieldii	Testudines	Turtle	Ukraine	Europe	FB	https://reptile-database.reptarium.cz/species?genus=Testudo&species=horsfieldii&search_param=%28%28common_name%3D%27Testudo+horsfieldii%27%29%29
87	154	Horsefield's tortoise	Testudo horsfieldii	Testudines	Turtle	Ukraine	Europe	FB	https://reptile-database.reptarium.cz/species?genus=Testudo&species=horsfieldii&search_param=%28%28common_name%3D%27Testudo+horsfieldii%27%29%29
87	154	Horsefield's tortoise	Testudo horsfieldii	Testudines	Turtle	Ukraine	Europe	FB	https://reptile-database.reptarium.cz/species?genus=Testudo&species=horsfieldii&search_param=%28%28common_name%3D%27Testudo+horsfieldii%27%29%29
87	154	Horsefield's tortoise	Testudo horsfieldii	Testudines	Turtle	Ukraine	Europe	FB	https://reptile-database.reptarium.cz/species?genus=Testudo&species=horsfieldii&search_param=%28%28common_name%3D%27Testudo+horsfieldii%27%29%29
87	154	Horsefield's tortoise	Testudo horsfieldii	Testudines	Turtle	Ukraine	Europe	FB	https://reptile-database.reptarium.cz/species?genus=Testudo&species=horsfieldii&search_param=%28%28common_name%3D%27Testudo+horsfieldii%27%29%29
87	154	Horsefield's tortoise	Testudo horsfieldii	Testudines	Turtle	Ukraine	Europe	FB	https://reptile-database.reptarium.cz/species?genus=Testudo&species=horsfieldii&search_param=%28%28common_name%3D%27Testudo+horsfieldii%27%29%29
87	154	Horsefield's tortoise	Testudo horsfieldii	Testudines	Turtle	Ukraine	Europe	FB	https://reptile-database.reptarium.cz/species?genus=Testudo&species=horsfieldii&search_param=%28%28common_name%3D%27Testudo+horsfieldii%27%29%29
87	154	Horsefield's tortoise	Testudo horsfieldii	Testudines	Turtle	Ukraine	Europe	FB	https://reptile-database.reptarium.cz/species?genus=Testudo&species=horsfieldii&search_param=%28%28common_name%3D%27Testudo+horsfieldii%27%29%29
87	154	Horsefield's tortoise	Testudo horsfieldii	Testudines	Turtle	Ukraine	Europe	FB	https://reptile-database.reptarium.cz/species?genus=Testudo&species=horsfieldii&search_param=%28%28common_name%3D%27Testudo+horsfieldii%27%29%29
87	154	Horsefield's tortoise	Testudo horsfieldii	Testudines	Turtle	Ukraine	Europe	FB	https://reptile-database.reptarium.cz/species?genus=Testudo&species=horsfieldii&search_param=%28%28common_name%3D%27Testudo+horsfieldii%27%29%29

Table S1: Animal species sampled in this study - country origin and categorization as captive bred (CB), farm bred (FB) and wild-caught (WC)

Shipment No.	Sample batch	Animal species	Scientific name of animal/s	Order	Suborder	Country origin	Continent	Captive bred (CB), farm bred (FB), wild-caught (WC)	Web URL used for assigning animal species to categories CB, FB and WC
87	154	Horsefield's tortoise	Testudo horsfieldii	Testudines	Turtle	Ukraine	Europe	FB	https://reptile-database.reptarium.cz/species?genus=Testudo&species=horsfieldii&search_param=%28%28common_name%3D%27Testudo+horsfieldii%27%29%29
87	154	Horsefield's tortoise	Testudo horsfieldii	Testudines	Turtle	Ukraine	Europe	FB	https://reptile-database.reptarium.cz/species?genus=Testudo&species=horsfieldii&search_param=%28%28common_name%3D%27Testudo+horsfieldii%27%29%29
87	154	Horsefield's tortoise	Testudo horsfieldii	Testudines	Turtle	Ukraine	Europe	FB	https://reptile-database.reptarium.cz/species?genus=Testudo&species=horsfieldii&search_param=%28%28common_name%3D%27Testudo+horsfieldii%27%29%29
87	154	Horsefield's tortoise	Testudo horsfieldii	Testudines	Turtle	Ukraine	Europe	FB	https://reptile-database.reptarium.cz/species?genus=Testudo&species=horsfieldii&search_param=%28%28common_name%3D%27Testudo+horsfieldii%27%29%29
87	155	Veiled chameleon	Chamaeleo calyptratus	Squamata	Lizard	Ukraine	Europe	CB	https://reptile-database.reptarium.cz/species?genus=Chamaeleo&species=calyptratus&search_param=%28%28common_name%3D%27Chamaeleo+calyptratus%27%29%29
87	155	Veiled chameleon	Chamaeleo calyptratus	Squamata	Lizard	Ukraine	Europe	CB	https://reptile-database.reptarium.cz/species?genus=Chamaeleo&species=calyptratus&search_param=%28%28common_name%3D%27Chamaeleo+calyptratus%27%29%29
87	155	Veiled chameleon	Chamaeleo calyptratus	Squamata	Lizard	Ukraine	Europe	CB	https://reptile-database.reptarium.cz/species?genus=Chamaeleo&species=calyptratus&search_param=%28%28common_name%3D%27Chamaeleo+calyptratus%27%29%29
87	155	Veiled chameleon	Chamaeleo calyptratus	Squamata	Lizard	Ukraine	Europe	CB	https://reptile-database.reptarium.cz/species?genus=Chamaeleo&species=calyptratus&search_param=%28%28common_name%3D%27Chamaeleo+calyptratus%27%29%29
88	156	Horsefield's tortoise	Testudo horsfieldii	Testudines	Turtle	Macedonia	Europe	FB	https://reptile-database.reptarium.cz/species?genus=Testudo&species=horsfieldii&search_param=%28%28common_name%3D%27Testudo+horsfieldii%27%29%29
88	156	Horsefield's tortoise	Testudo horsfieldii	Testudines	Turtle	Macedonia	Europe	FB	https://reptile-database.reptarium.cz/species?genus=Testudo&species=horsfieldii&search_param=%28%28common_name%3D%27Testudo+horsfieldii%27%29%29
88	156	Horsefield's tortoise	Testudo horsfieldii	Testudines	Turtle	Macedonia	Europe	FB	https://reptile-database.reptarium.cz/species?genus=Testudo&species=horsfieldii&search_param=%28%28common_name%3D%27Testudo+horsfieldii%27%29%29
88	156	Horsefield's tortoise	Testudo horsfieldii	Testudines	Turtle	Macedonia	Europe	FB	https://reptile-database.reptarium.cz/species?genus=Testudo&species=horsfieldii&search_param=%28%28common_name%3D%27Testudo+horsfieldii%27%29%29

Table S1: Animal species sampled in this study - country origin and categorization as captive bred (CB), farm bred (FB) and wild-caught (WC)

Shipment No.	Sample batch	Animal species	Scientific name of animal/s	Order	Suborder	Country origin	Continent	Captive bred (CB), farm bred (FB), wild-caught (WC)	Web URL used for assigning animal species to categories CB, FB and WC
88	156	Horsefield's tortoise	Testudo horsfieldii	Testudines	Turtle	Macedonia	Europe	FB	https://reptile-database.reptarium.cz/species?genus=Testudo&species=horsfieldii&search_param=%28%28common_name%3D%27Testudo+horsfieldii%27%29%29
88	156	Horsefield's tortoise	Testudo horsfieldii	Testudines	Turtle	Macedonia	Europe	FB	https://reptile-database.reptarium.cz/species?genus=Testudo&species=horsfieldii&search_param=%28%28common_name%3D%27Testudo+horsfieldii%27%29%29
88	156	Horsefield's tortoise	Testudo horsfieldii	Testudines	Turtle	Macedonia	Europe	FB	https://reptile-database.reptarium.cz/species?genus=Testudo&species=horsfieldii&search_param=%28%28common_name%3D%27Testudo+horsfieldii%27%29%29
89	157	Common green iguana	Iguana iguana	Squamata	Lizard	El Salvador	America	FB	https://reptile-database.reptarium.cz/species?genus=Iguana&species=iguana&search_param=%28%28common_name%3D%27Iguana+iguana%27%29%29
89	157	Common green iguana	Iguana iguana	Squamata	Lizard	El Salvador	America	FB	https://reptile-database.reptarium.cz/species?genus=Iguana&species=iguana&search_param=%28%28common_name%3D%27Iguana+iguana%27%29%29
89	157	Common green iguana	Iguana iguana	Squamata	Lizard	El Salvador	America	FB	https://reptile-database.reptarium.cz/species?genus=Iguana&species=iguana&search_param=%28%28common_name%3D%27Iguana+iguana%27%29%29
89	157	Common green iguana	Iguana iguana	Squamata	Lizard	El Salvador	America	FB	https://reptile-database.reptarium.cz/species?genus=Iguana&species=iguana&search_param=%28%28common_name%3D%27Iguana+iguana%27%29%29
89	157	Common green iguana	Iguana iguana	Squamata	Lizard	El Salvador	America	FB	https://reptile-database.reptarium.cz/species?genus=Iguana&species=iguana&search_param=%28%28common_name%3D%27Iguana+iguana%27%29%29
90	158	Horsefield's tortoise	Testudo horsfieldii	Testudines	Turtle	Uzbekistan	Asia	FB	https://reptile-database.reptarium.cz/species?genus=Testudo&species=horsfieldii&search_param=%28%28common_name%3D%27Testudo+horsfieldii%27%29%29
90	158	Horsefield's tortoise	Testudo horsfieldii	Testudines	Turtle	Uzbekistan	Asia	FB	https://reptile-database.reptarium.cz/species?genus=Testudo&species=horsfieldii&search_param=%28%28common_name%3D%27Testudo+horsfieldii%27%29%29
90	158	Horsefield's tortoise	Testudo horsfieldii	Testudines	Turtle	Uzbekistan	Asia	FB	https://reptile-database.reptarium.cz/species?genus=Testudo&species=horsfieldii&search_param=%28%28common_name%3D%27Testudo+horsfieldii%27%29%29
90	158	Horsefield's tortoise	Testudo horsfieldii	Testudines	Turtle	Uzbekistan	Asia	FB	https://reptile-database.reptarium.cz/species?genus=Testudo&species=horsfieldii&search_param=%28%28common_name%3D%27Testudo+horsfieldii%27%29%29

Table S1: Animal species sampled in this study - country origin and categorization as captive bred (CB), farm bred (FB) and wild-caught (WC)

Shipment No.	Sample batch	Animal species	Scientific name of animal/s	Order	Suborder	Country origin	Continent	Captive bred (CB), farm bred (FB), wild-caught (WC)	Web URL used for assigning animal species to categories CB, FB and WC
90	158	Horsefield's tortoise	Testudo horsfieldii	Testudines	Turtle	Uzbekistan	Asia	FB	https://reptile-database.reptarium.cz/species?genus=Testudo&species=horsfieldii&search_param=%28%28common_name%3D%27Testudo+horsfieldii%27%29%29
91	159	Central bearded dragon	Pogona vitticeps	Squamata	Lizard	Ukraine	Europe	CB	https://reptile-database.reptarium.cz/species?genus=Pogona&species=vitticeps&search_param=%28%28common_name%3D%27Pogona+vitticeps%27%29%29
91	159	Central bearded dragon	Pogona vitticeps	Squamata	Lizard	Ukraine	Europe	CB	https://reptile-database.reptarium.cz/species?genus=Pogona&species=vitticeps&search_param=%28%28common_name%3D%27Pogona+vitticeps%27%29%29
91	159	Central bearded dragon	Pogona vitticeps	Squamata	Lizard	Ukraine	Europe	CB	https://reptile-database.reptarium.cz/species?genus=Pogona&species=vitticeps&search_param=%28%28common_name%3D%27Pogona+vitticeps%27%29%29
91	159	Central bearded dragon	Pogona vitticeps	Squamata	Lizard	Ukraine	Europe	CB	https://reptile-database.reptarium.cz/species?genus=Pogona&species=vitticeps&search_param=%28%28common_name%3D%27Pogona+vitticeps%27%29%29
91	159	Central bearded dragon	Pogona vitticeps	Squamata	Lizard	Ukraine	Europe	CB	https://reptile-database.reptarium.cz/species?genus=Pogona&species=vitticeps&search_param=%28%28common_name%3D%27Pogona+vitticeps%27%29%29
91	159	Central bearded dragon	Pogona vitticeps	Squamata	Lizard	Ukraine	Europe	CB	https://reptile-database.reptarium.cz/species?genus=Pogona&species=vitticeps&search_param=%28%28common_name%3D%27Pogona+vitticeps%27%29%29
91	159	Central bearded dragon	Pogona vitticeps	Squamata	Lizard	Ukraine	Europe	CB	https://reptile-database.reptarium.cz/species?genus=Pogona&species=vitticeps&search_param=%28%28common_name%3D%27Pogona+vitticeps%27%29%29
91	159	Central bearded dragon	Pogona vitticeps	Squamata	Lizard	Ukraine	Europe	CB	https://reptile-database.reptarium.cz/species?genus=Pogona&species=vitticeps&search_param=%28%28common_name%3D%27Pogona+vitticeps%27%29%29
92	160	Red-footed tortoise	Chelonoidis carbonarius	Testudines	Turtle	Brazil	America	WC/FB	https://reptile-database.reptarium.cz/species?genus=Chelonoidis&species=carbonarius&search_param=%28%28common_name%3D%27Chelonoidis+carbonarius%27%29%29
92	160	Red-footed tortoise	Chelonoidis carbonarius	Testudines	Turtle	Brazil	America	WC/FB	https://reptile-database.reptarium.cz/species?genus=Chelonoidis&species=carbonarius&search_param=%28%28common_name%3D%27Chelonoidis+carbonarius%27%29%29
92	160	Red-footed tortoise	Chelonoidis carbonarius	Testudines	Turtle	Brazil	America	WC/FB	https://reptile-database.reptarium.cz/species?genus=Chelonoidis&species=carbonarius&search_param=%28%28common_name%3D%27Chelonoidis+carbonarius%27%29%29

Table S1: Animal species sampled in this study - country origin and categorization as captive bred (CB), farm bred (FB) and wild-caught (WC)

Shipment No.	Sample batch	Animal species	Scientific name of animal/s	Order	Suborder	Country origin	Continent	Captive bred (CB), farm bred (FB), wild-caught (WC)	Web URL used for assigning animal species to categories CB, FB and WC
92	160	Red-footed tortoise	Chelonoidis carbonarius	Testudines	Turtle	Brazil	America	WC/FB	https://reptile-database.reptarium.cz/species?genus=Chelonoidis&species=carbonarius&search_param=%28%28common_name%3D%27Chelonoidis+carbonarius%27%29%29
92	160	Red-footed tortoise	Chelonoidis carbonarius	Testudines	Turtle	Brazil	America	WC/FB	https://reptile-database.reptarium.cz/species?genus=Chelonoidis&species=carbonarius&search_param=%28%28common_name%3D%27Chelonoidis+carbonarius%27%29%29
