# Supplementary material for: Imported Pet Reptiles and Their “Blind Passengers”—In-Depth Characterization of 80 Acinetobacter Species Isolates
Source: Microorganisms. 2022 Apr 24;10(5):893. doi: 10.3390/microorganisms10050893 (PMC9144363; doi:10.3390/microorganisms10050893)
Supplement: Supplementary file 1 [file microorganisms-10-00893-s001.zip › Suppl. Table S7_Acc-Nos_Ab genomes-25-03-22.pdf]

Table S7: NCBI Reference numbers of *A. baumannii* genomes included in Figure 2 of the main text.

Strain name	NCBI Reference Sequence	International Clone (IC)
AYE	NC_010410.1	IC1
AB0057	NC_011586.2	IC1
1656-2	NC_017162.1	IC2
MDR-TJ	NC_017847.1	IC2
XH386	NZ_CP021326.1	IC2
Naval-81	NZ_AFDB02000005.1	IC3
OIFC137	NZ_AFDK01000004.1	IC3
NIPH 1669	NZ_APOQ00000000.1	IC3
Ab825	NZ_NTFR00000000.1	IC4
UKK_0004	ERR1226902 (Sequence Read Archive)	IC4
NIPH 1734	NZ_APOX00000000.1	IC4
11510	NZ_CP018861.2	IC5
AB030	NZ_CP009257.1	IC5
AF-401	NZ_CP018254.1	IC5
Ab-3909	AEOZ00000000.1	IC6
AB4332	RJLV00000000.1	IC6
3365	WIVN00000000.1	IC6
OCU_Ac2	NZ_BHFY00000000.1	IC7
161/07	NZ_JZCA00000000.1	IC7
4300STDY7045893	NZ_UFPC00000000.1	IC7
LUH6220	NZ_JZBW00000000.1	IC7
XH693	NZ_LYIY00000000.1	IC8
LAC-4	NU_CP007712.1	IC8
MRSN15574	NZ_VHGP00000000.1	IC9
AC-20	NZ_JABESZ000000000.1	IC9
MRSN14427	NZ_VHGX00000000.1	not assigned
SP816	NZ_JAAGTY000000000.1	not assigned
BA22685	NZ_JAAOQK000000000.1	not assigned
R20	NZ_PUDN00000000.1	not assigned
571B5_12EESBL	NZ_CACSGY000000000.1	not assigned
6200	NZ_CP010397.1	not assigned
PG20180064	NZ_CP043180.1	not assigned
4300STDY7045766	NZ_UFKQ00000000.1	not assigned
