# Supplementary figures and images for: Imported Pet Reptiles and Their “Blind Passengers”—In-Depth Characterization of 80 Acinetobacter Species Isolates
Source: Microorganisms. 2022 Apr 24;10(5):893. doi: 10.3390/microorganisms10050893 (PMC9144363; doi:10.3390/microorganisms10050893)

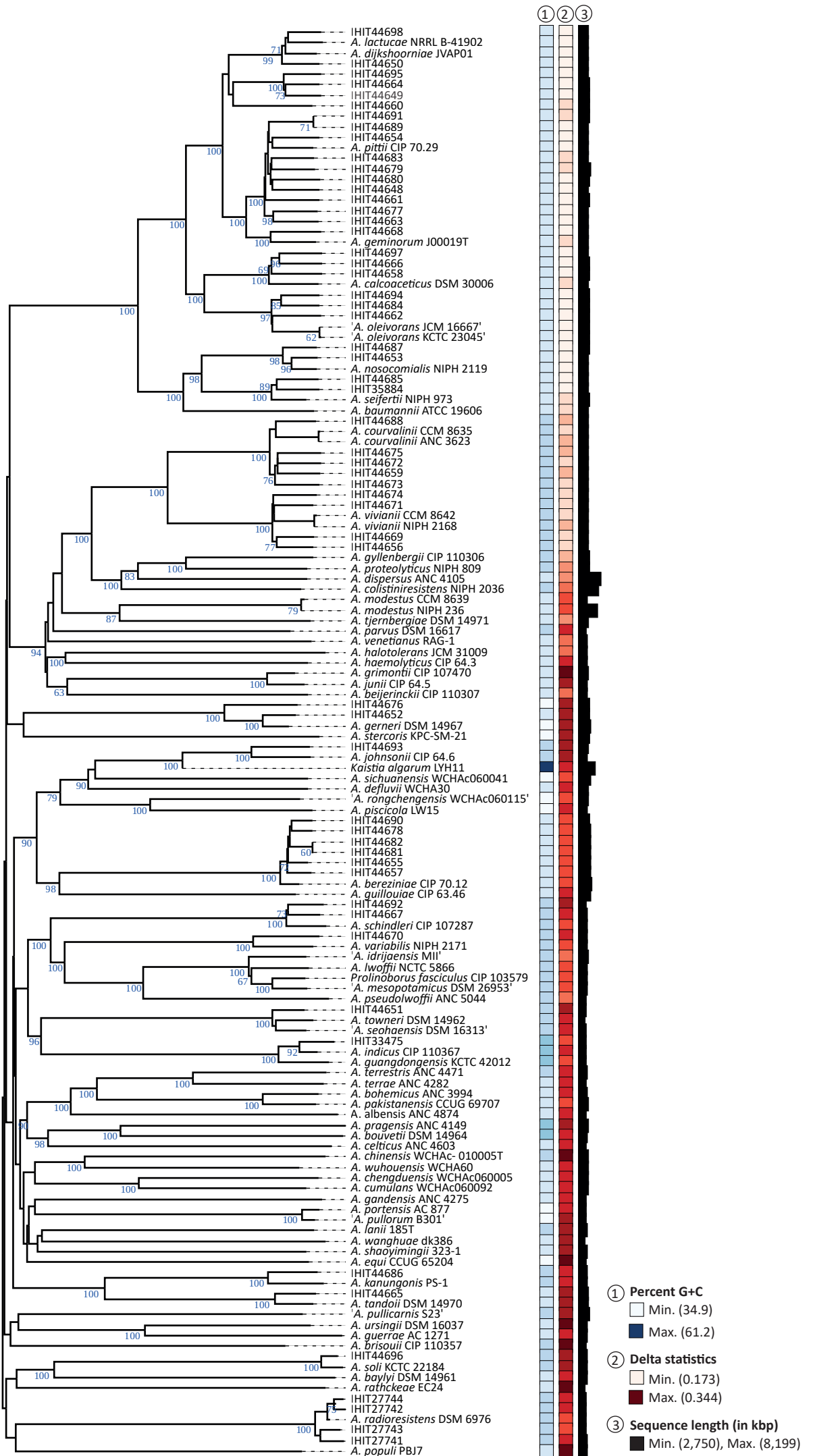

Supplement: Supplementary file 1 [file microorganisms-10-00893-s001.zip › Suppl. Figure S1-TYGS_TREE-Spezies-Einteilung.pdf]
